# An Isolated Lithium *ortho*-Carboranyl Cuprate Complex for the Synthesis of Multiple-Carborane-Substituted Arenes from (Hetero)Aryl Bromides and Chlorides

**DOI:** 10.1021/jacs.5c13004

**Published:** 2025-10-01

**Authors:** Yusei Hisata, Daina Morishita, Yoichi Hoshimoto

**Affiliations:** † Department of Applied Chemistry, Graduate School of Engineering, 13013 The University of Osaka, Suita, Osaka 565-0871, Japan; ‡ Center for Future Innovation (CFi), Graduate School of Engineering, The University of Osaka, Suita, Osaka 565-0871, Japan

## Abstract

Carborane-substituted arenes have emerged as versatile building blocks in medicinal chemistry, materials science, and coordination chemistry owing to the unique three-dimensional aromaticity, exceptional stability, and bioisosteric properties of carboranes. However, the existing synthetic routes to carborane-substituted arenes via C–C bond formation often rely on complex and laborious *in situ* procedures using aryl iodides, thus severely limiting the scope of their practical applications. Here, we report the isolation and characterization of a lithium bis­(*o*-carboran-1-yl)­cuprate complex (**Li/Cu-1**) that enables the efficient “dump-and-stir” synthesis of carborane-substituted arenes from readily available aryl bromides and chlorides. Remarkably, isophthalonitrile functions as a ligand for the Li center in **Li/Cu-1**, leaving the Cu center available for the oxidative addition to aryl halides. This represents a paradigm shift from traditional approaches using pyridines as ligands for Cu centers. Our method provides access to a diverse array of unprecedented molecules, including multiple-carborane-substituted or carborane-fused arenes, thereby converting a hitherto difficult transformation in synthetic chemistry into a practical and scalable process.

## Graphical abstract

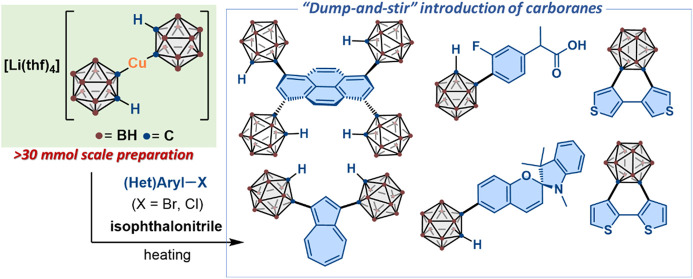

## Introduction

Achieving operational simplicity in molecular synthesis is a key goal in modern synthetic chemistry. Simple and operationally facile methods using well-characterized isolated reagents can considerably expand the scope and applicability of reported synthetic methods by reducing the complex experimental manipulations required for the *in situ* preparation of reactive species. Such “dump-and-stir” methods are highly desirable, especially for introducing expensive but multifunctional substituents into versatile molecular frameworks. For example, the synthesis of (hetero)­arenes bearing *closo*-1,2-carborane (*o*-carborane, *o*-1,2-C_2_B_10_H_12_; Figure 1a) via C–C bond formation using isolable and readily available carboranyl-metal nucleophiles and (hetero)­aryl electrophiles is highly practical. Such *C*-arylated *o*-carborane derivatives have been extensively applied in diverse fields, including medicinal, materials, and coordination chemistry,^1–13^ owing to the distinctive properties of carboranes, such as neutron absorption, high thermal stability, high chemical stability toward oxidants and strong acids, three-dimensional electron delocalization (3D aromaticity),^14^ remarkable hydrophobicity, and comparable pseudospherical size to both adamantane and the effective spherical volume occupied by a rotating phenyl ring (i.e., bioisosterism).^1,15^ Given that a variety of biphenyl-based bioactive compounds have found applications in our daily lives,^16^ strategic bioisosteric replacement of one of their phenyl groups with carboranes is worth exploring in the interest of pursuing underexplored molecular applications.^17–19^ Unfortunately, hitherto reported synthetic procedures are characterized by a severely limited range of accessible (*o*-carboran-1-yl)­arenes.

**Figure 1:**
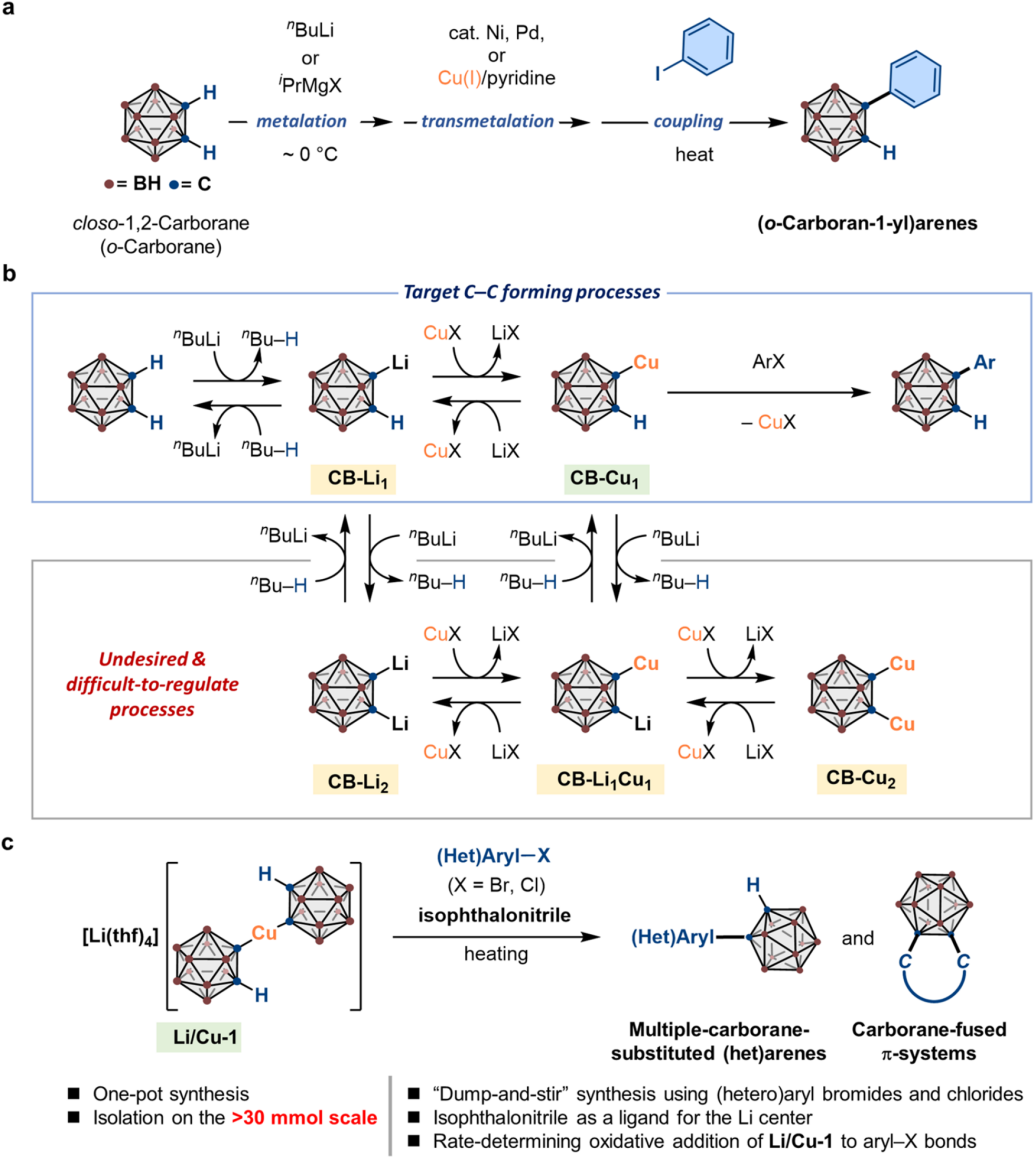
Synthesis of (*o*-carboran-1-yl)­arenes via C–C bond formation. (a) Schematic representation of reported *in situ* procedures using *o*-carborane and aryl iodides. (b) Selected species proposed to be generated *in situ* under Ullmann-type conditions using ^*n*^BuLi and Cu­(I) salts. X represents halides. (c) This work: a ’dump-and-stir’ synthesis of multiple-carborane-substituted or carborane-fused (hetero)­arenes.

Several pioneering methods have been developed for the synthesis of (*o*-carboran-1-yl)­arenes via C–C bond formation between aryl iodides and *in situ*-generated *o*-carboranyl-metal (i.e., Cu, Ni, Pd) species from commercially available *o*-carborane (Figure 1a).^20–26^ These methods constitute alternative approaches to access this class of compounds without using toxic and explosive decaborane (B_10_H_14_) under harsh conditions.^2,3,6,27^ However, these transition-metal-mediated processes usually involve laborious multistep manipulations, including the *in situ* generation of active *o*-carboranyl-lithium or -magnesium species at low temperatures in ethereal solvents (e.g., THF or 1,2-dimethoxyethane (DME)) and the transmetalation with transition metals followed by coupling with aryl iodides, which has prevented extending these approaches to more synthetically accessible aryl bromides and chlorides and often result in poor reproducibility. For example, to achieve an efficient C–C bond formation between carboranyl-copper species **CB-Cu**_**1**_, which remains elusive,^28^ and aryl halides via the so-called Ullman-type mechanism,^29,30^ the equilibrium involving **CB-Cu**_**1**_, carboranyl-lithium species (**CB-Li**_**1**_ and **CB-Li**_**2**_),^31^ and other copper species (**CB-Li**_**1**_ **Cu**_**1**_ and **CB-Cu**_**2**_) should be precisely regulated to avoid undesired side reactions such as the formation of bis-*o*-carborane triggered by **CB-Cu**_**2**_ (Figure 1b).^20,21^ Nevertheless, the presence of salts (LiX and/or CuX) in the equilibrium renders controlling these reactions difficult, and their complete removal during the aforementioned multistep procedures is challenging. In addition, **CB-Cu**_**1**_ cannot be expected to be sufficiently stable under the hitherto reported reaction conditions, as evident from the low to moderate efficiency described in previous reports.

During our investigations to expand the library of triarylboranes,^32,33^ we examined the incorporation of multiple *o*-carboranyl groups in single arenes using readily available CuCl and 1,4-dibromobenzene (**A1**) as a model substrate in the presence of pyridine, which has been proposed as a suitable ligand for **CB-Cu**_**1**_ species by Wade and co-workers.^21^ However, these reactions are complicated, and 1-bromo-4-(*o*-carboran-1-yl)­benzene (**C1**) was predominantly obtained in <30% yield, even after an extensive screening of conditions (Figure S31), while the yield of the targeted 1,4-bis­(*o*-carboran-1-yl)­benzene (**B1**) remained <5% (for the structures of **B1** and **C1**, see Figure 2b). A nickel-catalyzed procedure reported by Xie and co-workers^34^ was also unsuccessful under the applied conditions using **A1** (Figure S32). Therefore, we envisioned that a dump-and-stir method using an isolated *o*-carboranyl-copper reagent could efficiently afford multiple-carborane-substituted arenes from readily available aryl halides (Figure 1c). Herein, we report the development of a decagram-scale (>30 mmol) synthesis of lithium bis­(*o*-carboran-1-yl)­cuprate **Li/Cu-1**, which proved to be sufficiently reactive in C–C bond-forming reactions with a variety of (hetero)­aryl bromides and chlorides. Moreover, we demonstrate the key role of isophthalonitrile as a ligand for the Li center in **Li/Cu-1** to effectively stabilize a transition state for the rate-determining oxidative addition step; whereas pyridine- or phosphine-derived ligands, which tend to coordinate to the Cu center, are unsuitable for this reaction. We also performed preliminary mechanistic studies on the Ullmann-type coupling involving **Li/Cu-1**.

**Figure 2:**
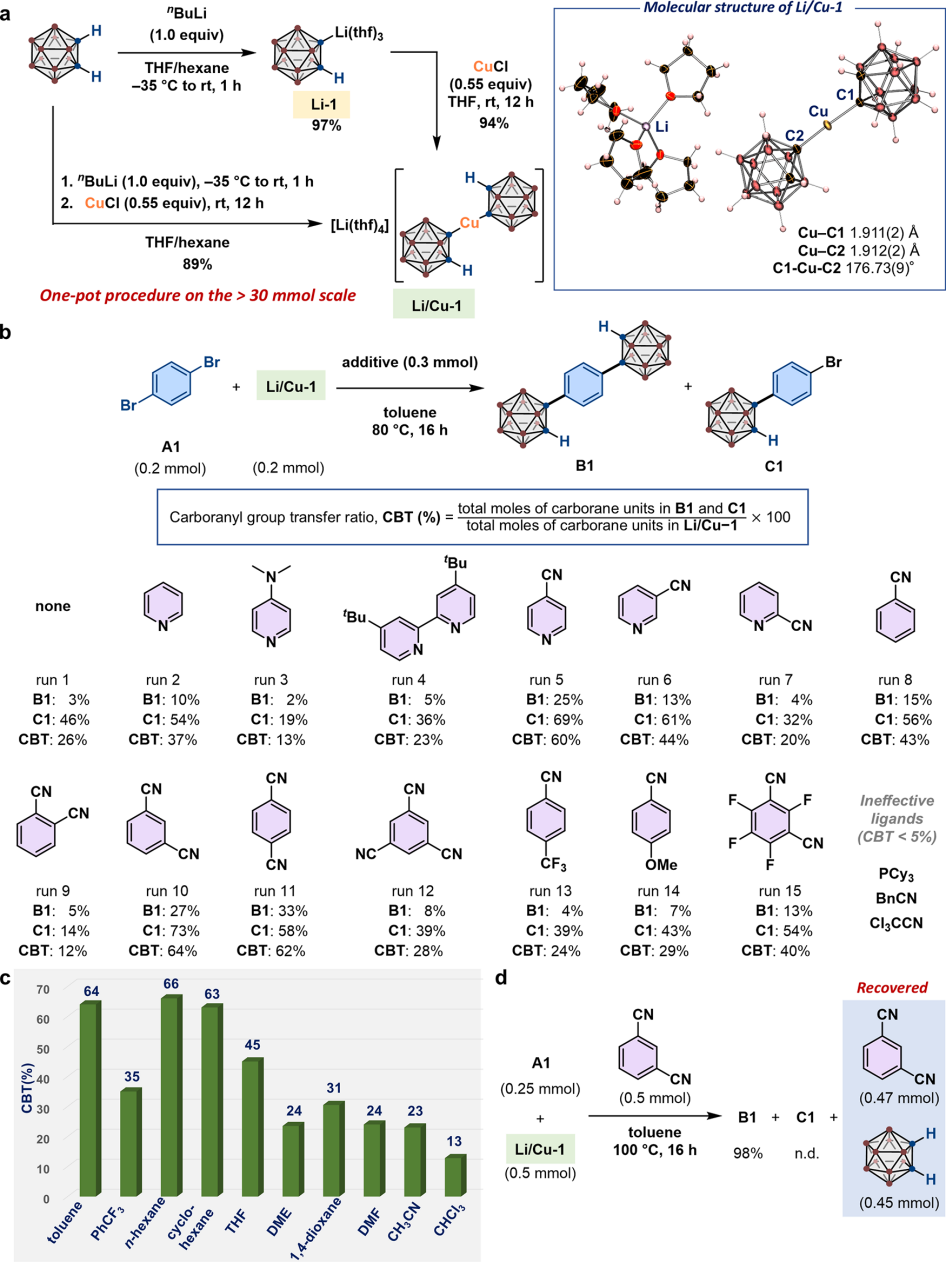
Isolation and application of lithium bis­(*o*-carboran-1-yl)­cuprate **Li/Cu-1**. (a) Synthesis of **Li/Cu-1**. The molecular structure of **Li/Cu-1** in the crystalline state was determined by an SC-XRD analysis (thermal ellipsoids are shown at 30% probability). (b) Ligand screening for the coupling reaction between **A1** and **Li/Cu-1**. (c) Solvent effects. (d) Optimized reaction conditions for the synthesis of **B1**. Isolated yields are shown. For carborane or carboranyl groups, brown spheres represent B–H units while blue spheres represent C atoms, as shown in Figure 1a.

## Results and Discussion

To establish a dump-and-stir method for the efficient synthesis of multiple-carborane-substituted arenes, a readily isolable and well-characterized reagent containing the *o*-carboranyl group is essential. To this end, we initially isolated (*o*-carboran-1-yl)­Li­(thf)_3_ (**Li-1**) (Figure 2a), given that the *in situ* generation of carboranyl-lithium species and subsequent carborane transfer is a common procedure for constructing chemical bonds between *o*-carborane and electrophiles. The molecular structure of **Li-1** was unambiguously confirmed by a single-crystal X-ray diffraction (SC-XRD) analysis (Figure S4). It should be also noted that an identical structure has previously been proposed for **Li-1** by Willans and co-workers based on calculated NMR data.^31^ **Li-1** was isolated as a white powder that can be easily handled, whereas isolation of the corresponding **CB-Li** species bearing DME was unsuccessful. Subsequently, we mixed **Li-1** with CuCl (1.1 equiv) in THF at room temperature, assuming that these compounds would react in a 1:1 stoichiometry to furnish the **CB-Cu_1_** species shown in Figure 1b. However, we eventually confirmed the formation of unprecedented lithium bis­(*o*-carboran-1-yl)­cuprate complex **Li/Cu-1**, which was isolated in 94% yield as a brownish solid after optimizing the stoichiometry of CuCl to 0.55 equiv with respect to **Li-1**.^35^ Finally, we established a straightforward one-pot procedure to synthesize **Li/Cu-1** on a decagram scale (19.9 g, 30.9 mmol, 89% isolated yield) using *o*-carborane (69.3 mmol), ^*n*^BuLi (1.0 equiv), and CuCl (0.55 equiv). The molecular structure of **Li/Cu-1** was determined via multinuclear NMR and SC-XRD analyses (Figure 2a, right). The Cu center in **Li/Cu-1** exhibits a linear geometry with a C1–Cu–C2 angle of 176.73(9)° and is located almost equidistant between the C1 and C2 atoms, whereby the Cu–C1 and Cu–C2 bonds are approximately 1.91 Å. **Li/Cu-1** is stable under an inert-gas atmosphere, showing no observable decomposition after one month, although it decomposes rapidly in the presence of air/moisture. It dissolves in CH_2_Cl_2_ and ethereal solvents such as THF, DME, and 1,4-dioxane, whereas it exhibits limited solubility in aromatic hydrocarbon solvents such as toluene and PhCF_3_. On the basis of these properties, we concluded that **Li/Cu-1** could potentially serve as a useful reagent for introducing *o*-carboranyl groups into arenes using a dump-and-stir method.

Employing the isolated **Li/Cu-1** reagent facilitated the rapid and thorough optimization of the reaction conditions using **A1** as a model substrate (Figure 2b). In the hitherto reported Cu-mediated Ullmann-type coupling reactions for the synthesis of (*o*-carboran-1-yl)­arenes, pyridines have been predominantly used as ligands for the Cu center.^20,21^ In fact, we confirmed that the carboranyl-group transfer ratio (CBT; Figure 2b), for which a value of 100% means that two carboranyl groups in a single **Li/Cu-1** molecule are transferred to give **B1**, increased to 37% in the presence of pyridine (yield of **B1** = 10%, yield of **C1** = 54%; run 2) compared to the results without additives (CBT = 26%; run 1). Nevertheless, electron-rich pyridine derivatives such as 4-dimethylaminopyridine (CBT = 13%; run 3) and 4,4′-di-*tert*-butyl-2,2′-bipyridine (CBT = 23%; run 4) did not lead to improved results. Remarkably, the CBT value was significantly enhanced to 60% when 4-cyanopyridine was employed, affording **B1** and **C1** in 25% and 69% yield, respectively (run 5), demonstrating that both carboranyl groups in **Li/Cu-1** can be transferred to electrophiles. We therefore explored the effectiveness of 3- and 2-cyano-substituted pyridines (runs 6 and 7); however, the CBT values decreased in both cases, although 3-cyanopyridine still afforded better results (CBT = 44%, yield of **B1** = 13%, yield of **C1** = 61%) than pyridine (run 2). Subsequently, we performed a control experiment using benzonitrile (run 8), confirming that the carboranyl-group transfer from **Li/Cu-1** to **A1** still occurred to produce **B1** (15%) and **C1** (56%). Benzonitrile derivatives have been rarely employed as additives in Cu-mediated C–C bond formation reactions.^36^ The addition of isophthalonitrile (run 10) or terephthalonitrile (run 11) resulted in further improved CBT values (64% and 62%, respectively); however, phthalonitrile (CBT = 12%; run 9) and 1,3,5-tricyanobenzene (CBT = 28%; run 12) were ineffective. To confirm the key role of two cyano groups as coordinating units, we examined electron-deficient and electron-rich benzonitriles (runs 13 and 14), finding that the CBT values considerably decreased compared to those obtained with benzonitrile, isophthalonitrile, and terephthalonitrile (runs 8, 10, and 11). These results demonstrate that the additional cyano groups in isophthalonitrile and terephthalonitrile act not only as electron-withdrawing substituents, but also as secondary coordination sites (*vide infra*). Further modification of isophthalonitrile by substituting H atoms with F atoms also failed to improve the results (CBT = 40%, yield of **B1** = 13%, yield of **C1** = 54%; run 15). The formation of **B1** and **C1** was not observed when using PCy_3_ or benzyl cyanide, and only a trace amount of **C1** (7%) was obtained in the presence of Cl_3_CCN.

Given these results, we subsequently employed isophthalonitrile to perform the solvent screening (Figure 2c). Interestingly, ethereal solvents (i.e., THF, DME, or 1,4-dioxane) that fully dissolve **Li/Cu-1** furnished rather low CBT values (<45%). A higher CBT value of 66%, similar to that obtained in toluene (64%), was obtained when the reaction was conducted in *n*-hexane, even though the system appeared heterogeneous. Cyclohexane also afforded **B1** (25%) and **C1** (75%), resulting in a CBT of 63%. The corresponding carboranyl transfer did not proceed satisfactorily in PhCF_3_, DMF, CH_3_CN, or CHCl_3_. Considering the limited solubility of most (hetero)­aryl bromides in hexanes, we used toluene when exploring the substrate scope. It should also be noted that a significant decrease in CBT values was observed in the copresence of CuCl and/or LiCl additives, both of which coexist in the equilibrium under the reported *in situ* experimental conditions, demonstrating the importance of using the isolated **Li/Cu-1** (Figure S3, runs 10–13). After optimization of other reaction parameters (Figure S3), the dump-and-stir protocol for synthesizing **B1** as the main product was set as follows: **A1** (0.25 mmol, [**A1**] = 0.5 M) was treated with two equivalents of **Li/Cu-1** (i.e., 1.00 mmol of *o*-carboranyl groups) and isophthalonitrile in toluene, and the resultant heterogeneous mixture was stirred for 16 h at 100 °C (Figure 2d). Under these conditions, **B1** was isolated in 98% yield (i.e., 0.49 mmol of *o*-carboranyl units were incorporated) by column chromatography, while 0.45 mmol of *o*-carborane was recovered, demonstrating that the present procedure allows minimizing the loss of valuable carboranyl groups. Isophthalonitrile was also recovered in 94% yield (0.47 mmol). Therefore, our method is characterized by operational simplicity and high efficiency in introducing multiple *o*-carboranyl groups into aryl cores and in recovering the valuable *o*-carborane reagent.

Next, we applied the optimized conditions for the synthesis of a variety of multiple-carboranyl-substituted arenes (**B2**–**B12**) from the corresponding aryl bromides (**A2**–**A12**) (Figure 3) using equimolar amounts of **Li/Cu-1** and isophthalonitrile with respect to the number of Br atoms in each substrate. For example, the reaction between 1,3,5-Br_3_C_6_H_3_ (**A2**, 0.25 mmol, i.e., 0.75 mmol of Br units) and **Li/Cu-1** (0.75 mmol, i.e., 1.50 mmol of *o*-carboranyl units) proceeded in the presence of isophthalonitrile (0.75 mmol) to afford tris­(*o*-carboran-1-yl)­benzene (**B2**), which was isolated in 90% yield and unambiguously characterized by an SC-XRD analysis. Importantly, **B2** was also synthesized from 1,3,5-Cl_3_C_6_H_3_ (**A2′**) under the optimized reaction conditions in 71% yield, demonstrating the wide applicability of the present method for incorporating multiple *o*-carboranyl groups in aryl cores. In fact, *o*-carboranyl groups were multiply introduced into naphthalene and pyrene cores in **A3** and **A4**, respectively, to afford **B3** (86%) and **B4** (63%). The solid-state structures of these compounds revealed distorted arene frameworks resulting from the repulsion between the bulky *o*-carboranyl groups and the adjacent C–H bonds. These results were also reproduced in the DFT-optimized gas-phase structures of **B3** and **B4** (Figure S42). In particular, the repulsion between the C–H bonds in the pyrene K-region (i.e., C7–H, C8–H, C13–H, and C14–H) and the adjacent *o*-carboranyl C/B–H bonds induced helicity in **B4** with C1–C5–C6–C7, C2–C11–C9-C8, C3–C10–C12-C13, and C4–C16–C15-C14 torsion angles of –21.4(5)°, 19.6(5)°, 18.9(5)°, and −17.7(5)°, respectively. 1,3-Dibromoazulene (**A5**) was successfully converted into 1,3-bis­(*o*-carboran-1-yl)­azulene (**B5**), which was isolated as a purple solid in 95% yield and unambiguously characterized by an SC-XRD analysis. 1,4-Dibromobenzenes **A6** and **A8**, which bear fluorine or methoxy groups at the 2,5-positions, afforded the corresponding bis-carboranyl benzenes (**B6** and **B8**) in excellent yields. Additionally, a gram-scale experiment using **A7** (4.84 g, 17.8 mmol) also proceeded efficiently to afford 2,4-difluoro derivative **B7** in 49% isolated yield (3.49 g). We also synthesized *N*-(*o*-carboran-1-yl)­aryl carbazole **B9** in 86% yield, demonstrating the practicality of our dump-and-stir method given that *N*-aryl carbazoles have attracted much attention for their applications in organic light-emitting diodes (OLEDs), particularly as hole-transporting materials, bipolar hosts, and thermally activated delayed fluorescence (TADF) emitters.^37^ Two *o*-carboranyl groups were introduced in dibromo-substituted pyrazine and pyridazine cores (**A10**-**A12**) to afford **B10** (52%), **B11** (97%), and **B12** (93%), respectively.

**Figure 3:**
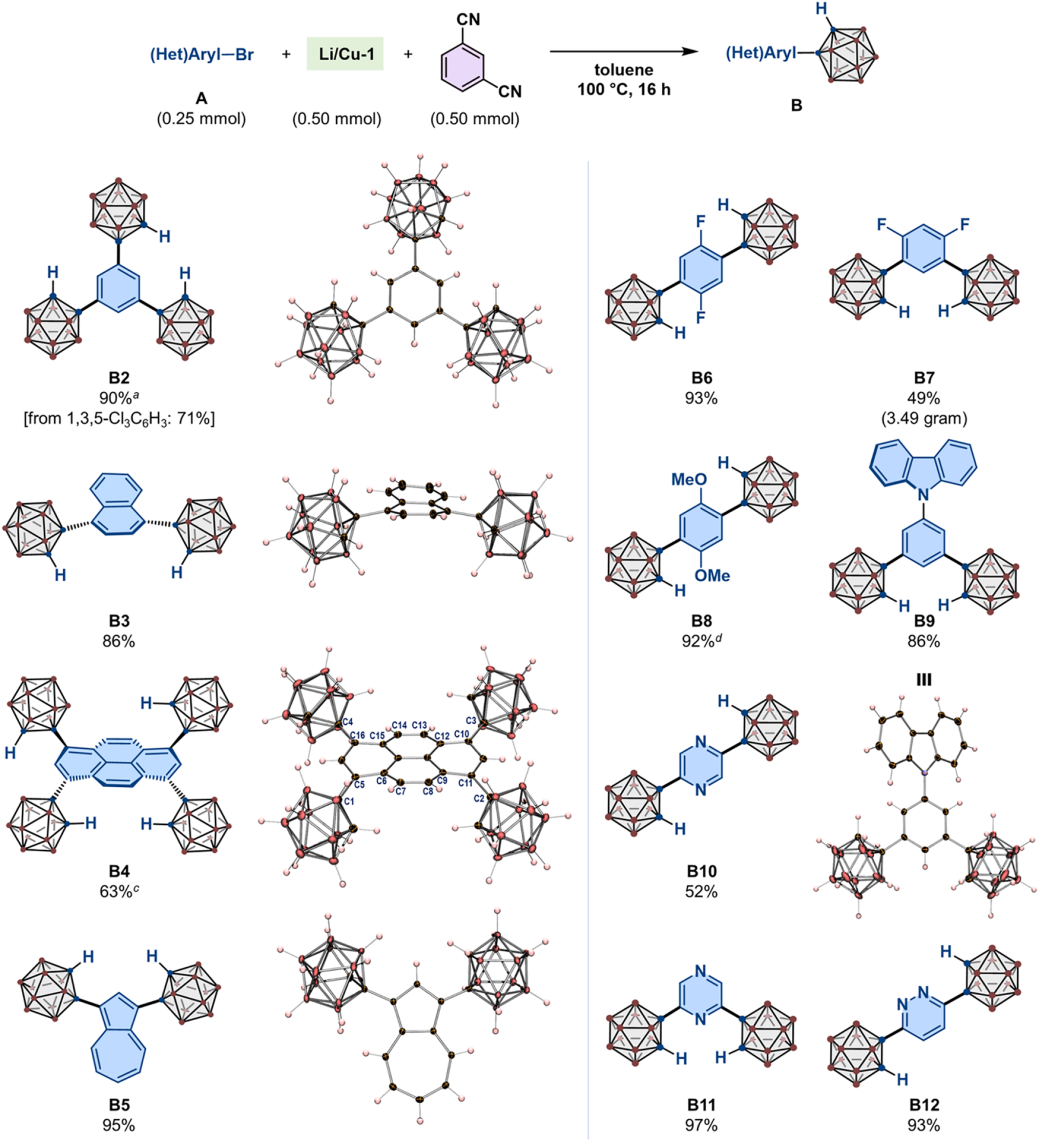
Synthesis of **B2**–**B12**. Unless otherwise noted, the reactions were performed using the optimized conditions shown in Figure 2d; isolated yields are given. Molecular structures of **B2**–**B5**, and **B9**, determined by SC-XRD analyses, are shown with thermal ellipsoids at 30% probability (solvate molecules are omitted for clarity). ^*a*^0.75 mmol of **Li/Cu-1** and isophthalonitrile were used. ^*c*^1.00 mmol of **Li/Cu-1** and isophthalonitrile were used. ^*d*^Reaction time: 32 h. For carborane or carboranyl groups, brown spheres represent B–H units while blue spheres represent C atoms, as shown in Figure 1a.

Subsequently, we synthesized monocarboranyl arenes **C13**–**C30** by simply mixing equimolar amounts of aryl bromides, **Li/Cu-1**, and isophthalonitrile in toluene, followed by stirring at 100 °C (Figure 4a). These results highlight several characteristics of **Li/Cu-1** in terms of reactivity. First, the present system tolerates Lewis-acidic (e.g., the pinacolato boron group in **A14**) and -basic (e.g., N, S, or O-heterocycles in **A23**–**A30**) functional groups, affording a variety of (*o*-carboran-1-yl)­arenes in high to excellent isolated yield.^38^ In particular, **C20** and **C21** feature a fusion of strongly electron-withdrawing and 3D-aromatic carboranes with stimuli-responsive spirobenzopyran and 1,8-naphthalimide cores, which have been widely applied in materials and biological sciences.^39,40^ However, the presence of acidic protons in **A16** (−OH) and **A17** (−NH) affected the reaction progress owing to protonation of the Cu–carboranyl bonds. In these cases, we added 1.5 equiv of **Li/Cu-1** (i.e., 3.0 equiv of *o*-carboranyl units) and isophthalonitrile with respect to each substrate, and eventually isolated **C16** and **C17** in 81% and 88% yield, respectively. Again, in the latter case, *o*-carborane and isophthalonitrile were easily recovered in excellent yields from the crude mixture. Moreover, **C22**, a bioisostere analogue of flurbiprofen, which exhibits versatile pharmaceutical properties (e.g., anti-inflammatory, antifungal, and analgesic),^16^ was prepared in 61% yield from **A22** without protection of the carboxylic group. Second, **Li/Cu-1** can distinguish aryl bromides from the corresponding chlorides and fluorides when different halogen substituents coexist in a single molecule, thus enabling the selective synthesis of **C18** (97%), **C19** (93%), and **C23** (80%). Our approach is also valuable as a method complementary to the nucleophilic aromatic substitution using 2-halopyridines and *in situ*-generated carboranyl-lithium species (e.g., **Li-1** in Figure 2a). For example, Lu and co-workers have reported the selective substitution of the 2-Cl atom in 5-bromo-2-chloropyridine (**A23**) while the 5-Br atom remained intact,^41^ whereas the 5-Br atom selectively reacted in our system to afford **C23** with a 2-Cl atom. To further confirm the applicability of the present *C*-carboranylation method to aryl chlorides, 4-chlorotoluene (**A13**′), 4-chlorophenol (**A16′**), and 4-chloro-2-cyanopyridine (**A24′**) were subjected to the optimal reaction conditions, affording **C13**, **C16** and **C24** in 71%, 54% and 26% yield, respectively. The corresponding carboranylation of 4-iodotoluene (**A13**″) proceeded effectively even at 60 °C to afford **C13** in 95% yield after 48 h.

**Figure 4:**
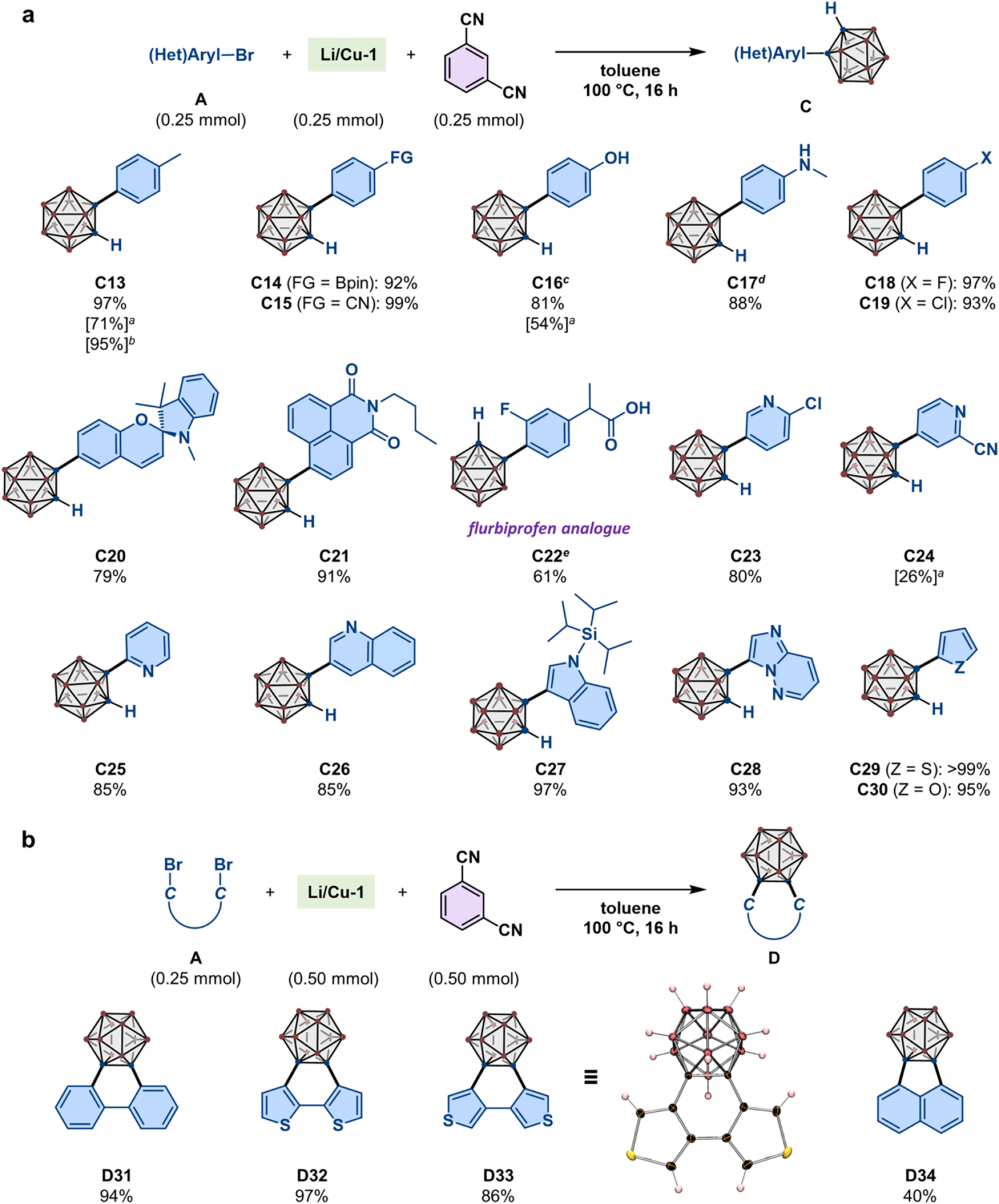
Substrate scope. (a) Synthesis of **C13**–**C30**; isolated yields are given. ^*a*^Reactions using the corresponding aryl chlorides. ^*b*^Reaction was carried out with 4-iodotoluene (**A13″**) at 60 °C for 48 h. ^*c*^1.5 equiv of **Li/Cu-1** and isophthalonitrile were used. ^*d*^1.5 equiv of **Li/Cu-1** and isophthalonitrile vs **A17** (0.50 mmol) were used. *o*-Carborane (0.95 mmol) and isophthalonitrile (0.71 mmol) were recovered. ^*e*^2.0 equiv of **Li/Cu-1** and isophthalonitrile were used. (b) Synthesis of **D31**–**D34**. The molecular structure of **D33**, determined by an SC-XRD analysis, is shown with thermal ellipsoids at 30% probability. For carborane or carboranyl groups, brown spheres represent B–H units and blue spheres represent C atoms, as shown in Figure 1a.

Importantly, during the formation of these compounds, we did not observe any signals with the chemical shift (δ_H_ 3.81) that suggests the formation of bis-*o*-carborane, which tends to be formed easily through the generation of **CB-Cu_2_** species in aromatic hydrocarbon solvents (Figure 1b),^42^ manifesting the benefit to use an isolated **Li/Cu-1** reagent.

We then attempted the construction of 2,2′-bis­(*o*-carboran-1-yl)­biphenyl using 2,2′-dibromobiphenyl (**A31**) under the optimized conditions; however, carborane-fused biphenyl **D31** was obtained in nearly quantitative yield (Figure 4b). Similarly, bithiophene-fused **D32** (97%) and **D33** (86%) as well as naphthalene-fused **D34** (40%) were obtained. These results demonstrate another practical application of **Li/Cu-1** for the construction of carborane-fused π-conjugated (hetero)­arenes, as some of these intriguing compounds were previously obtained in low to moderate yield from reactions between decaborane (or its thiol adducts) and alkynes that were also prepared through multistep Pd-catalyzed coupling processes.^43–45^

Through the synthesis of novel carborane-substituted products (e.g., **B3**–**B12**, **C14**, **C17**, **C20**–**C24**, **C26**–**C28**, and **D33**) and their SC-XRD analyses, we found that the carboranyl C–C bond lengths are ca. 1.65 Å, with rather larger values (∼1.68 Å) observed when the carboranes are introduced in sterically crowded environments e.g., **B3**, **B4**, and **C27**. It should also be noted here that the unreacted C–H bonds in the carborane units remain in **B1**–**B12** and **C13**–**C30**, which could be used for further molecular derivatization. The ^1^H NMR chemical shifts of these C–H bonds vary depending on the surrounding environment and tend to shift downfield when adjacent heteroatoms that can form hydrogen-bonding interactions are present.

To investigate the reaction mechanisms for the C–C bond formation between **Li/Cu-1** and PhBr, we performed DFT calculations at the PBE0-D3/Def2-TZVPD­(Cu) and Def2-TZVP­(others) level in the gas phase. A plausible reaction pathway is depicted in Figure 5a along with the structures of selected molecules (*o*-carboranyl groups are shown as R). The relative Gibbs free energies of each molecule are given with respect to [**Li/Cu-1** + 2 PhBr] (0.0 kcal mol^–1^). First, the *ortho*-carbon (labeled as C4) in PhBr coordinates to the Cu center in **Li/Cu-1** to give energetically slightly favorable **Int1** (−0.9 kcal mol^–1^). Then, dissociation of one THF molecule from the Li center generates **Int2** (+2.2 kcal mol^–1^), which undergoes an oxidative addition to the Ph–Br bond via **TS**_**OA1**_ **-v1** (+27.7 mol mol^–1^) as the rate-determining step (Δ*G* ^‡^ = +28.6 kcal mol^–1^). The oxidative addition can also occur directly from **Int1** without the dissociation of one THF molecule via **TS**_**OA1**_ **-v2** (+29.1 kcal mol^–1^; Figure S39), although this transition state is rather unfavorable (Δ*G* ^‡^ = +30.0 kcal mol^–1^). In addition, we confirmed that [Li­(thf)_3_]^+^ does not facilitate the oxidative addition, proceeding via **TS_OA1_-v3** (+33.6 kcal mol^–1^; Figure S39), by acting as a Lewis acid to promote cleavage of the C–Br bond. The formed distorted square-planar *cis*-R_2_Cu­(III) complex (**Int3**, + 24.6 kcal mol^–1^) can be expected to readily isomerize to its *trans*-conformer (**Int4**; + 12.3 kcal mol^–1^), which contains a Br···Li^+^ interaction. This interaction effectively promotes the subsequent reductive elimination via **TS**_**RE1**_ **-v1** (+19.6 mol mol^–1^) to produce PhR and [Li­(thf)_3_]­[CuR­(Br)]. In contrast, we also found a substantially less stable transition state **TS**_**RE1**_ **-v2** (+29.9 mol mol^–1^; Figure S39), which includes a [Li­(thf)_4_]^+^ unit and thus cannot form the corresponding interaction between the Li^+^ and Br centers during the reductive elimination. As **Li/Cu-1** can transfer two carboranyl groups (i.e., CBT > 50%; Figure 2b), we further explored the reaction pathways to generate another equivalent of PhR from [Li­(thf)_3_]­[CuR­(Br)] and PhBr. We identified two key transition states, i.e., **TS_OA2_** (−10.8 kcal mol^–1^) and **TS_RE2_** (−10.7 kcal mol^–1^), in the pathway leading to the final products (PhR + [Li­(thf)_3_]­[CuBr_2_]; – 71.5 kcal mol^–1^). The energy barrier to overcome **TS_RE2_** (Δ*G* ^‡^ = +28.2 kcal mol^–1^) limits the reaction progress after the generation of [Li­(thf)_3_]­[CuR­(Br)]. Combining all these results, the overall energy barrier of approximately +28 kcal mol^–1^ to overcome **TS**_**OA1**_ **-v1** and **TS_RE2_** is consistent with the sluggish progress of the reaction in the absence of a ligand (Figure 2b, run 1).

**Figure 5:**
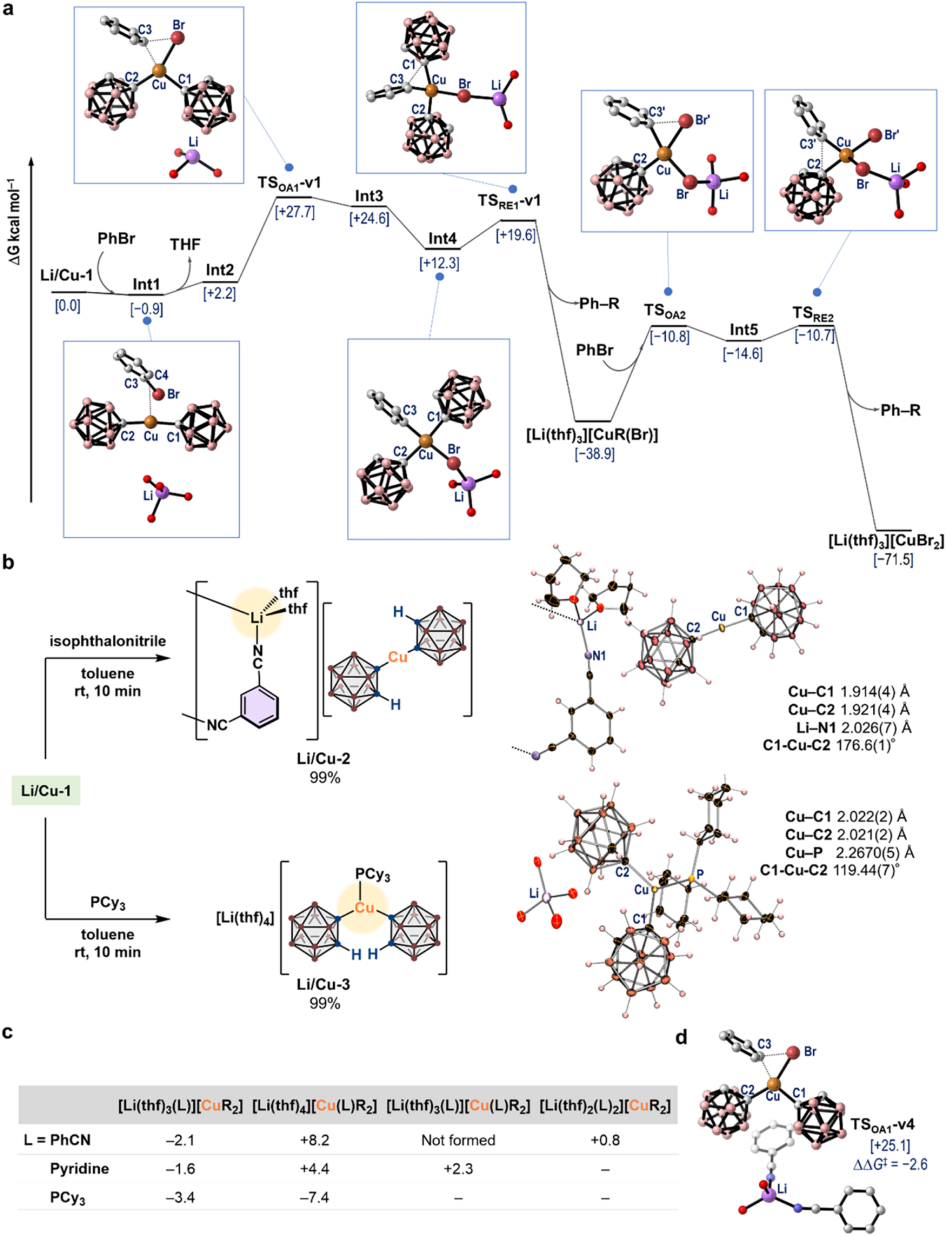
Mechanistic studies (R = *o*-carboranyl group). (a) Plausible reaction pathway and selected optimized molecular structures in the gas phase calculated at the PBE0-D3/Def2-TZVPD­(Cu) and Def2-TZVP­(others) level. Selected atoms (Li: purple; B: pink; C: gray; O: red; Cu: orange; Br: reddish brown) and relative Gibbs free energies (kcal mol^–1^) with respect to [**Li/Cu-1** + 2 PhBr] are shown. (b) Isolation of **Li/Cu-2** and **Li/Cu-3**. Their molecular structures, determined via SC-XRD analyses, are shown with thermal ellipsoids at 30% probability (THF molecules in **Li/Cu-3** are omitted for clarity). (c) Relative Gibbs free energies of {[Li­(thf)_3_(L)]­[CuR_2_] + THF + L}, {[Li­(thf)_4_]­[Cu­(L)­R_2_] + L}, {[Li­(thf)_3_(L)]­[Cu­(L)­R_2_] + THF}, and {[Li­(thf)_2_(L)_2_]­[CuR_2_] + 2 THF} with respect to [**Li/Cu-1** + 2 L]. (d) Optimized gas-phase structure of **TS**_**OA1**_ **-v4** and its relative Gibbs free energy with respect to [**Li/Cu-1** + 2 PhCN].

*N*-Donor ligands such as pyridine have been proposed to coordinate to the Cu­(I) center in Ullmann-type coupling reactions.^29^ To explore whether isophthalonitrile can similarly coordinate to the Cu center, we conducted a stoichiometric reaction between isophthalonitrile and **Li/Cu-1**, and quantitatively isolated complex **Li/Cu-2**, which comprises polymeric [Li­(thf)_2_]^+^ structures bridged by isophthalonitriles (Figure 5b, top). In stark contrast, the phosphine-coordinated Cu complex **Li/Cu-3** was isolated in 99% yield when equimolar amounts of **Li/Cu-1** and PCy_3_ were mixed (Figure 5b, bottom). The molecular structures of these complexes were determined by SC-XRD analyses. The structure of the bis­(carboran-1-yl)­cuprate unit in **Li/Cu-2** is almost identical to the corresponding structure in **Li/Cu-1**, suggesting that the oxidative addition of aryl halides can be expected to proceed similarly. However, the coordination of PCy_3_ to the Cu center causes significant changes in the geometry of the cuprate unit, which would prevent the key oxidative-addition step in **Li/Cu-3**. Furthermore, we theoretically compared the stability of [Li­(thf)_3_(L)]­[CuR_2_] and [Li­(thf)_4_]­[Cu­(L)­R_2_] complexes (R = *o*-carboranyl) using pyridine, PCy_3_, and PhCN as a model for isophthalonitrile to facilitate the discussion of monomeric lithium cuprate complexes (the relative Gibbs free energies are given with respect to [**Li/Cu-1** + 2 L]; Figure 5c). As expected, PhCN afforded [Li­(thf)_3_(CNPh)]­[CuR_2_] (−2.1 kcal mol^–1^), which is more stable than [Li­(thf)_4_]­[Cu­(CNPh)­R_2_] (+8.2 kcal mol^–1^). Although [Li­(thf)_3_(CNPh)_2_]­[CuR_2_] (+0.8 kcal mol^–1^) is also a plausible species, optimization of [Li­(thf)_3_(CNPh)]­[Cu­(CNPh)­R_2_] failed due to dissociation of PhCN from the Cu center. The critical benefit of using isophthalonitrile is thus rationalized by its coordination to the Li center while leaving the Cu center available for the oxidative addition. In fact, the oxidative addition to PhBr proceeded via **TS**_**OA1**_ **-v4** (+25.1 kcal mol^–1^; Figures 5d and S40) from [Li­(thf)_3_(CNPh)_2_]­[CuR_2_], whereby this path is far more favorable (ΔΔ*G* ^‡^ = −2.6 kcal mol^–1^) than the pathway proceeding via **TS**_**OA1**_ **-v1** (Figure 5a). In this context, based on the atoms-in-molecules analysis, we confirmed that noncovalent interactions participate between PhCN and carboranyl units, efficiently stabilizing **TS**_**OA1**_ **-v4** (Figure S41). Conversely, in the case of PCy_3_, its coordination to the Cu center was clearly preferred over the formation of [Li­(thf)_3_(PCy_3_)]­[CuR_2_]. These experimental and theoretical results are consistent with the fact that no reaction occurred in the presence of PCy_3_ (Figure 2b). In the case of pyridine, the energy gaps between [Li­(thf)_3_(pyridine)]­[CuR_2_] (−1.6 kcal mol^–1^) and pyridine-cuprate species such as [Li­(thf)_4_]­[Cu­(pyridine)­R_2_] (+4.4 kcal mol^–1^) and [Li­(thf)_3_(pyridine)]­[Cu­(pyridine)­R_2_] (+2.3 kcal mol^–1^) are smaller than the case using PhCN. Therefore, the lower CBT value of 37% (Figure 2b, run 2) can be attributed to the partial formation of such pyridine-cuprate species, which would exist in an equilibrium with pyridine-lithium species under the applied reaction conditions.^46^

## Conclusions

We have developed a novel cuprate complex that bears two *o*-carboranyl groups (**Li/Cu-1**), which enables the incorporation of multiple *o*-carborane groups into arenes via an efficient and practical dump-and-stir synthesis with readily available (hetero)­aryl bromides and chlorides. We have also demonstrated that isophthalonitrile coordinates to the Li center rather than to the cuprate unit, allowing the Cu center to undergo the rate-determining oxidative addition to carbon–halide bonds. This counterintuitive ligand design represents a paradigm shift from traditional approaches that focus on the coordination of pyridines and phosphines to Cu. Overall, our findings demonstrate that well-characterized isolated reagents can overcome the limitations of traditional *in situ* approaches, not only rendering the synthetic process more straightforward and practical but also facilitating the optimization of reaction conditions and providing access to a wider range of molecules.

## Supplementary Material





## Notes

Full details pertaining to the experimental methods, identification of the compounds, and DFT calculations (PDF)
SI-DFT-details (XLSX)
