# Supplementary material for: An Isolated Lithium ortho-Carboranyl Cuprate Complex for the Synthesis of Multiple-Carborane-Substituted Arenes from (Hetero)Aryl Bromides and Chlorides
Source: J Am Chem Soc. 2025 Oct 1;147(41):37677–87. doi: 10.1021/jacs.5c13004 (PMC12532192; doi:10.1021/jacs.5c13004)

Supporting Information

An Isolated Lithium *ortho*-Carboranyl Cuprate Complex for the Synthesis of Multiple-Carborane-Substituted Arenes from (Hetero)Aryl Bromides and Chlorides

Yusei Hisata,[a] Daina Morishita,[a] and Yoichi Hoshimoto*[a,b]

[a] Department of Applied Chemistry, Graduate School of Engineering, The University of Osaka, Suita, Osaka 565-0871, Japan
[b] Center for Future Innovation (CFi), Graduate School of Engineering, The University of Osaka, Suita, Osaka 565-0871, Japan

*Corresponding author: hoshimoto@chem.eng.osaka-u.ac.jp (to Y.Ho.)

Table of Contents

[1] General considerations p. S3

[2] Materials pp. S3−S4

[3] Optimization of reaction conditions pp. S5−S6

3-1. Screening of the additives

3-2. Screening of other parameters

[4] Preparation of carboranyl metal compounds pp. S7–S9

4-1. Preparation of **Li-1**

4-2. Preparation of **Li/Cu-1**

4-3. Preparation of **Li/Cu-2**

4-4. Preparation of **Li/Cu-3**

[5] Reaction between aryl halides and Li/Cu-1 pp. S10–S27

[6] Reaction of Li/Cu-1 with pyridine p. S28

[7] Results of *in situ* experiments pp. S29−S30

7-1. *In situ* generation of carboranyl copper species

7-2. Ni-catalyzed carboranylation of **A1**

[8] Recovery of *o*-carborane and isophthalonitrile p. S31

8-1. Reaction between **A1** and **Li/Cu-1**

8-2. Reaction between **A17** and **Li/Cu-1**

[9] Synthesis of 1,1′-bis(*o*-carborane) p. S32

[10] Theoretical studies pp. S33–S42

10-1. Computational details

10-2. Method Optimization

10-3. Mechanistic Studies

10-4. Atoms in molecule (AIM) analysis

10-5. Comparison of gas-phase-optimized and SC-XRD structures of **B3** and **B4**

[11] Reference and Notes pp. S43–S44

[12] NMR spectra pp. S45−S123

[1] General considerations

Unless otherwise noted, all manipulations were conducted under a N_2 atmosphere using standard Schlenk line or grove box (GB) techniques. ^{1}H, ^{11}B, ^{13}C, ^{19}F, and ^{31}P NMR spectra were recorded on a Bruker AVANCE III 400 or at 25 °C. The chemical shifts in the ^{1}H NMR spectra were recorded relative to Me_4Si or residual protonated solvent ($CHCl_3$ (δ 7.26), $CDCl_2H$ (δ 5.32), C_6D_5H (δ 7.16)). The chemical shifts in the ^{11}B NMR spectra were recorded relative to $BF_3{\cdot}Et_2O$. The chemical shifts in the ^{13}C spectra were recorded relative to Me_4Si or deuterated solvent ($CDCl_3$ (δ 77.16), CD_2Cl_2 (δ 53.84), C_6D_6 (δ 128.06), DMSO-d_6 (δ 39.52)). The chemical shifts in the ^{19}F NMR spectra were recorded relative to α,α,α-trifluorotoluene (δ −65.64). The chemical shifts in the ^{31}P NMR spectra were recorded relative to 85% H_3PO_4 as an external standard. Assignment of the resonances in ^{1}H and ^{13}C NMR spectra was based on ^{1}H−^{1}H COSY, HMQC, and HMBC experiments. High resolution mass spectrometry (HRMS) and elementary analyses were performed at the Instrumental Analysis Center, Faculty of Engineering, Osaka University. Exact mass spectra were obtained using a double-focusing mass spectrometer (JMS-700; JEOL), DART (JMST-100LP; JEOL), and MALDI-TOF (JMS-S3000; JEOL) mass spectrometry.

X-ray crystal data were collected using the Rigaku XtaLAB Synergy equipped with the HyPix-6000HE detector. Using Olex[1], the structure was solved with the SHELXT2[2] structure solution program using Intrinsic Phasing and refined with the SHELXL[3] refinement package using Least Squares minimization. It should be noted that the boron and carbon atoms in the *o*-carborane moieties might be incorrectly assigned due to the difficulty of distinguishing them by XRD analysis, especially when crystallographic disorder of the pseudo-spherical cluster is observed[4].

[2] Materials

All commercially available reagents including super-dehydrated solvents (*n*-hexane, toluene, tetrahydrofuran), were purchased from Sigma Aldrich, TCI, and Wako Pure Chemical Industries, and used as received. Benzene-d_6 was distilled from sodium benzophenone ketyl prior to use. CD_2Cl_2 was once degassed by several freeze-pump-thaw cycles and stored inside GB over molecular sieves (4 Å). *o*-Carborane was purchased from Angene and used after sublimation. Substrates used in this work are summarized in Figure S1.

Metrical data for the solid-state structures are available from Cambridge Crystallographic Data Centre: CCDC 2469686 (**Li-1**), 2486445 (**Li/Cu-1**), 2469688 (**Li/Cu-2**), 2469689 (**Li/Cu-3**), 2469669 (**B2**), 2469670 (**B3**), 2469671 (**B4**), 2469672 (**B5**), 2469673 (**B6**), 2469674 (**B7**), 2469675 (**B8**), 2469676 (**B9**), 2469666 (**B10**), 2469667 (**B11**), 2469680 (**B12**), 2469677 (**C14**), 2469678 (**C17**), 2469679 (**C20**), 2469680 (**C21**), 2470754 (**C22**), 2469681 (**C23**), 2469682 (**C24**), 2483553 (**C26**), 2469683 (**C27**), 2469684 (**C28**), 2469685 (**D33**).

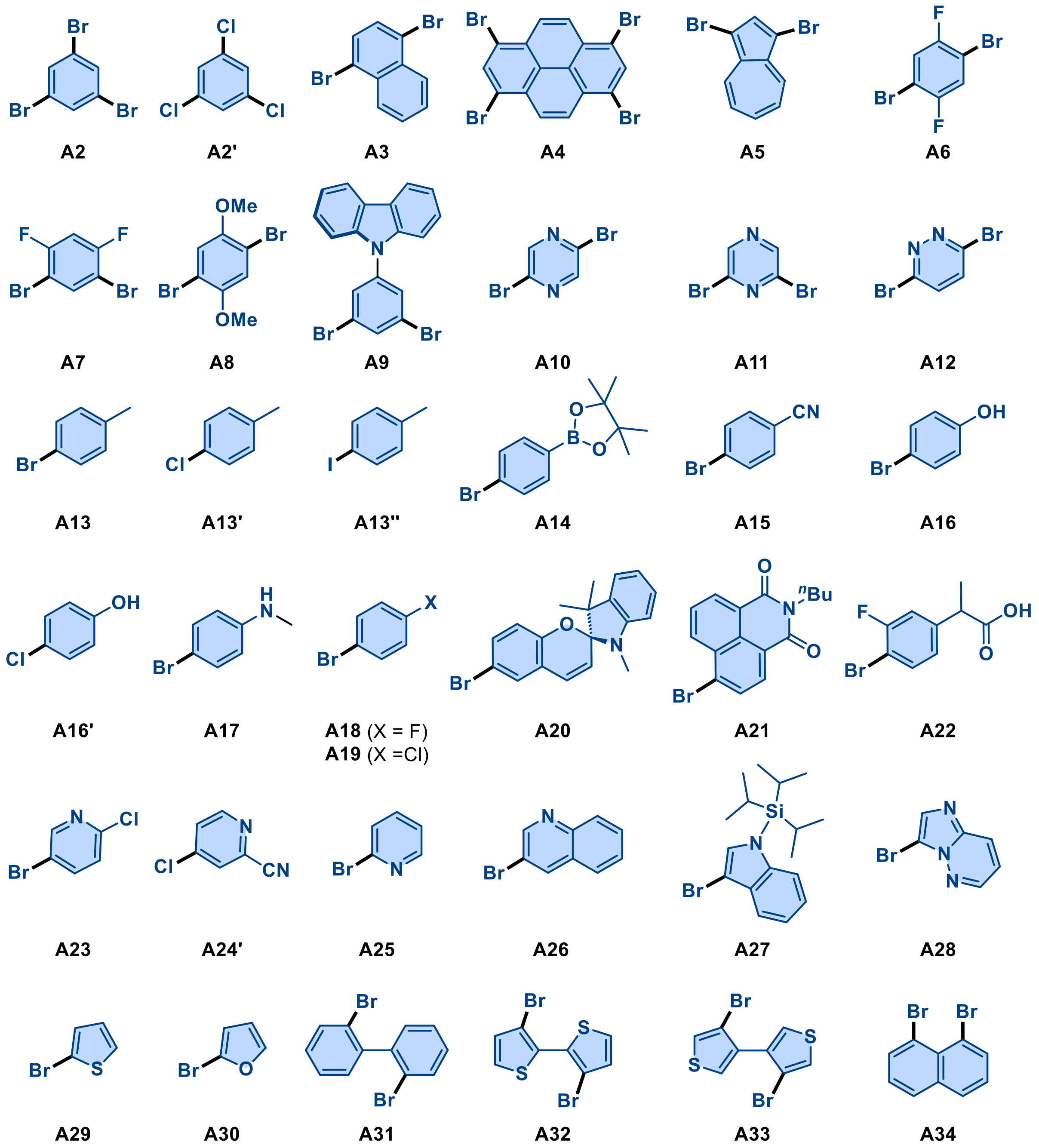

Figure S1. Substrates used in this work

[3] Optimization of reaction conditions

3-1. Screening of the additives

In a screw-cap reaction vessel equipped with a magnetic stirrer bar, **Li/Cu-1** (0.20 mmol), an additive (0.30 mmol), and 1,4-dibromobenzene (**A1**, 0.20 mmol) were added in this order. Toluene (0.4 mL) was then added, and the vessel was sealed. The mixture was stirred at 80 °C for 16 h. After cooling to rt, the resultant mixture was diluted with wet Et_2O. The resulting mixture was then filtered through filter paper, and the volatiles were removed under reduced pressure. The yields of **B1** and **C1** were determined by NMR analysis using 1,3,5-trimethoxybenzene as an internal standard (Figure 2b, and additional results are given in Figure S2). CBT (carboranyl-group transfer ratio) values were calculated using the following equation:

$$\text{CBT (\%)} = \frac{\text{total moles of carborane units in } \mathbf{B1} \text{ and } \mathbf{C1}}{\text{total moles of carborane units in } \mathbf{Li/Cu\text{-}1}} \times 100$$

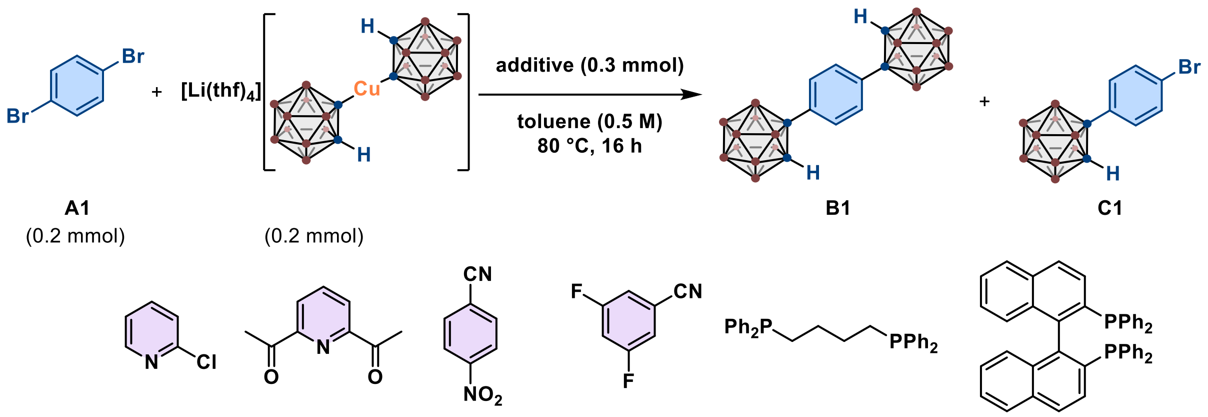

**B1** [%]	7	0	12	7	0	0
**C1** [%]	32	0	55	42	0	0
CBT [%]	23	0	40	28	0	0

Figure S2. Screening of additives (additional results to those shown in Figure 2b).

3-2. Screening of other parameters

In a screw-cap reaction vessel equipped with a magnetic stirrer bar, **A1** (47.2 mg, 0.200 mmol), **Li/Cu-1** (129.1 mg, 0.200 mmol, 1.0 equiv), isophthalonitrile (X equiv), and an additional additive (1.0 or 2.0 equiv, or none)**,** were added. Toluene (0.25 M, 0.5 M, or 1.0 M with respect to **Li/Cu-1**) was then added, and the vessel was sealed. The mixture was stirred at 70, 80, or 100 °C for 16 h. After cooling to rt, the reaction mixture was diluted with wet Et_2O. The resulting mixture was filtered through filter paper, and the volatiles were removed under reduced pressure. The yields of **B1** and **C1** were determined by NMR analysis using 1,3,5-trimethoxybenzene as an internal standard. The results of the solvent screening are shown in Figure 2c, while others are given in Figure S3.

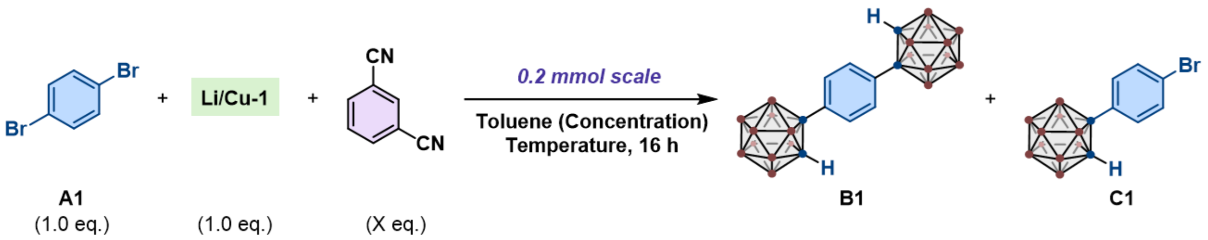

Run	X [eq.]	Concentration	Temperature [°C]	Additive	**B1** [%]	**C1** [%]	CBT [%]
1	0.5	0.5	80	none	7	44	29
2	1.0	0.5	80	none	31	69	66
3	2.0	0.5	80	none	28	72	64
4	2.5	0.5	80	none	23	74	60
5	3.0	0.5	80	none	30	70	65
6	3.5	0.5	80	none	29	71	65
7	1.0	0.25	80	none	4	29	19
8	1.0	1.0	80	none	17	66	50
9	1.0	0.5	70	none	4	18	13
10	1.0	0.5	80	CuCl (1.0 eq.)	14	62	45
11	1.0	0.5	80	LiCl (1.0 eq.)	17	69	52
12	1.0	0.5	80	CuCl (1.0 eq.) + LiCl (1.0 eq.)	16	60	46
13	1.0	0.5	80	CuCl (1.0 eq.) + LiCl (2.0 eq.)	17	64	49
14	1.0	0.5	100	none	33	67	67

Figure S3. Optimization of reaction conditions with isophthalonitrile ligand.

[4] Preparation of carboranyl metal compounds

4-1. Preparation of **Li-1** (Figure 2a)

A solution of *o*-carborane (6.30 g, 43.7 mmol, 1.0 M in THF) was slowly treated with ^{n}BuLi (28.4 mL of a 1.54 M solution in *n*-hexane, 43.7 mmol) at 0 °C, and the mixture was allowed to warm to rt over 1 h with stirring, followed by removal of all volatiles in vacuo. The residue was then washed with *n*-hexane (10 mL × 4) and dried in vacuo, affording **Li-1** as a white solid (15.45 g, 42.2 mmol, 96%). A single crystal of **Li-1** was obtained from a saturated solution of **Li-1** in toluene at rt. **^{1}H NMR** (400 MHz, C_6D_6, rt, δ/ppm): 3.40 (t, J = 6.4 Hz, 12H, O-CH_2), 3.11 (s, 1H, cage CH), 1.52–1,31 (m, 12H, CH_2-CH_2-CH_2), 3.80–1.65 (brm, BH). **^{11}B NMR** (128 MHz, C_6D_6, rt, δ/ppm): 0.90–−3.30 (m, 2B), −4.53–−6.89 (m, 2B), −7.68–−12.96 (m, 6B). **^{13}C{^{1}H} NMR** (100 MHz, C_6D_6, rt, δ/ppm): 90.7, 68.4, 62.2, 25.6. **Li-1**: M = 366.46, colorless, monoclinic, $P2_1/n$ (#14), a = 13.5724(5) Å, b = 11.5145(5) Å, c = 14.3564(7) Å, α = 90°, β = 96.460(4)°, γ = 90°, V = 2229.36(17) Å^3, Z = 4, $Dcalcd$ = 1.092 g/cm^3 , T = 123.15 K, R_1 (wR_2) = 0.0866 (0.2371).

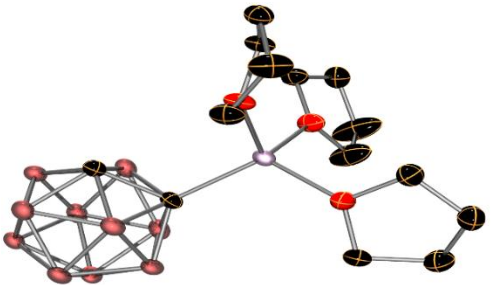

Figure S4. Molecular structure of **Li-1** with ellipsoids set at 30% probability. H atoms are omitted for clarity.

4-2. Preparation of **Li/Cu-1**

Method A (Figure 2a)**:** CuCl (1.53 g, 15.5 mmol, 0.55 equiv) was added to a solution of **Li-1** (10.32 g, 28.2 mmol, 1.0 M in THF) at rt, and the mixture was stirred for 12 h. After removal of all volatiles in vacuo, the residue was washed with *n*-hexane (10 mL × 4). The residue was then extracted with toluene through a Celite pad, followed by removal of all volatiles in vacuo, affording **Li/Cu-1** as a brownish solid (8.53 g, 13.2 mmol, 94%). A single crystal of **Li/Cu-1** was obtained from a saturated solution of **Li/Cu-1** in dichloromethane at rt. **^{1}H NMR** (400 MHz, CD_2Cl_2, rt, δ/ppm): 3.80 (t, J = 6.4 Hz, 16H, O-CH_2), 3.28 (s, 2H, cage CH), 2.10–1,95 (m, 16H, CH_2-CH_2-CH_2), 2.77–1.18 (brm, BH). **^{11}B NMR** (128 MHz, CD_2Cl_2, rt, δ/ppm): −0.25–−4.50 (m, 4B), −6.91–−13.79 (m, 16B). **^{13}C{^{1}H} NMR** (100 MHz, CD_2Cl_2, rt, δ/ppm): 75.3, 69.1 (t, J = 13.1 Hz), 62.2, 25.9. **X-ray** data for **Li/Cu-1** (Figure 2a): M = 645.31, colorless, monoclinic, $P2_1/c$ (#14), a = 14.9640(2) Å, b = 14.6836(2) Å, c = 18.0146(3) Å, α = 90°, β = 113.296(2)°, γ = 90°, V = 3635.56(10) Å^3, Z = 4, $Dcalcd$ = 1.179 g/cm^3 , T = 123.15 K, R_1 (wR_2) = 0.0488 (0.1369).

Method B (Figure 2a)**:** A solution of *o*-carborane (10.0 g, 69.3 mmol, 1.0 M in THF) was slowly treated with ^{n}BuLi (45.0 mL of a 1.54 M solution in *n*-hexane, 69.3 mmol, 1.0 equiv) at −35 °C, and the mixture was allowed to warm to rt over 1 h with stirring. CuCl (3.78 g, 38.2 mmol, 0.55 equiv) was then added

to the resultant solution at rt, and the mixture was stirred for 12 h. After removal of all volatiles in vacuo, the residue was washed with *n*-hexane (10 mL × 4). The residue was then extracted with toluene through a Celite pad, followed by removal of all volatiles in vacuo, affording **Li/Cu-1** as a brownish solid (19.9 g, 30.8 mmol, 89%).

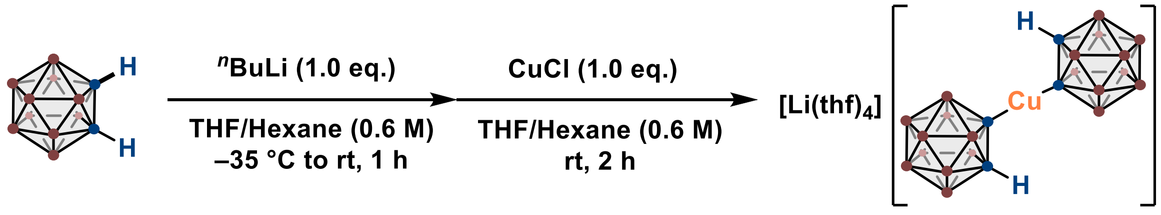

Figure S5. One-pot synthesis of **Li/Cu-1** from *o*-carborane with CuCl (1.0 equiv)

Method C (Figure S5): A solution of *o*-carborane (4.66 g, 32.3 mmol, 1.0 M in THF) was slowly treated with ^{n}BuLi (20.9 mL of a 1.55 M solution in *n*-hexane, 32.4 mmol, 1.0 equiv) at −35 °C, and the mixture was allowed to warm to rt over 1 h with stirring. CuCl (3.20 g, 32.3 mmol, 1.0 equiv) was then added to the resultant solution at rt, and the mixture was stirred for 2 h. After removal of all volatiles in vacuo, the residue was washed with *n*-hexane (10 mL × 4) and toluene (10 mL)**.** The residue was then extracted with dichloromethane through a Celite pad, followed by removal of all volatiles in vacuo, affording **Li/Cu-1** as a brownish solid (9.41 g, 14.6 mmol, 90%).

4-3. Preparation of **Li/Cu-2** (Figure 5b)

Isophthalonitrile (64.1 mg, 0.500 mmol, 1.0 equiv) was added to a suspension of **Li/Cu-1** (322.7 mg, 0.500 mmol, 0.5 M in toluene) at rt, and the resultant mixture was stirred for 10 min, followed by removal of all volatiles in vacuo, affording **Li/Cu-2** as a dark brown solid (314.0 mg, 0.499 mmol, 99%). A single crystal of **Li/Cu-2** was obtained from a saturated solution of **Li/Cu-2** in toluene at rt. **^{1}H NMR** (400 MHz, CD_2Cl_2, rt, δ/ppm): 8.10–7.97 (m, 3H, Ar-*H*), 7.77 (brs, 1H, Ar-*H*), 3.85–3.79 (m, 8H, O-CH_2), 3.30 (m, 2H, cage C*H*), 2.01–1.92 (m, 8H, CH_2-CH_2-CH$_2$), 3.16–1.11 (brm, B*H*). **^{11}B NMR** (128 MHz, CD_2Cl_2, rt, δ/ppm): −0.31–−4.64 (m, 4B), −6.81–−13.72 (m, 16B). **^{13}C{^{1}H} NMR** (100 MHz, CD_2Cl_2, rt, δ/ppm): 137.6, 136.5, 131.3, 113.3, 74.9, 69.0 (t, J = 13.9 Hz), 62.2, 25.9. A signal of the ipso-carbon with respect to the cyano group was not identified. **X-ray** data for (**Li/Cu-2)·C_7H_8**: M = 721.37, colorless, monoclinic, $P2_1/c$ (#14), a = 16.9524(10) Å, b = 11.4965(5) Å, c = 22.1848(15) Å, α = 90°, β = 109.811(7)°, γ = 90°, V = 4067.8(4) Å^3, Z = 4, $Dcalcd$ = 1.178 g/cm^3 , T = 123.15 K, R_1 (wR_2) = 0.0617 (0.1483).

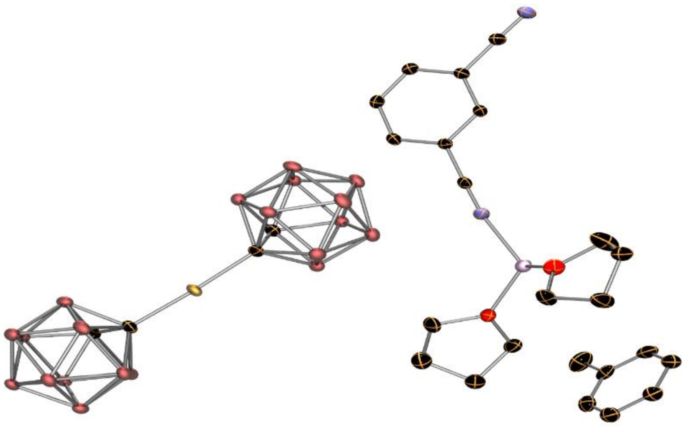

Figure S6. Molecular structure of (**Li/Cu-2**)·C_7H_8 with ellipsoids set at 30% probability. H atoms and a disordered molecule are omitted for clarity.

4-4. Preparation of **Li/Cu-3** (Figure 5b)

PCy_3 (140.2 mg, 0.500 mmol, 1.0 equiv) was added to a suspension of **Li/Cu-1** (322.7 mg, 0.500 mmol, 0.5 M in toluene) at rt, and the resultant mixture was stirred for 10 min, followed by removal of all volatiles in vacuo, affording **Li/Cu-3** as a gray solid (461.5 mg, 0.500 mmol, 99%). A single crystal of **Li/Cu-3** was obtained from a saturated solution of **Li/Cu-3** in toluene at rt. **^{1}H NMR** (400 MHz, CD_2Cl_2, rt, δ/ppm): 3.82–3.67 (m, 16H, O-CH_2), 3.28 (m, 2H, cage CH), 1.91–1.86 (m, 16H, CH_2-CH_2-CH_2), 3.06–1.47 (brm, BH), 1.41–1.22 (m, 18H, CH on PCy_3). **^{11}B NMR** (128 MHz, CD_2Cl_2, rt, δ/ppm): 0.02–−4.44 (m, 4B), −6.26–−14.47 (m, 16B). **^{13}C{^{1}H} NMR** (100 MHz, CD_2Cl_2, rt, δ/ppm): 75.7, 68.7 (t, J = 12.3 Hz), 31.7, 31.5, 31.4, 27.5 (d, J = 11.0 Hz), 26.3, 25.9. **^{31}P{^{1}H} NMR** (162 MHz, CD_2Cl_2, rt, δ/ppm): 29.8 (s, 1P, PCy$_3$). **X-ray** data for **2(Li/Cu-3)**: M = 1851.44, colorless, triclinic, P-1 (#2), a = 11.95758(14) Å, b = 14.1918(2) Å, c = 17.3976(3) Å, α = 106.0821(13)°, β = 102.3359(11)°, γ = 99.6132(11)°, V = 2689.68(7) Å^3, Z = 1, $Dcalcd$ = 1.143 g/cm^3 , T = 123.15 K, R_1 (wR_2) = 0.0462 (0.1390).

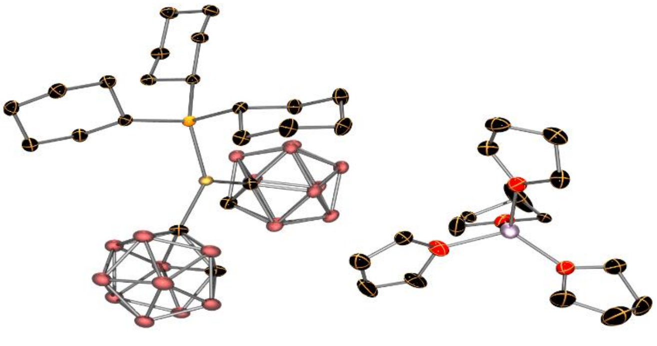

Figure S7. Molecular structure of **Li/Cu-3** with ellipsoids set at 30% probability. H atoms and a disordered molecule are omitted for clarity.

[5] Reaction between aryl halides and Li/Cu-1

General: In a screw-cap reaction vessel equipped with a magnetic stirrer bar, **Li/Cu-1** (1.0 equiv per molar amounts of Br or Cl units), isophthalonitrile (1.0 equiv with respect to **Li/Cu-1**), and the aryl halide were added. Toluene (0.5 M with respect to **Li/Cu-1**) was then added, and the vessel was sealed. The mixture was stirred at 100 °C (or 120 °C when aryl chlorides were used) for 16 h. After cooling to rt, the reaction mixture was diluted with wet Et_2O. The resulting mixture was filtered through filter paper, and the volatiles were removed under reduced pressure *(Note: to prevent the sublimation of o-carborane, removal of volatiles should be conducted at or below rt. If recovery of o-carborane is not necessary, evaporation can be carried out under heating conditions*). The crude product was purified by silica gel column chromatography (*n*-hexane/EtOAc) to afford the product.

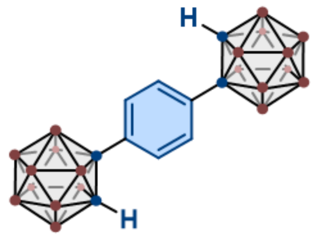

B1[5]: The general procedure was followed with **A1** (59.0 mg, 0.250 mmol), **Li/Cu-1** (322.7 mg, 0.500 mmol, 2.0 equiv), isophthalonitrile (64.1 mg, 0.500 mmol, 2.0 equiv), and toluene (1.0 mL). Purification by flash column chromatography on silica gel (*n*-hexane) afforded **B1** as a white solid (88.8 mg, 0.245 mmol, 98%). *The procedure for recovering o-carborane and isophthalonitrile is given in Section 8-1 (vide infra).* **^{1}H NMR** (400 MHz, $CDCl_3$, rt, δ/ppm): 7.45 (s, 4H, Ar-*H*), 3.92 (s, 2H, cage C*H*), 3.54–1.45 (brm, B*H*).

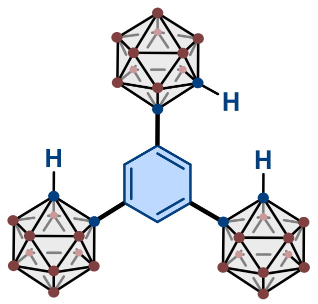

B2[6]: The general procedure was followed with **A2** (78.7 mg, 0.250 mmol), **Li/Cu-1** (484.0 mg, 0.750 mmol, 3.0 equiv), isophthalonitrile (96.1 mg, 0.750 mmol, 3.0 equiv), and toluene (1.5 mL). Purification by flash column chromatography on silica gel (*n*-hexane/EtOAc = 90/10) afforded **B2** as a white solid (113.5 mg, 0.225 mmol, 90%). A single crystal of **B2** was obtained from a saturated solution of **B2** in acetone at rt. **^{1}H NMR** (400 MHz, $CDCl_3$, rt, δ/ppm): 7.67 (s, 3H, Ar-*H*), 3.87 (s, 3H, cage C*H*), 3.29–1.55 (brm, B*H*). **X-ray** data for **B2·2(C_3H_6O)**: M = 620.86, colorless, monoclinic, $P2_1/n$ (#14), a = 6.8908(4) Å, b = 19.4046(10) Å, c = 27.7383(15) Å, α = 90°, β = 91.390(4)°, γ = 90°, V = 3707.9(4) Å^3, Z = 4, $Dcalcd$ = 1.112 g/cm^3 , T = 123.15 K, R_1 (wR_2) = 0.0555 (0.1288).

B2 was also synthesized by following the general procedure with **A2′** (45.4 mg, 0.250 mmol), **Li/Cu-1** (484.0 mg, 0.750 mmol, 3.0 equiv), isophthalonitrile (96.1 mg, 0.750 mmol, 3.0 equiv), and toluene (1.5 mL). Purification by flash column chromatography on silica gel (*n*-hexane/EtOAc = 90/10) afforded **B2** as a white solid (89.3 mg, 0.177 mmol, 71%).

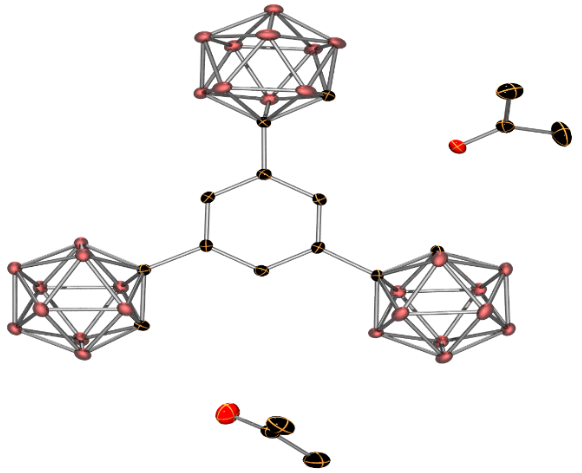

Figure S8. Molecular structure of **B2·2(C_3H_6O)** with ellipsoids set at 30% probability. H atoms are omitted for clarity.

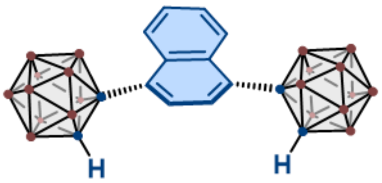

B3: The general procedure was followed with **A3** (71.5 mg, 0.250 mmol), **Li/Cu-1** (322.7 mg, 0.500 mmol, 2.0 equiv), isophthalonitrile (64.1 mg, 0.500 mmol, 2.0 equiv), and toluene (1.0 mL). Purification by flash column chromatography on silica gel (*n*-hexane/EtOAc = 95/5 to 85/15) afforded **B3** as a white solid (88.7 mg, 0.215 mmol, 86%). A single crystal of **B3** was obtained from a saturated solution of **B3** in acetone at rt. **^{1}H NMR** (400 MHz, $CDCl_3$, rt, δ/ppm): 8.88–8.81 (m, 2H, Ar-*H*), 7.69–7.60 (m, 4H), 4.56 (s, 2H, cage C*H*), 3.75–1.25 (brm, B*H*). **^{11}B NMR** (128 MHz, $CDCl_3$, rt, δ/ppm): −0.25–−3.57 (m, 4B), −6.23–−16.71 (m, 16B). **^{13}C{^{1}H} NMR** (100 MHz, $CDCl_3$, rt, δ/ppm): 131.8, 131.3, 127.2, 126.9, 125.6, 76.3, 61.5. **HRMS** (EI$^+$): m/z Calculated for $C_{14}H_{28}B_{20}$ ($[M]^+$) 416.4052, found 416.4110. **X-ray** data for **2(B3)**: M = 825.12, colorless, monoclinic, $P2_1/n$ (#14), a = 20.4921(5) Å, b = 11.6173(3) Å, c = 21.1929(5) Å, α = 90°, β = 113.695(3)°, γ = 90°, V = 4619.9(2) Å^3, Z = 4, $Dcalcd$ = 1.186 g/cm^3 , T = 153.15 K, R_1 (wR_2) = 0.0456 (0.1229).

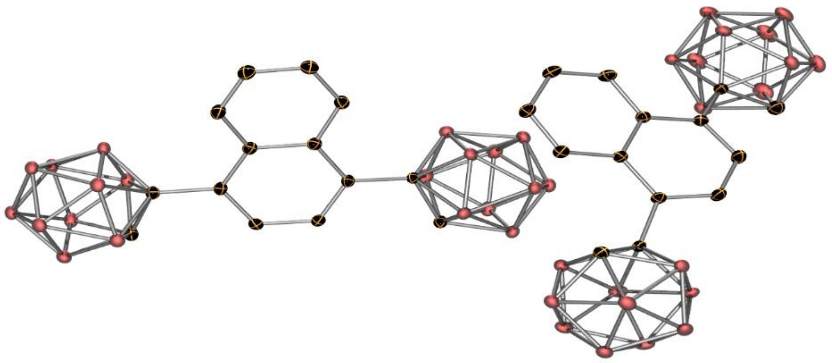

Figure S9. Molecular structure of **2(B3)** with ellipsoids set at 30% probability. H atoms are omitted for clarity.

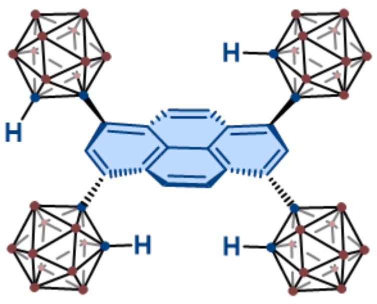

B4: The general procedure was followed with **A4** (129.5 mg, 0.250 mmol), **Li/Cu-1** (645.3 mg, 1.00 mmol, 4.0 equiv), isophthalonitrile (128.1 mg, 1.00 mmol, 4.0 equiv), and toluene (2.0 mL). Purification by flash column chromatography on silica gel (*n*-hexane/acetone = 2/1) afforded **B4** as a white solid (122.2 mg, 0.158 mmol, 63%). A single crystal of **B4** was obtained from a saturated solution of **B4** in EtOAc at rt. **^{1}H NMR** (400 MHz, $CDCl_3$/acetone-d_6 = 1/1, rt, δ/ppm): 8.69 (s, 4H, Ar-*H*), 8.24 (s, 2H, Ar-*H*), 5.02 (s, 4H, cage C*H*), 3.20–0.97 (brm, B*H*). **^{11}B NMR** (128 MHz, $CDCl_3$/acetone-d_6 = 1/1, rt, δ/ppm): 4.81–1.60 (m, 8B), −0.53–−12.02 (m, 32B). **^{13}C{^{1}H} NMR** (100 MHz, $CDCl_3$/EtOAc-d_8 = 1/1, rt, δ/ppm): 128.2, 126.9, 125.9, 125.2, 76.3, 62.1 (d, J = 7.2 Hz). One carbon resonance was observed at approximately 123.9 ppm, but it was not significant enough to be automatically detected, leaving its details unclear. **HRMS** (MALDI-TOF, m/z): Calculated for $C_{24}H_{50}{}^{10}B_{7}{}^{11}B_{33}$ ($[M]^+$) 771.7879, found 771.7896. **X-ray** data for **B4·2($C_4H_8O_2$)**: M = 1035.35, colorless, orthorhombic, $P2_12_12_1$ (#19), a = 11.6992(2) Å, b = 17.4486(3) Å, c = 28.7669(4) Å, α = 90°, β = 90°, γ = 90°, V = 5872.32(16) Å^3, Z = 4, $Dcalcd$ = 1.171 g/cm^3 , T = 153.15 K, R_1 (wR_2) = 0.0593 (0.1568). A solvent mask was calculated and 196 electrons were found in a volume of 708 Å^3 in 1 void per unit cell. This is consistent with the presence of 1[$CH_3COOCH_2CH_3$] per formula unit which accounts for 192 electrons per unit cell.

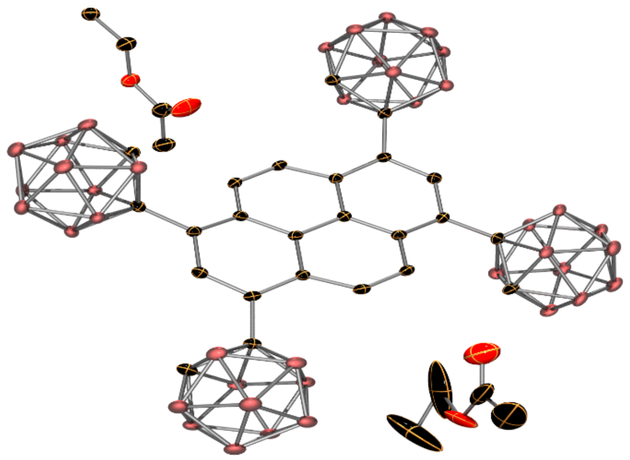

Figure S10. Molecular structure of **B4·2($C_4H_8O_2$)** with ellipsoids set at 30% probability. H atoms are omitted for clarity.

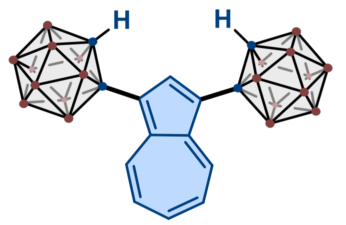

B5: The general procedure was followed with **A5** (71.5 mg, 0.250 mmol), **Li/Cu-1** (322.7 mg, 0.500 mmol, 2.0 equiv), isophthalonitrile (64.1 mg, 0.500 mmol, 2.0 equiv), and toluene (1.0 mL). Purification by flash column chromatography on silica gel (*n*-hexane/EtOAc = 95/5) afforded **B5** as a purple solid (97.7 mg, 0.237 mmol, 95%). A single crystal of **B5** was obtained from a saturated solution of **B5** in acetone at rt. **^{1}H NMR** (400 MHz, $CDCl_3$, rt, δ/ppm): 9.15 (d, J = 10.4 Hz, 2H, Ar-*H*), 8.08 (s, 1H, Ar-*H*), 7.93 (t, J = 9.8 Hz, 1H, Ar-*H*), 7.57 (dd, J = 10.4 Hz, 10.0 Hz, 2H, Ar-*H*), 4.04 (s, 2H, cage C*H*), 3.68–1.41 (brm, B*H*). **^{11}B NMR** (128 MHz, $CDCl_3$, rt, δ/ppm): −0.51–−5.02 (m, 4B), −6.09–−16.18 (m, 16B). **^{13}C{^{1}H} NMR** (100 MHz, $CDCl_3$, rt, δ/ppm): 141.8, 140.0 (d, J = 4.6 Hz), 138.5, 137.5, 127.7, 117.7, 74.1, 64.0 (d, J = 15.4 Hz). **HRMS** (EI^+): m/z Calculated for $C_{14}H_{28}B_{20}$ ($[M]^+$) 416.4052, found 416.4063. **X-ray** data

for **2(B5)**: *M* = 825.12, dark violet, triclinic, *P*-1 (#2), *a* = 12.02530(10) Å, *b* = 12.17120(10) Å, *c* = 17.05730(10) Å, *α* = 81.4290(10)°, *β* = 79.4140(10)°, *γ* = 73.8090(10)°, *V* = 2344.04(3) Å^3, *Z* = 2, *Dcalcd* = 1.169 g/cm^3 , *T* = 153.15 K, R_1 (wR_2) = 0.0476 (0.1267).

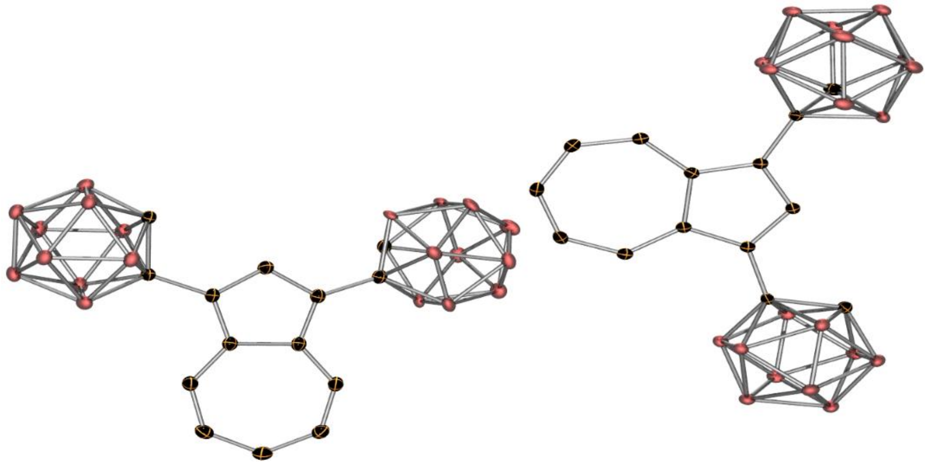

Figure S11. Molecular structure of **2(B5)** with ellipsoids set at 30% probability. H atoms and a disordered molecule are omitted for clarity.

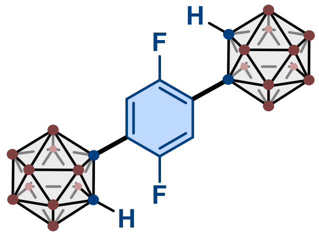

B6: The general procedure was followed with **A6** (68.0 mg, 0.250 mmol), **Li/Cu-1** (322.7 mg, 0.500 mmol, 2.0 equiv), isophthalonitrile (64.1 mg, 0.500 mmol, 2.0 equiv), and toluene (1.0 mL). Purification by flash column chromatography on silica gel (*n*-hexane/EtOAc = 95/5 to 85/15) afforded **B6** as a white solid (92.6 mg, 0.232 mmol, 93%). A single crystal of **B6** was obtained from a saturated solution of **B6** in acetone at rt. **^{1}H NMR** (400 MHz, $CDCl_3$, rt, *δ*/ppm): 7.35 (t, 2H, *J* = 10.0 Hz, Ar-*H*), 4.57 (s, 2H, cage C*H*), 3.35–1.39 (brm, B*H*). **^{11}B NMR** (128 MHz, $CDCl_3$, rt, *δ*/ppm): −0.04–−4.20 (m, 4B), −6.85–−16.02 (m, 16B). **^{13}C{^{1}H} NMR** (100 MHz, $CDCl_3$, rt, *δ*/ppm): 154.5 (dd, $^1J_{C,F}$ = 248.7 Hz, $^4J_{C,F}$ = 3.8 Hz, *C*-F), 124.4 (t, *J* = 10.1 Hz), 120.6 (m), 70.1, 58.9 (m). **^{19}F NMR** (376 MHz, $CDCl_3$, rt, *δ*/ppm): −121.2 (t, 2F, *J* = 10.0 Hz). **HRMS** (EI$^+$): m/z Calculated for $C_{10}H_{24}B_{20}F_2$ ([M]$^+$) 402.3707, found 402.3736. **X-ray** data for **1/2(B6)**: *M* = 199.25, colorless, monoclinic, $P2_1/c$ (#14), *a* = 7.2815(5) Å, *b* = 11.6189(9) Å, *c* = 12.6617(7) Å, *α* = 90°, *β* = 97.341(6)°, *γ* = 90°, *V* = 1062.44(13) Å^3, *Z* = 4, *Dcalcd* = 1.246 g/cm^3 , *T* = 153.15 K, R_1 (wR_2) = 0.0648 (0.1688).

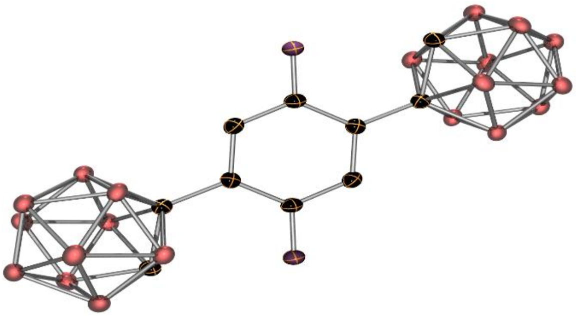

Figure S12. Molecular structure of **B6** with ellipsoids set at 30% probability. H atoms are omitted for clarity.

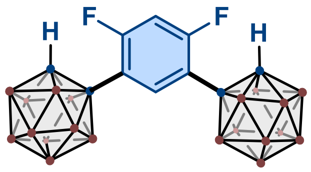

B7: The general procedure was followed with **A7** (4.84 g, 17.8 mmol), **Li/Cu-1** (23.0 g, 35.6 mmol, 2.0 equiv), isophthalonitrile (4.56 g, 35.6 mmol, 2.0 equiv), and toluene (71.2 mL). Purification by flash column chromatography on silica gel (*n*-hexane/EtOAc = 90/10) afforded **B7** as a white solid (3.49 g, 8.76 mmol, 49%). A single crystal of **B7** was obtained from a saturated solution of **B7** in acetone at rt. **^{1}H NMR** (400 MHz, $CDCl_3$, rt, δ/ppm): 7.98 (t, J = 8.8 Hz, 1H, Ar-*H*), 7.13 (t, J = 12.0 Hz, 1H, Ar-*H*), 4.52 (s, 2H, cage C*H*), 3.41–1.22 (brm, B*H*). **^{11}B NMR** (128 MHz, $CDCl_3$, rt, δ/ppm): 0.50–−4.98 (m, 4B), −7.01–−14.50 (m, 16B). **^{13}C{^{1}H} NMR** (100 MHz, $CDCl_3$, rt, δ/ppm): 159.3 (dd, $^{1}J_{C,F}$ = 256.9 Hz, $^{3}J_{C,F}$ = 13.6 Hz), 136.0, 119.1–118.7 (m), 107.1–106.3 (m), 70.6, 59.1–58.7 (m). **^{19}F NMR** (376 MHz, $CDCl_3$, rt, δ/ppm): −111.4 (t, 2F, $^{4}J_{F,F}$ = 10.2 Hz). **HRMS** (EI^+): m/z Calculated for $C_{10}H_{24}B_{20}$ ($[M]^+$) 402.3707, found 402.3795. **X-ray** data for **B7**: M = 398.49, colorless, monoclinic, $P2_1/c$ (#14), a = 6.99490(10) Å, b = 18.25550(10) Å, c = 16.97230(10) Å, α = 90°, β = 97.1190(10)°, γ = 90°, V = 2150.58(4) Å^3, Z = 4, *Dcalcd* = 1.231 g/cm^3 , T = 123.15 K, R_1 (wR_2) = 0.0407 (0.1061).

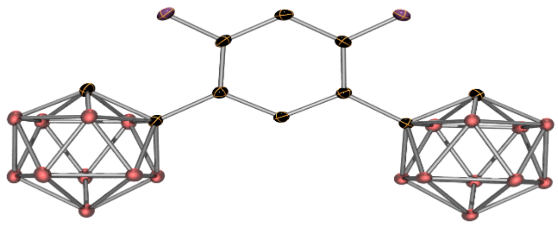

Figure S13. Molecular structure of **B7** with ellipsoids set at 30% probability. H atoms are omitted for clarity.

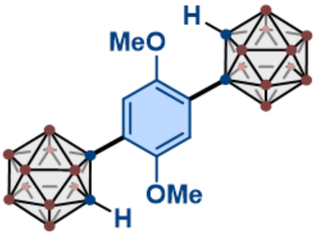

B8: The general procedure was followed with **A8** (74.0 mg, 0.250 mmol), **Li/Cu-1** (322.7 mg, 0.500 mmol, 2.0 equiv), isophthalonitrile (64.1 mg, 0.500 mmol, 2.0 equiv), and toluene (1.0 mL) , and the reaction was carried out for 32 h. Purification by flash column chromatography on silica gel (*n*-hexane/EtOAc = 90/10) afforded **B8** as a white solid (97.1 mg, 0.230 mmol, 92%). A single crystal of **B8** was obtained from a saturated solution of **B8** in acetone at rt. **^{1}H NMR** (400 MHz, $CDCl_3$, rt, δ/ppm):7.11 (s, 2H, Ar-*H*), 5.28 (s, 2H, cage C*H*). 3.83 (s, 6H, CH_3), 3.24–1.38 (brm, B*H*). **^{11}B NMR** (128 MHz, $CDCl_3$, rt, δ/ppm): −1.31–−5.15 (m, 4B), −6.71–−17.96 (m, 16B). **^{13}C{^{1}H} NMR** (100 MHz, $CDCl_3$, rt, δ/ppm): 149.8, 123.4, 116.9, 73.7, 59.6 (d, J = 11.1 Hz), 56.7 (d, J = 9.2 Hz). **HRMS** (EI^+): m/z Calculated for $C_{12}H_{30}B_{20}O_2$ ($[M]^+$) 426.4107, found 426.4159. **X-ray** data for **1/2(B8)**: M = 211.28, colorless, monoclinic, $C2/c$ (#15), a = 10.6054(9) Å, b = 10.5708(13) Å, c = 22.338(2) Å, α = 90°, β = 101.970(9)°, γ = 90°, V = 2449.9(4) Å^3, Z = 8, *Dcalcd* = 1.146 g/cm^3 , T = 263.15 K, R_1 (wR_2) = 0.0845 (0.215).

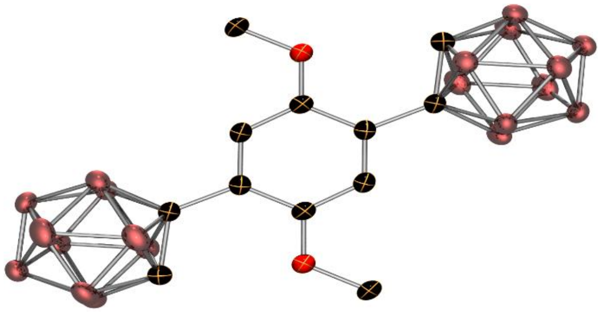

Figure S14. Molecular structure of **B8** with ellipsoids set at 30% probability. H atoms are omitted for clarity.

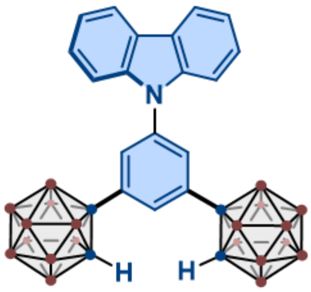

B9: The general procedure was followed with **A9** (100.3 mg, 0.250 mmol), **Li/Cu-1** (322.7 mg, 0.500 mmol, 2.0 equiv), isophthalonitrile (64.1 mg, 0.500 mmol, 2.0 equiv), and toluene (1.0 mL). Purification by flash column chromatography on silica gel (*n*-hexane/EtOAc = 95/5) afforded **B9** as a white solid (114.0 mg, 0.216 mmol, 86%). A single crystal of **B9** was obtained from a saturated solution of **B9** in acetone at rt. **^{1}H NMR** (400 [1.6 Hz, Ar-*H*), 7.70 (d, 1H, *J* = 1.6 Hz, Ar-*H*), 7.48 (m, 2H), 7.37 (t, 2H, *J* = 7.4 Hz, Ar-*H*), 7.31 (d, 2H, *J* = 8.0 Hz, Ar-*H*), 3.97 (s, 2H, cage C*H*), 3.51–1.47 (brm, B*H*). **^{11}B NMR** (128 MHz, $CDCl_3$, rt, *δ*/ppm): 1.83–−5.10 (m, 4B), −6.62–−18.64 (m, 16B). **^{13}C{^{1}H} NMR** (100 MHz, $CDCl_3$, rt, *δ*/ppm): 140.0, 139.3, 136.4, 127.3, 126.8, 125.5, 124.1, 121.4, 121.0, 109.0, 74.6, 60.2 (d, *J* = 12.9 Hz). **HRMS** (EI^+): m/z Calculated for $C_{22}H_{33}B_{20}N_1$ ($[M]^+$) 531.4474, found 531.4506. **X-ray** data for **B9·(C_3H_6O)**: *M* = 643.85, colorless, orthorhombic, $P2_12_12_1$ (#19), *a* = 7.1744(4) Å, *b* = 21.0702(13) Å, *c* = 24.1870(12) Å, *α* = 90°, *β* = 90°, *γ* = 90°, *V* = 3656.3(4) Å^3, *Z* = 4, *Dcalcd* = 1.170 g/cm^3 , *T* = 153.15 K, R_1 (wR_2) = 0.0485 (0.1042). A solvent mask was calculated and 108 electrons were found in a volume of 448 Å^3 in 1 void per unit cell. This is consistent with the presence of [C_3H_6O] per formula unit, which accounts for 128 electrons per unit cell.

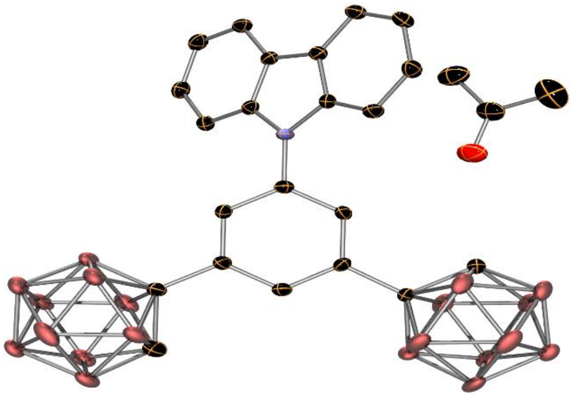

Figure S15. Molecular structure of **B9·(C_3H_6O)** with ellipsoids set at 30% probability. H atoms are omitted for clarity.

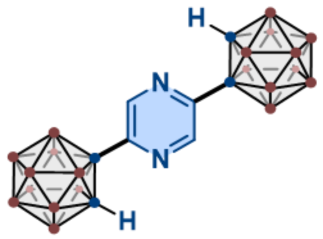

B10[7]: The general procedure was followed with **A10** (59.5 mg, 0.250 mmol), **Li/Cu-1** (322.7 mg, 0.500 mmol, 2.0 equiv), isophthalonitrile (64.1 mg, 0.500 mmol, 2.0 equiv), and toluene (1.0 mL). Purification by flash column chromatography on silica gel (*n*-hexane/EtOAc = 90/10) afforded **B10** as a white solid (47.8 mg, 0.131 mmol, 52%). A single crystal of **B10** was obtained from a saturated solution of **B10** in acetone at rt. **^{1}H NMR** (400 MHz, $CDCl_3$, rt, δ/ppm): 8.60 (s, 2H, Ar-*H*), 4.77 (s, 2H, cage C*H*), 3.42–1.33 (brm, B*H*). **X-ray** data for **1/2(B10)**: M = 182.25, colorless, monoclinic, $P2_1/c$ (#14), a = 7.1448(4) Å, b = 11.7803(7) Å, c = 12.3900(7) Å, α = 90°, β = 97.814(6)°, γ = 90°, V = 1033.16(10) Å^3, Z = 4, *Dcalcd* = 1.172 g/cm^3 , T = 153.15 K, R_1 (wR_2) = 0.0479 (0.1293).

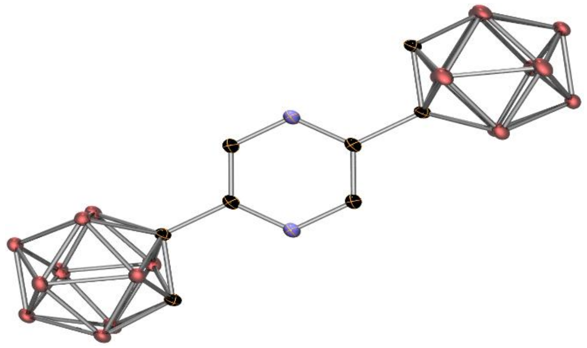

Figure S16. Molecular structure of **B10** with ellipsoids set at 30% probability. H atoms are omitted for clarity.

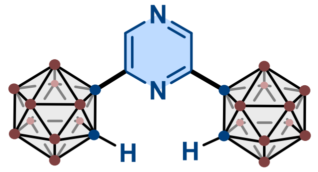

B11: The general procedure was followed with **A11** (59.5 mg, 0.250 mmol), **Li/Cu-1** (322.7 mg, 0.500 mmol, 2.0 equiv), isophthalonitrile (64.1 mg, 0.500 mmol, 2.0 equiv), and toluene (1.0 mL). Purification by flash column chromatography on silica gel (*n*-hexane/EtOAc = 90/10) afforded **B11** as a white solid (88.7 mg, 0.243 mmol, 97%). A single crystal of **B11** was obtained from a saturated solution of **B11** in acetone at rt. **^{1}H NMR** (400 MHz, $CDCl_3$, rt, δ/ppm): 8.90 (s, 2H, Ar-*H*), 4.42 (s, 2H, cage C*H*), 3.32–1.43 (brm, B*H*). **^{11}B NMR** (128 MHz, $CDCl_3$, rt, δ/ppm): −0.29–−4.68 (m, 4B), −6.04–−15.15 (m, 16B). **^{13}C{^{1}H} NMR** (100 MHz, $CDCl_3$, rt, δ/ppm): 145.6, 143.8 (d, J = 3.7 Hz), 71.5, 56.8 (d, J = 7.3 Hz). **HRMS** (EI$^+$): m/z Calculated for $C_8H_{24}B_{20}N_2$ ([M]$^+$) 368.3800, found 368.3871. **X-ray** data for **B11**: M = 364.49, colorless, monoclinic, $I1a1$ (#9), a = 11.9825(5) Å, b = 14.3028(5) Å, c = 12.7724(5) Å, α = 90°, β = 106.159(4)°, γ = 90°, V = 2102.50(15) Å^3, Z = 4, *Dcalcd* = 1.151 g/cm^3 , T = 153.15 K, R_1 (wR_2) = 0.0488 (0.1282).

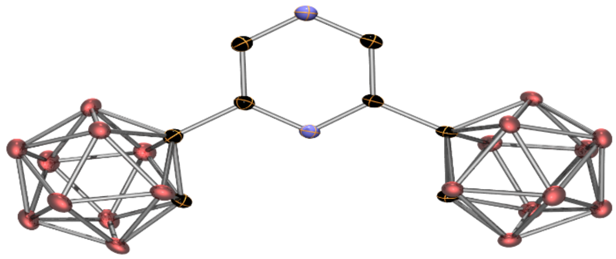

Figure S17. Molecular structure of **B11** with ellipsoids set at 30% probability. H atoms are omitted for clarity.

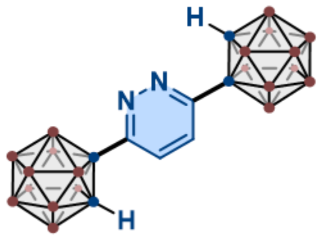

B12: The general procedure was followed with **A12** (59.5 mg, 0.250 mmol), **Li/Cu-1** (322.7 mg, 0.500 mmol, 2.0 equiv), isophthalonitrile (64.1 mg, 0.500 mmol, 2.0 equiv), and toluene (1.0 mL). Purification by flash column chromatography on silica gel (*n*-hexane/EtOAc = 90/10) afforded **B12** as a white solid (76.1 mg, 0.209 mmol, 83%). A single crystal of **B12** was obtained from a saturated solution of **B12** in acetone at rt. **^{1}H NMR** (400 MHz, $CDCl_3$, rt, δ/ppm): 7.74 (s, 2H, Ar-*H*), 5.03 (s, 2H, cage C*H*), 3.28–1.39 (brm, B*H*). **^{11}B NMR** (128 MHz, $CDCl_3$, rt, δ/ppm): −0.78–−4.87 (m, 4B), −6.93–−15.88 (m, 16B). **^{13}C{^{1}H} NMR** (100 MHz, $CDCl_3$, rt, δ/ppm): 155.5, 126.5, 71.8, 57.0 (d, J = 10.2 Hz). **HRMS** (EI$^+$): m/z Calculated for $C_8H_{24}B_{20}N_2$ ($[M]^+$) 368.3800, found 368.3856. **X-ray** data for **B12**: M = 364.49, colorless, orthorhombic, $Pna2_1$ (#33), a = 24.7124(3) Å, b = 6.75550(10) Å, c = 12.4271(2) Å, α = 90°, β = 90°, γ = 90°, V = 2074.64(5) Å^3, Z = 4, $Dcalcd$ = 1.167 g/cm^3 , T = 153.15 K, R_1 (wR_2) = 0.0374 (0.0967).

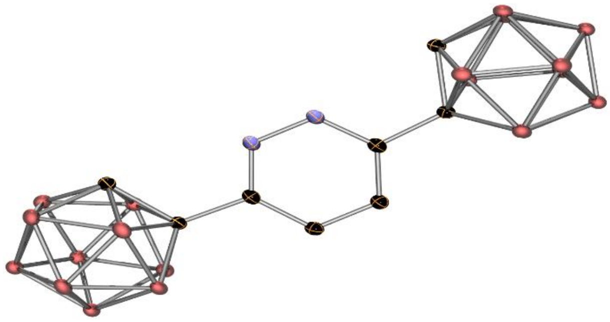

Figure S18. Molecular structure of **B12** with ellipsoids set at 30% probability. H atoms are omitted for clarity.

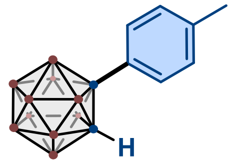

C13[8]: The general procedure was followed with **A13** (42.8 mg, 0.250 mmol), **Li/Cu-1** (161.3 mg, 0.250 mmol, 1.0 equiv), isophthalonitrile (32.0 mg, 0.250 mmol, 1.0 equiv), and toluene (0.5 mL). Purification by flash column chromatography on silica gel (*n*-hexane) afforded **C13** as a white solid (56.6 mg, 0.242 mmol, 97%). **^{1}H NMR** (400 MHz, $CDCl_3$, rt, δ/ppm): 7.36 (d, 2H, J = 8.4 Hz, Ar-*H*), 7.13 (d, 2H, J = 8.0 Hz, Ar-*H*), 3.92 (s, 1H, cage C*H*), 2.34 (s, 3H, CH_3), 3.32–1.64 (brm, B*H*).

C13 was also synthesized by following the general procedure with **A13′** (63.2 mg, 0.499 mmol), **Li/Cu-1** (322.7 mg, 0.500 mmol, 1.0 equiv), isophthalonitrile (64.1 mg, 0.500 mmol, 1.0 equiv), and toluene (1.0 mL). Purification by flash column chromatography on silica gel (*n*-hexane) afforded **C13** as a white solid (83.3 mg, 0.355 mmol, 71%).

C13 was also synthesized by following the general procedure with **A13″** (54.5 mg, 0.250 mmol), **Li/Cu-1** (161.3 mg, 0.250 mmol, 1.0 equiv), isophthalonitrile (32.0 mg, 0.250 mmol, 1.0 equiv), and toluene (0.5 mL), and the reaction was carried out for 48 h. Purification by flash column chromatography on silica gel (*n*-hexane) afforded **C13** as a white solid (55.7 mg, 0.238 mmol, 95%).

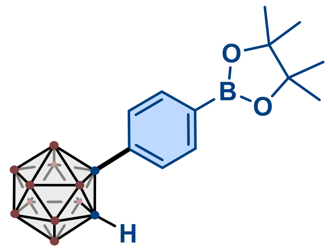

C14: The general procedure was followed with **A14** (70.7 mg, 0.250 mmol), **Li/Cu-1** (161.3 mg, 0.250 mmol, 1.0 equiv), isophthalonitrile (32.0 mg, 0.250 mmol, 1.0 equiv), and toluene (0.5 mL). Purification by flash column chromatography on silica gel (*n*-hexane/EtOAc = 80/20) afforded **C14** as a white solid (79.6 mg, 0.230 mmol, 92%). A single crystal of **C14** was obtained from a saturated solution of **C14** in acetone at rt. **^{1}H NMR** (400 MHz, $CDCl_3$, rt, δ/ppm): 7.76 (d, J = 8.4 Hz, 2H, Ar-*H*), 7.46 (d, J = 8.4 Hz, 2H, Ar-*H*), 3.99 (s, 1H, cage C*H*), 1.33 (s, 12H, C-CH_3), 3.01–1.66 (brm, B*H*). **^{11}B NMR** (128 MHz, $CDCl_3$, rt, δ/ppm): 30.3 (s, 1B, Ar-*B*Pin), −0.0–−6.6 (m, 2B), −6.9–−15.9 (m, 8B). **^{13}C{^{1}H} NMR** (100 MHz, $CDCl_3$, rt, δ/ppm): 136.2, 135.3, 126.7, 84.4, 76.5, 59.9 (d, J = 15.3 Hz), 25.0 (d, J = 6.6 Hz). A signal of the ipso-carbon bonded to the boron was not identified. **HRMS** (EI^+): m/z Calculated for $C_{14}H_{27}B_{11}O_2$ ($[M]^+$) 348.3030, found 348.3057. **X-ray** data for **C14**: M = 346.26, colorless, monoclinic, $P2_1/n$ (#14), a = 7.6804(3) Å, b = 23.5249(11) Å, c = 11.3975(4) Å, α = 90°, β = 91.154(4)°, γ = 90°, V = 2058.89(14) Å^3, Z = 4, $Dcalcd$ = 1.117 g/cm^3 , T = 153.15 K, R_1 (wR_2) = 0.0459 (0.1198).

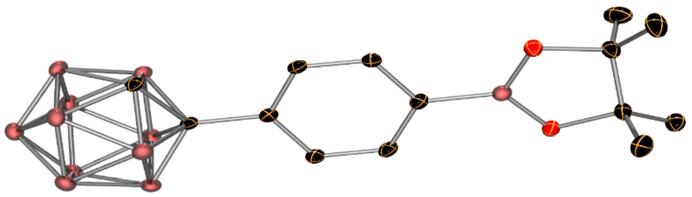

Figure S19. Molecular structure of **C14** with ellipsoids set at 30% probability. H atoms are omitted for clarity.

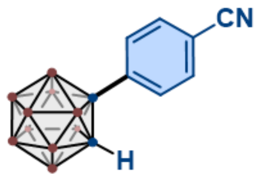

C15[9]: The general procedure was followed with **A15** (45.5 mg, 0.250 mmol), **Li/Cu-1** (161.3 mg, 0.250 mmol, 1.0 equiv), isophthalonitrile (32.0 mg, 0.250 mmol, 1.0 equiv), and toluene (0.5 mL). Purification by flash column chromatography on silica gel (*n*-hexane/EtOAc = 90/10) afforded **C15** as a white solid (60.4 mg, 0.246 mmol, 99%). **^{1}H NMR** (400 MHz, $CDCl_3$, rt, δ/ppm): 7.68–7.58 (m, 4H, Ar-*H*), 4.00 (s, 1H, cage C*H*), 3.49–1.45 (brm, B*H*). **HRMS** (EI^+): m/z Calculated for $C_9H_{15}B_{10}N_1$ ($[M]^+$) 247.2135, found 247.2141.

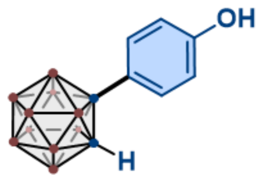

C16[10]: The general procedure was followed with **A16** (43.3 mg, 0.250 mmol), **Li/Cu-1** (242.0 mg, 0.375 mmol, 1.5 equiv), isophthalonitrile (48.0 mg, 0.375 mmol, 1.5 equiv), and toluene (0.75 mL). Purification by flash column chromatography on silica gel (*n*-hexane/EtOAc = 70/30) afforded **C16** as a white solid (47.6 mg, 0.202 mmol, 81%). **^{1}H NMR** (400 MHz, $CDCl_3$, rt, δ/ppm): 7.42–7.35 (m, 2H, Ar-*H*), 6.81–6.71 (m, 2H, Ar-*H*), 5.01 (s, 1H, O*H*), 3.86 (s, 1H, cage C*H*), 3.41–1.46 (brm, B*H*).

C16 was also synthesized by following the general procedure with **A16′** (64.2 mg, 0.499 mmol), **Li/Cu-1** (484.0 mg, 0.750 mmol, 1.5 equiv), isophthalonitrile (96.1 mg, 0.750 mmol, 1.5 equiv), and toluene (1.5 mL). Purification by flash column chromatography on silica gel (*n*-hexane/EtOAc = 70/30) afforded **C16** as a white solid (63.6 mg, 0.270 mmol, 54%).

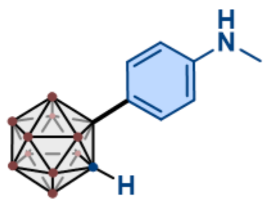

C17: The general procedure was followed with **A17** (93.0 mg, 0.500 mmol), **Li/Cu-1** (484.0 mg, 0.750 mmol, 3.0 equiv), isophthalonitrile (96.1 mg, 0.750 mmol, 3.0 equiv), and toluene (1.5 mL). Purification by flash column chromatography on silica gel (*n*-hexane/EtOAc = 85/15) afforded **C17** as a white solid (109.4 mg, 0.441 mmol, 88%). *The procedure for recovering o-carborane and isophthalonitrile is given in Section 8-2 (see Figure S33).* A single crystal of **C17** was obtained from a saturated solution of **C17** in acetone at rt. **^{1}H NMR** (400 MHz, $CDCl_3$, rt, δ/ppm): 7.30 (m, 2H, Ar-*H*), 6.47 (m, 2H, Ar-*H*), 3.95 (brs, N*H*), 3.82 (s, 1H, cage C*H*), 2.84 (s, 3H, CH_3), 3.44–1.22 (brm, B*H*). **^{11}B NMR** (128 MHz, $CDCl_3$, rt, δ/ppm): −0.50–−3.09 (m, 1B), −3.63–−6.63 (m, 1B), −8.17–−15.34 (m, 8B). **^{13}C{^{1}H} NMR** (100 MHz, $CDCl_3$, rt, δ/ppm): 150.4, 129.3, 121.6, 111.8 (d, J = 6.0 Hz), 78.2, 61.6 (d, J = 14.2 Hz), 30.4 (d, J = 8.4 Hz). **HRMS** (EI^+): m/z Calculated for $C_9H_{19}B_{10}N_1$ ($[M]^+$) 251.2448, found 251.2452. **X-ray** data for **C17**: M = 249.35, colorless, monoclinic, $P2_1/n$ (#14), a = 7.1546(2) Å, b = 18.9206(7) Å, c = 10.4931(4) Å, α = 90°, β = 92.876(3)°, γ = 90°, V = 1418.65(9) Å^3, Z = 4, $Dcalcd$ = 1.167 g/cm^3 , T = 153.15 K, R_1 (wR_2) = 0.0599 (0.1613).

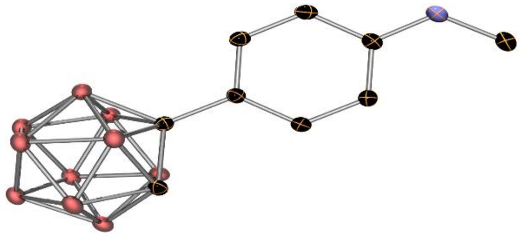

Figure S20. Molecular structure of **C17** with ellipsoids set at 30% probability. H atoms are omitted for clarity.

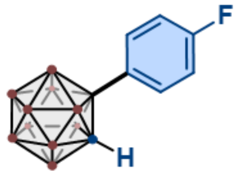

C18[11]: The general procedure was followed with **A18** (43.8 mg, 0.250 mmol), **Li/Cu-1** (161.3 mg, 0.250 mmol, 1.0 equiv), isophthalonitrile (32.0 mg, 0.250 mmol, 1.0 equiv), and toluene (0.5 mL). Purification by flash column chromatography on silica gel (*n*-hexane) afforded **C18** as a white solid (58.1 mg, 0.244 mmol, 97%). **^{1}H NMR** (400 MHz, $CDCl_3$, rt, δ/ppm): 7.52–7.44 (m, 2H, Ar-*H*), 7.05–6.99 (m, 2H, Ar-*H*), 3.90 (s, 1H, cage C*H*), 3.38–1.36 (brm, B*H*). **^{19}F NMR** (376 MHz, $CDCl_3$, rt, δ/ppm): −113.74–−113.84 (m, 1F, Ar-*F*).

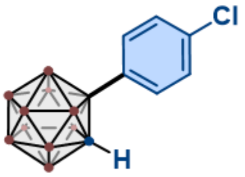

C19[11]: The general procedure was followed with **A19** (47.9 mg, 0.250 mmol), **Li/Cu-1** (161.3 mg, 0.250 mmol, 1.0 equiv), isophthalonitrile (32.0 mg, 0.250 mmol, 1.0 equiv), and toluene (0.5 mL). Purification by flash column chromatography on silica gel (*n*-hexane) afforded **C19** as a white solid (59.0 mg, 0.232 mmol, 93%) **^{1}H NMR** (400 MHz, $CDCl_3$, rt, δ/ppm): 7.46–7.39 (m, 2H, Ar-*H*), 7.34–7.28 (m, 2H, Ar-*H*), 3.92 (s, 1H, cageC*H*), 3.40–1.46 (brm, B*H*).

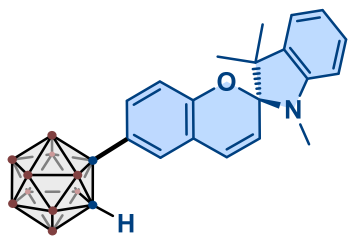

C20[12]: The general procedure was followed with **A20** (89.1 mg, 0.250 mmol), **Li/Cu-1** (161.3 mg, 0.250 mmol, 1.0 equiv), isophthalonitrile (32.0 mg, 0.250 mmol, 1.0 equiv), and toluene (0.5 mL). Purification by flash column chromatography on silica gel (*n*-hexane/EtOAc = 99/1 to 90/10) afforded **C20** as a pale yellow solid (83.2 mg, 0.198 mmol, 79%). A single crystal of **C20** was obtained from a saturated solution of **C20** in acetone at rt. **<u>^{1}H NMR</u>** (400 MHz, $CDCl_3$, rt, δ/ppm): 7.24–7.14 (m, 3H, Ar-*H*), 7.07 (d, J = 7.6 Hz, 1H, Ar-*H*), 6.89–6.78 (m, 2H, Ar-*H* and Ar-C*H*-CH), 6.63 (d, J = 7.6 Hz, 1H, Ar-*H*), 6.53 (d, J = 7.6 Hz, 1H, Ar-*H*), 5.76 (d, J = 10.4 Hz, 1H, C-C*H*-CH), 3.85 (s, 1H, cage C*H*), 2.71 (s, 3H, N-CH_3), 1.28 (s, 3H, C-CH_3), 1.16 (s, 3H, C-CH_3), 3.35–1.42 (brm, B*H*). **<u>^{11}B NMR</u>** (128 MHz, $CDCl_3$, rt, δ/ppm): −0.93–−5.68 (m, 2B), −8.04–−16.22 (m, 8B). **<u>^{13}C{^{1}H} NMR</u>** (100 MHz, $CDCl_3$, rt, δ/ppm): 155.9, 148.1, 136.5, 129.3, 128.7 (d, J = 4.8 Hz), 127.9, 126.6, 125.0, 121.7, 121.2, 119.6, 118.9, 115.3 (d, J = 5.6 Hz), 107.1, 105.3, 61.2 (d, J = 13.5 Hz), 52.1, 29.0 (d, J = 8.5 Hz), 26.0 (d, J = 4.6 Hz), 20.2 (d, J = 5.4 Hz). **<u>HRMS</u>** (EI^+): m/z Calculated for $C_{21}H_{29}B_{10}N_1O_1$ ($[M]^+$) 421.3180, found 421.3230. **<u>X-ray</u>** data for **C20**: M = 419.55, orange, orthorhombic, *Pbca* (#61), a = 14.9882(9) Å, b = 12.2029(7) Å, c = 25.5284(13) Å, α = 90°, β = 90°, γ = 90°, V = 4669.1(5) Å^3, Z = 8, $Dcalcd$ = 1.194 g/cm^3 , T = 153.15 K, R_1 (wR_2) = 0.0731 (0.1684).

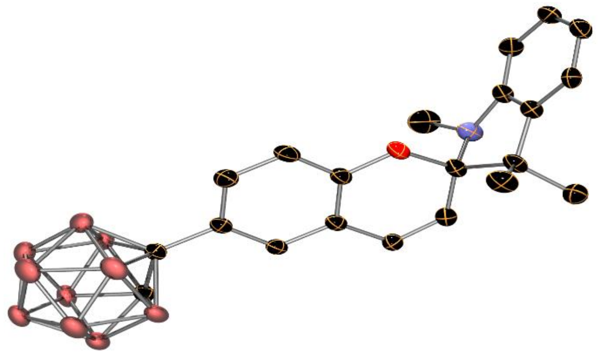

Figure S21. Molecular structure of **C20** with ellipsoids set at 30% probability. H atoms are omitted for clarity.

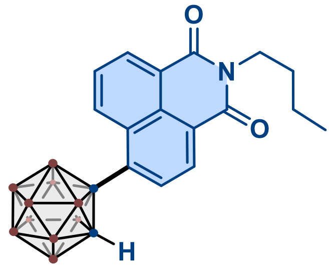

C21: The general procedure was followed with **A21** (83.0 mg, 0.250 mmol), **Li/Cu-1** (161.3 mg, 0.250 mmol, 1.0 equiv), isophthalonitrile (32.0 mg, 0.250 mmol, 1.0 equiv), and toluene (0.5 mL). Purification by flash column chromatography on silica gel (*n*-hexane only) afforded **C21** as a white solid (89.5 mg, 0.226 mmol, 91%). A single crystal of **C21** was obtained from a saturated solution of **C21** in acetone at rt. **<u>^{1}H NMR</u>** (400 MHz, $CDCl_3$, rt, δ/ppm): 9.16 (d, 1H, J = 9.2 Hz, Ar-*H*), 8.68 (d, 1H, J = 7.2 Hz, Ar-*H*), 8.52 (d, 1H, J = 8.0 Hz, Ar-*H*), 7.93 (d, 1H, J = 8.0 Hz, Ar-*H*), 7.86 (dd, 1H, J = 8.8 Hz, 7.2 Hz, Ar-*H*), 4.67 (s, 1H, cage C*H*), 4.16 (t, 2H, J = 7.6 Hz, N-CH_2), 1.70 (quin, 2H, J = 7.6 Hz, CH_2-CH_2-CH_2) 1.43 (sx, 2H, J = 7.5 Hz, CH_2-CH_2-CH_3), 0.97 (t, 3H, J = 7.2 Hz, CH_2-CH_3), 3.63–1.50 (brm, B*H*). **<u>^{11}B NMR</u>** (128 MHz, $CDCl_3$, rt, δ/ppm): −0.04–−3.85 (m, 2B), −6.47–−15.71 (m, 8B). **<u>^{13}C{^{1}H} NMR</u>** (100 MHz, $CDCl_3$, rt, δ/ppm): 163.8, 163.1, 135.4, 131.8, 131.2, 129.7, 129.6, 129.0, 127.8, 124.7, 123.8, 76.0, 61.4, 40.6, 30.2, 20.5, 13.9. **<u>HRMS</u>** (EI^+): m/z Calculated for $C_{18}H_{25}B_{10}N_1O_2$ ($[M]^+$) 397.2816, found 397.2840. **<u>X-ray</u>** data for **C21**: M = 395.49, colorless, triclinic, *P*-1 (#2), a = 7.3843(3)

Å, b = 8.7494(4) Å, c = 16.6349(8) Å, α = 77.660(4)°, β = 78.871(4)°, γ = 88.299(4)°, V = 1030.10(8) Å^3, Z = 2, $Dcalcd$ = 1.275 g/cm^3 , T = 153.15 K, R_1 (wR_2) = 0.0578 (0.1508).

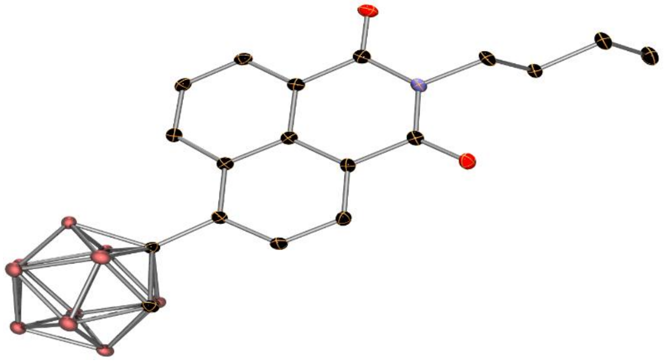

Figure S22. Molecular structure of **C21** with ellipsoids set at 30% probability. H atoms are omitted for clarity.

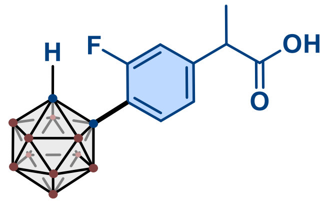

C22: The general procedure was followed with **A22** (61.8 mg, 0.250 mmol), **Li/Cu-1** (322.7 mg, 0.500 mmol, 2.0 equiv), isophthalonitrile (64.1 mg, 0.500 mmol, 2.0 equiv), and toluene (1.0 mL). Purification by flash column chromatography on silica gel (n-hexane/EtOAc = 1/2 to 2/3) afforded **C22** as a white solid (47.3 mg, 0.152 mmol, 61%). A single crystal of **C22** was obtained from a saturated solution of **C22** in acetone at rt. **^{1}H NMR** (400 MHz, $CDCl_3$, rt, δ/ppm): 7.57 (t, J = 8.6 Hz, 1H, Ar-H), 7.11 (d, J = 8.4 Hz, 1H, Ar-H), 7.04 (d, J = 14.4 Hz, 1H, Ar-H), 4.63 (s, 1H, cage CH), 3.73 (q, J = 7.1 Hz, 1H, CH_3-CH), 1.51 (d, J = 7.2 Hz, 3H, CH-CH_3), 3.36–1.15 (brm, BH). **^{11}B NMR** (128 MHz, $CDCl_3$, rt, δ/ppm): −1.37– −4.20 (m, 2B), −4.85–−16.10 (m, 8B). **^{13}C{^{1}H} NMR** (100 MHz, $CDCl_3$, rt, δ/ppm): 178.6, 159.0 (d, $^1J_{C,F}$ = 248.8 Hz), 143.9 (d, J = 8.7 Hz), 132.2, 124.5, 120.2 (d, J = 8.4 Hz), 116.4 (d, J = 24.9 Hz), 72.1, 59.4 (d, J = 18.6 Hz), 44.5, 18.0. **^{19}F NMR** (376 MHz, $CDCl_3$, rt, δ/ppm): −117.7 (s, 1F). **HRMS** (EI$^+$): m/z Calculated for $C_{11}H_{19}B_{10}F_1O_2$ ([M]$^+$) 312.2300, found 312.2322. **X-ray** data for **C22**: M = 310.36, colorless, triclinic, P-1 (#2), a = 6.88599(18) Å, b = 9.9543(3) Å, c = 12.7186(2) Å, α = 96.886(2)°, β = 101.2502(19)°, γ = 101.555(2)°, V = 826.17(4) Å^3, Z = 2, $Dcalcd$ = 1.248 g/cm^3, T = 123.15 K, R_1 (wR_2) = 0.0539 (0.1406).

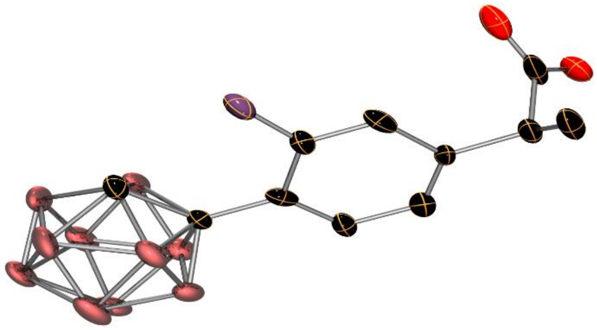

Figure S23. Molecular structure of **C22** with ellipsoids set at 30% probability. One of the disordered molecules is shown. H atoms are omitted for clarity.

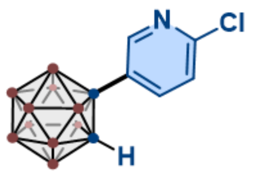

C23: The general procedure was followed with **A23** (48.1 mg, 0.250 mmol), **Li/Cu-1** (161.3 mg, 0.250 mmol, 1.0 equiv), isophthalonitrile (32.0 mg, 0.250 mmol, 1.0 equiv), and toluene (0.5 mL). Purification by flash column chromatography on silica gel (*n*-hexane/EtOAc = 90/10) afforded **C23** as a white solid (50.9 mg, 0.199 mmol, 80%). A single crystal of **C23** was obtained from a saturated solution of **C23** in acetone at rt. **^{1}H NMR** (400 MHz, $CDCl_3$, rt, δ/ppm): 8.52 (d, 1H, J = 2.8 Hz, Ar-*H*), 7.78 (dd, 1H, J = 8.4 H, 2.8 Hz, Ar-*H*), 7.33 (d, 1H, J = 8.8 Hz, Ar-*H*), 3.92 (s, 1H, cage C*H*), 3.42–1.41 (brm, B*H*). **^{11}B NMR** (128 MHz, $CDCl_3$, rt, δ/ppm): −0.02–−5.34 (m, 2B), −7.17–−15.25 (m, 8B). **^{13}C{^{1}H} NMR** (100 MHz, $CDCl_3$, rt, δ/ppm): 153.5, 148.5, 138.3, 128.8, 124.3, 72.7, 60.3. **HRMS** (EI^+): m/z Calculated for $C_7H_{14}B_{10}Cl_1N_1$ ($[M]^+$) 257.1745, found 257.1766. **X-ray** data for **C23**: M = 255.74, colorless, orthorhombic, $Pna2_1$ (#33), a = 26.0585(9) Å, b = 7.1993(3) Å, c = 7.1951(3) Å, α = 90°, β = 90°, γ = 90°, V = 1349.82(9) Å^3, Z = 4, $Dcalcd$ = 1.258 g/cm^3 , T = 153.15 K, R_1 (wR_2) = 0.0310 (0.0829).

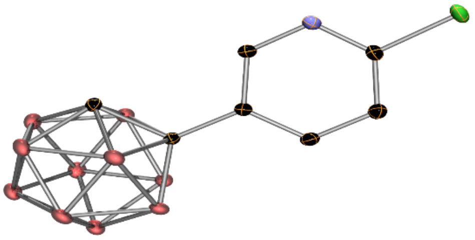

Figure S24. Molecular structure of **C23** with ellipsoids set at 30% probability. H atoms are omitted for clarity.

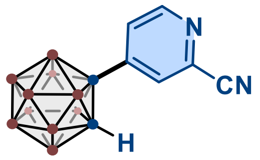

C24: The general procedure was followed with aryl chloride **A24′** (69.3 mg, 0.500 mmol), **Li/Cu-1** (322.7 mg, 0.500 mmol, 2.0 equiv), isophthalonitrile (64.1 mg, 0.500 mmol, 2.0 equiv), and toluene (1.0 mL). Purification by flash column chromatography on silica gel (*n*-hexane/EtOAc = 90/10) afforded **C24** as a white solid (31.9 mg, 0.130 mmol, 26%). A single crystal of **C24** was obtained from a saturated solution of **C24** in acetone at rt. **^{1}H NMR** (400 MHz, $CDCl_3$, rt, *δ*/ppm): 8.78–8.71 (m, 1H, Ar-*H*), 7.74 (s, 1H, Ar-*H*), 7.59 (d, 1H, *J* = 4.8 Hz, Ar-*H*), 4.00 (s, 1H, cage C*H*), 3.30–1.05 (brm, B*H*). **^{11}B NMR** (128 MHz, $CDCl_3$, rt, *δ*/ppm): 0.14–−3.74 (m, 2B), −6.03–−15.32 (m, 8B). **^{13}C{^{1}H} NMR** (100 MHz, $CDCl_3$, rt, *δ*/ppm): 151.9, 143.9, 135.2, 126.5, 125.0, 116.3, 72.1, 59.1 (d, *J* = 13.8 Hz). **HRMS** (EI^+): m/z Calculated for $C_8H_{14}B_{10}N_2$ ($[M]^+$) 248.2087, found 248.2085. **X-ray** data for **2(C24)**: M = 492.62, colorless, monoclinic, $P2_1/c$ (#14), a = 6.7996(2) Å, b = 20.5686(6) Å, c = 19.3753(5) Å, α = 90°, β = 91.061(2)°, γ = 90°, V = 2709.33(13) Å^3, Z = 4, *Dcalcd* = 1.208 g/cm^3 , T = 153.15 K, R_1 (wR_2) = 0.0663 (0.1602).

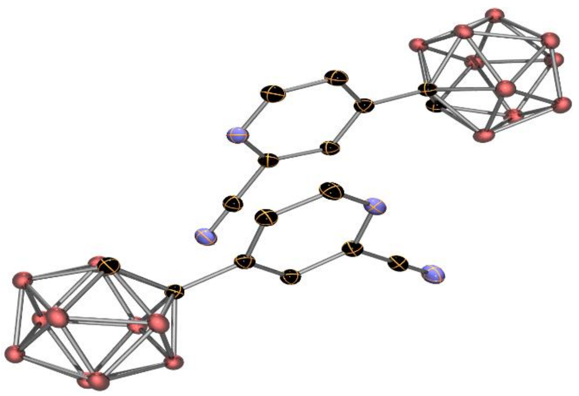

Figure S25. Molecular structure of **2(C24)** with ellipsoids set at 30% probability. H atoms are omitted for clarity.

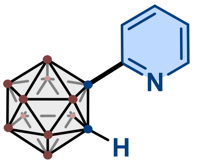

C25[13]:The general procedure was followed with **A25** (39.5 mg, 0.250 mmol), **Li/Cu-1** (161.3 mg, 0.250 mmol, 1.0 equiv), isophthalonitrile (32.0 mg, 0.250 mmol, 1.0 equiv), and toluene (0.5 mL). Purification by flash column chromatography on silica gel (*n*-hexane/EtOAc = 90/10) afforded **C25** as a white solid (47.1 mg, 0.213 mmol, 85%). **^{1}H NMR** (400 MHz, $CDCl_3$, rt, *δ*/ppm): 8.41 (d, *J* = 4.8 Hz, 1H, Ar-*H*), 7.70 (t, *J* = 7.8 Hz, 1H, Ar-*H*), 7.53 (d, *J* = 8.0 Hz, 1H, Ar-*H*), 7.31 (dd, *J* = 7.8 Hz, *J* = 5.0 Hz, 1H, Ar-*H*), 4.99 (s, 1H, cage C*H*), 3.351–0.73 (brm, B*H*).

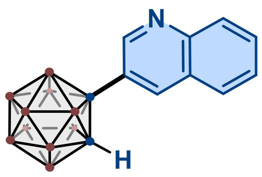

C26: The general procedure was followed with **A26** (52.0 mg, 0.250 mmol), **Li/Cu-1** (161.3 mg, 0.250 mmol, 1.0 equiv), isophthalonitrile (32.0 mg, 0.250 mmol, 1.0 equiv), and toluene (0.5 mL). Purification by flash column chromatography on silica gel (*n*-hexane/EtOAc = 90/10) afforded **C26** as a white solid (57.6 mg, 0.212 mmol, 85%). A single crystal of **C26** was obtained from a saturated solution of **C26** in acetone at rt. **^{1}H NMR** (400 MHz, $CDCl_3$, rt, *δ*/ppm): 8.99 (d, *J* = 2.0 Hz, 1H, Ar-*H*), 8.30 (d, *J* = 2.0 Hz, 1H, Ar-*H*), 8.12 (d, *J* = 8.4 Hz, 1H, Ar-*H*), 7.84 (d, *J* = 8.0 Hz, 1H, Ar-*H*), 7.80 (t, *J* = 7.6 Hz, 1H, Ar-*H*), 7.64 (t, *J* = 7.8 Hz, 1H, Ar-*H*), 4.07 (s, 1H, cage C*H*), 3.62–1.11 (brm, B*H*). **^{11}B NMR** (128 MHz, $CDCl_3$, rt, *δ*/ppm): 0.03–−4.99 (m, 2B), −6.09–−15.73 (m, 8B).

^{13}C{^{1}H} NMR (100 MHz, $CDCl_3$, rt, δ/ppm): 148.3, 148.2, 135.8, 131.5, 129.5, 128.4–127.9 (m, two kinds of peaks overlapped), 126.6, 126.4, 74.2, 60.6 (d, *J* = 15.8 Hz). **HRMS** (EI^+): m/z Calculated for $C_{10}H_{24}B_{20}$ ($[M]^+$) 273.2291, found 273.2301. **X-ray** data for **C26**: *M* = 271.35, colorless, orthorhombic, $Pna2_1$ (#33), *a* = 6.98320(10) Å, *b* = 21.0279(3) Å, *c* = 10.1776(2) Å, α = 90°, β = 90°, γ = 90°, *V* = 1494.50(4) Å^3, *Z* = 4, *Dcalcd* = 1.206 g/cm^3 , *T* = 123.15 K, R_1 (wR_2) = 0.0433 (0.1106).

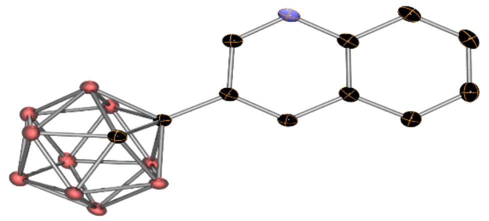

Figure S26. Molecular structure of **C26** with ellipsoids set at 30% probability. H atoms are omitted for clarity.

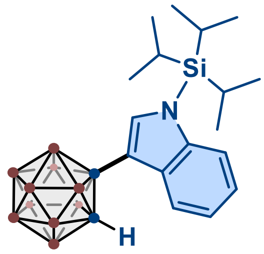

C27: The general procedure was followed with **A27** (88.1 mg, 0.250 mmol), **Li/Cu-1** (161.3 mg, 0.250 mmol, 1.0 equiv), isophthalonitrile (32.0 mg, 0.250 mmol, 1.0 equiv), and toluene (0.5 mL). Purification by flash column chromatography on silica gel (*n*-hexane) afforded **C27** as a white solid (101.0 mg, 0.243 mmol, 97%). A single crystal of **C27** was obtained from a saturated solution of **C27** in acetone at rt. **^{1}H NMR** (400 MHz, $CDCl_3$, rt, δ/ppm): 7.96–7.89 (m, 1H, Ar-*H*), 7.54–7.46 (m, 2H, Ar-*H*), 7.25–7.17 (m, 2H, Ar-*H*), 3.96 (s, 1H, cage C*H*), 1.69 (m, 3H, C*H*(CH_3)$_2$), 1.15 (d, 18H, *J* = 7.6 Hz, CH(C*H*$_3$)$_2$), 3.60–1.46 (brm, B*H*). **^{11}B NMR** (128 MHz, $CDCl_3$, rt, δ/ppm): −0.49–−5.97 (m, 2B), −6.79–−17.52 (m, 8B). **^{13}C{^{1}H} NMR** (100 MHz, $CDCl_3$, rt, δ/ppm): 141.2, 133.8 (d, *J* = 2.2 Hz), 128.2, 122.7, 121.3, 119.8, 114.7, 112.0, 73.7, 63.9 (d, *J* = 13.6 Hz), 18.1, 12.9. **HRMS** (EI^+): m/z Calculated for $C_{19}H_{37}B_{10}N_1Si_1$ ($[M]^+$) 417.3626, found 417.3669. **X-ray** data for **C27**: *M* = 415.68, colorless, monoclinic, $P2_1/c$ (#14), *a* = 13.7789(4) Å, *b* = 7.3417(2) Å, *c* = 24.5423(7) Å, α = 90°, β = 91.987(3)°, γ = 90°, *V* = 2481.22(12) Å^3, *Z* = 4, *Dcalcd* = 1.113 g/cm^3, *T* = 153.15 K, R_1 (wR_2) = 0.0363 (0.0976).

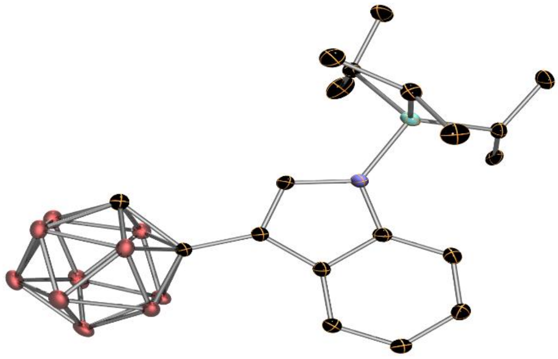

Figure S27. Molecular structure of **C27** with ellipsoids set at 30% probability. H atoms and a disordered molecule are omitted for clarity.

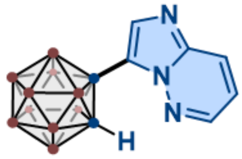

C28: The general procedure was followed with **A28** (49.5 mg, 0.250 mmol), **Li/Cu-1** (161.3 mg, 0.250 mmol, 1.0 equiv), isophthalonitrile (32.0 mg, 0.250 mmol, 1.0 equiv), and toluene (0.5 mL). Purification by flash column chromatography on silica gel (*n*-hexane/EtOAc = 90/10) afforded **C28** as a white solid (61.0 mg, 0.233 mmol, 93%). A single crystal of **C28** was obtained from a saturated solution of **C28** in acetone at rt. **^{1}H NMR** (400 MHz, $CDCl_3$, rt, δ/ppm): 8.40 (d, J = 3.6 Hz, 1H, C*H*-N), 8.03 (d, J = 8.8 Hz, 1H, C-C*H*-CH), 7.95 (s, 1H, N-C*H*-C), 7.15 (dd, 1H, J = 9.2 Hz, 4.4 Hz, CH-C*H*-CH), 5.52 (s, 1H, cage C*H*), 3.49–0.60 (brm, B*H*). **^{11}B NMR** (128 MHz, $CDCl_3$, rt, δ/ppm): −1.29–−5.00 (m, 2B), −6.63–−16.2 (m, 8B). **^{13}C{^{1}H} NMR** (100 MHz, DMSO-d_6, rt, δ/ppm): 144.3, 140.0, 136.4 (d, J = 4.7 Hz), 126.4, 119.3, 119.1, 67.3, 63.1. **HRMS** (DART$^+$, m/z): Calculated for $C_8H_{16}B_{10}N_3$ ([M+H]$^+$) 264.22693, found 264.22858. **X-ray** data for **C28**: M = 261.33, colorless, monoclinic, $P2_1/n$ (#14), a = 6.9911(2) Å, b = 9.4471(3) Å, c = 20.9966(6) Å, α = 90°, β = 96.610(3)°, γ = 90°, V = 1377.52(7) Å^3, Z = 4, $Dcalcd$ = 1.260 g/cm^3, T = 123.15 K, R_1 (wR_2) = 0.0411 (0.1152).

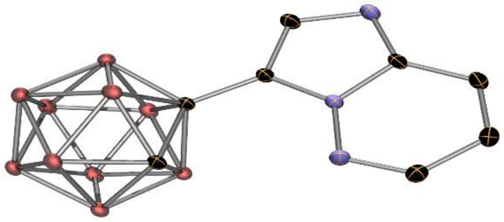

Figure S28. Molecular structure of **C28** with ellipsoids set at 30% probability. H atoms are omitted for clarity.

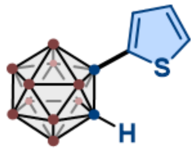

C29[14]: The general procedure was followed with **A29** (40.8 mg, 0.250 mmol), **Li/Cu-1** (161.3 mg, 0.250 mmol, 1.0 equiv), isophthalonitrile (32.0 mg, 0.250 mmol, 1.0 equiv), and toluene (0.5 mL). Purification by flash column chromatography on silica gel (*n*-hexane only) afforded **C29** as a white solid (56.4 mg, 0.249 mmol, 99%). **^{1}H NMR** (400 MHz, $CDCl_3$, rt, δ/ppm): 7.29–7.17 (m, 2H, S-C*H*-CH and C-C*H*-CH, overlapped with $CHCl_3$), 6.91 (dd, 1H, J = 5.2 Hz, 4.0 Hz, CH-C*H*-CH), 3.85 (s, 1H, cage C*H*), 3.58–1.40 (brm, B*H*).

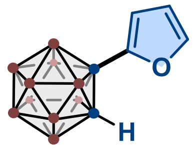

C30: The general procedure was followed with **A30** (73.5 mg, 0.500 mmol), **Li/Cu-1** (322.7 mg, 0.500 mmol, 2.0 equiv), isophthalonitrile (64.1 mg, 0.500 mmol, 2.0 equiv), and toluene (1.0 mL). Purification by flash column chromatography on silica gel (*n*-hexane only) afforded **C30** as a colorless liquid (100.0 mg, 0.476 mmol, 95%). **^{1}H NMR** (400 MHz, $CDCl_3$, rt, δ/ppm): 7.31 (m, 1H, O-C*H*-CH), 6.51 (d, 1H, J = 3.6 Hz, CH-C*H*-C), 6.34 (dd, 1H, J = 3.4 Hz, 1.8 Hz, CH-C*H*-CH), 3.93 (s, 1H, cage C*H*), 3.56–1.41 (brm, B*H*). **^{11}B NMR** (128 MHz, $CDCl_3$, rt, δ/ppm): 0.40–−5.58 (m, 2B), −7.44–−15.35 (m, 8B). **^{13}C{^{1}H} NMR** (100 MHz, $CDCl_3$, rt, δ/ppm): 146.0, 143.7, 111.7 (d, J = 6.1 Hz), 111.2 (d, J = 6.8 Hz), 68.5, 60.8 (d, J = 14.1 Hz). **HRMS** (EI$^+$): m/z Calculated for $C_6H_{14}B_{10}O_1$ ([M]$^+$) 212.1975, found 212.1986.

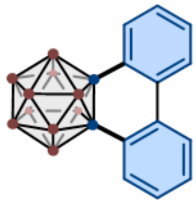

D31[15]: The general procedure was followed with **A31** (78.0 mg, 0.250 mmol), **Li/Cu-1** (322.7 mg, 0.500 mmol, 2.0 equiv), isophthalonitrile (64.1 mg, 0.500 mmol, 2.0 equiv), and toluene (1.0 mL). Purification by flash column chromatography on silica gel (*n*-hexane only) afforded **D31** as a white solid (69.4 mg, 0.236 mmol, 94%). **^{1}H NMR** (400 MHz, $CDCl_3$, rt, δ/ppm): 8.11 (d, 2H, J = 7.6 Hz, Ar-H), 7.72 (dd, 2H, J = 8.0 Hz, J = 1.2 Hz, Ar-H), 7.52 (td, 2H, J = 7.6 Hz, J = 1.2 Hz, Ar-H), 7.42 (td, 2H, J = 7.6 Hz, J = 1.2 Hz, Ar-H), 3.65–1.04 (brm, BH).

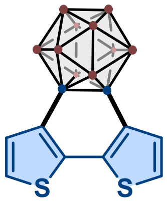

D32[16]: The general procedure was followed with **A32** (81.0 mg, 0.250 mmol), **Li/Cu-1** (322.7 mg, 0.500 mmol, 2.0 equiv), isophthalonitrile (64.1 mg, 0.500 mmol, 2.0 equiv), and toluene (1.0 mL). Purification by flash column chromatography on silica gel (*n*-hexane/EtOAc = 97/3) afforded **D32** as a white solid (74.0 mg, 0.241 mmol, 97%). **^{1}H NMR** (400 MHz, $CDCl_3$, rt, δ/ppm): 7.29 (d, J = 5.2 Hz, 2H, S-CH), 7.22 (d, J = 5.2 Hz, 2H, C-CH-CH), 3.64–0.76 (brm, BH).

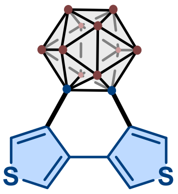

D33: The general procedure was followed with **A33** (81.0 mg, 0.250 mmol), **Li/Cu-1** (322.7 mg, 0.500 mmol, 2.0 equiv), isophthalonitrile (64.1 mg, 0.500 mmol, 2.0 equiv), and toluene (1.0 mL). Purification by flash column chromatography on silica gel (*n*-hexane/EtOAc = 97/3) afforded **D33** as a white solid (66.2 mg, 0.216 mmol, 86%). A single crystal of **D33** was obtained from a saturated solution of **D33** in acetone at rt. **^{1}H NMR** (400 MHz, $CDCl_3$, rt, δ/ppm): 7.55–7.48 (m, 4H, S-CH), 3.40–1.53 (m, cage BH), 3.59–1.55 (brm, BH). **^{11}B NMR** (128 MHz, $CDCl_3$, rt, δ/ppm): −4.18–−6.79 (m, 2B), −7.99–−13.08 (m, 8B). **^{13}C{^{1}H} NMR** (100 MHz, $CDCl_3$, rt, δ/ppm): 131.4, 128.4, 124.8, 119.6, 71.4. **HRMS** (EI^+): m/z Calculated for $C_{10}H_{14}B_{10}S_2$ ($[M]^+$) 308.1467, found 308.1495. **X-ray** data for **2**(**D33**): M = 612.86, colorless, monoclinic, $P2_1/c$ (#14), a = 13.8635(2) Å, b = 11.52150(10) Å, c = 20.2307(3) Å, α = 90°, β = 108.359(2)°, γ = 90°, V = 3066.94(8) Å^3, Z = 4, $Dcalcd$ = 1.327 g/cm^3, T = 123.15 K, R_1 (wR_2) = 0.0453 (0.1236).

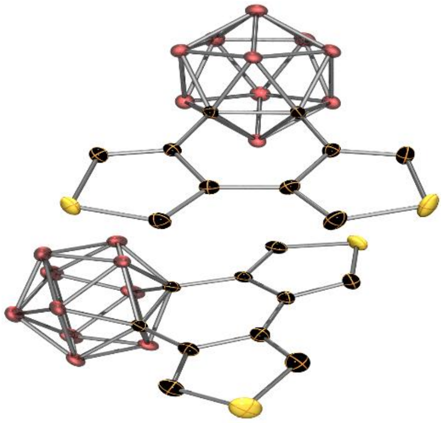

Figure S29. Molecular structure of **2(D33)** with ellipsoids set at 30% probability. H atoms are omitted for clarity.

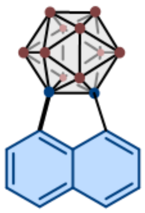

D34[17]: The general procedure was followed with **A34** (71.5 mg, 0.250 mmol), **Li/Cu-1** (322.7 mg, 0.500 mmol, 2.0 equiv), isophthalonitrile (64.1 mg, 0.500 mmol, 2.0 equiv), and toluene (1.0 mL). Purification by flash column chromatography on silica gel (*n*-hexane only) afforded **D34** as a white solid (26.8 mg, 0.100 mmol, 40%). A single crystal of **D34** was obtained from a saturated solution of **D34** in acetone at rt. **^{1}H NMR** (400 MHz, $CDCl_3$, rt, *δ*/ppm): 7.80 (d, 2H, *J* = 8.0 Hz, Ar-*H*), 7.59 (d, 2H, *J* = 7.2 Hz, Ar-*H*), 7.52 (dd, 2H, *J* = 8.4 Hz, 7.2 Hz, Ar-*H*), 3.42–1.20 (brm, B*H*). **^{11}B NMR** (128 MHz, $CDCl_3$, rt, *δ*/ppm): −2.86–−8.82 (m, 6B), −10.46–−14.15 (m, 8B). **^{13}C{^{1}H} NMR** (100 MHz, $CDCl_3$, rt, *δ*/ppm): 139.4, 135.9, 131.5, 127.8, 126.9, 81.7. **HRMS** (EI^+): m/z Calculated for $C_{12}H_{16}B_{10}$ ($[M]^+$) 270.2183, found 270.2181.

[6] Reaction of Li/Cu-1 with pyridine

Li/Cu-1 (32.3 mg, 0.050 mmol) and pyridine (1.0 equiv, 5.0 equiv, or none) were mixed in CD_2Cl_2 (0.7 mL), and the resultant mixture was transferred into a J. Young NMR tube. After standing at rt for 1 h, ^1H NMR data were collected (Figure S30). Upon increasing the amount of pyridine, signals of THF and some pyridine signals were obviously upfield-shifted, suggesting the existence of a coordination equilibrium.

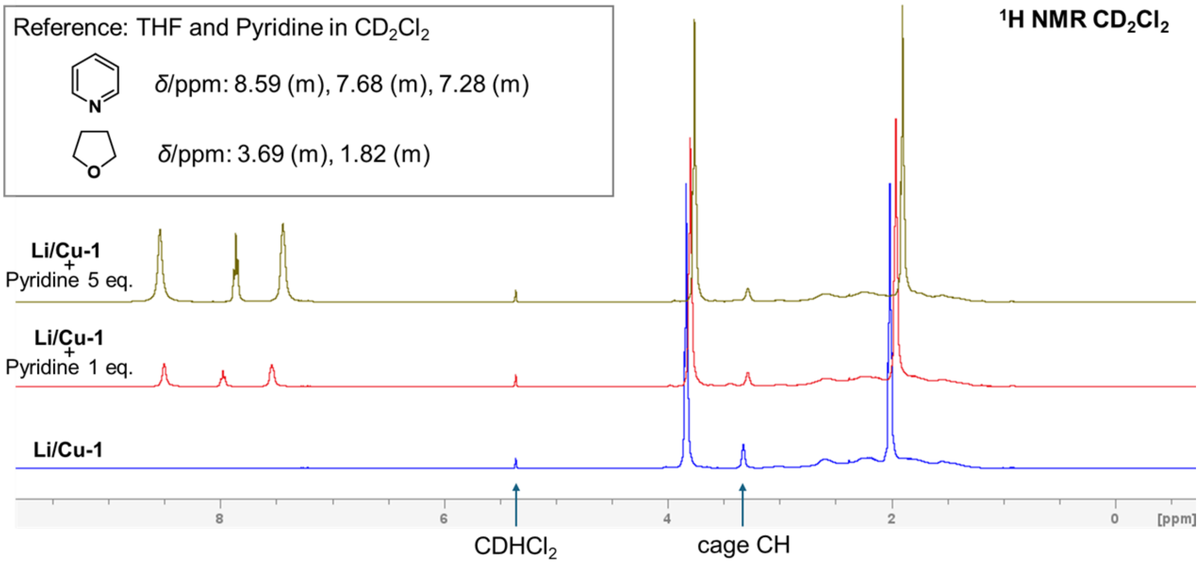

Figure S30. ^1H NMR of **Li/Cu-1** (bottom); after the addition of 1 equiv of pyridine (middle); after the addition of 5 equiv of pyridine (top).

[7] Results of *in situ* experiments

7-1. *In situ* generation of carboranyl copper species

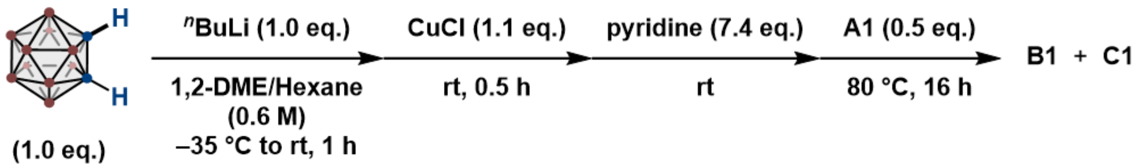

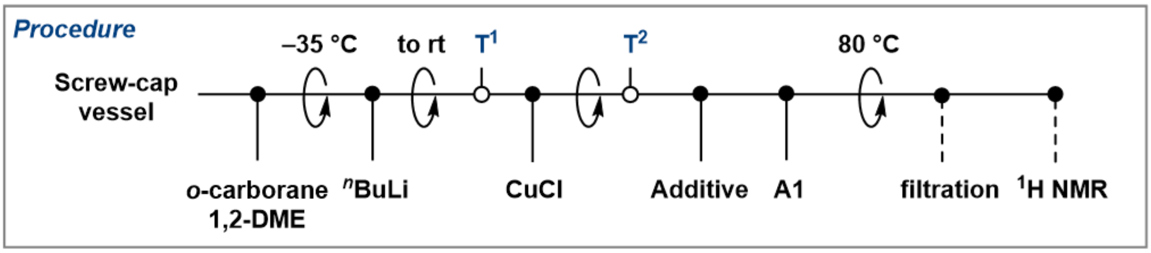

Run	Variation from the standard conditions	**B1** [%]	**C1** [%]	CBT [%]
1	None	1	18	10
2	CuI (1.1 eq.) instead of CuCl (1.1 eq.) was used	0	12	6
3	$[CuOTf]_2 \cdot C_6H_6$ (1.1 eq.) instead of CuCl (1.1 eq.) was used	0	4	2
4	^{i}PrMgCl (1.1 eq.) instead of ^{n}BuLi (1.1 eq.) was used	0	9	5
5	A premixed solution of CuCl (1.1 eq.) and pyridine (7.4 eq.) was added at T^1	1	16	9
6	Pyridine (3.8 eq.) was used	2	19	12
7	Pyridine (20.0 eq.) was used	0	12	6
8	DME/Hexane (1.0 M) instead of DME/Hexane (0.6 M)	1	15	9
9	DME/Hexane (0.6 M) was removed in vacuo and THF (0.6 M) was then added at T^1	2	23	14
10	DME/Hexane (0.6 M) was removed in vacuo and THF (0.6 M) was then added at T^2	2	19	12
11	DME/Hexane (0.6 M) was removed in vacuo and DMSO (0.6 M) was then added at T^1	1	27	15
12	DME/Hexane (0.6 M) was removed in vacuo and DMF (0.6 M) was then added at T^1	2	17	11
13	DME/Hexane (0.6 M) was removed in vacuo and 1,4-dioxane (0.6 M) was then added at T^1	1	19	11
14	DME/Hexane (0.6 M) was removed in vacuo and MeCN (0.6 M) was then added at T^1	0	0	0
15	DME/Hexane (0.6 M) was removed in vacuo and Toluene (0.6 M) was then added at T^1	2	27	16
16	DME/Hexane (0.6 M) was removed in vacuo and Toluene (0.6 M) was then added at T^1; pyridine (3.8 equiv) was used.	5	26	18
17	DME/Hexane (0.6 M) was removed in vacuo and Toluene (0.6 M) was then added at T^1; pyridine (2.0 equiv) was used.	5	29	20

Figure S31. Results of *in situ* experiments. T^1 and T^2 represent the points at which reagents were added.

General conditions (Figure S31, run 1): Based on the reported procedure[18], in a screw-cap reaction vessel equipped with a magnetic stirrer bar, a solution of *o*-carborane (86.5 mg, 0.600 mmol, 1.0 equiv, 1.0 M in DME) was slowly treated with ^{n}BuLi (0.397 mL, 0.599 mmol, 1.0 equiv, 1.51 M in hexane) at −35 °C, and the mixture was stirred for 1 h while gradually warming to rt. CuCl (65.9 mg, 0.666 mmol, 1.1 equiv) was then added, and the mixture was stirred for an additional 30 min, followed by the addition of pyridine (0.360 mL, 4.46 mmol, 7.4 equiv). Subsequently, **A1** (70.8 mg, 0.30 mmol, 0.5 equiv) was added, and the reaction mixture was stirred at 80 °C for 16 h. After cooling to rt, the resulting mixture was diluted with wet Et_2O. The mixture was then filtered through filter paper, and the volatiles were removed under reduced pressure. The yields of **B1** and **C1** were determined by NMR analysis using 1,3,5-trimethoxybenzene as an internal standard.

7-2. Ni-catalyzed carboranylation of **A1**

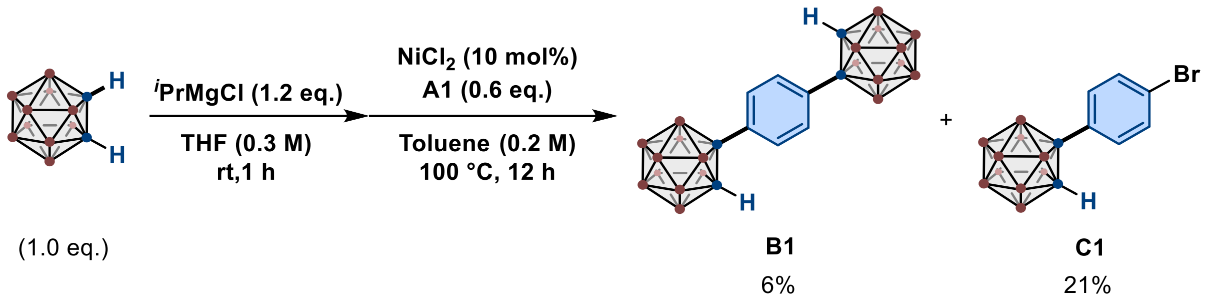

Figure S32. Ni-catalyzed carboranylation of **A1**

Based on the reported procedure[11], a solution of *o*-carborane (79.0 mg, 0.548 mmol, 1.0 equiv, 0.5 M in THF) was slowly treated with ^{i}PrMgCl (0.666 mL of a 1.0 M solution in THF, 0.666 mmol, 1.2 equiv) at rt, and the mixture was stirred for 1 h at rt. After removing all volatiles under reduced pressure, the residue was treated with toluene (2.78 mL), $NiCl_2$ (7.2 mg, 0.056 mmol, 10 mol%), and **A1** (78.3 mg, 0.308 mmol, 0.6 equiv). The mixture was then stirred at 100 °C for 12 h. After cooling to rt, the reaction mixture was diluted with wet Et_2O. The resulting mixture was filtered through filter paper, and the volatiles were removed under reduced pressure. The yields of **B1** and **C1** were determined by NMR analysis using 1,3,5-trimethoxybenzene as an internal standard.

[8] Recovery of *o*-carborane and isophthalonitrile

8-1. Reaction between **A1** and **Li/Cu-1** (Figure 2d)

The procedure follows the steps outlined in Section 5. After quenching the reaction synthesizing **B1**, the crude mixture was subjected to sublimation for 15 min at 100 °C under 0.2 mmHg. The remaining residue was purified by silica gel column chromatography, yielding **B1** (*vide supra*) and isophthalonitrile (60.4 mg, 0.471 mmol, 94%). The sublimated solid was collected with *n*-hexane, and the solvent was removed under reduced pressure to obtain *o*-carborane (64.8 mg, 0.449 mmol).

8-2. Reaction between **A17** and **Li/Cu-1** (Figure 4a)

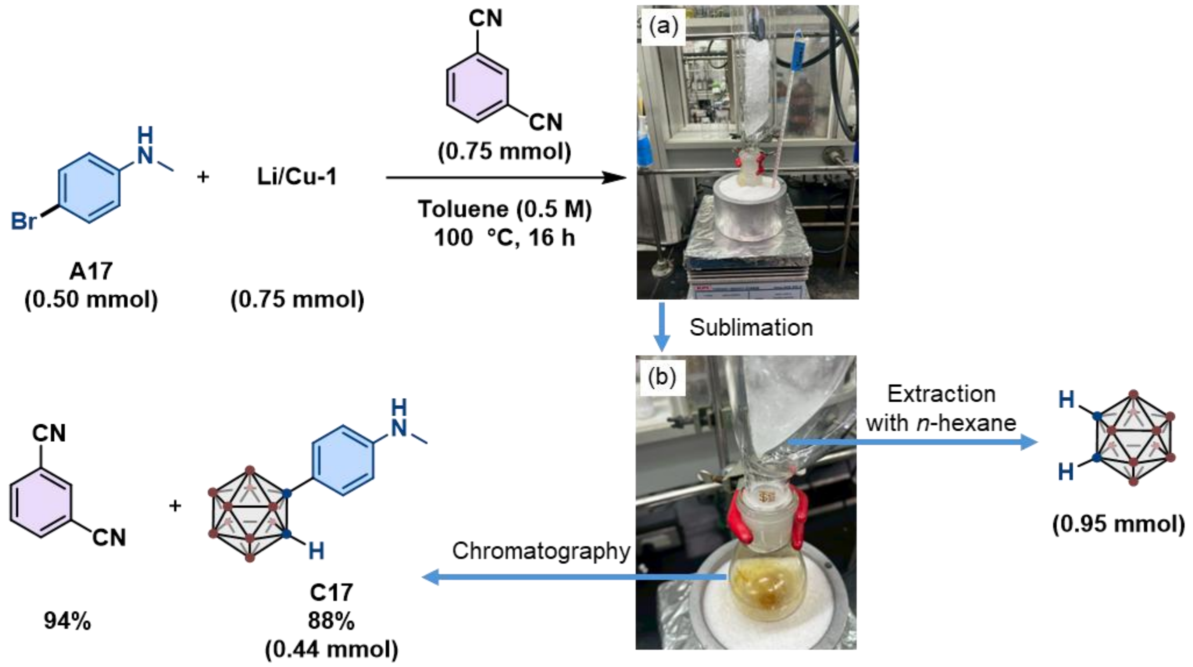

Figure S33. Recovery of *o*-carborane and isophthalonitrile from the reaction between **A17** and **Li/Cu-1**

The procedure follows the steps outlined in Section 5. After quenching the reaction synthesizing **C17** (Figure S33a), the crude mixture was subjected to sublimation for 15 min at 100 °C under 0.2 mmHg (Figure S33b). The remaining residue was purified by silica gel column chromatography, which yielded **C17** (*vide supra*) and isophthalonitrile (90.4 mg, 0.706 mmol, 94%). The sublimated solid was collected with *n*-hexane, and the solvent was removed under reduced pressure to afford *o*-carborane (136.3 mg, 0.945 mmol).

[9] Synthesis of 1,1′-bis(*o*-carborane)[19]

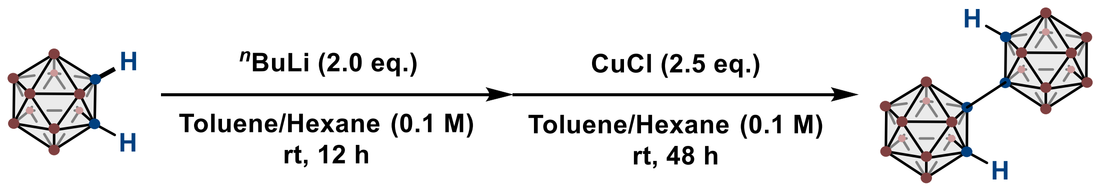

Figure S34. Synthesis of 1,1′-bis(*o*-carborane)

A solution of *o*-carborane (432 mg, 3.00 mmol, 0.15 M in Toluene) was slowly treated with ^{n}BuLi (3.90 mL of a 1.54 M solution in hexane, 6.01 mmol, 2.0 equiv) at rt and stirred for 12 h. CuCl (750 mg, 7.58 mmol, 2.5 equiv) was then added to the resultant solution at rt, and the mixture was stirred for 48 h. The reaction mixture was quenched with 3 M HCl, and the organic layer was separated. The aqueous phase was extracted with Et_2O, and the combined organic extracts were removed under reduced pressure. The crude product was purified by silica gel column chromatography (*n*-hexane) afforded as a 1,1′-bis(*o*-carborane) (147.2 mg, 0.514 mmol, 34%). **^{1}H NMR** (400 MHz, $CDCl_3$, rt, δ/ppm): 3.81 (s, 2H, cage C*H*), 3.60–1.23 (brm, B*H*).

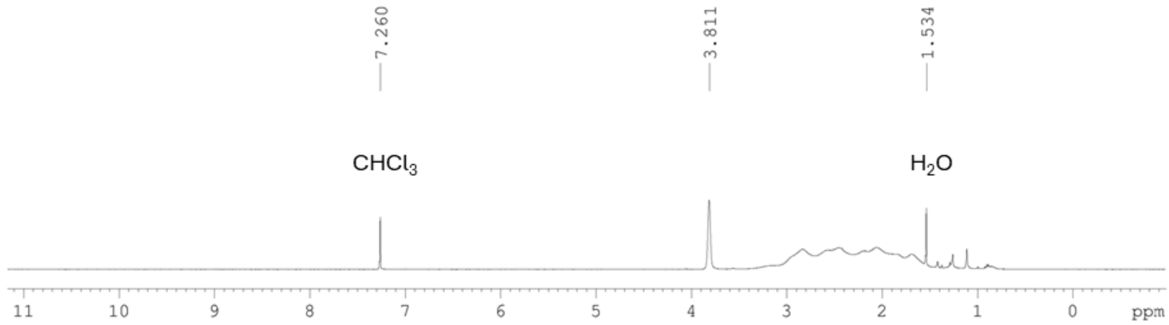

Figure S35. ^{1}H NMR (400 MHz, $CDCl_3$, rt) of the 1,1′-bis(*o*-carborane)

[10] Theoretical studies

10-1. Computational details

Density functional theory (DFT) calculations were performed using Gaussian 16 (Revision C.01) software[20]. Molecular structures were optimized and their single-point energies were calculated at the PBE0-D3 level with the Def2-TZVPD (Cu) and Def2-TZVP (others) basis sets in the gas phase [21,22]. Harmonic frequency calculations were performed at the same level of theory to verify that intermediates have no imaginary frequency, whereas transition states have only one imaginary frequency. The appropriateness of the connections between each reactant and product via a transition state was confirmed using intrinsic reaction coordinate (IRC) calculations[23]. The reported Gibbs free energies were calculated at 298.15 K. It should be noted that these calculations involve a certain margin of error. The Def2-TZVPD basis sets were obtained from the Basis Set Exchange website (https://www.basissetexchange.org/)[24].

10-2. Method Optimization

We carried out optimization of the gas-phase structure of **Li/Cu-1** at various theoretical levels shown in Figure S36 with the Def2-TZVPD (Cu) and Def2-TZVP (others) basis sets. By comparing the obtained structural parameters with the SC-XRD results, we decided to use the PBE0-D3 level in this work due to its high reproducibility.

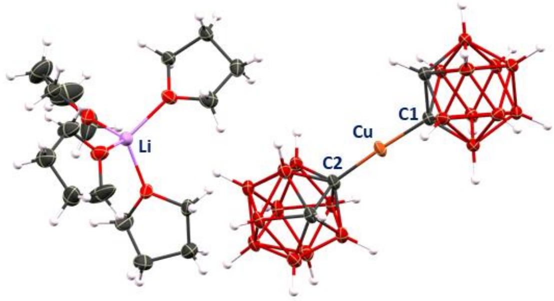

	XRD	B3LYP-D3	M06-D3	PBE0-D3	PBE0	wB97XD
Cu-C1	1.91	1.95	1.93	1.93	1.94	1.94
Cu-C2	1.91	1.95	1.94	1.93	1.94	1.94
Ave. Li-O	1.93	1.95	1.95	1.94	1.96	1.95
C1-Cu-C2	176.7	177.0	162.4	177.0	176.1	173.8

Figure S36. Optimization of theoretical levels. Bond lengths (Å) and angles (deg) are given.

To discuss the suitability of using PhCN as a mimicetic ligand for isophthalonitrile in the mechanistic studies, we compared the structures of **Li/Cu-2** and its mimic $[Li(thf)_2(CNPh)_2][Cu(o\text{-}carboran\text{-}1\text{-}yl)_2]$ under the optimized calculation conditions, as shown in Figure S37. To evaluate the long-range dispersion corrections, the ωB97XD level was also tested[25]. As a result, we confirmed the PBE0-D3 can adequately reproduce the monomeric structure of **Li/Cu-2** without the long-range dispersion corrections, although the distance between the Li and Cu centers found in the SC-XRD

analysis was not reproduced as it would be sensitive to crystal packing effects.

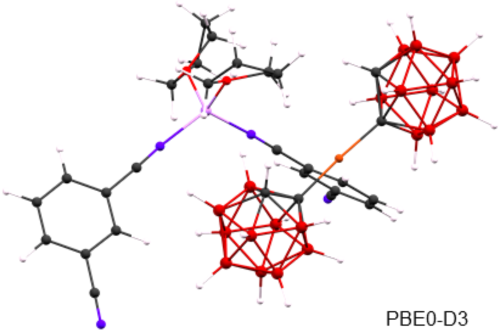

	XRD[1]	PBE0-D3	wB97XD
Cu-C1	1.91	1.93	1.94
Cu-C2	1.92	1.94	1.94
Ave. N-Li	2.03	2.02	2.07
Ave. Li-O	1.90	1.94	1.96
C1-Cu-C2	176.6	175.5	176.2
Ave. C-N-Li	172.0	174.1	171.0
Li---Cu	8.39	5.11	5.05

[1]Including toluene in the crystal lattice

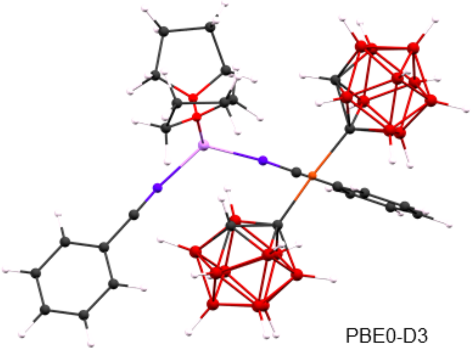

	PBE0-D3	wB97XD
Cu-C1	1.94	1.94
Cu-C2	1.94	1.94
Ave. N-Li	2.01	2.05
Ave. Li-O	1.96	1.99
C1-Cu-C2	176.5	177.2
Ave. C-N-Li	173.4	171.8
Li---Cu	5.13	5.02

Figure S37. Comparison of structural parameters between DFT-optimized **Li/Cu-2** and [Li(thf)$_2$(CNPh)$_2$][Cu(*o*-carboran-1-yl)$_2$]. Bond lengths (Å) and angles (deg) are given.

10-3. Mechanistic Studies

First, we examined how the number of coordinated THF molecules impacts the stability of **Li/Cu-1** (Figure S38). We found that dissociation of one THF molecule is possible, but dissociation of more than two THFs is unfavorable from the Li center. Additionally, forming the neutral Cu(*o*-carboran-1-yl) species is thermodynamically very unfavorable.

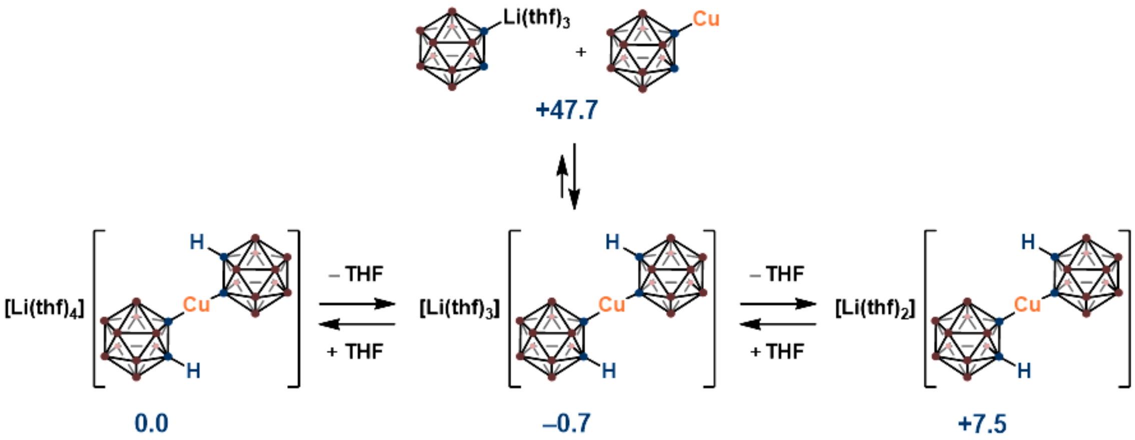

Figure S38. Influence of THF coordination on the Li center.

Then, we explored possible reaction mechanisms and found that the mechanism shown in Figure 5a is plausible after conducting the conformational searches for intermediates and transition states when required at the PBE0-D3/Def2-SVPD(Cu) and Def2-SVP(others) level of theory. The optimized structures of the involved compounds and transition states, along with their selected geometrical parameters, are shown in Figure S35.

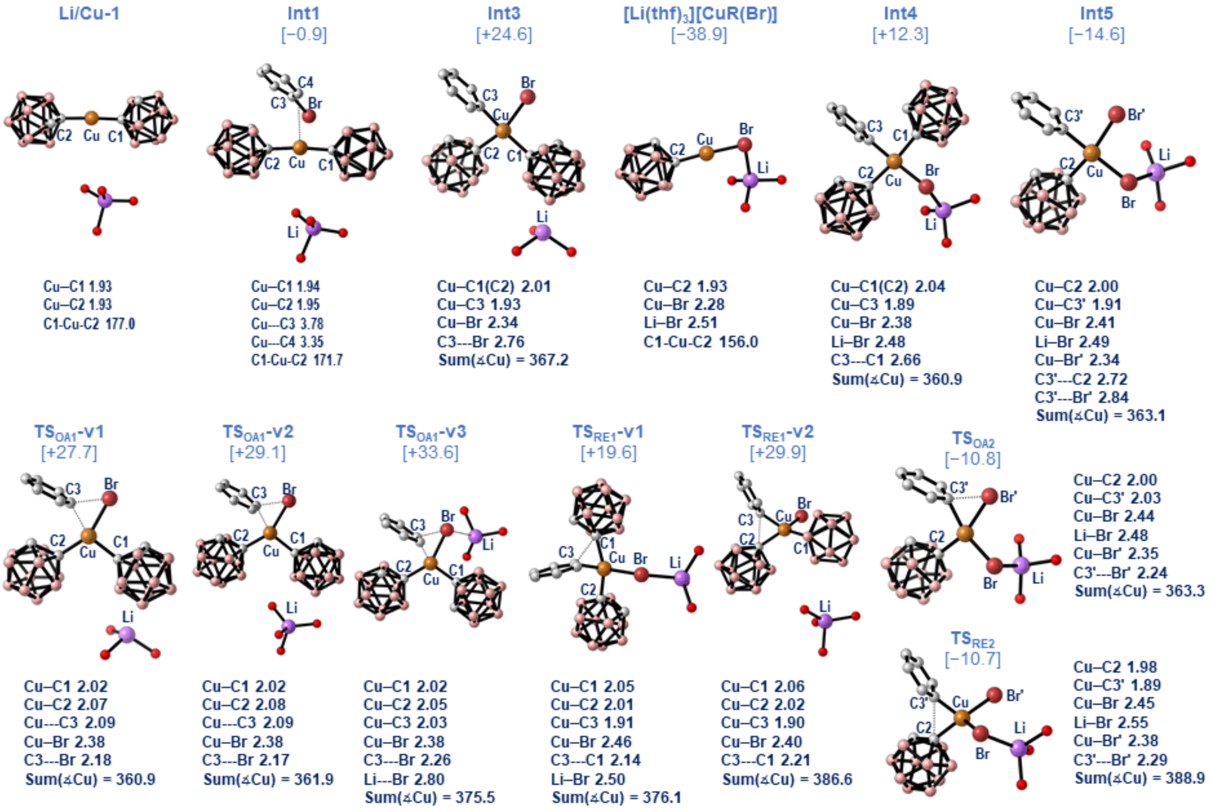

Figure S39. Optimized structures of the involved compounds and transition states, shown in Figure 5a. Their relative Gibbs free energies [kcal mol^{-1}] with respect to **Li/Cu-1** and selected geometrical parameters (bond lengths in Å and angles in degrees) are given.

Moreover, we explored the plausible oxidative addition paths from [Li(thf)$_3$(CNPh)][Cu(*o*-carboran-1-yl)$_2$] and [Li(thf)$_2$(CNPh)$_2$][Cu(*o*-carboran-1-yl)$_2$] to clarify the influence of PhCN molecules on the rate-determining steps (Figure 5c, Figure 5d, and Figure S40). The path proceeding via **TS$_{\mathbf{OA1}}$-v1** from **Int1** (i.e., $\Delta G_{\mathrm{v1}}^{\ddagger}$ = +29.8 kcal mol^{-1}) is also shown for comparison. It should be noted that the potential energy surface is different in the presence of PhCN and thus $\Delta G_{\mathrm{v1}}^{\ddagger}$ has changed from the values shown in Figure 5a. In the presence of one molecule of PhCN, oxidative addition occurs via **TS$_{\mathbf{OA1}}$-v5** with a nearly identical energy barrier ($\Delta G_{\mathrm{v5}}^{\ddagger}$ = +29.9 kcal mol^{-1}). On the other hand, the coordination of two molecules of PhCN, as seen in the SC-XRD structure of **Li/Cu-2**, significantly promotes the oxidative addition by stabilizing **TS$_{\mathbf{OA1}}$-v4** (+25.1 kcal mol^{-1}; $\Delta G_{\mathrm{v4}}^{\ddagger}$ = +27.2 kcal mol^{-1})

through non-covalent interactions between phenyl groups in PhCN and carboranyl B−H bonds. Indeed, such interactions are confirmed by the atoms in molecules (AIM) analysis (Figure S41).

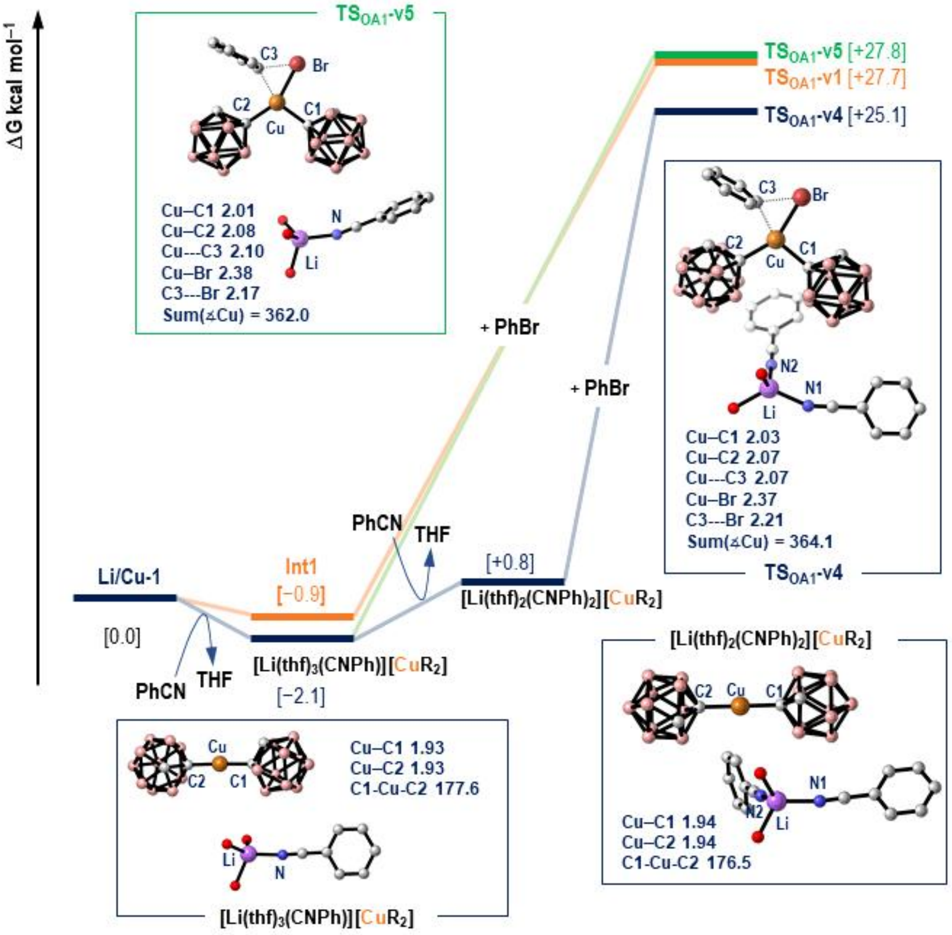

Figure S40. Oxidative addition processes in the presence of PhCN ligands. The relative Gibbs free energies [kcal mol^{-1}] with respect to [**Li/Cu-1** + 2 PhCN + PhBr] and selected geometrical parameters (bond lengths in Å and angles in degrees) are given.

10-4. Atoms in molecule (AIM) analysis

The analysis on the quantum theory of the atoms in molecules were carried out using the AIMAll program (Version 19.10.12)[26], in which wave functional files were prepared based on the SCF density at the PBE0-D3/Def2-TZVPD (Cu) and Def2-TZVP (others)/gas-phase level. In Table S1, details of the AIM analysis are given, wherein atom labels are identical to those found in the optimized DFT structures (Rho, electron density in $e\ r_{\mathrm{Bohr}}^{-3}$ oat BCPs; G and V, the kinetic and potential energy densities in $E_h\ r_{\mathrm{Bohr}}^{-3}$; δ(BCP) values show the average number of electrons shared at a BCP). In Figure S41, the AIM bond paths (white lines) are shown with the bond critical points (BCP; $e\ r_{\mathrm{Bohr}}^{-3}$; green dots), while C–H/B–H bonds that do not form NCIs and THF molecules are omitted for clarity. As demonstrated, several NCIs participate between the carborane and PhCN groups to stabilize **TS$_{\mathbf{OA1}}$-v4**.

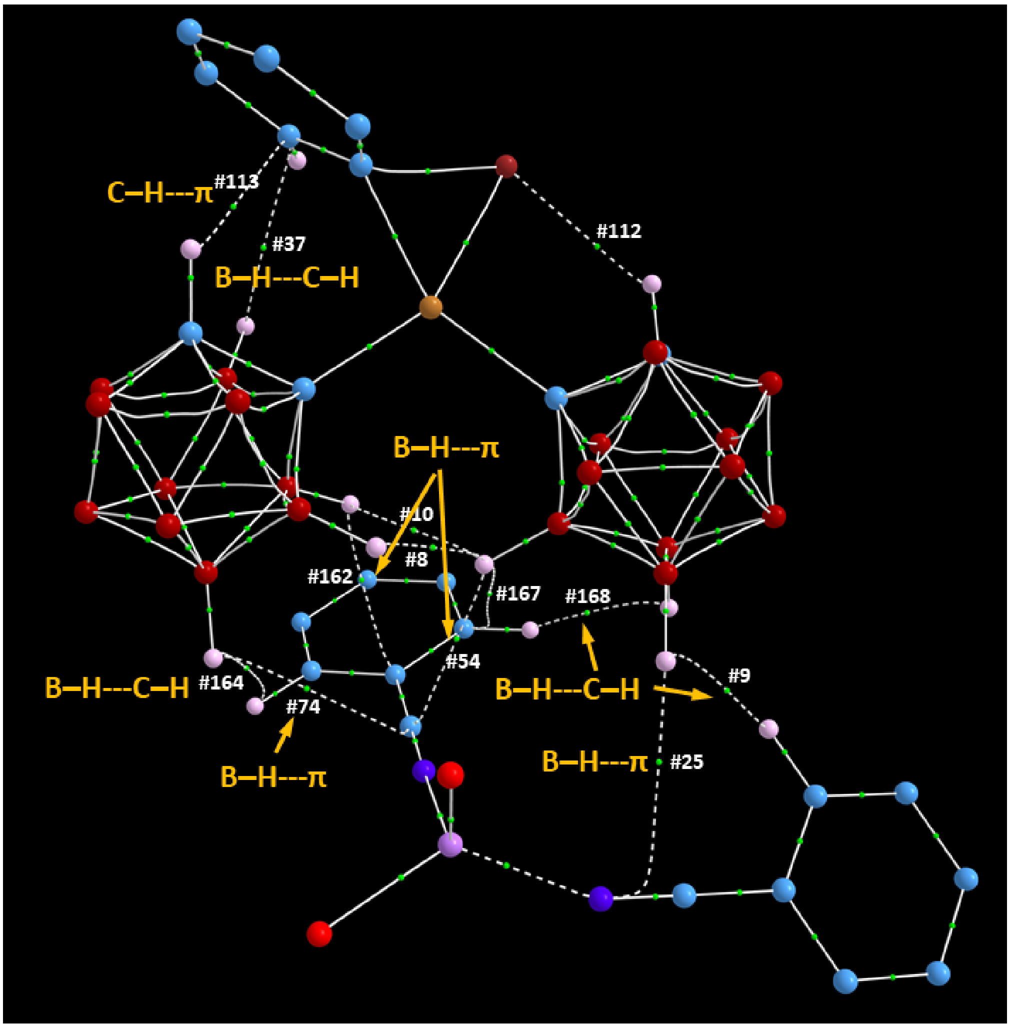

Figure S41. Selected bond paths, their names, and critical points for **TS$_{OA1}$-v4**.

Table S1. Results of the AIM analysis for **TS$_{OA1}$-v4**

BCP#	Atoms	Rho	DelSqRho	*G*	*V*	*δ*(BCP)
BCP1	C2 - H3	0.293919	-1.088791	0.03453	-0.341258	0.884006
BCP2	Cu1 - C4	0.095207	0.230127	0.093827	-0.130122	0.620709
BCP3	C2 - C4	0.177698	-0.158615	0.079643	-0.19894	0.98941
BCP4	B14 - B36	0.116378	-0.097534	0.039914	-0.104211	0.293687
BCP5	H35 - H70	0.009082	0.025417	0.005369	-0.004384	0.032562
BCP6	C4 - B5	0.127016	0.019615	0.119064	-0.233224	0.533071
BCP7	B14 - H15	0.185698	-0.328168	0.129022	-0.340087	0.646499
BCP8	H11 - H33	0.010623	0.030483	0.006521	-0.005422	0.040925
BCP9	H35 - H92	0.008738	0.026491	0.005465	-0.004306	0.025706
BCP10	H6 - H33	0.007536	0.021819	0.004593	-0.003731	0.028633
BCP11	B5 - H6	0.184648	-0.308352	0.131773	-0.340635	0.666525
BCP12	Cu1 - C7	0.087138	0.227172	0.086888	-0.116983	0.570129
BCP13	C7 - B58	0.127755	0.007484	0.116949	-0.232028	0.527899
BCP14	B32 - B58	0.119837	-0.11288	0.041268	-0.110755	0.324446
BCP15	B32 - B38	0.120929	-0.122553	0.038663	-0.107965	0.446041
BCP16	B27 - B42	0.121225	-0.126464	0.038608	-0.108833	0.459431
BCP17	C7 - B8	0.125473	-0.071948	0.089677	-0.197342	0.409757
BCP18	B8 - H9	0.185657	-0.331304	0.128148	-0.339121	0.651167

BCP19	C2 - B36	0.126533	0.096698	0.136562	-0.24895	0.519905
BCP20	C2 - B14	0.127392	0.049121	0.127404	-0.242527	0.409139
BCP21	H11 - H24	0.008791	0.024884	0.005306	-0.004391	0.034872
BCP22	C4 - B10	0.126205	0.015759	0.116888	-0.229836	0.53076
BCP23	B5 - B10	0.119513	-0.113264	0.040626	-0.109568	0.332265
BCP24	B14 - B20	0.122101	-0.129724	0.039138	-0.110708	0.452574
BCP25	H35 - N99	0.004131	0.01306	0.002551	-0.001837	0.019803
BCP26	C4 - B14	0.124725	-0.069243	0.089253	-0.195816	0.407167
BCP27	B5 - B14	0.121084	-0.121658	0.040953	-0.11232	0.333804
BCP28	B10 - H11	0.186396	-0.312579	0.133518	-0.345181	0.661891
BCP29	C2 - B12	0.127851	0.056048	0.129682	-0.245351	0.409592
BCP30	C4 - B12	0.124998	-0.06344	0.091962	-0.199783	0.408298
BCP31	H11 - H68	0.006333	0.019982	0.004119	-0.003243	0.016562
BCP32	B10 - B12	0.120946	-0.12107	0.040922	-0.112112	0.330111
BCP33	C2 - B18	0.12779	0.099325	0.138813	-0.252794	0.522908
BCP34	H24 - H68	0.007715	0.020927	0.004528	-0.003824	0.027475
BCP35	B12 - H13	0.185482	-0.330224	0.128143	-0.338842	0.648517
BCP36	B25 - B34	0.118644	-0.116223	0.037886	-0.104828	0.45599
BCP37	H15 - C48	0.007267	0.022117	0.004565	-0.0036	0.025332
BCP38	Cu1 - C47	0.08628	0.113071	0.059294	-0.090321	0.527354
BCP39	B16 - B25	0.121687	-0.127875	0.0388	-0.109568	0.449742
BCP40	C7 - B16	0.123815	-0.079569	0.082983	-0.185859	0.403943
BCP41	C7 - C59	0.176025	-0.150668	0.079734	-0.197136	0.993413
BCP42	B22 - B27	0.121622	-0.12868	0.038493	-0.109156	0.465498
BCP43	B16 - H17	0.185866	-0.331655	0.128467	-0.339847	0.65157
BCP44	B12 - B45	0.121663	-0.127252	0.03872	-0.109252	0.451171
BCP45	C66 - H68	0.281617	-0.976077	0.040142	-0.324304	0.908163
BCP46	B10 - B45	0.120588	-0.121377	0.038808	-0.10796	0.444386
BCP47	C69 - H71	0.285743	-1.016782	0.035783	-0.325762	0.919403
BCP48	C66 - C72	0.245532	-0.597481	0.062267	-0.273904	0.977735
BCP49	B12 - B18	0.115208	-0.091318	0.040241	-0.103311	0.281329
BCP50	B18 - H19	0.183503	-0.309538	0.129578	-0.33654	0.684036
BCP51	B5 - B20	0.119662	-0.118187	0.038456	-0.10646	0.436842
BCP52	B20 - B36	0.121306	-0.1287	0.038217	-0.108608	0.461473
BCP53	B5 - B29	0.120219	-0.121724	0.03809	-0.106611	0.455597
BCP54	H33 - C100	0.007332	0.022784	0.004774	-0.003853	0.019247
BCP55	B36 - H37	0.183425	-0.309384	0.129488	-0.336323	0.685257
BCP56	B18 - B45	0.122493	-0.134102	0.038177	-0.109879	0.471349
BCP57	B20 - H21	0.179475	-0.277403	0.130714	-0.330779	0.71065
BCP58	B42 - C59	0.127576	0.094556	0.137432	-0.251224	0.524534
BCP59	B8 - C59	0.127977	0.060471	0.130903	-0.246689	0.409666
BCP60	B16 - B22	0.115932	-0.095826	0.039783	-0.103522	0.289597
BCP61	B22 - C59	0.126501	0.094867	0.136103	-0.248489	0.520011
BCP62	B22 - H23	0.183743	-0.31213	0.129366	-0.336764	0.686231
BCP63	B22 - B25	0.121363	-0.129379	0.038218	-0.10878	0.462262
BCP64	B25 - H26	0.179813	-0.281417	0.130316	-0.330987	0.714849
BCP65	B25 - B27	0.117234	-0.10886	0.037884	-0.102984	0.437803
BCP66	C7 - B32	0.127867	0.015048	0.118972	-0.234181	0.523424
BCP67	B27 - H28	0.178228	-0.269301	0.130723	-0.328771	0.705406
BCP68	H11 - H65	0.007093	0.022964	0.004719	-0.003698	0.017681
BCP69	B10 - B29	0.121032	-0.124594	0.03855	-0.108249	0.457406
BCP70	B36 - B40	0.121426	-0.128554	0.038602	-0.109342	0.46266

BCP71	O61 - C63	0.247842	-0.513753	0.201924	-0.532287	0.847616
BCP72	Li60 - N112	0.026248	0.180317	0.037447	-0.029816	0.068052
BCP73	B20 - B29	0.118601	-0.117104	0.037647	-0.10457	0.459427
BCP74	H30 - C100	0.004123	0.013534	0.00263	-0.001876	0.00962
BCP75	Li60 - N99	0.023981	0.164079	0.033812	-0.026604	0.06342
BCP76	O61 - H85	0.006464	0.026431	0.005298	-0.003988	0.020055
BCP77	B20 - B40	0.117064	-0.108464	0.037866	-0.102847	0.434628
BCP78	B29 - H30	0.176183	-0.246109	0.132929	-0.327385	0.69331
BCP79	Cu1 - Br46	0.065985	0.175121	0.059775	-0.075769	0.604608
BCP80	B27 - B34	0.119714	-0.121882	0.037897	-0.106264	0.472589
BCP81	B8 - B32	0.120948	-0.119865	0.041676	-0.113318	0.32151
BCP82	C88 - C89	0.31392	-0.920263	0.104874	-0.439813	1.317054
BCP83	B38 - H39	0.176213	-0.247972	0.132357	-0.326707	0.699179
BCP84	B32 - H33	0.187063	-0.311159	0.134875	-0.34754	0.658728
BCP85	B32 - B34	0.121324	-0.124877	0.038575	-0.108369	0.453608
BCP86	B34 - H35	0.177071	-0.245426	0.134353	-0.330062	0.688665
BCP87	B18 - B36	0.117614	-0.102344	0.039633	-0.104852	0.316696
BCP88	B8 - B38	0.121726	-0.127198	0.038406	-0.108611	0.453439
BCP89	B27 - B38	0.117607	-0.109426	0.038219	-0.103795	0.441993
BCP90	B34 - B38	0.119106	-0.116396	0.038269	-0.105636	0.46073
BCP91	H44 - B45	0.17798	-0.25881	0.132606	-0.329914	0.699272
BCP92	C63 - H64	0.282526	-0.989637	0.037171	-0.321752	0.903946
BCP93	B18 - B40	0.120892	-0.125144	0.038751	-0.108789	0.454261
BCP94	B29 - B40	0.119506	-0.12158	0.037703	-0.105801	0.469298
BCP95	B40 - B45	0.117669	-0.110324	0.038146	-0.103874	0.438775
BCP96	C63 - H65	0.289956	-1.047651	0.034356	-0.330625	0.882588
BCP97	O62 - C84	0.253457	-0.542584	0.20636	-0.548367	0.861585
BCP98	H65 - H82	0.004598	0.016749	0.003294	-0.0024	0.007828
BCP99	B40 - H41	0.179955	-0.284419	0.129868	-0.330841	0.713957
BCP100	H44 - H82	0.00249	0.007888	0.001512	-0.001052	0.009936
BCP101	B8 - B42	0.114995	-0.090817	0.039997	-0.102699	0.279071
BCP102	B16 - C59	0.127757	0.055877	0.129508	-0.245046	0.411275
BCP103	B22 - B42	0.117415	-0.101283	0.039429	-0.104179	0.313531
BCP104	B38 - B42	0.12233	-0.133592	0.03807	-0.109538	0.473657
BCP105	B42 - H43	0.183291	-0.307893	0.129642	-0.336256	0.684962
BCP106	H44 - H65	0.008846	0.026506	0.005559	-0.004492	0.027211
BCP107	C69 - H70	0.289105	-1.038724	0.034311	-0.328304	0.887132
BCP108	Li60 - O61	0.028117	0.210481	0.043877	-0.035133	0.068273
BCP109	B29 - B45	0.118771	-0.115878	0.037981	-0.104932	0.458407
BCP110	H64 - H85	0.006099	0.023763	0.004847	-0.003754	0.009415
BCP111	Br46 - C47	0.089327	0.032016	0.037303	-0.066602	0.672665
BCP112	H31 - Br46	0.016036	0.046236	0.010342	-0.009124	0.072641
BCP113	H3 - C48	0.014493	0.045862	0.010017	-0.008568	0.027264
BCP114	B16 - B58	0.121446	-0.123879	0.040843	-0.112655	0.334868
BCP115	C48 - H51	0.287195	-1.030633	0.03716	-0.331978	0.928468
BCP116	C47 - C48	0.316731	-0.930463	0.108269	-0.449154	1.30147
BCP117	C47 - C49	0.316151	-0.923384	0.109005	-0.448856	1.313166
BCP118	C48 - C50	0.318458	-0.932139	0.11037	-0.453775	1.389948
BCP119	C49 - C52	0.31656	-0.924408	0.108826	-0.448754	1.380976
BCP120	C49 - H53	0.28544	-1.01842	0.037514	-0.329633	0.931917
BCP121	C50 - C54	0.317704	-0.93786	0.105889	-0.446243	1.369771

BCP122	C52 - C54	0.31927	-0.945111	0.107172	-0.450622	1.383798
BCP123	C50 - H55	0.285435	-1.017822	0.037386	-0.329228	0.949947
BCP124	C52 - H56	0.285508	-1.018516	0.037357	-0.329344	0.949626
BCP125	C54 - H57	0.285473	-1.01528	0.038352	-0.330525	0.955032
BCP126	B25 - B58	0.119947	-0.118892	0.038587	-0.106897	0.438006
BCP127	H24 - B58	0.182962	-0.291374	0.133097	-0.339037	0.661729
BCP128	B34 - B58	0.121147	-0.12439	0.038397	-0.107891	0.460499
BCP129	H31 - C59	0.290947	-1.065778	0.034633	-0.33571	0.898624
BCP130	H30 - H77	0.003967	0.011421	0.002355	-0.001855	0.01616
BCP131	Li60 - O62	0.025725	0.193955	0.040016	-0.031543	0.061744
BCP132	O61 - C69	0.245413	-0.501711	0.199701	-0.52483	0.844905
BCP133	C69 - C72	0.255575	-0.656814	0.061363	-0.28693	0.966076
BCP134	C63 - C66	0.256859	-0.662311	0.062437	-0.290451	0.963096
BCP135	C66 - H67	0.279273	-0.96394	0.041263	-0.32351	0.942447
BCP136	C72 - H73	0.279606	-0.967276	0.040593	-0.323006	0.940617
BCP137	C72 - H74	0.27771	-0.947251	0.042379	-0.321571	0.943588
BCP138	O62 - C75	0.252527	-0.515661	0.21707	-0.563056	0.85117
BCP139	C75 - H76	0.281961	-0.987436	0.036209	-0.319277	0.915545
BCP140	C75 - H77	0.289485	-1.047897	0.033849	-0.329671	0.895068
BCP141	C78 - H79	0.280106	-0.966774	0.041584	-0.324862	0.943497
BCP142	C78 - H80	0.279885	-0.962109	0.042003	-0.324533	0.946711
BCP143	C84 - H85	0.287338	-1.028439	0.035118	-0.327346	0.906433
BCP144	C75 - C81	0.25717	-0.663986	0.062008	-0.290014	0.963493
BCP145	C78 - C81	0.241445	-0.579951	0.060192	-0.265372	0.975993
BCP146	C81 - H82	0.281061	-0.971809	0.040543	-0.324038	0.924521
BCP147	C81 - H83	0.279402	-0.964503	0.041061	-0.323248	0.944485
BCP148	C78 - C84	0.245815	-0.609389	0.05749	-0.267328	0.953093
BCP149	C84 - H86	0.28435	-1.003284	0.036119	-0.323059	0.918737
BCP150	C87 - C88	0.288461	-0.853202	0.096675	-0.406651	1.082345
BCP151	C91 - C95	0.320457	-0.955684	0.106389	-0.451699	1.38893
BCP152	C88 - C90	0.313566	-0.917102	0.104104	-0.437484	1.315956
BCP153	C89 - C91	0.322955	-0.967128	0.108741	-0.459264	1.396931
BCP154	C91 - H96	0.287419	-1.03476	0.035812	-0.330315	0.941387
BCP155	C89 - H92	0.290651	-1.066605	0.033244	-0.333139	0.897131
BCP156	C90 - C93	0.322944	-0.964139	0.109828	-0.460692	1.409484
BCP157	C90 - H94	0.287112	-1.030955	0.036489	-0.330716	0.94509
BCP158	C93 - C95	0.319274	-0.949289	0.105654	-0.44863	1.381555
BCP159	C93 - H97	0.286395	-1.024474	0.037145	-0.330408	0.949849
BCP160	C95 - H98	0.286885	-1.029512	0.036374	-0.330125	0.949563
BCP161	C87 - N99	0.494742	-0.139599	0.939325	-1.913549	2.206744
BCP162	H6 - C101	0.007748	0.023737	0.004951	-0.003969	0.02711
BCP163	H77 - N112	0.007858	0.02851	0.0058	-0.004473	0.02982
BCP164	H30 - H107	0.005263	0.017043	0.003385	-0.00251	0.016155
BCP165	C100 - N112	0.49485	-0.134655	0.940673	-1.91501	2.177364
BCP166	C100 - C101	0.289863	-0.861741	0.098392	-0.41222	1.077433
BCP167	H33 - C102	0.005929	0.020134	0.004009	-0.002986	0.016029
BCP168	H39 - H105	0.007568	0.021594	0.004459	-0.003519	0.029423

BCP169	C101 - C102	0.314453	-0.922958	0.104874	-0.440488	1.305949
BCP170	C101 - C103	0.31453	-0.922961	0.104774	-0.440288	1.306225
BCP171	C102 - H105	0.289526	-1.053341	0.034609	-0.332554	0.913226
BCP172	C102 - C104	0.323374	-0.968174	0.109498	-0.461039	1.397543
BCP173	C103 - C106	0.323561	-0.968948	0.109785	-0.461807	1.396781
BCP174	C103 - H107	0.288465	-1.0425	0.035733	-0.33209	0.932751
BCP175	C104 - C108	0.320011	-0.953493	0.106055	-0.450484	1.382694
BCP176	C106 - C108	0.320046	-0.953636	0.106106	-0.450622	1.381465
BCP177	C104 - H109	0.286792	-1.028243	0.036738	-0.330536	0.947063
BCP178	C106 - H110	0.286933	-1.029221	0.036784	-0.330873	0.946871
BCP179	C108 - H111	0.286801	-1.028699	0.036515	-0.330204	0.950284

10-5. Comparison of gas-phase-optimized and SC-XRD structures of **B3** and **B4**

To confirm that distortions found in **B3** and **B4** by SC-XRD analyses are not caused by crystal packing effects, we optimized their isolated gas-phase structures at the PBE0-D3/Def2-SVP. As shown in Figure S42, the distorted structures are reproduced by DFT, showing that the repulsions between carboranyl C–H/B–H and aromatic C–H bonds are responsible for these distortions.

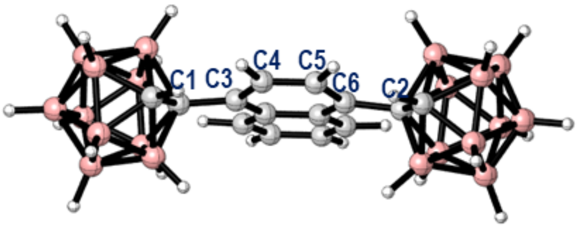

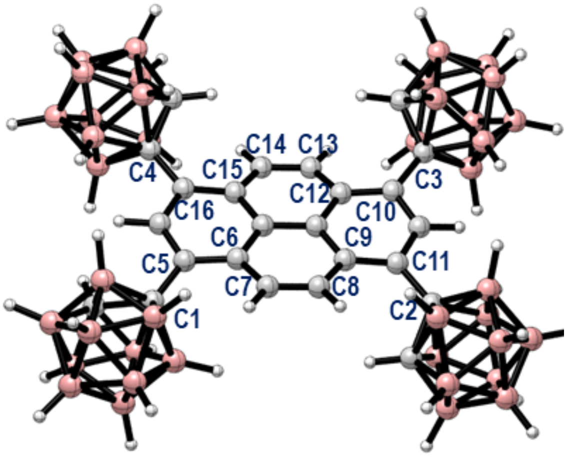

	XRD	PBE0-D3
C1-C3	1.52	1.52
C2-C6	1.52	1.52
C4-C5	1.40	1.40
C1-C3-C4-C5	168.0	173.8
C2-C6-C5-C4	−165.4	-173.7

	XRD	PBE0-D3
C1-C5-C6-C7	−21.4	−15.7
C2-C11-C9-C8	19.6	17.1
C3-C10-C12-C13	18.9	16.0
C4-C16-C15-C14	−17.7	−15.0

Figure S42. Selected geometrical parameters (bond lengths in Å and angles in degrees) for **B3** and **B4**

[11] Reference and Notes

1. Dolomanov, O. V.; Bourhis, L. J.; Gildea, R. J.; Howard, J. A. K.; Puschmann, H. OLEX2: a complete structure solution, refinement and analysis program. *J. Appl. Crystallogr.* **2009**, *42*, 339–341.
2. Sheldrick, G. M. *SHELXT* – Integrated space-group and crystal-structure determination. *Acta Crystallogr. A* **2015**, *71*, 3–8.
3. Sheldrick, G. M. Crystal structure refinement with *SHELXL*. *Acta Crystallogr. C* **2015**, *71*, 3–8.
4. Fox, M. A.; Hughes, A. K. Cage C–H···X interactions in solid-state structures of icosahedral carboranes. *Coord. Chem. Rev.* **2004**, *248*, 457–476.
5. Songkram, C.; Ohta, K.; Yamaguchi, K.; Pichierri, F.; Endo, Y. Conformational Control of Benzyl-*o*-carboranylbenzene Derivatives and Molecular Encapsulation of Acetone in the Dynamically Formed Space of 1,3,5-Tris(2-Benzyl-*o*-Carboran-1-yl)benzene. *Inorg. Chem.* **2010**, *49*, 11174–11183.
6. Jiang, W.; Knobler, C. B.; Hawthorne, M. F. Synthesis and Structural Characterization of Bis- and Tris(*closo*-1,2-$C_2B_{10}H_{11}$-1-yl)-Substituted Biphenyl and Benzene. *Inorg. Chem.* **1996**, *35*, 3056–3058.
7. Li, Y.; Zhao, P.; Jia, C.; Sun, N.; Ma, Y.-N.; Duan, Z. Reaction of Carboranyl Lithium and 2,3-Dichloroquinoxaline to Synthesize Carborane-Fused *N*-Heteroaromatic. *Organometallics* **2024**, *43*, 1077–1084.
8. Wyzlic, I. M.; Tjarks, W.; Soloway, A. H.; Perkins, D. J.; Burgos, M.; O'Reilly, K. P. Synthesis of Carboranyl Amino Acids, Hydantoins, and Barbiturates. *Inorg. Chem.* **1996**, *35*, 4541–4547.
9. Murphy, D. M.; Mingos, D. M. P.; Forward, J. M. Synthesis of icosahedral carboranes for second-harmonic generation. Part 1. *J. Mater. Chem.* **1993**, *3*, 67–76.
10. Causey, P. W.; Besanger, T. R.; Valliant, J. F. Synthesis and Screening of Mono- and Di-Aryl Technetium and Rhenium Metallocarboranes. A New Class of Probes for the Estrogen Receptor. *J. Med. Chem.* **2008**, *51*, 2833–2844.
11. Tang, C.; Xie, Z. Nickel-Catalyzed Cross-Coupling Reactions of *o*-Carboranyl with Aryl Iodides: Facile Synthesis of 1-Aryl-*o*-Carboranes and 1,2-Diaryl-*o*-Carboranes. *Angew. Chem. Int. Ed.* **2015**, *54*, 7662–7665.
12. Lee, S. H.; Kwon, Y. B.; Yoon, C. M. Synthesis of 5-*ortho*-Carboranylsalicylaldehyde and an Indolinospirobenzopyran. *Synth. Commun.* **2009**, *39*, 4069–4078.
13. Lu, J. Y.; Wan, H.; Zhang, J.; Wang, Z.; Li, Y.; Du, Y.; Li, C.; Liu, Z. T.; Liu, Z. W.; Lu, J. Palladium-Catalyzed Direct Cross-Coupling of Carboranyllithium with (Hetero)Aryl Halides. *Chem. Eur. J.* **2016**, *22*, 17542–17546.
14. Fabre, B.; Hao, E.; LeJeune, Z. M.; Amuhaya, E. K.; Barrière, F.; Garno, J. C.; Vicente, M. G. H. Polythiophenes Containing In-Chain Cobaltabisdicarbollide Centers. *ACS Appl. Mater. Interfaces* **2010**, *2*, 691–702.
15. Morisaki, Y.; Tominaga, M.; Ochiai, T.; Chujo, Y. *o*-Carborane-based Biphenyl and *p*-Terphenyl

Derivatives. *Chem. Asian J.* **2014**, *9*, 1247–1251.

16. Morisaki, Y.; Tominaga, M.; Chujo, Y. Synthesis and Properties of Thiophene-Fused Benzocarborane. *Chem. Eur. J.* **2012**, *18*, 11251–11257.
17. Jia, C.; Wang, L.; Ma, Y. N.; Duan, Z. Synthesis and Electronic Properties of Arene-Fused *o*-Carborane. *Organometallics* **2025**, *44*, 938–943.
18. Coult, R.; Fox, M. A.; Gill, W. R.; Herbertson, P. L.; MacBride, J. A. H.; Wade, K. C-arylation and C-heteroarylation of icosahedral carboranes via their copper(I) derivatives. *J. Organomet. Chem.* **1993**, *462*, 19–29.
19. Ren, S.; Xie, Z. A Facile and Practical Synthetic Route to 1,1′-Bis(*o*-carborane). *Organometallics* **2008**, *27*, 5167–5168.
20. Frisch, M. J.; Trucks, G. W.; Schlegel, H. B.; Scuseria, G. E.; Robb, M. A.; Cheeseman, J. R.; Scalmani, G.; Barone, V.; Petersson, G. A.; Nakatsuji, H.; Li, X.; Caricato, M.; Marenich, A. V.; Bloino, J.; Janesko, B. G.; Gomperts, R.; Mennucci, B.; Hratchian, H. P.; Ortiz, J. V.; Izmaylov, A. F.; Sonnenberg, J. L.; Williams-Young, D.; Ding, F.; Lipparini, F.; Egidi, F.; Goings, J.; Peng, B.; Petrone, A.; Henderson, T.; Ranasinghe, D.; Zakrzewski, V. G.; Gao, J.; Rega, N.; Zheng, G.; Liang, W.; Hada, M.; Ehara, M.; Toyota, K.; Fukuda, R.; Hasegawa, J.; Ishida, M.; Nakajima, T.; Honda, Y.; Kitao, O.; Nakai, H.; Vreven, T.; Throssell, K.; Montgomery, J. A., Jr.; Peralta, J. E.; Ogliaro, F.; Bearpark, M. J.; Heyd, J. J.; Brothers, E. N.; Kudin, K. N.; Staroverov, V. N.; Keith, T. A.; Kobayashi, R.; Normand, J.; Raghavachari, K.; Rendell, A. P.; Burant, J. C.; Iyengar, S. S.; Tomasi, J.; Cossi, M.; Millam, J. M.; Klene, M.; Adamo, C.; Cammi, R.; Ochterski, J. W.; Martin, R. L.; Morokuma, K.; Farkas, O.; Foresman, J. B.; Fox, D. J. Gaussian, Inc., Wallingford CT, 2016.
21. Adamo, C.; Barone, V. Toward reliable density functional methods without adjustable parameters: The PBE0 model. *J. Chem. Phys.* **1999**, *110*, 6158–6170.
22. Grimme, S.; Antony, J.; Ehrlich, S.; Krieg, H. **A** consistent and accurate ab initio parametrization of density functional dispersion correction (DFT-D) for the 94 elements H-Pu *J. Chem. Phys.* **2010**, *132*, 154104.
23. Fukui, K. The path of chemical reactions – the IRC approach. *Acc. Chem. Res.* **1981**, *14*, 363–368.
24. Pritchard, B. P.; Altarawy, D.; Didier, B.; Gibson, T. D.; Windus, T. L. New Basis Set Exchange: An Open, Up-to-Date Resource for the Molecular Sciences Community. *J. Chem. Inf. Model.* **2019**, *59*, 4814–4820.
25. Chai, J. D.; Head-Gordon, M. Long-range corrected hybrid density functionals with damped atom-atom dispersion corrections. *Phys. Chem. Chem. Phys.* **2008**, *10*, 6615–6620.
26. Keith, T. A. AIMAll (Version 19.10.12, Professional): Computational Chemistry Using the Quantum Theory of Atoms in Molecules (QTAIM). TK Gristmill Software, Overland Park, KS, 2017. Available at: http://aim.tkgristmill.com/.

[12] NMR spectra

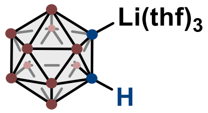

^{1}H NMR, (400 MHz, C_6D_6)

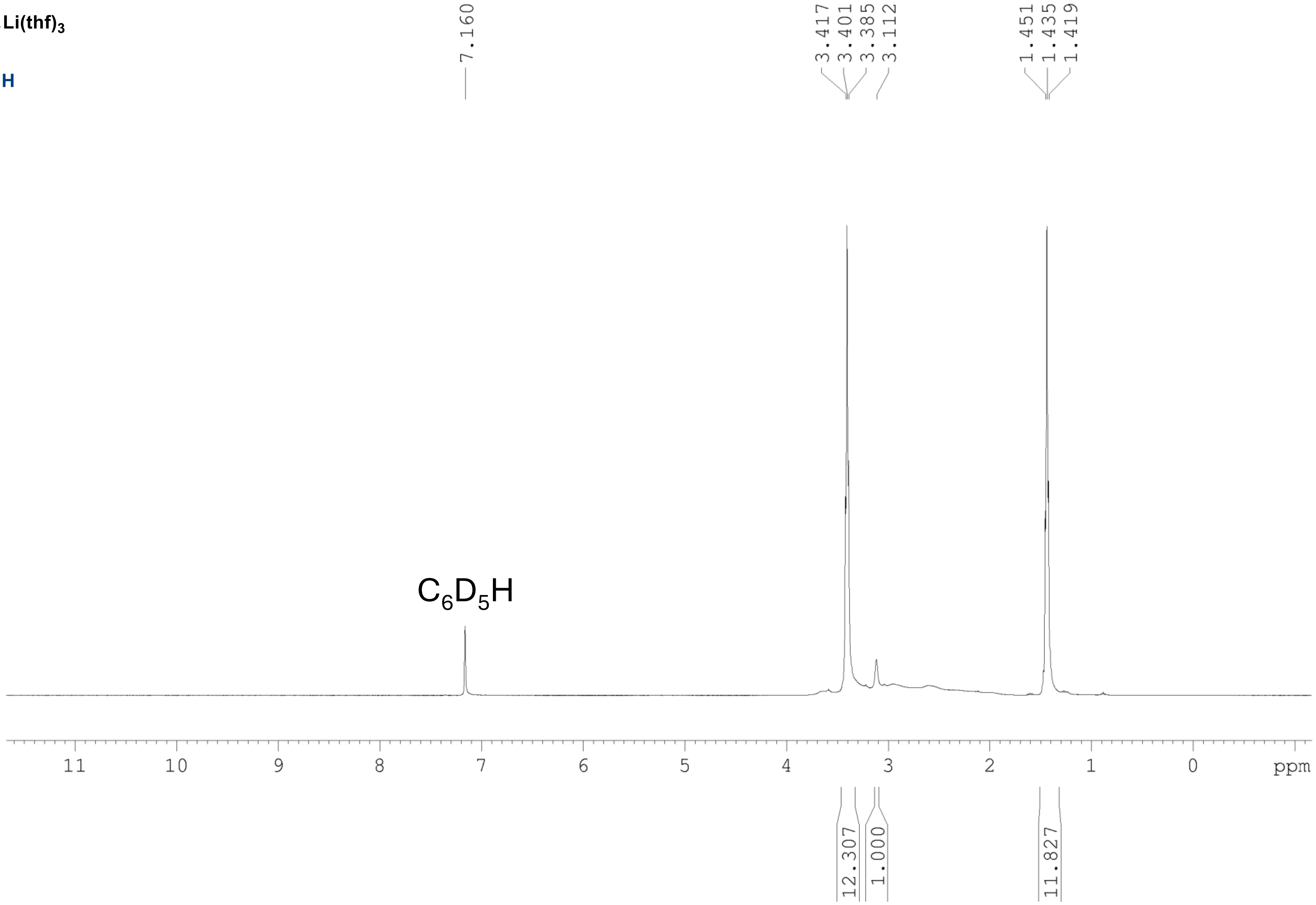

^{11}B NMR, (128 MHz, C_6D_6)

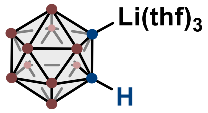

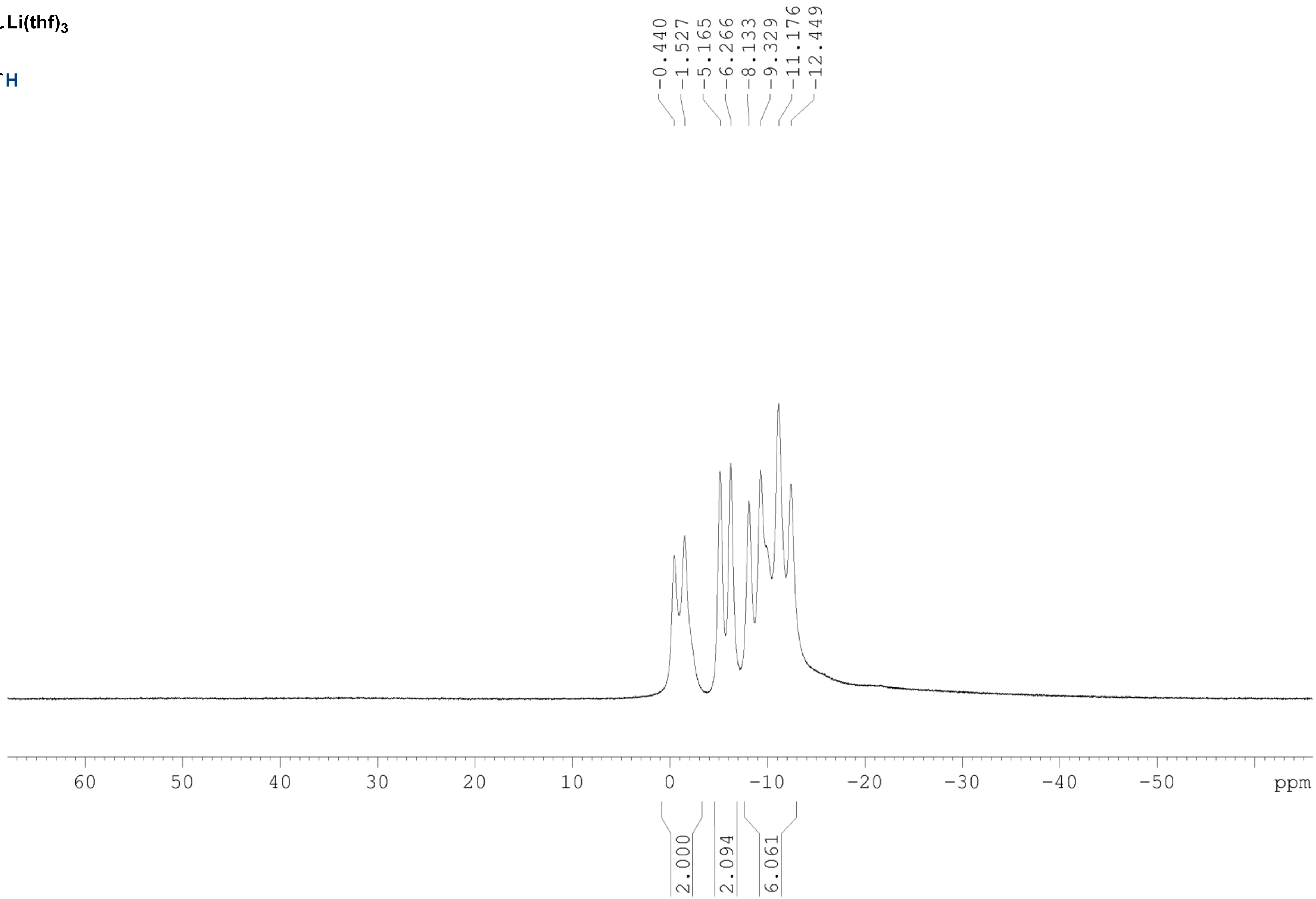

^{13}C NMR, (100 MHz, C_6D_6)

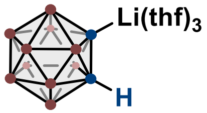

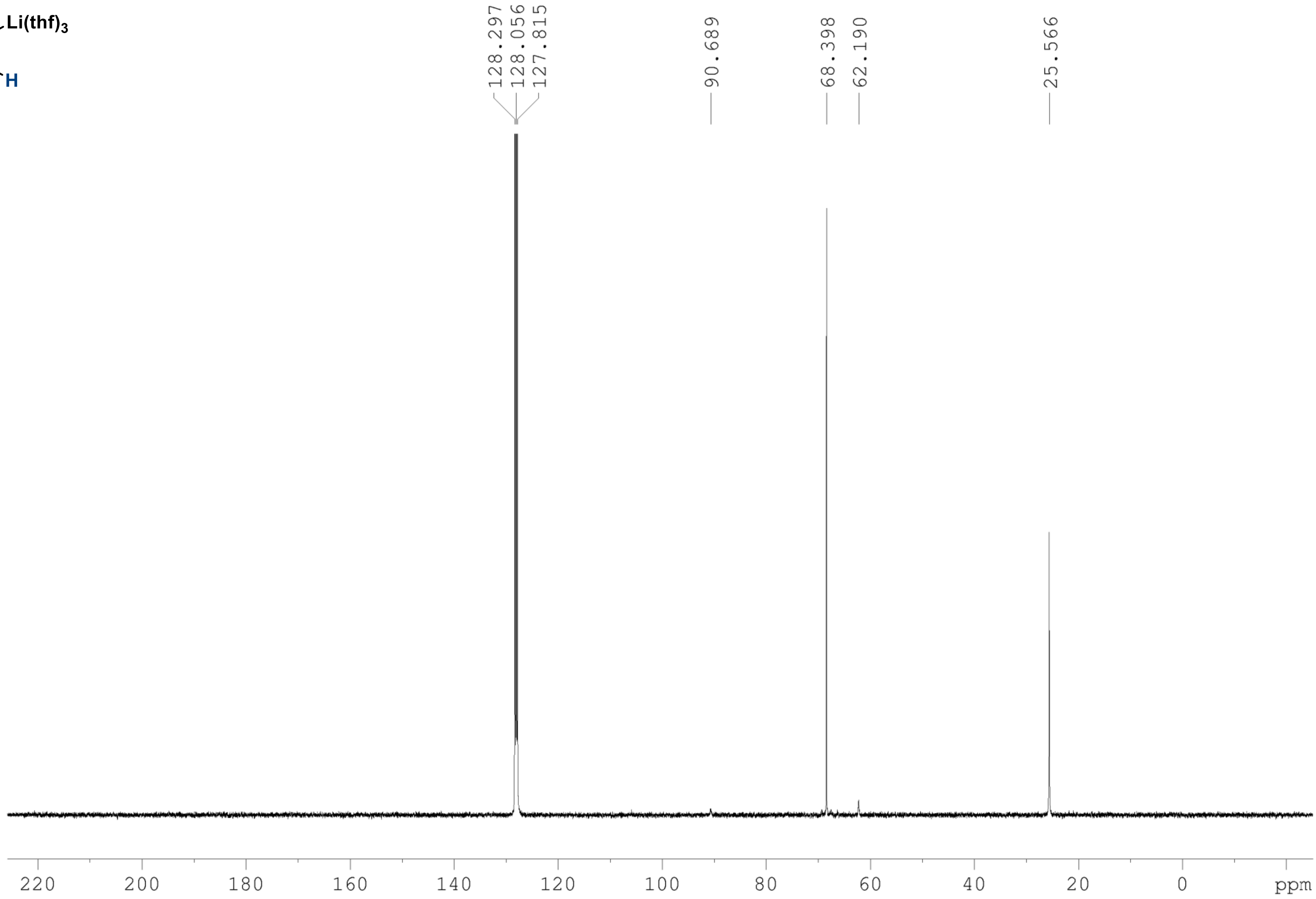

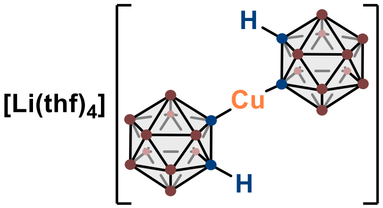

^{1}H NMR, (400 MHz, CD_2Cl_2)

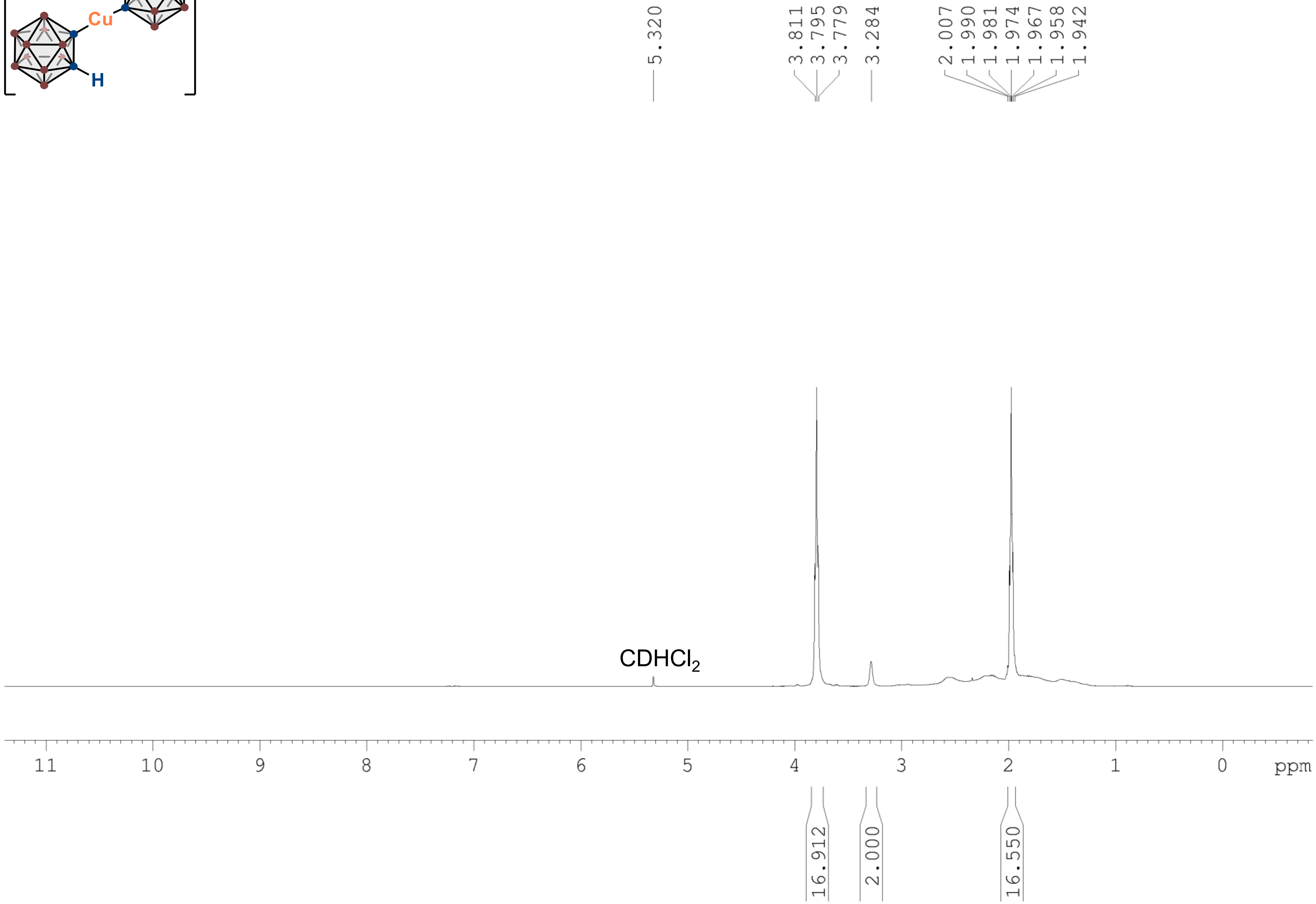

[Li(thf)$_4$]

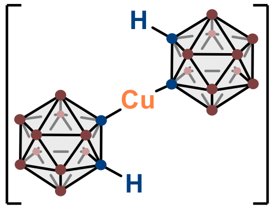

^{11}B NMR, (128 MHz, CD_2Cl_2)

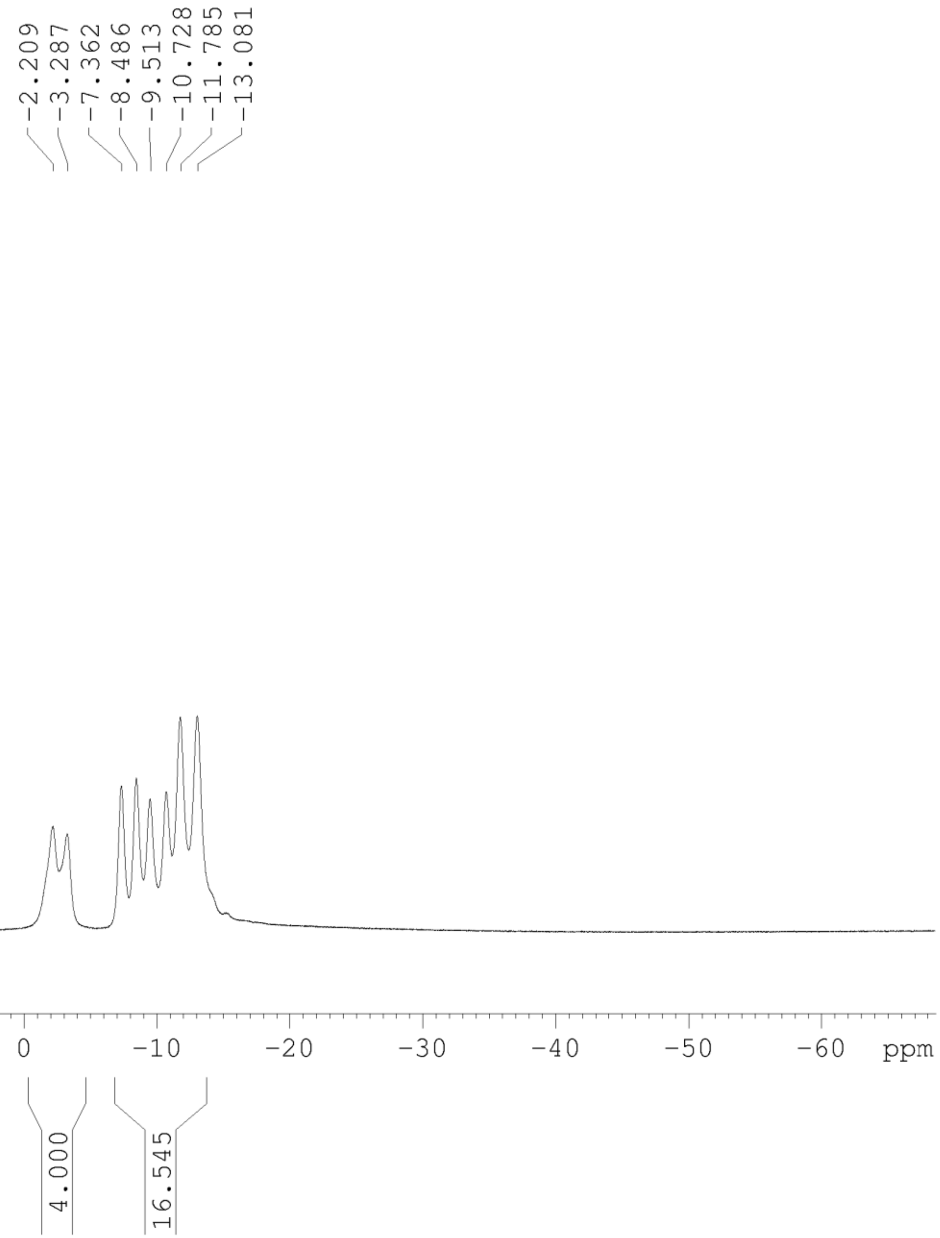

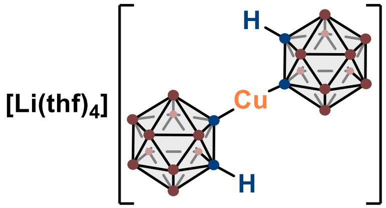

^{13}C NMR, (100 MHz, CD_2Cl_2)

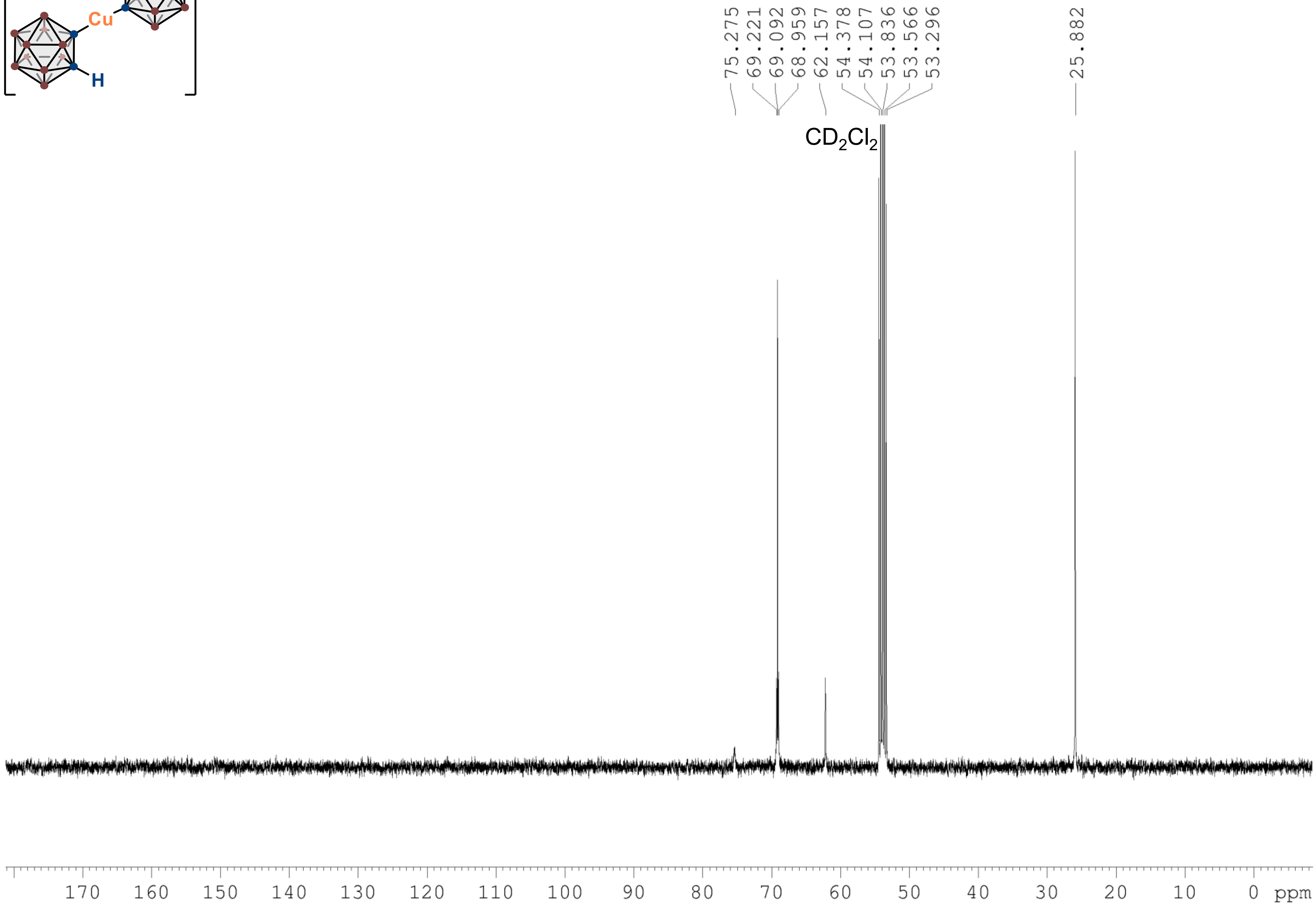

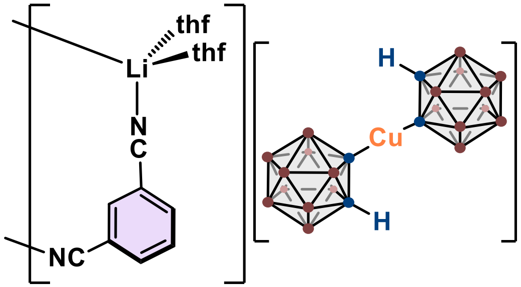

^1H NMR, (400 MHz, CD_2Cl_2)

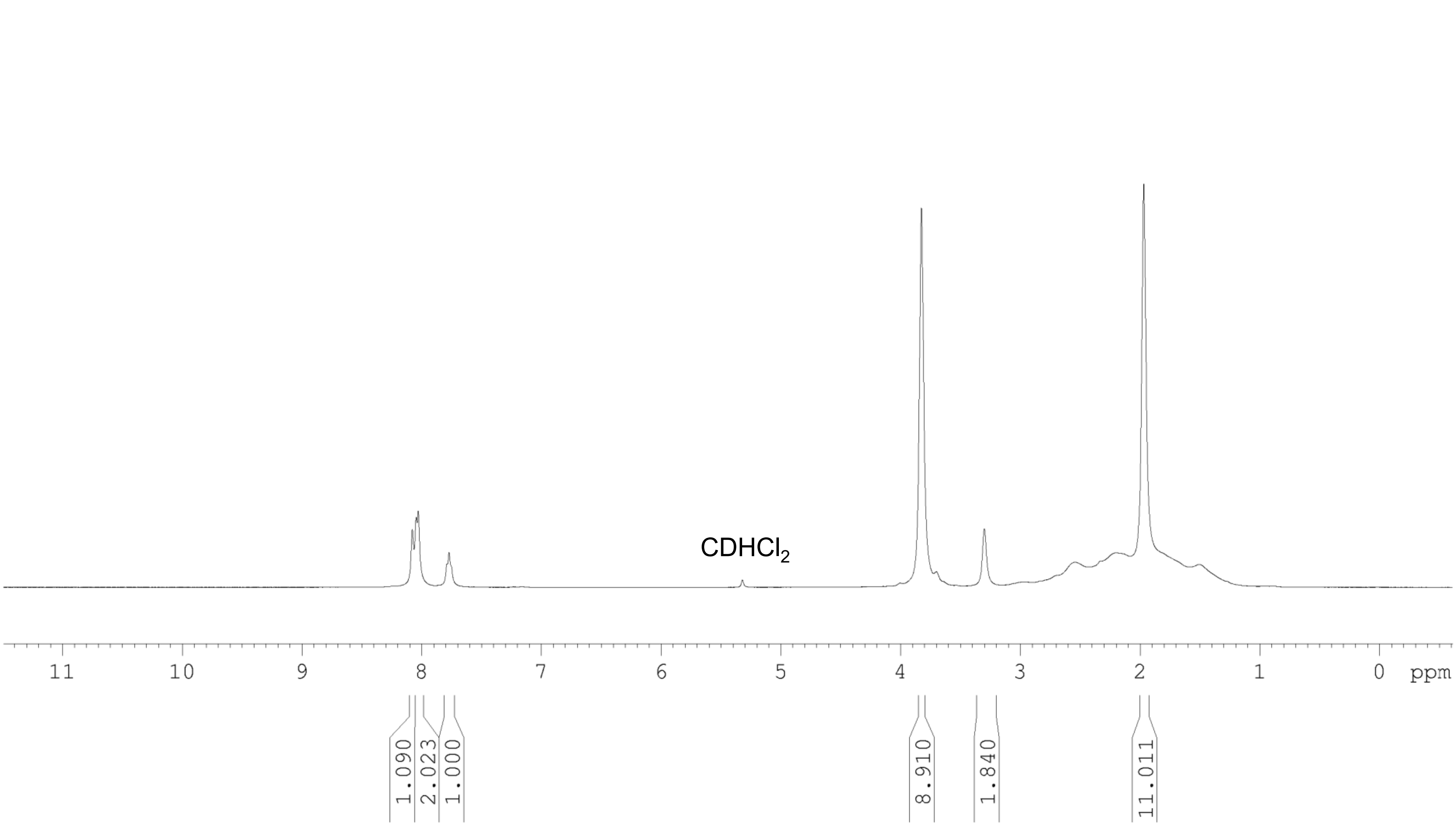

^{11}B NMR, (128 MHz, CD_2Cl_2)

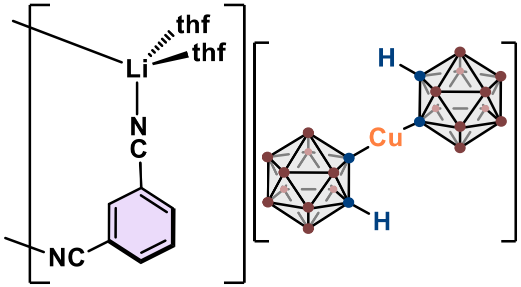

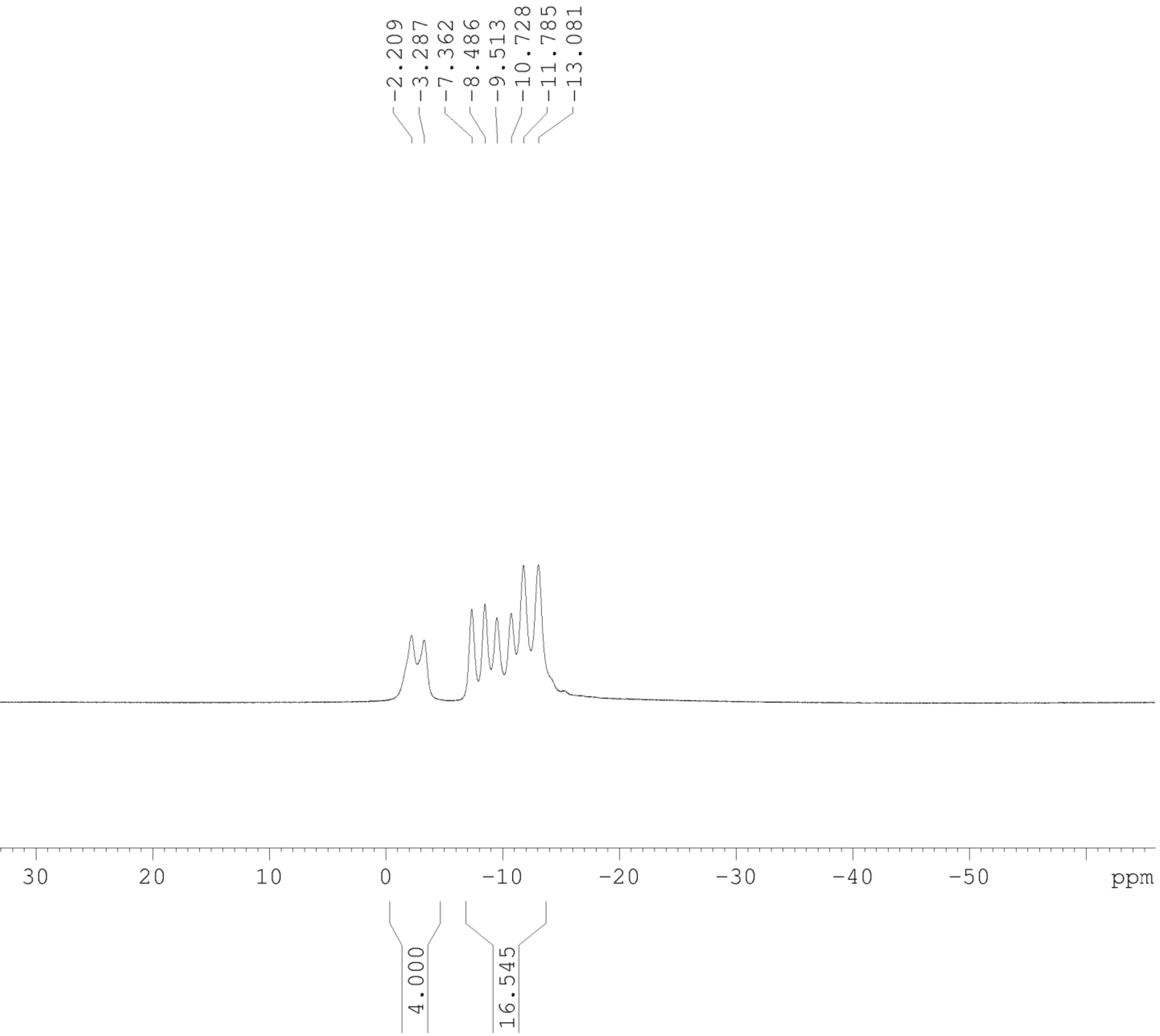

^{13}C NMR, (100 MHz, CD_2Cl_2)

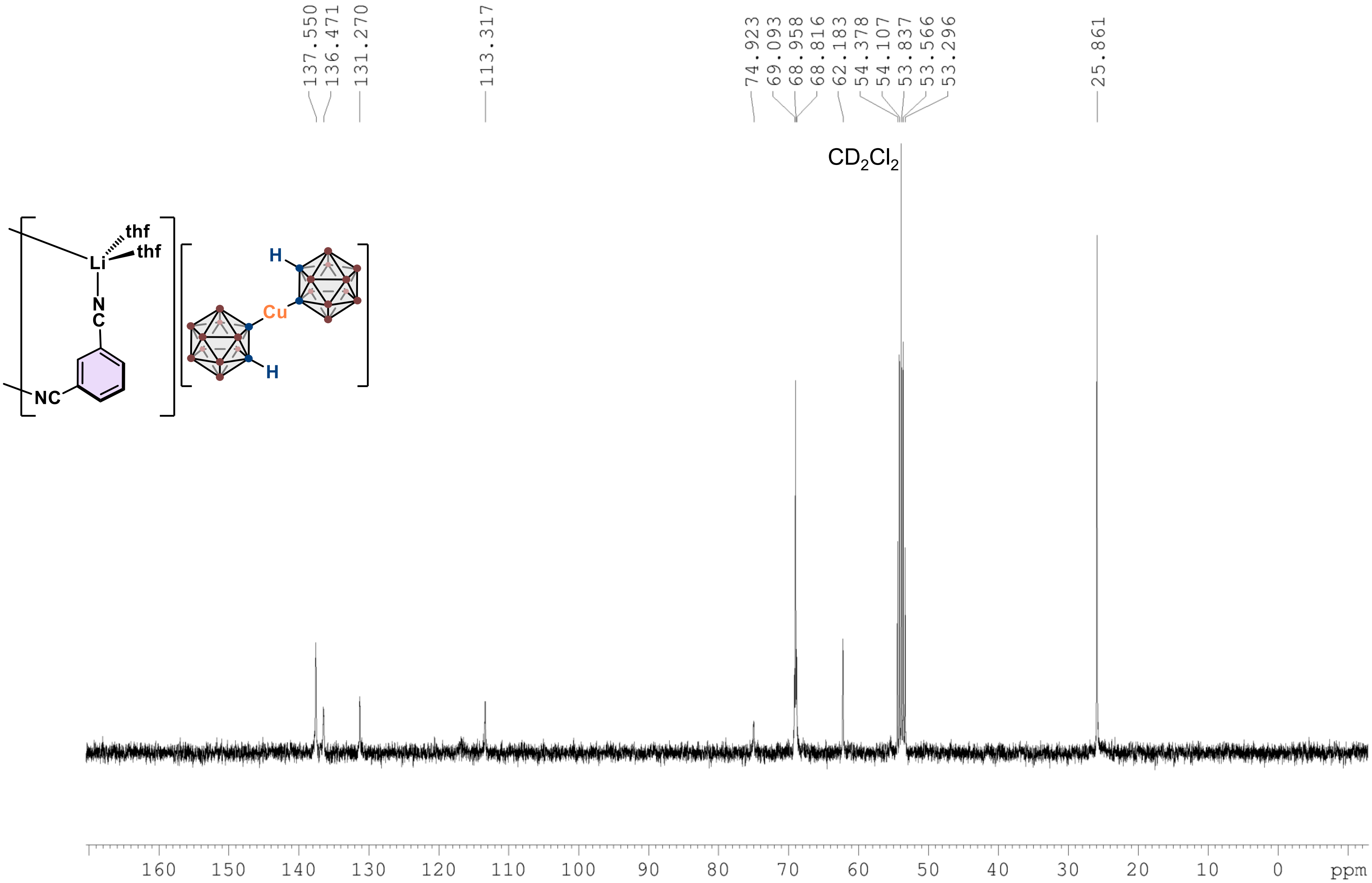

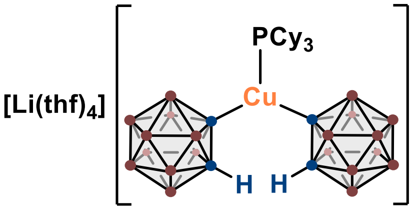

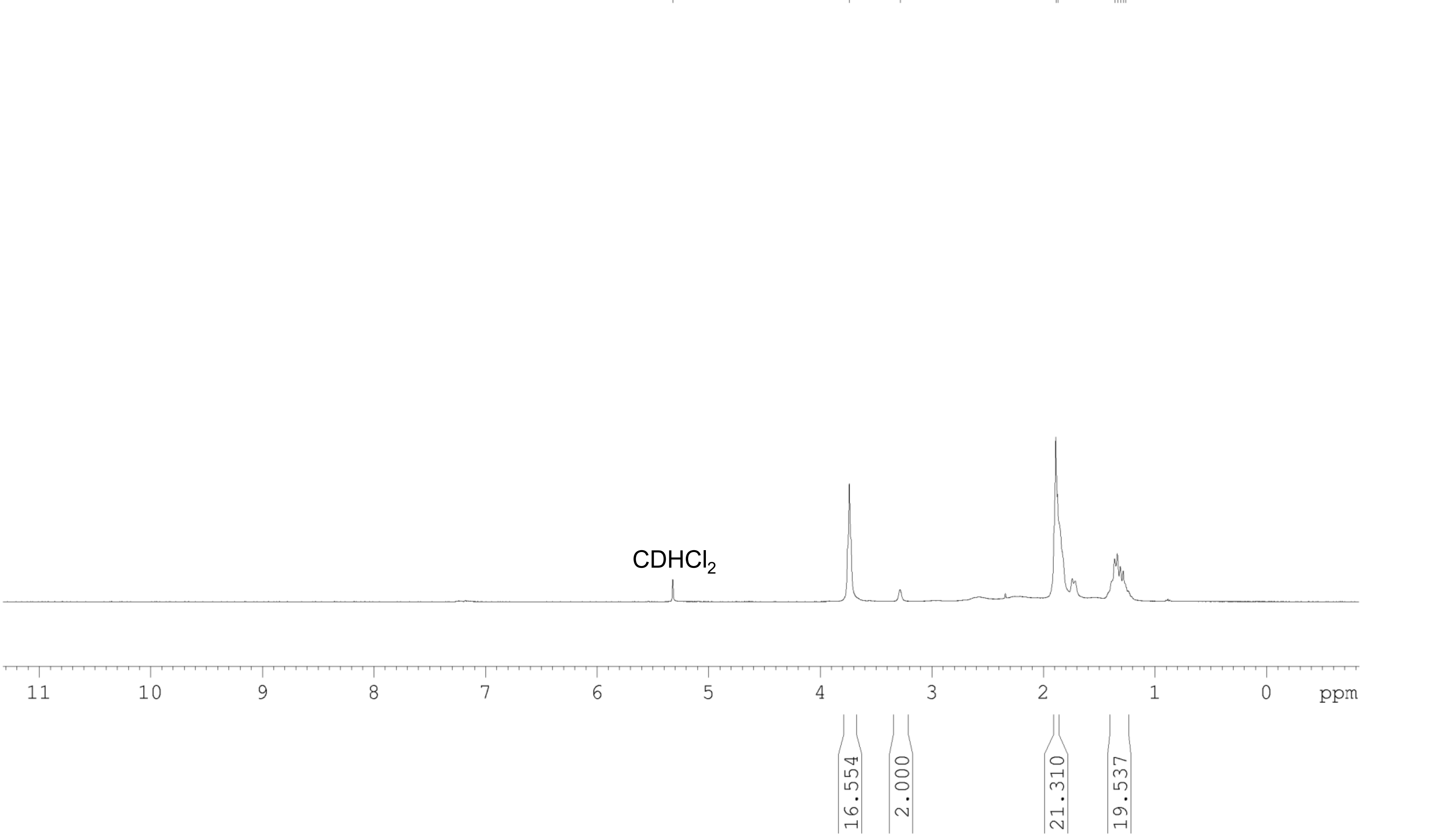

1H NMR, (400 MHz, CD2Cl2)
5.320
3.737
3.283
1.887
1.872
1.360
1.336
1.308
1.283
1.265
CDHCl2
11
10
9
8
7
6
5
4
3
2
1
0
ppm
16.554
2.000
21.310
19.537

^{11}B NMR, (128 MHz, CD_2Cl_2)

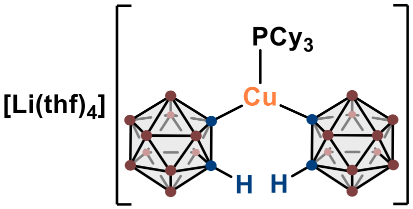

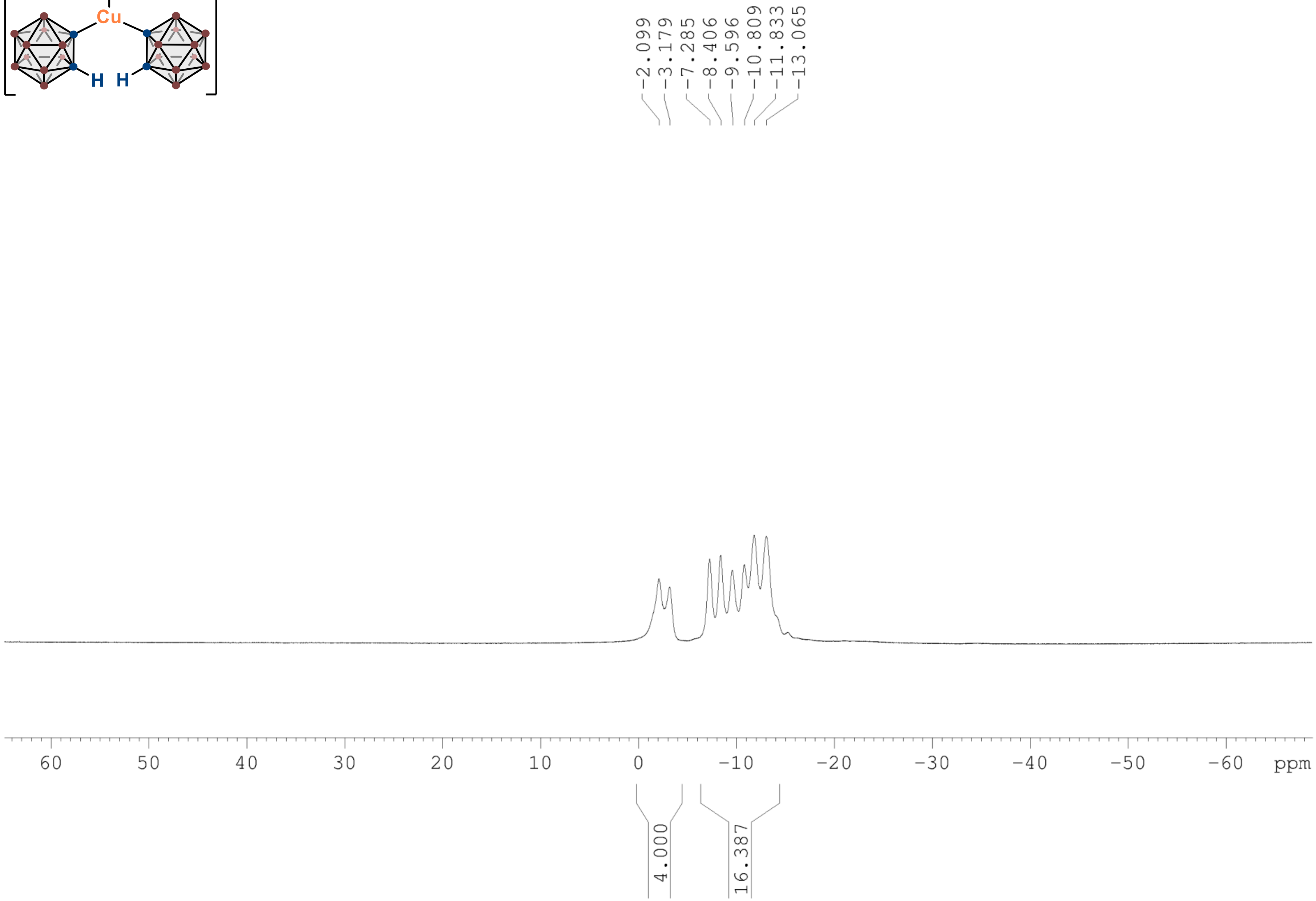

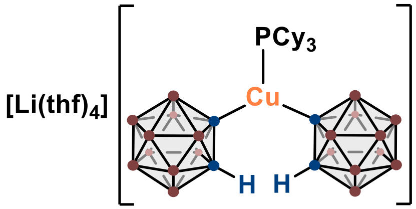
[Li(thf)4]
PCy3
Cu
H H

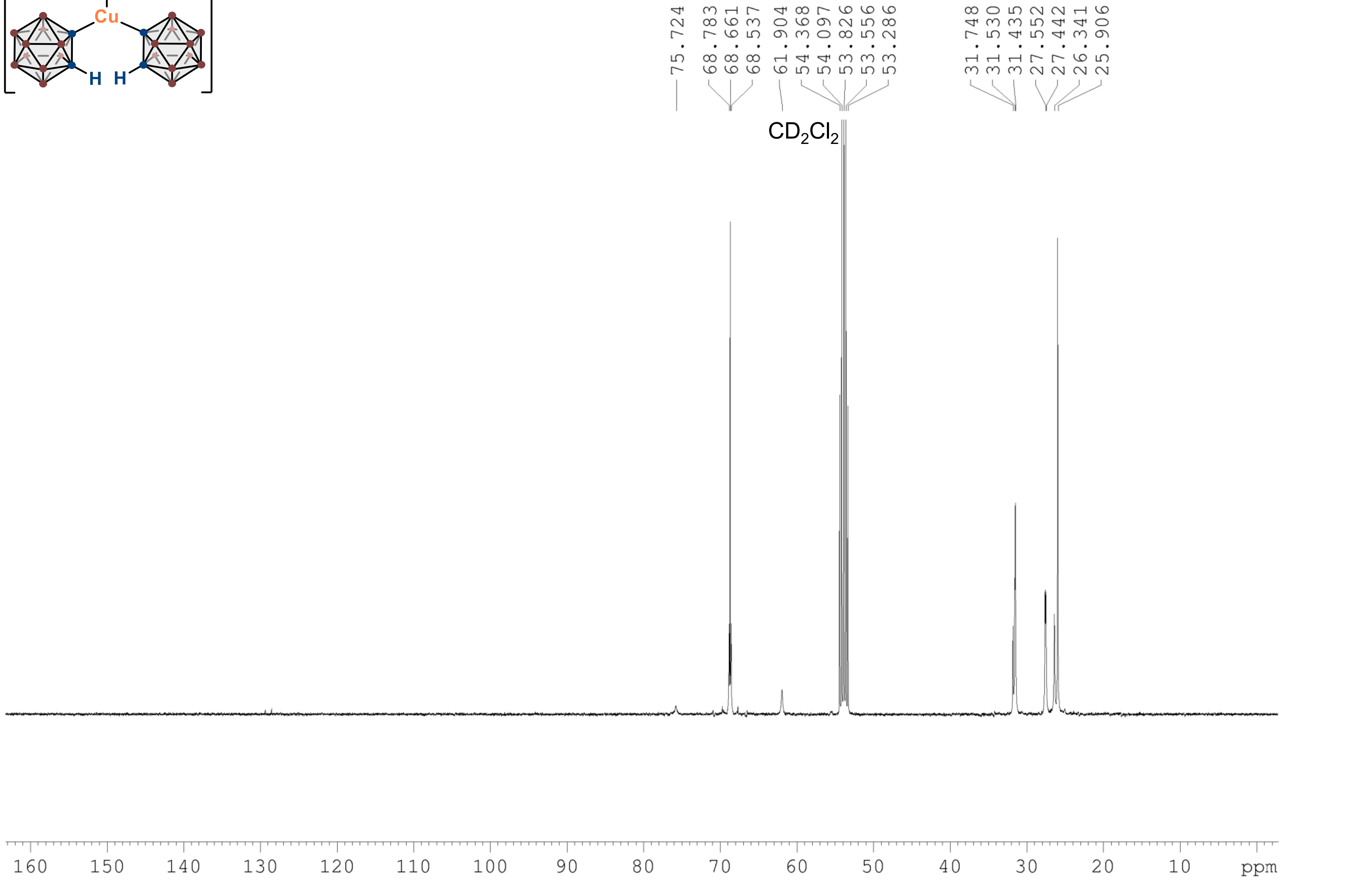
13C NMR, (100 MHz, CD2Cl2)
75.724
68.783
68.661
68.537
61.904
54.368
54.097
53.826
53.556
53.286
31.748
31.530
31.435
27.552
27.442
26.341
25.906
CD2Cl2
160
150
140
130
120
110
100
90
80
70
60
50
40
30
20
10
ppm

^{31}P NMR, (162 MHz, CD_2Cl_2)

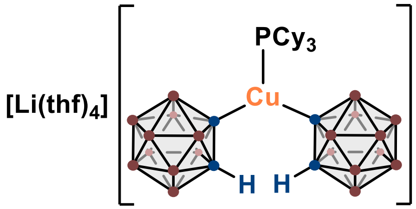

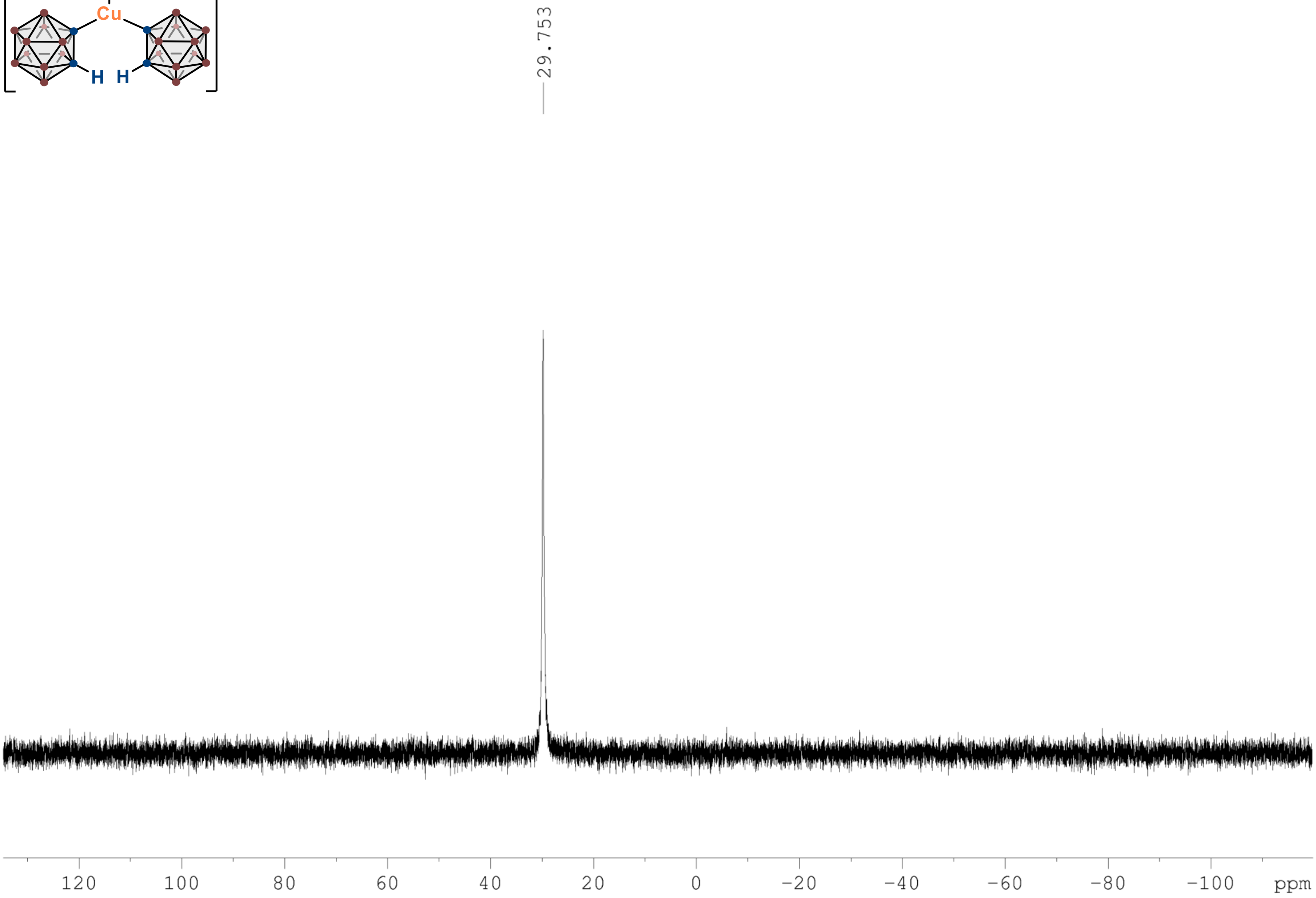

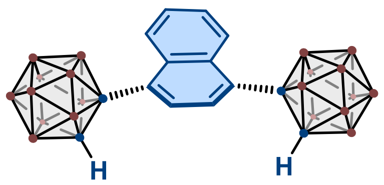

^{1}H NMR, (400 MHz, $CDCl_3$)

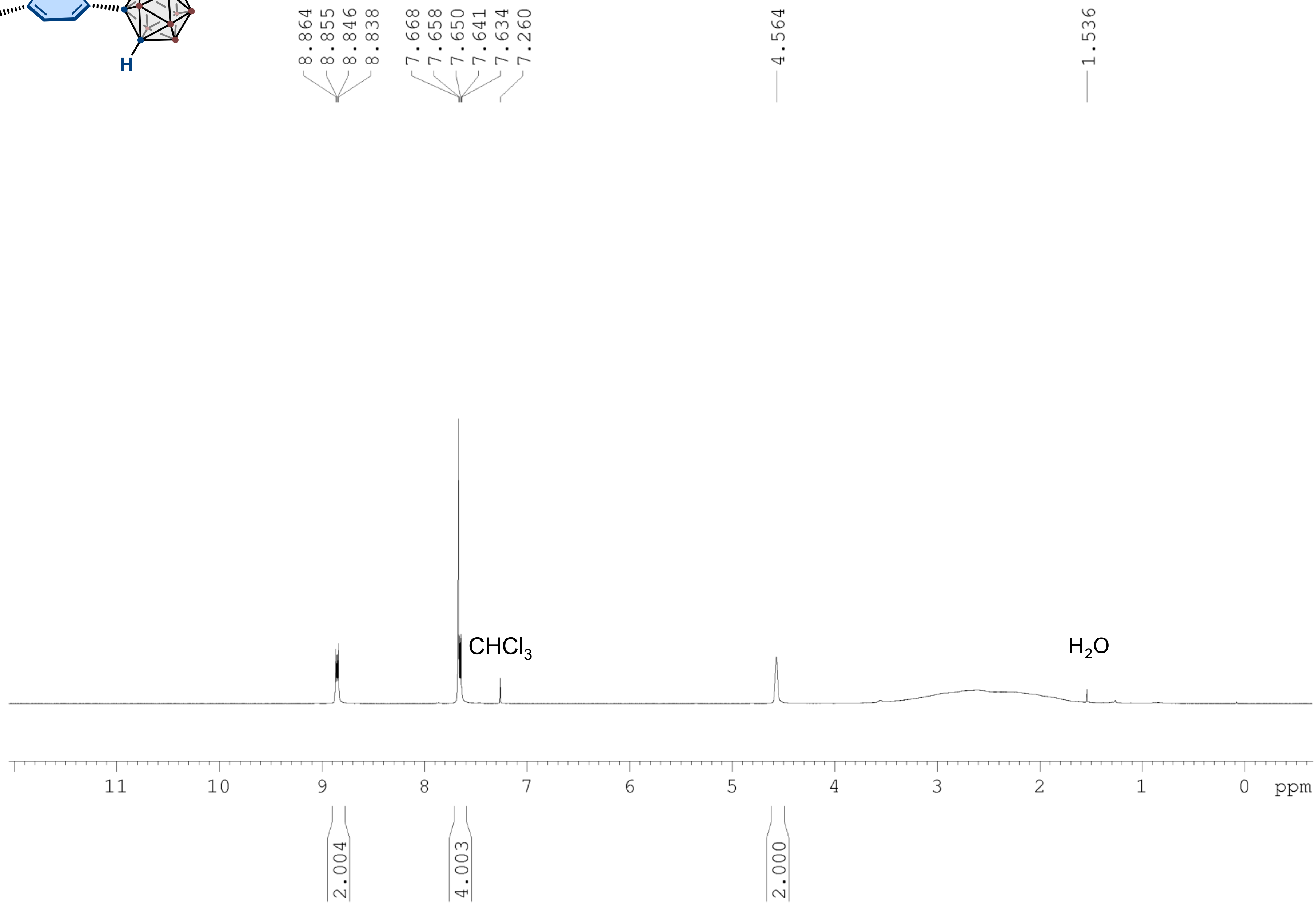

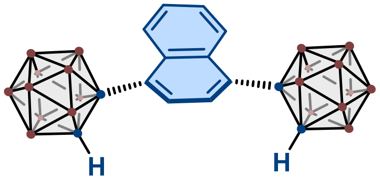

^{11}B NMR, (128 MHz, $CDCl_3$)

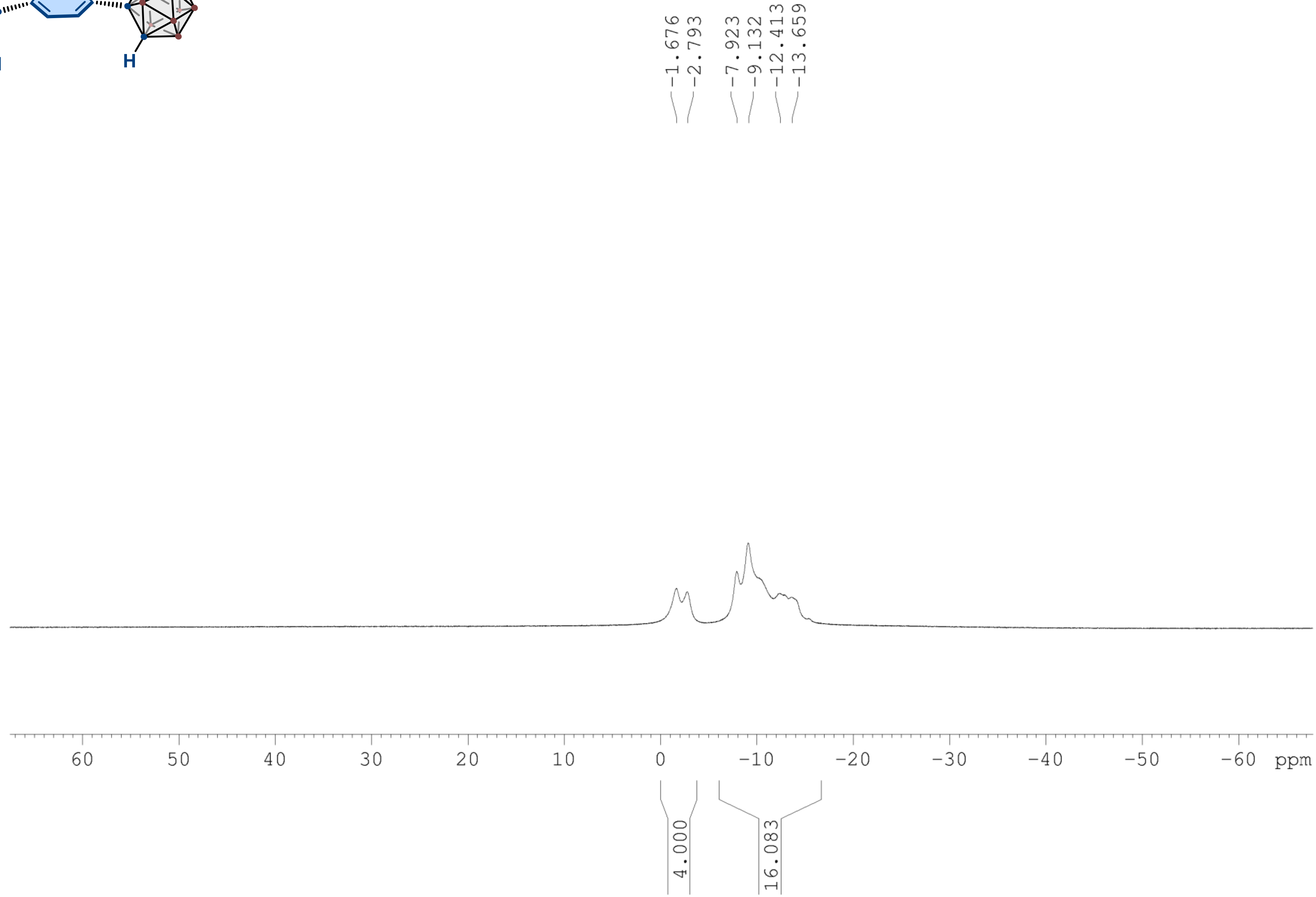

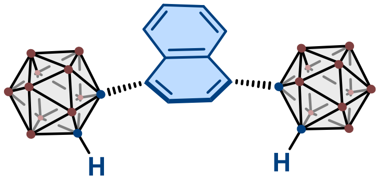

^{13}C NMR, (100 MHz, $CDCl_3$)

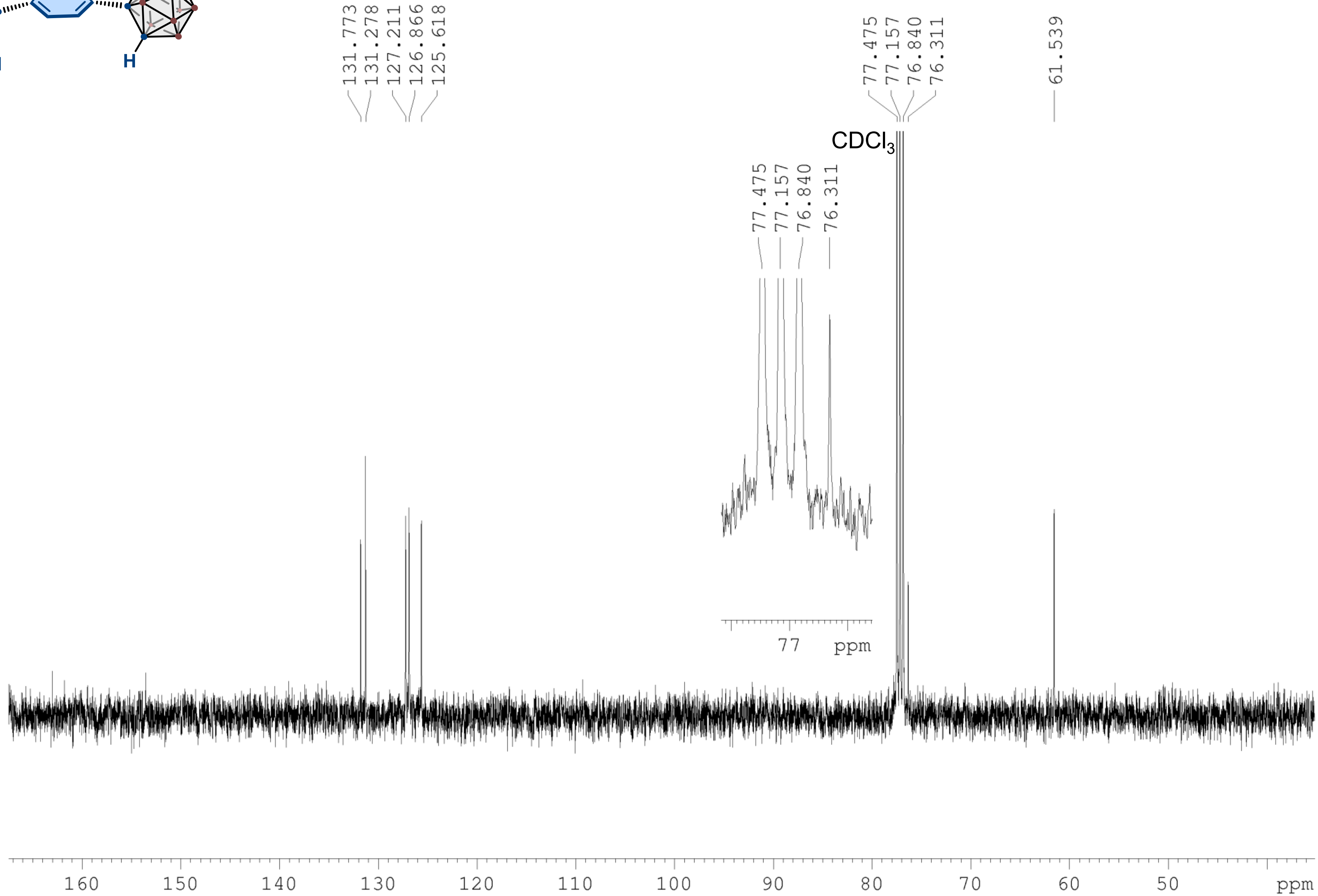

^1H NMR, (400 MHz, $CDCl_3$/acetone-d_6 = 1/1)

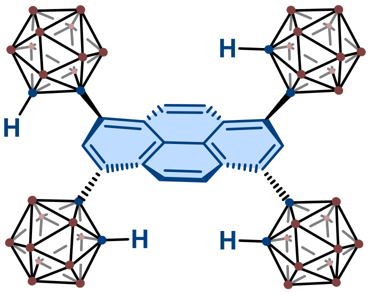

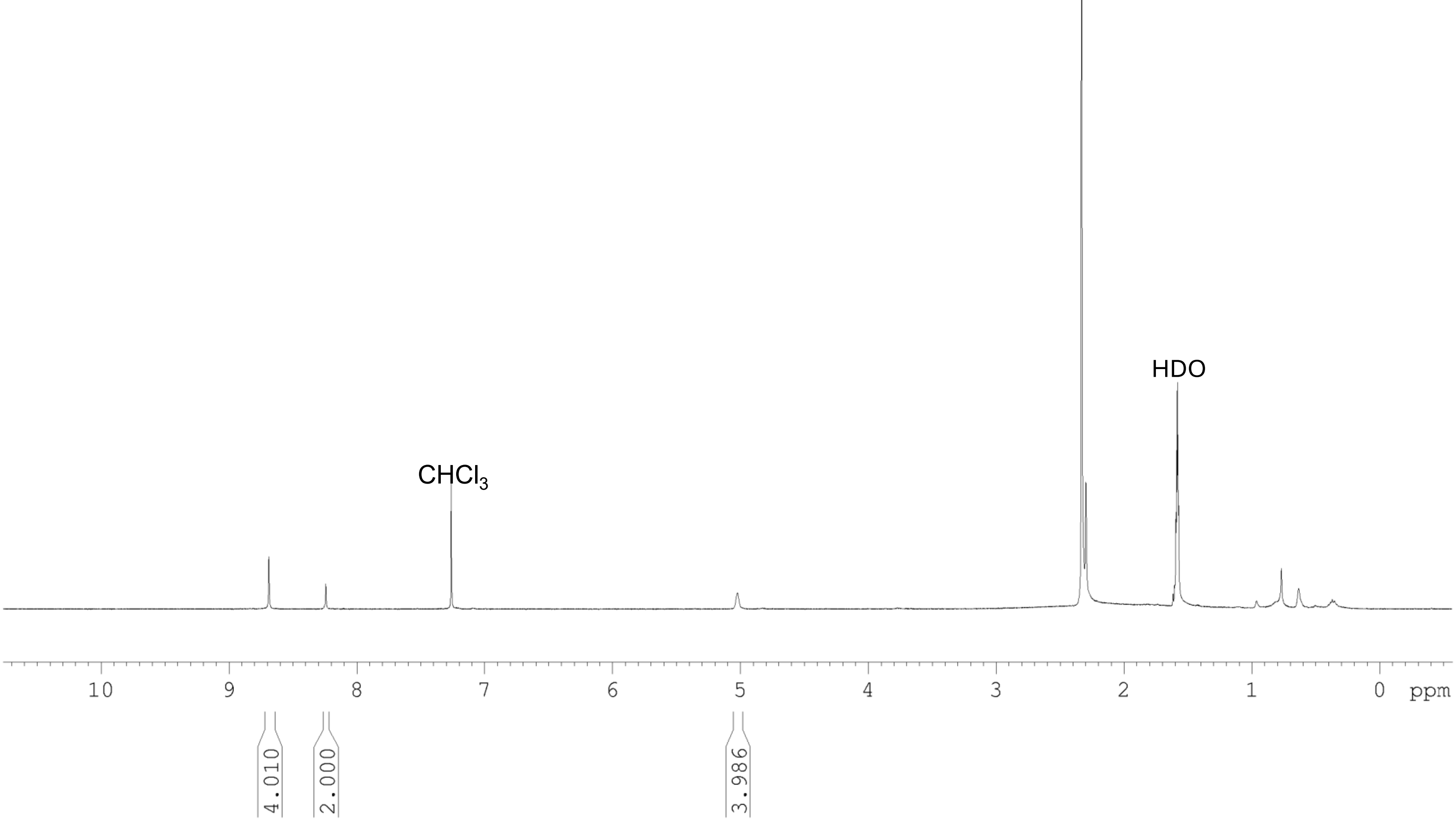

^{11}B NMR, (128 MHz, $CDCl_3$/acetone-d_6 = 1/1)

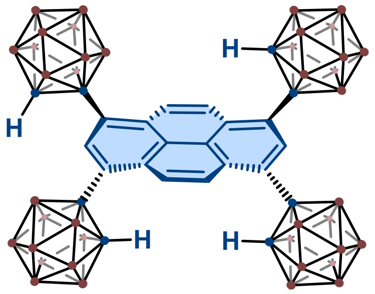

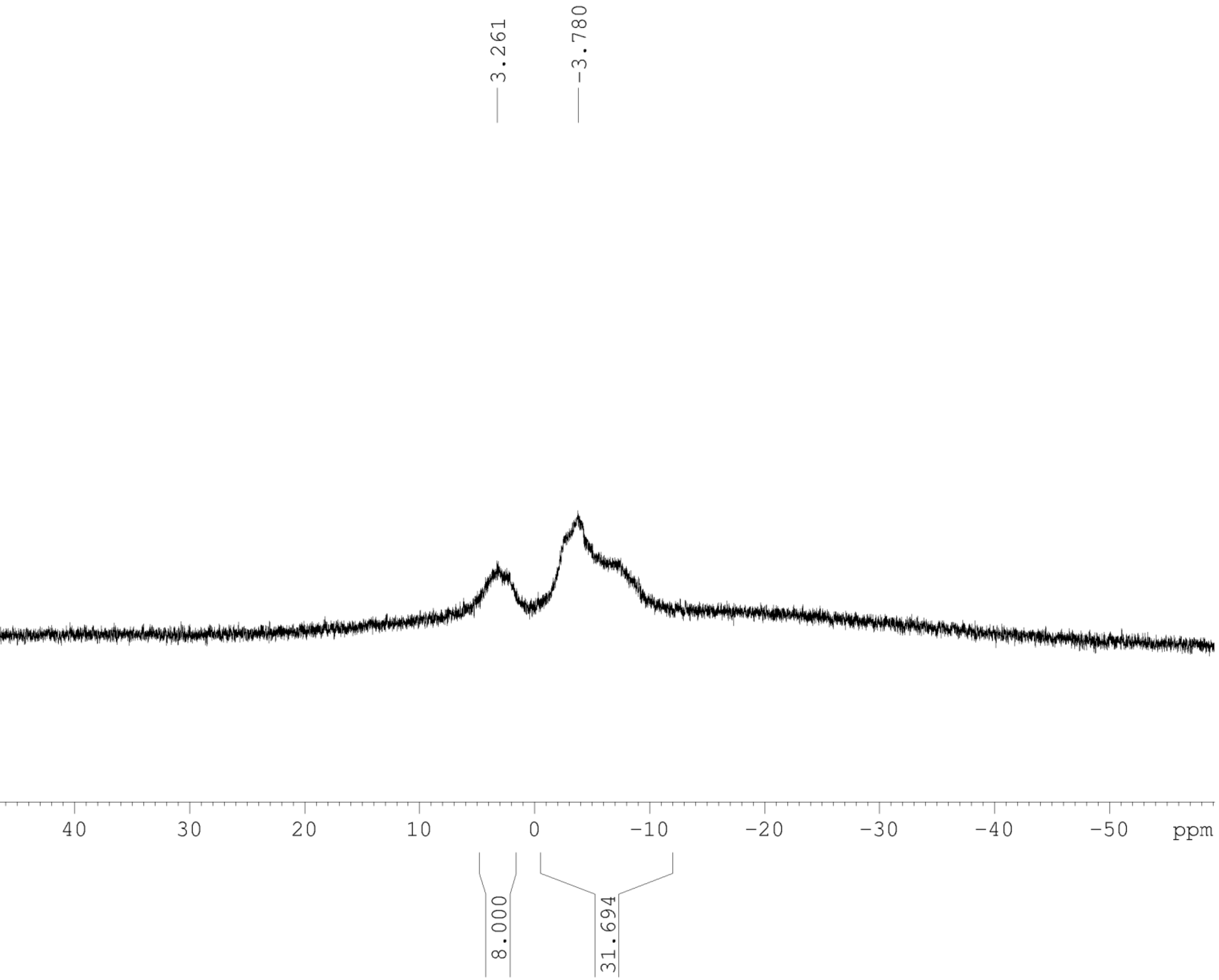

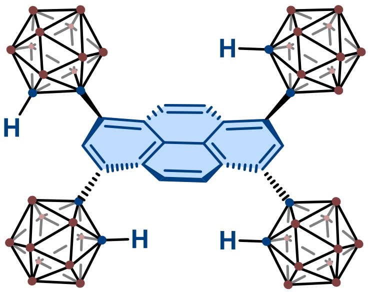

^{13}C NMR, (100 MHz, $CDCl_3$/EtOAc-d_8 = 1/1)

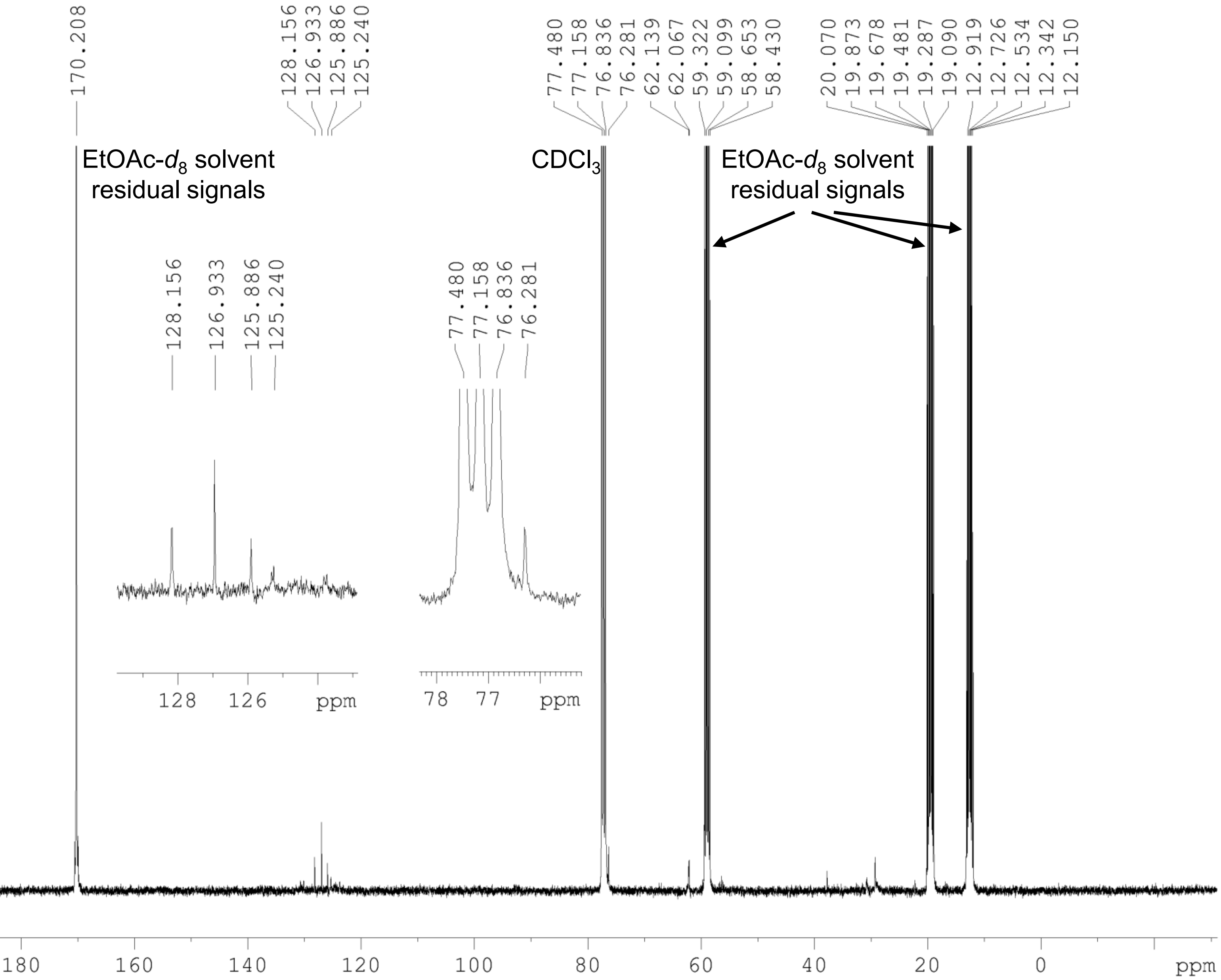

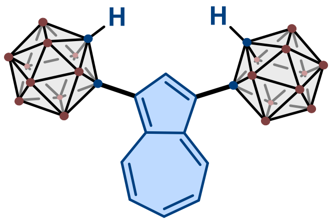

^1H NMR, (400 MHz, $CDCl_3$)

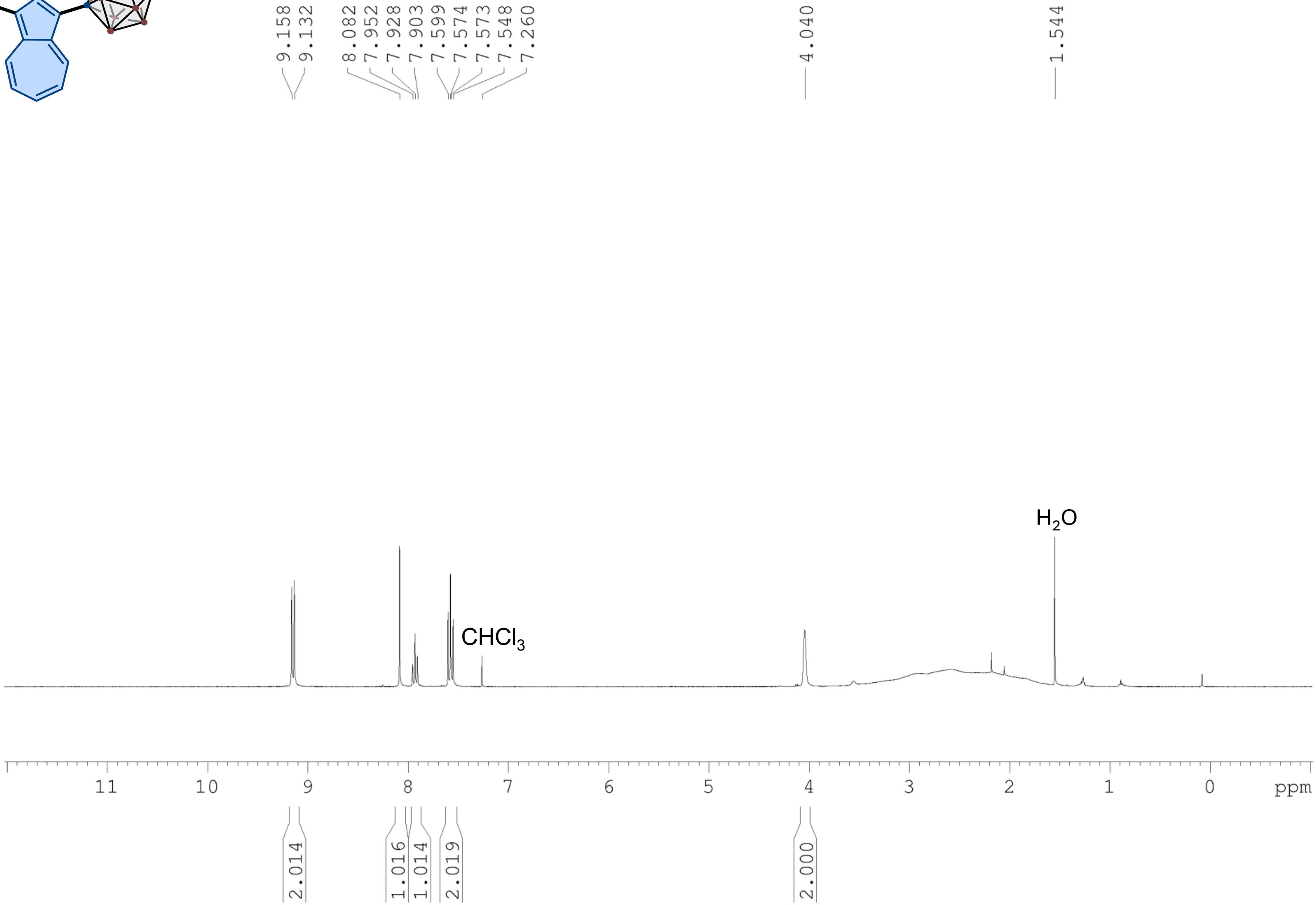

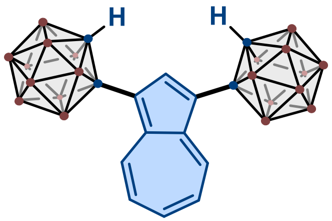

^{11}B NMR, (128 MHz, $CDCl_3$)

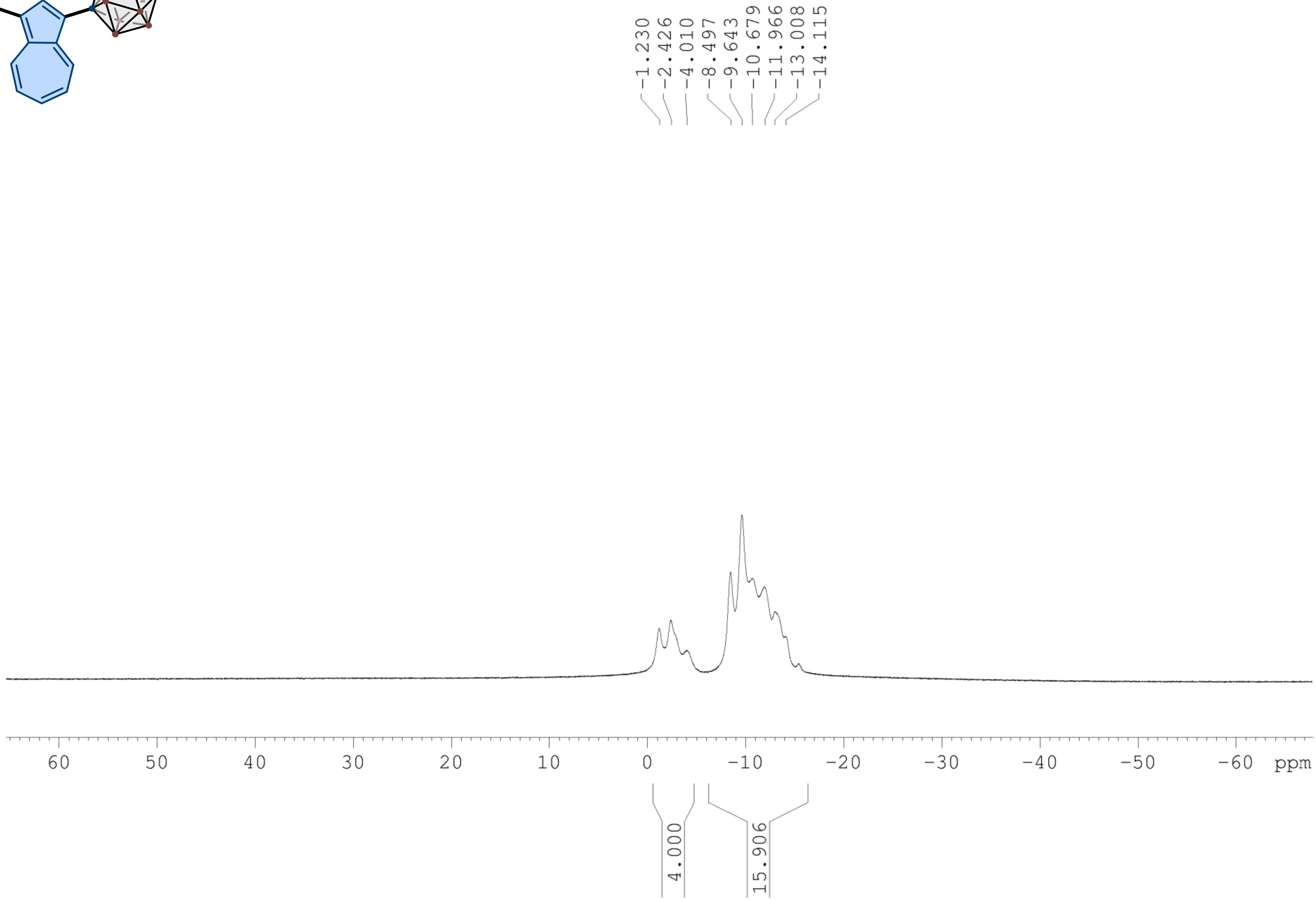

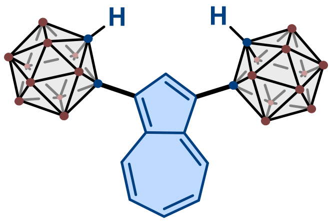

^{13}C NMR, (100 MHz, $CDCl_3$)

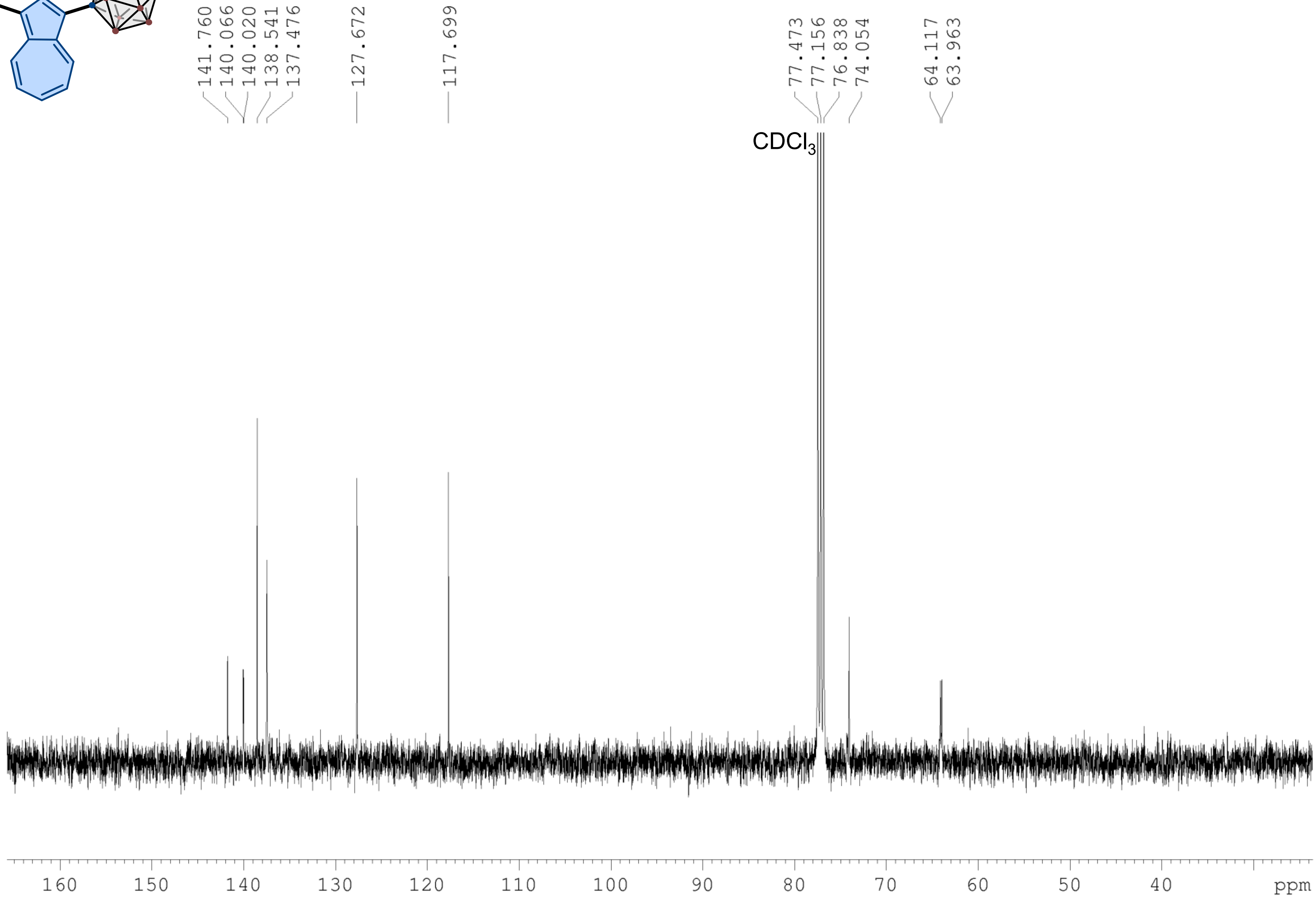

^1H NMR, (400 MHz, $CDCl_3$)

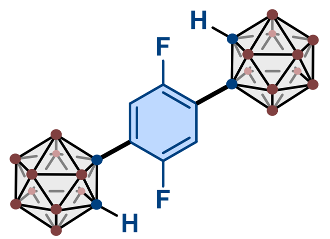

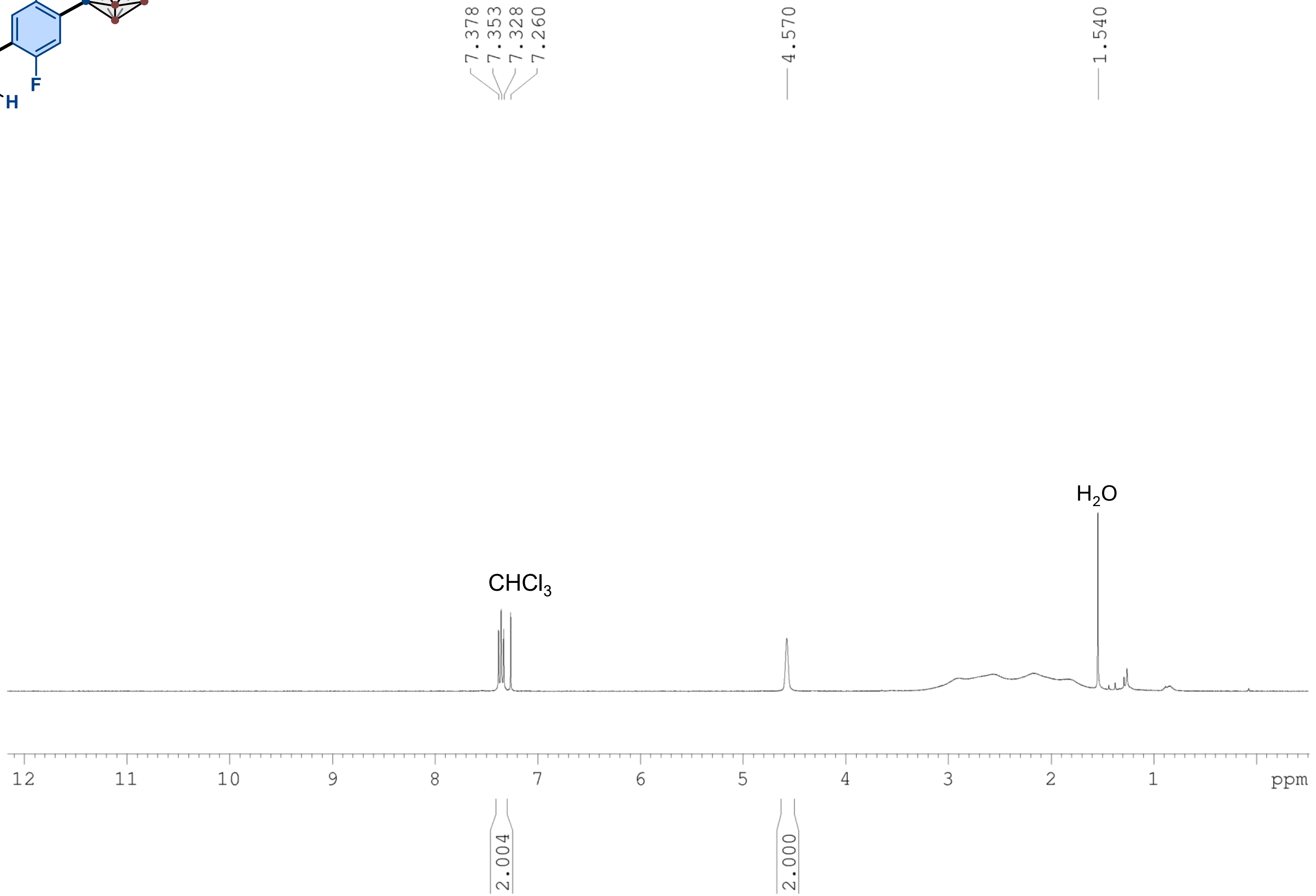

^{11}B NMR, (128 MHz, $CDCl_3$)

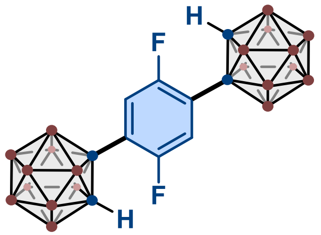

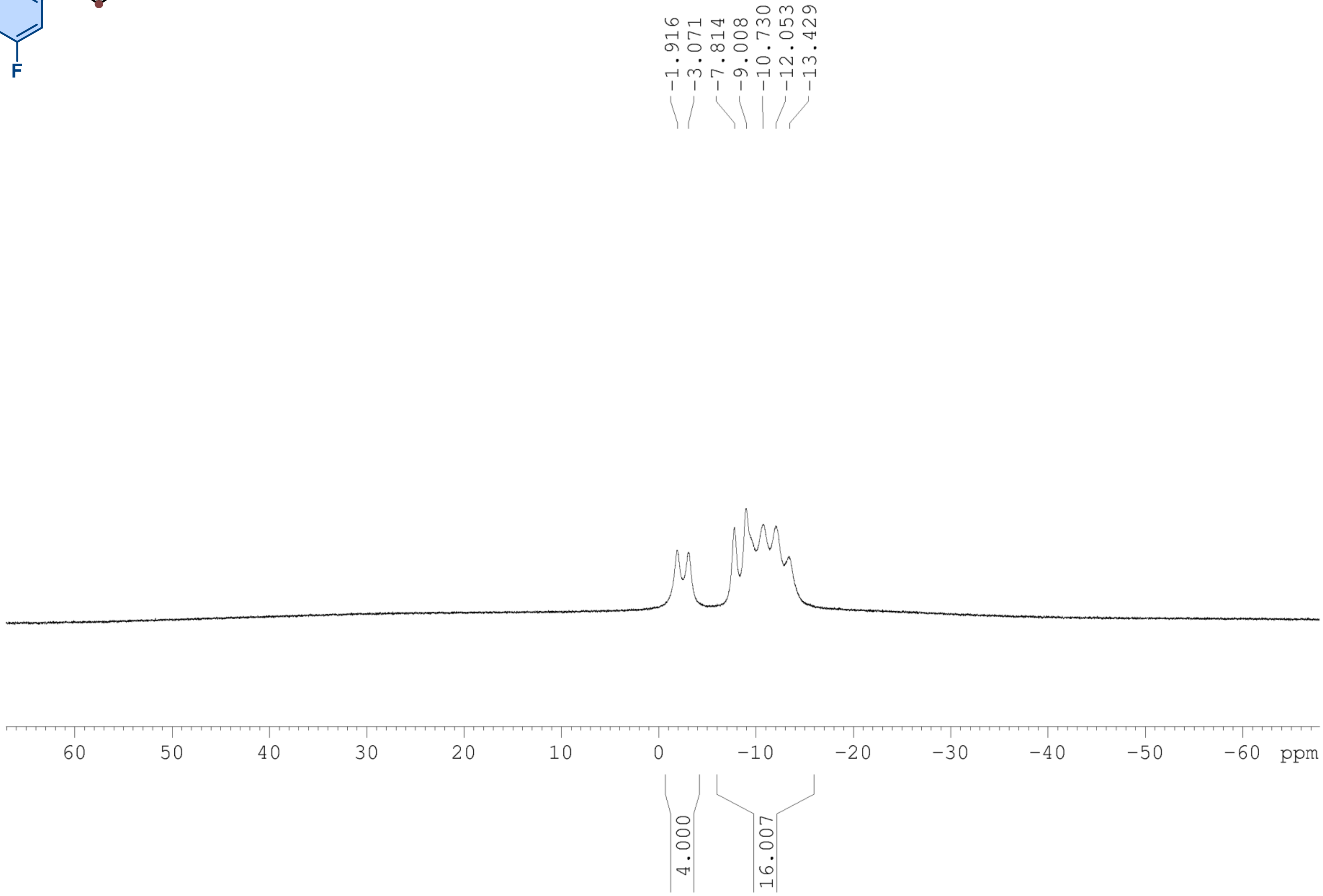

^{13}C NMR, (100 MHz, $CDCl_3$)

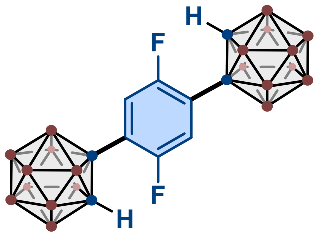

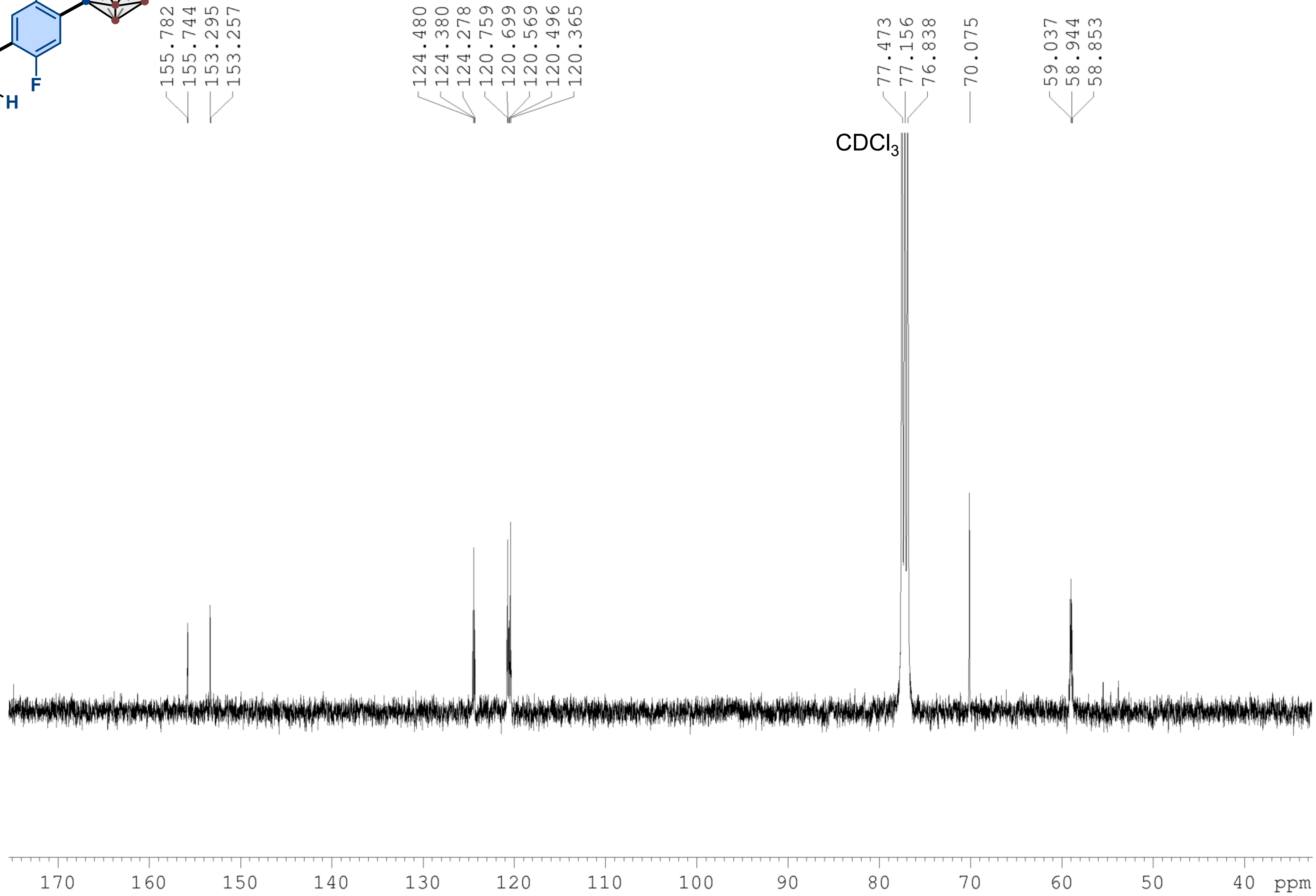

^{19}F NMR, (376 MHz, $CDCl_3$)

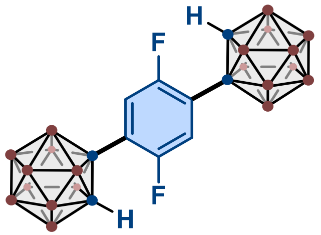

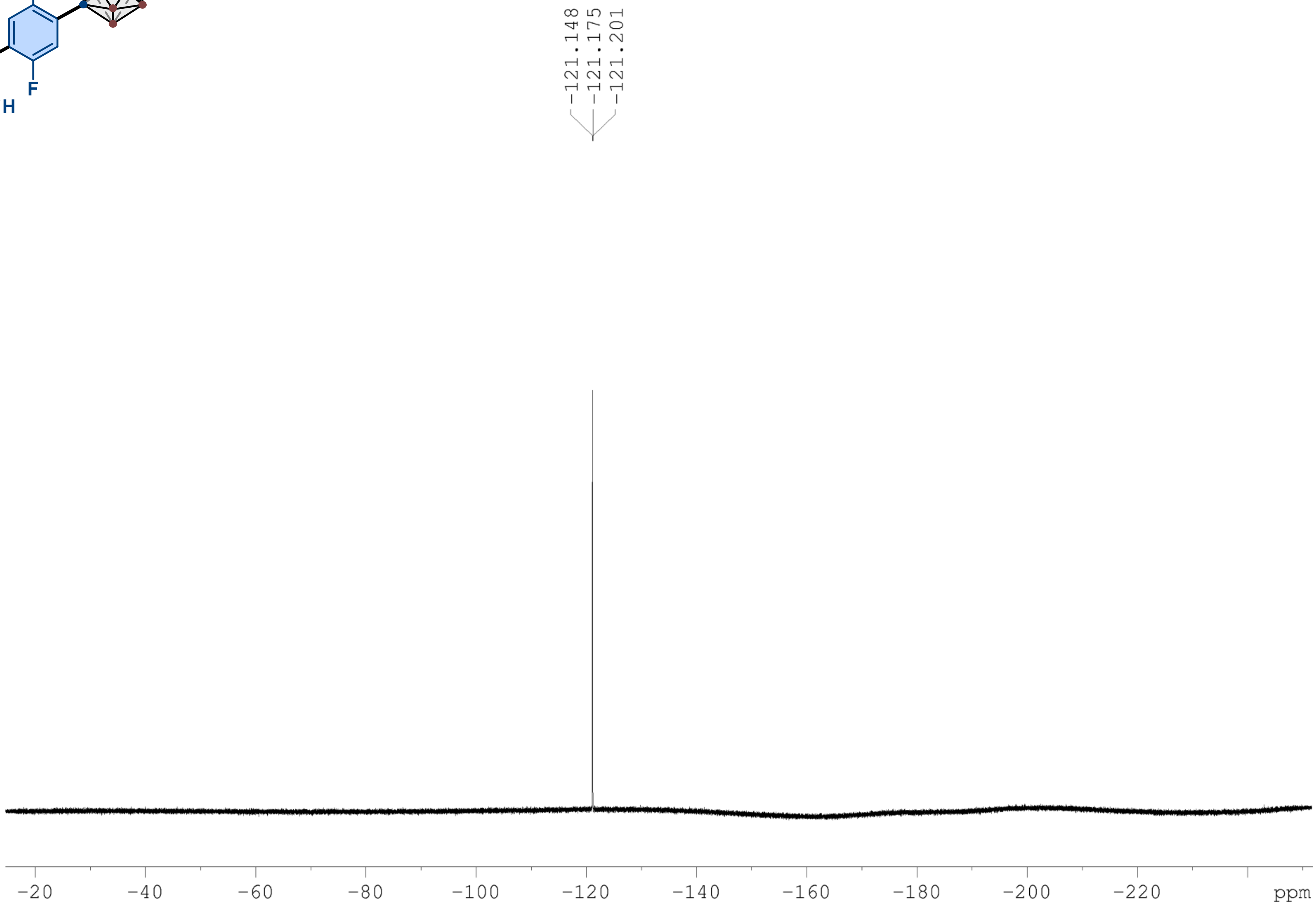

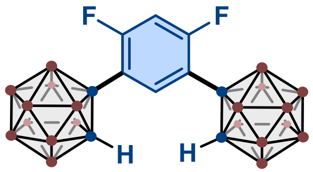

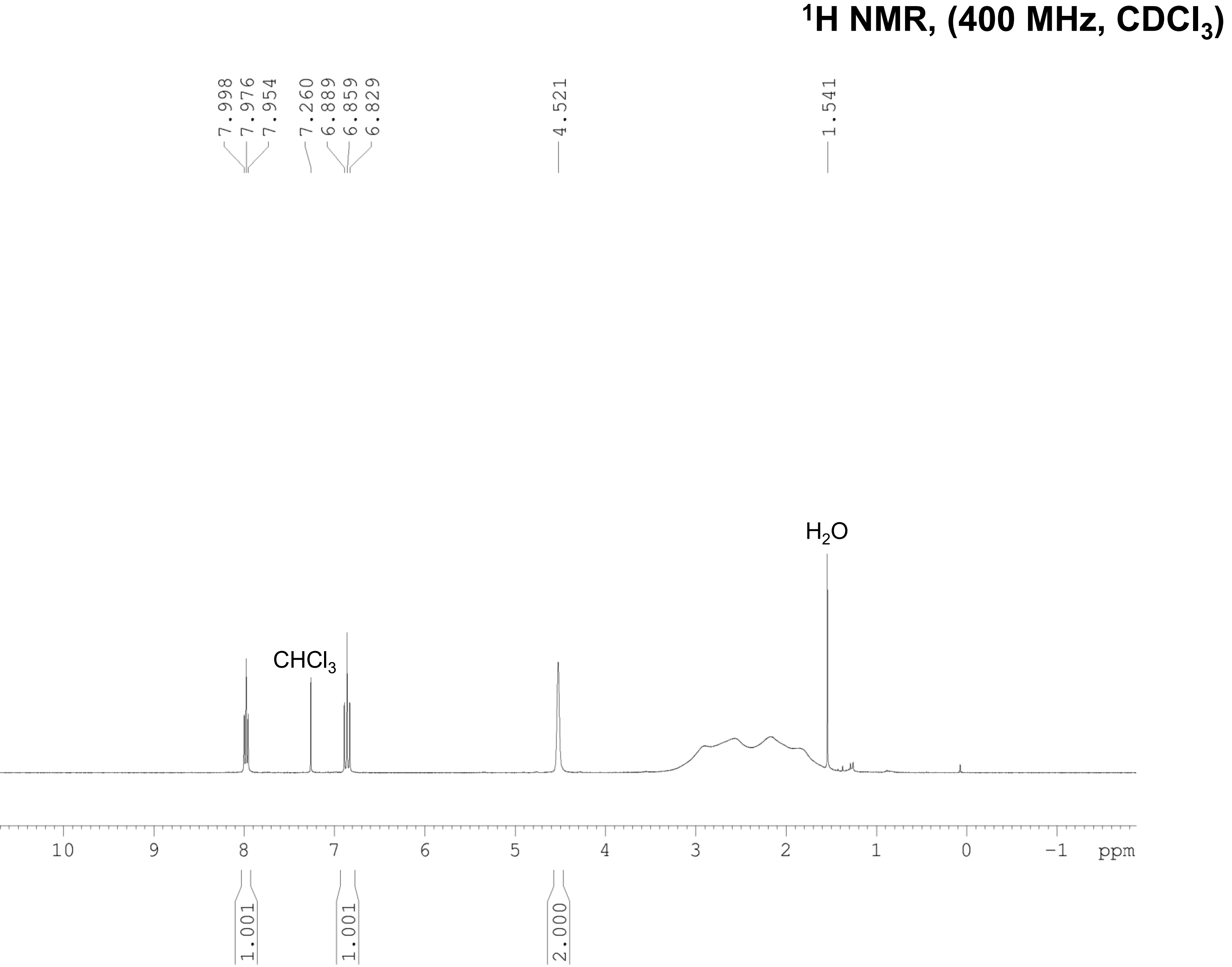

^{1}H NMR, (400 MHz, CDCl$_3$)
7.998
7.976
7.954
7.260
6.889
6.859
6.829
4.521
1.541
CHCl$_3$
H$_2$O
12
11
10
9
8
7
6
5
4
3
2
1
0
-1
ppm
1.001
1.001
2.000

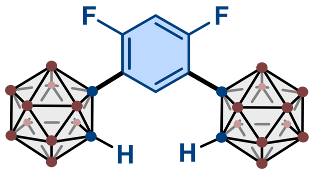

^{11}B NMR, (128 MHz, $CDCl_3$)

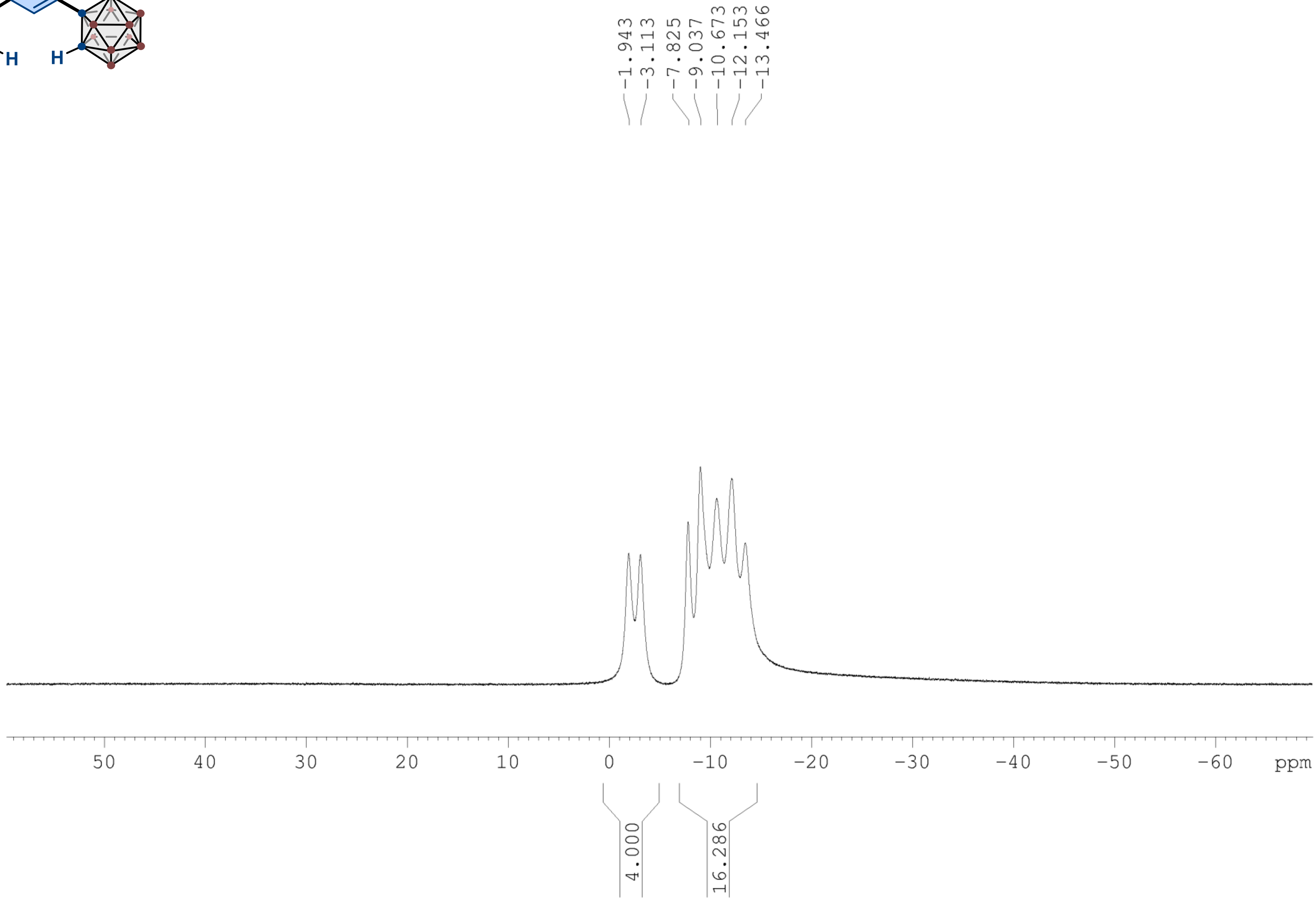

^{13}C NMR, (100 MHz, $CDCl_3$)

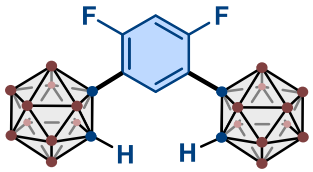

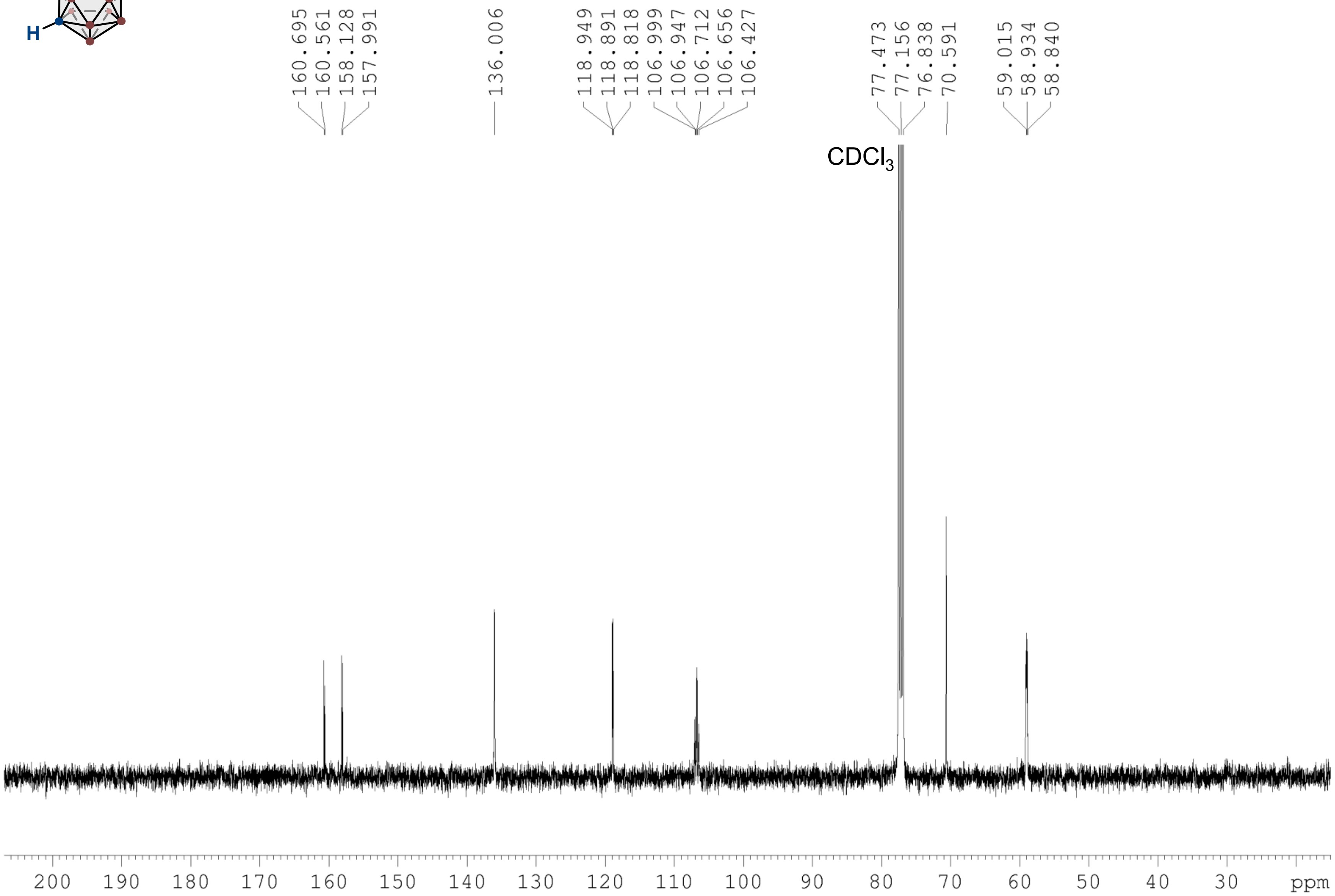

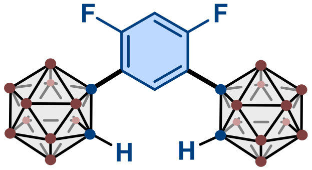

^{19}F NMR, (376 MHz, $CDCl_3$)

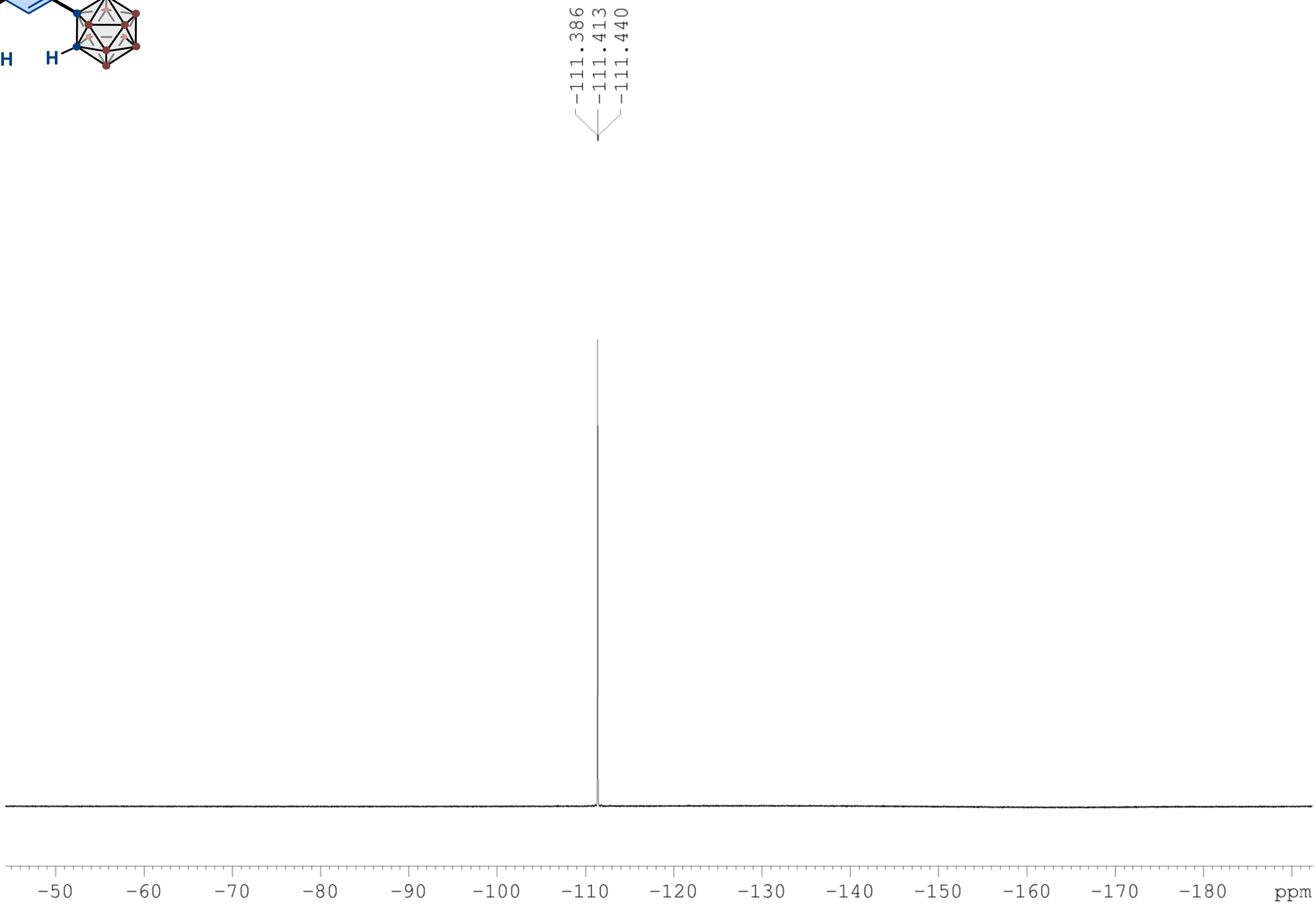

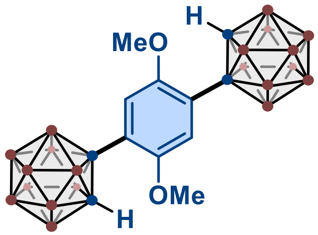

^{1}H NMR, (400 MHz, $CDCl_3$)

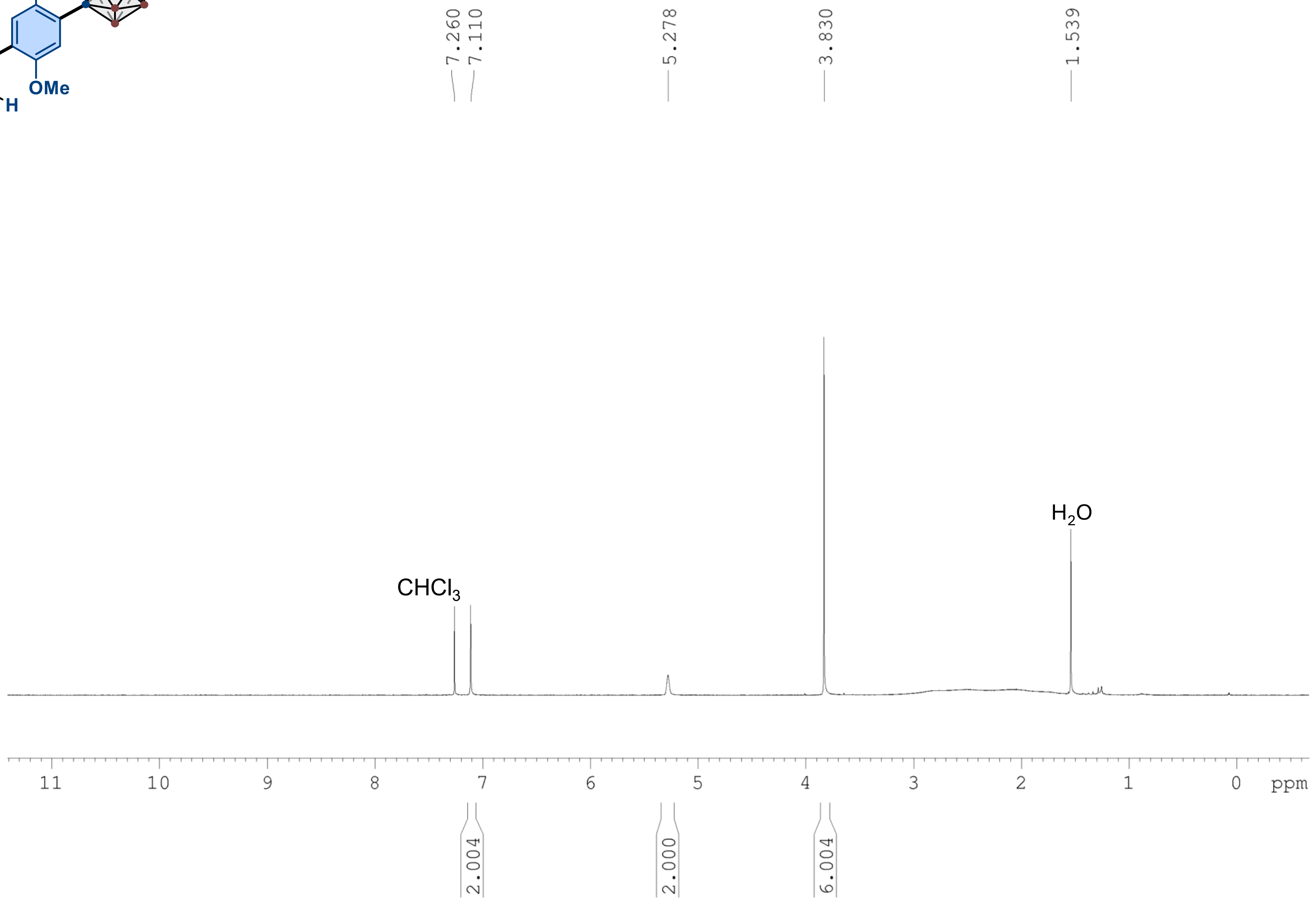

^{11}B NMR, (128 MHz, $CDCl_3$)

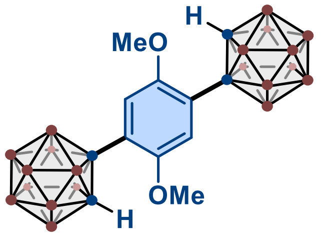

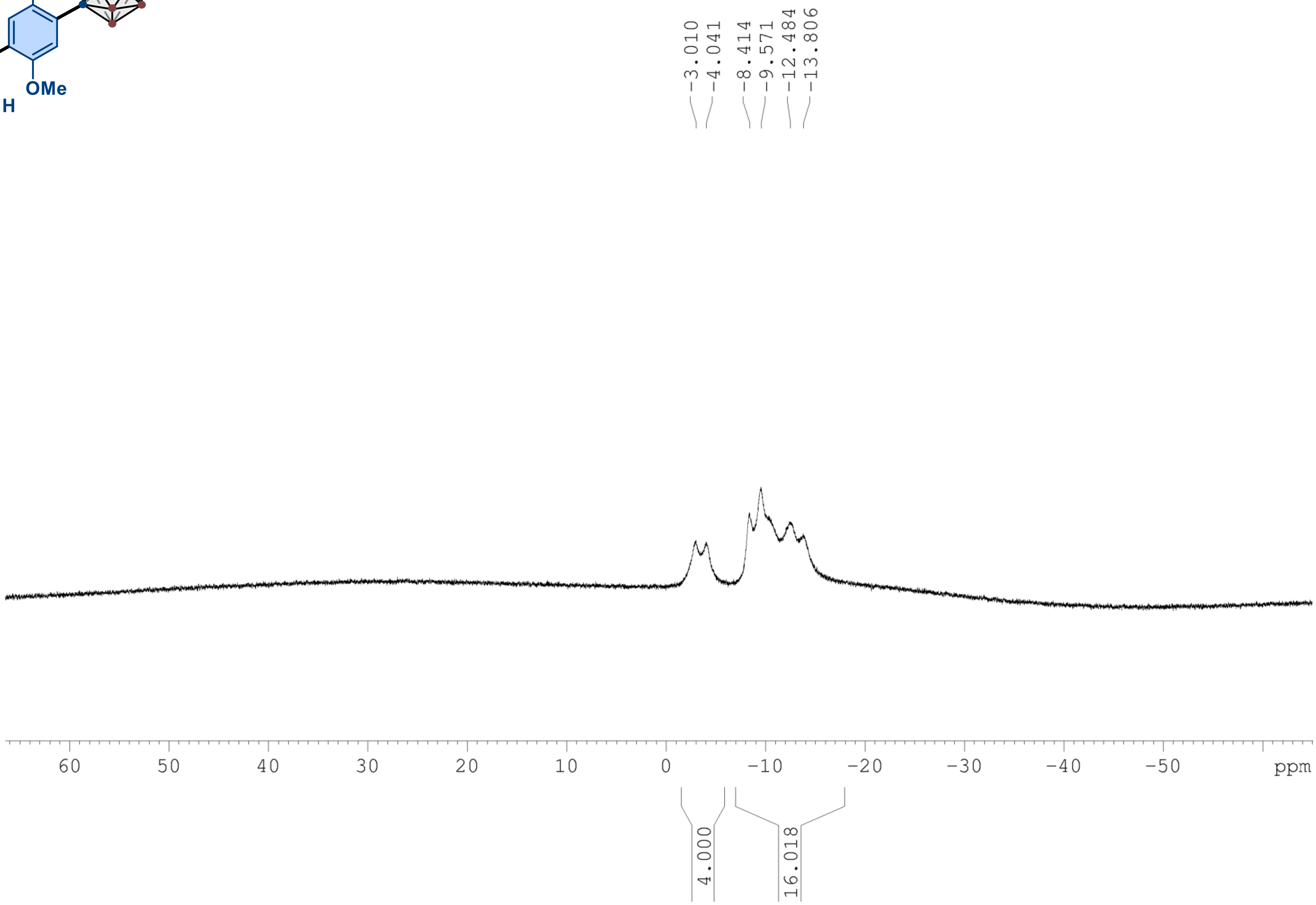

^{13}C NMR, (100 MHz, $CDCl_3$)

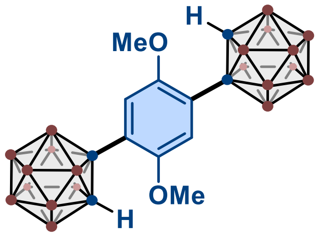

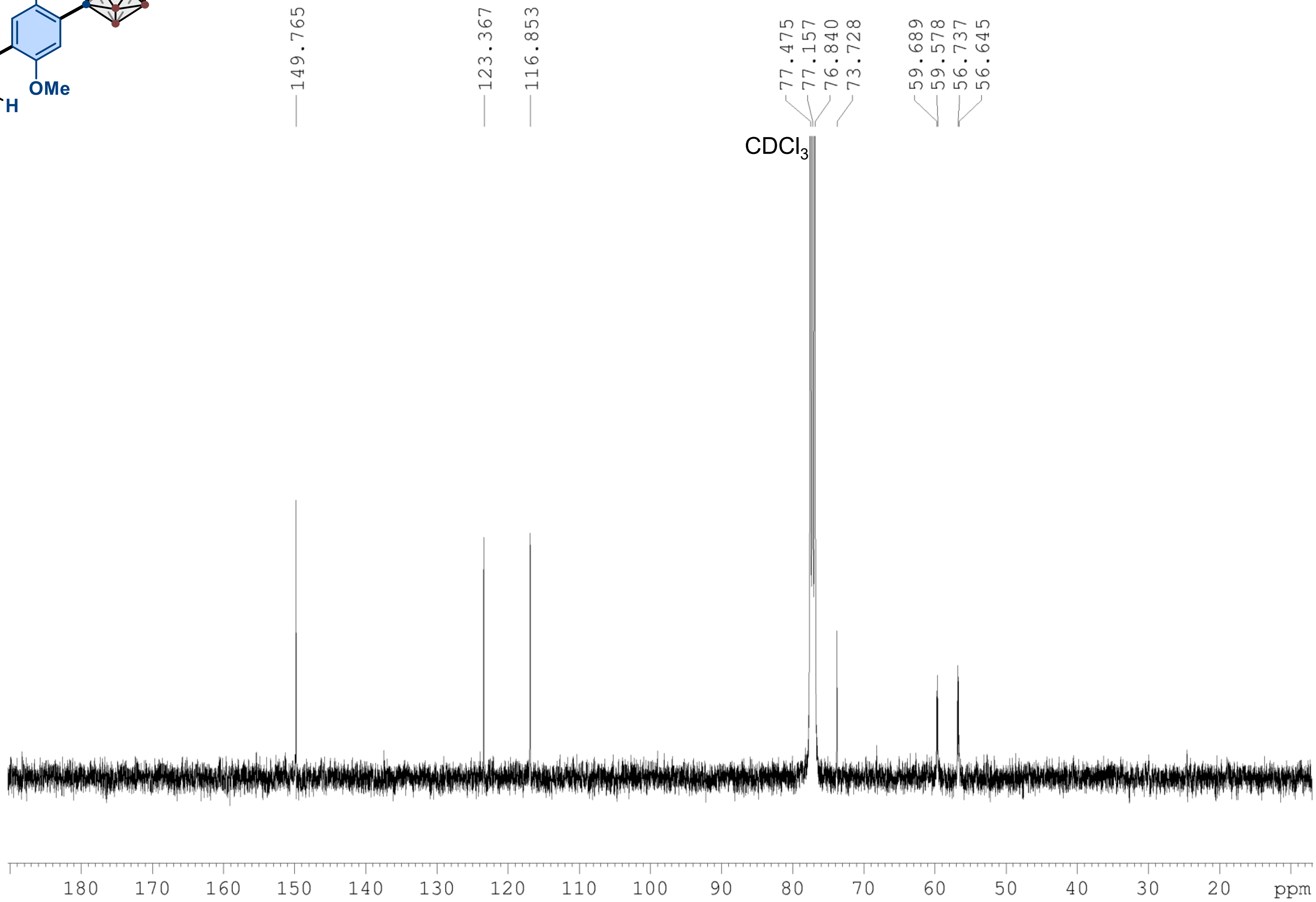

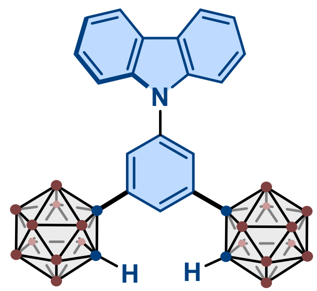

^{1}H NMR, (400 MHz, $CDCl_3$)

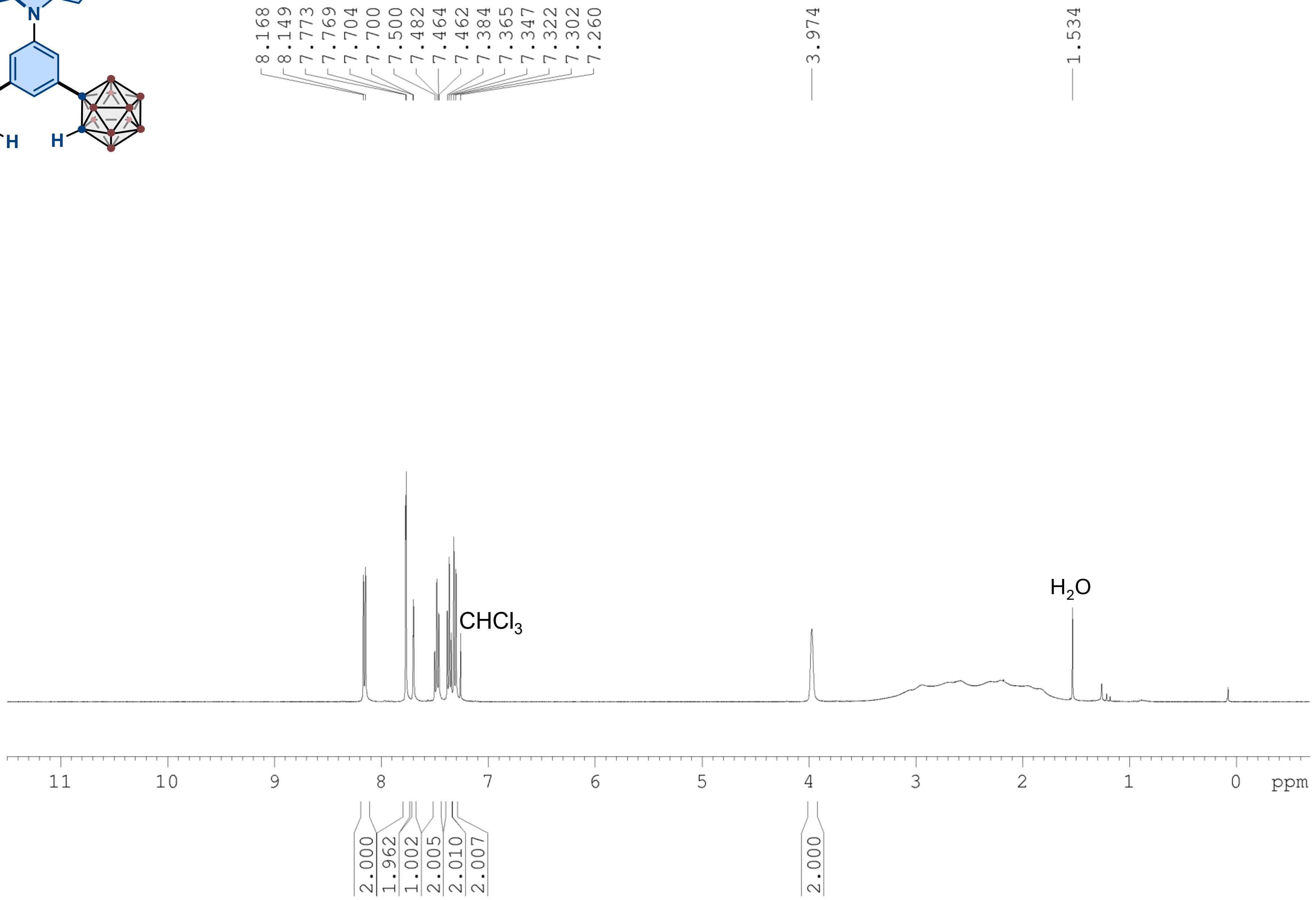

^{11}B NMR, (128 MHz, $CDCl_3$)

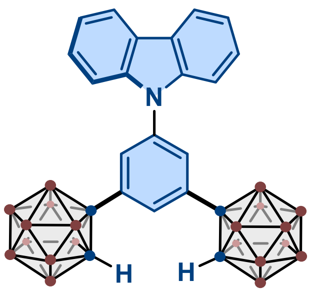

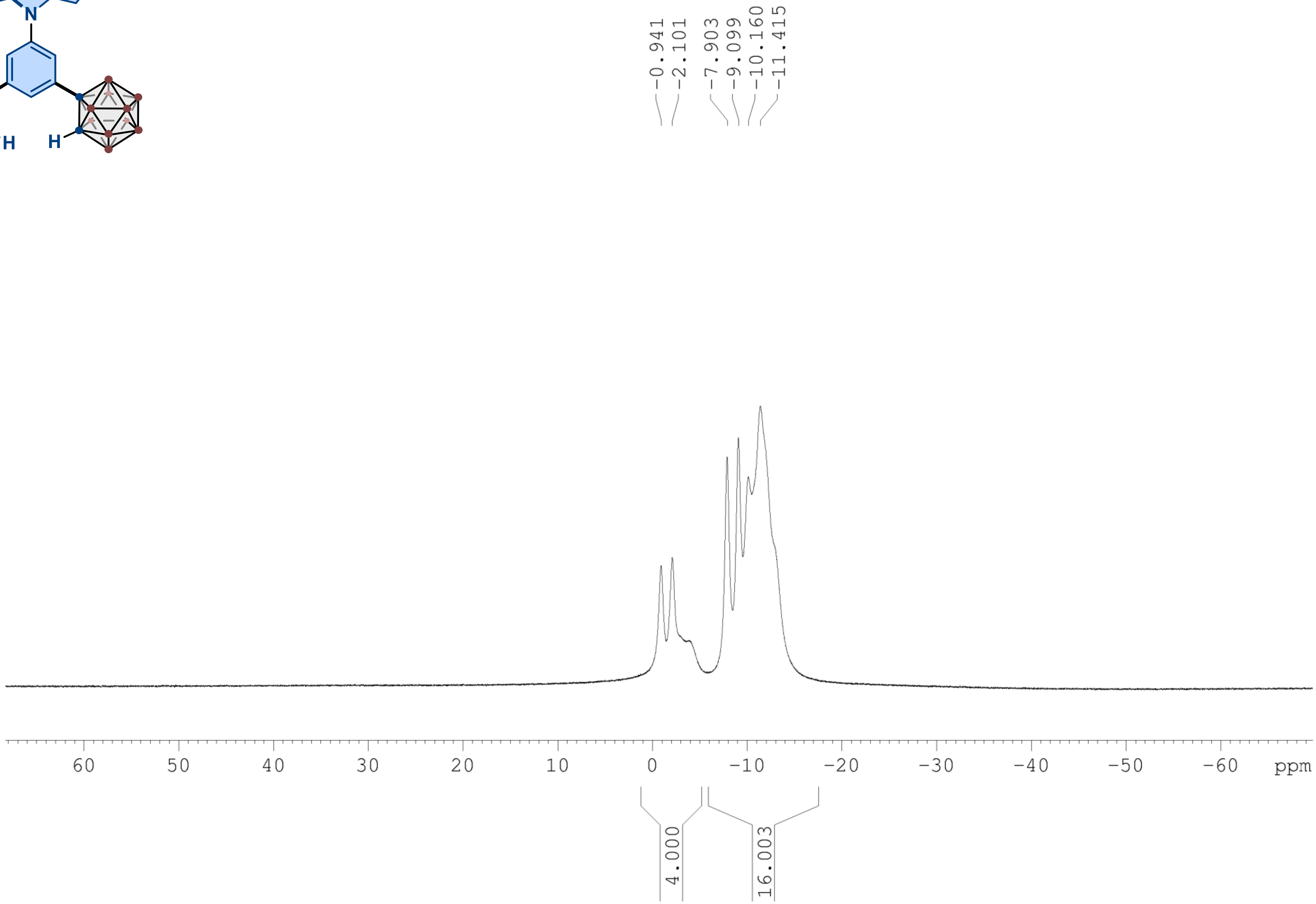

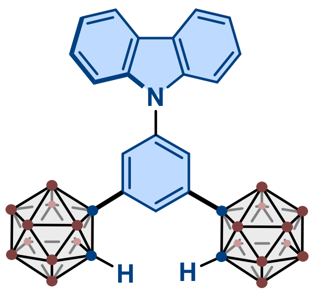

^{13}C NMR, (100 MHz, $CDCl_3$)

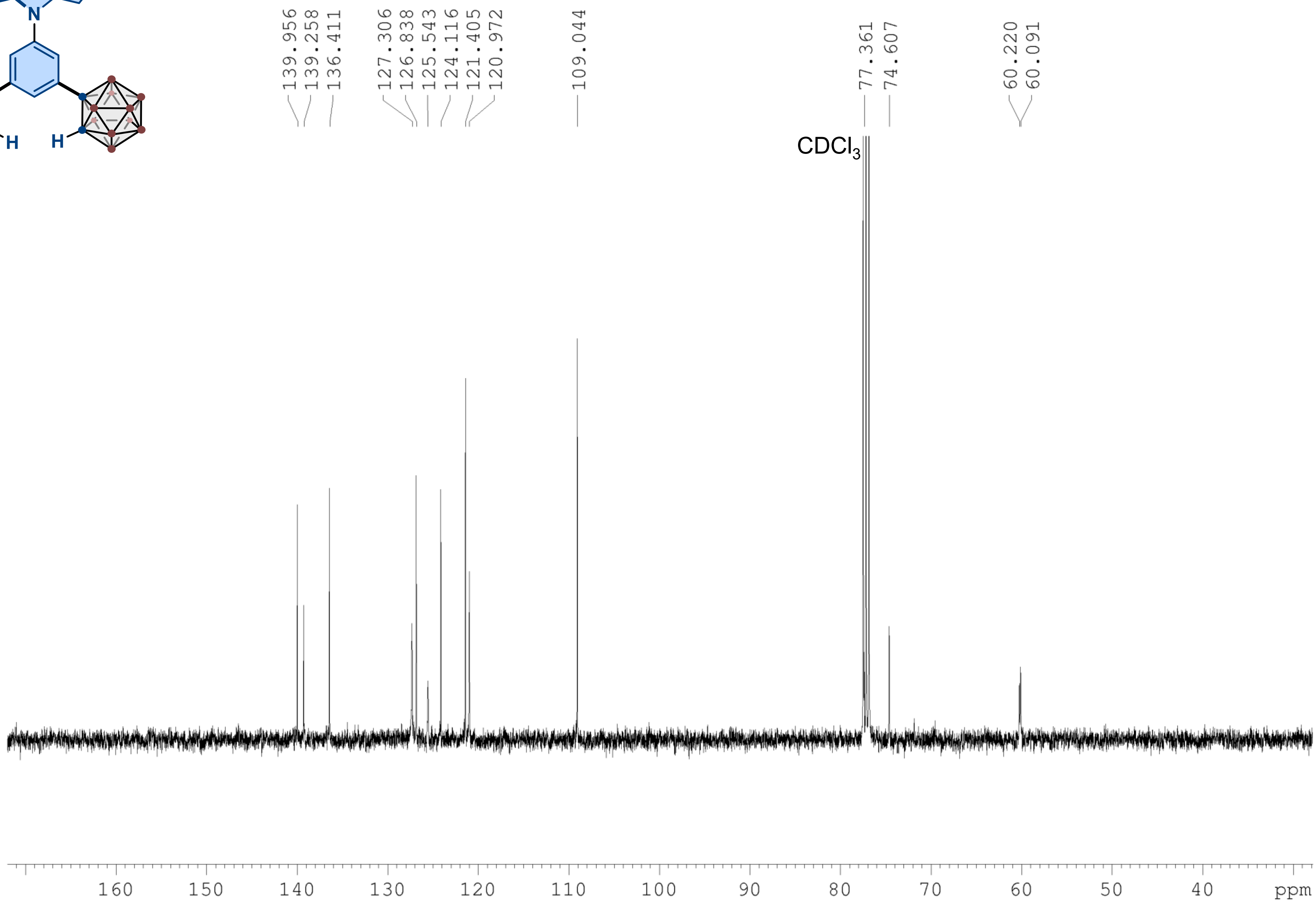

^1H NMR, (400 MHz, $CDCl_3$)

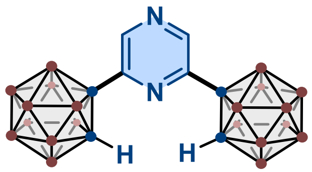

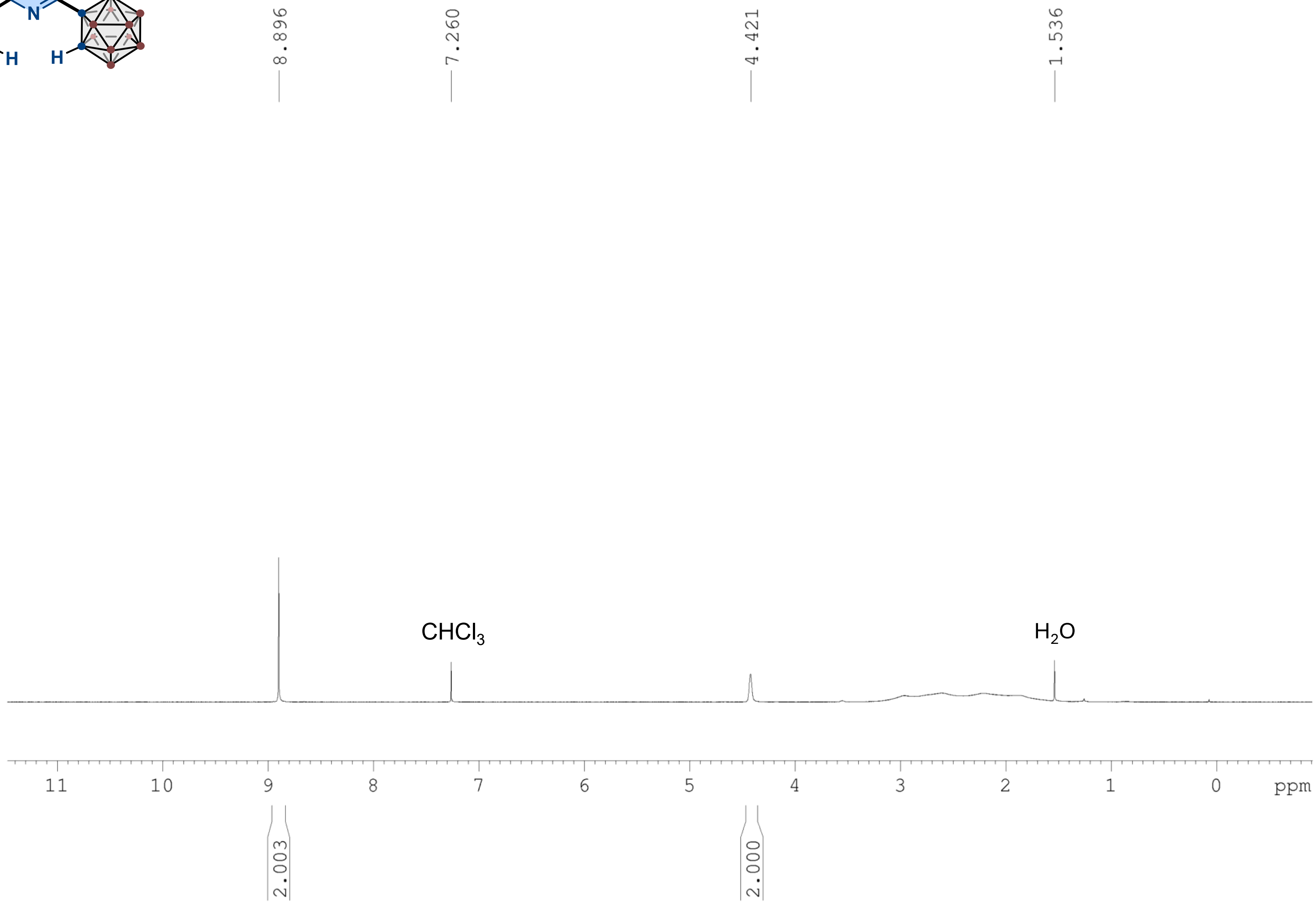

^{11}B NMR, (128 MHz, $CDCl_3$)

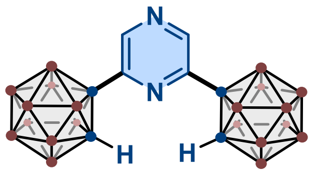

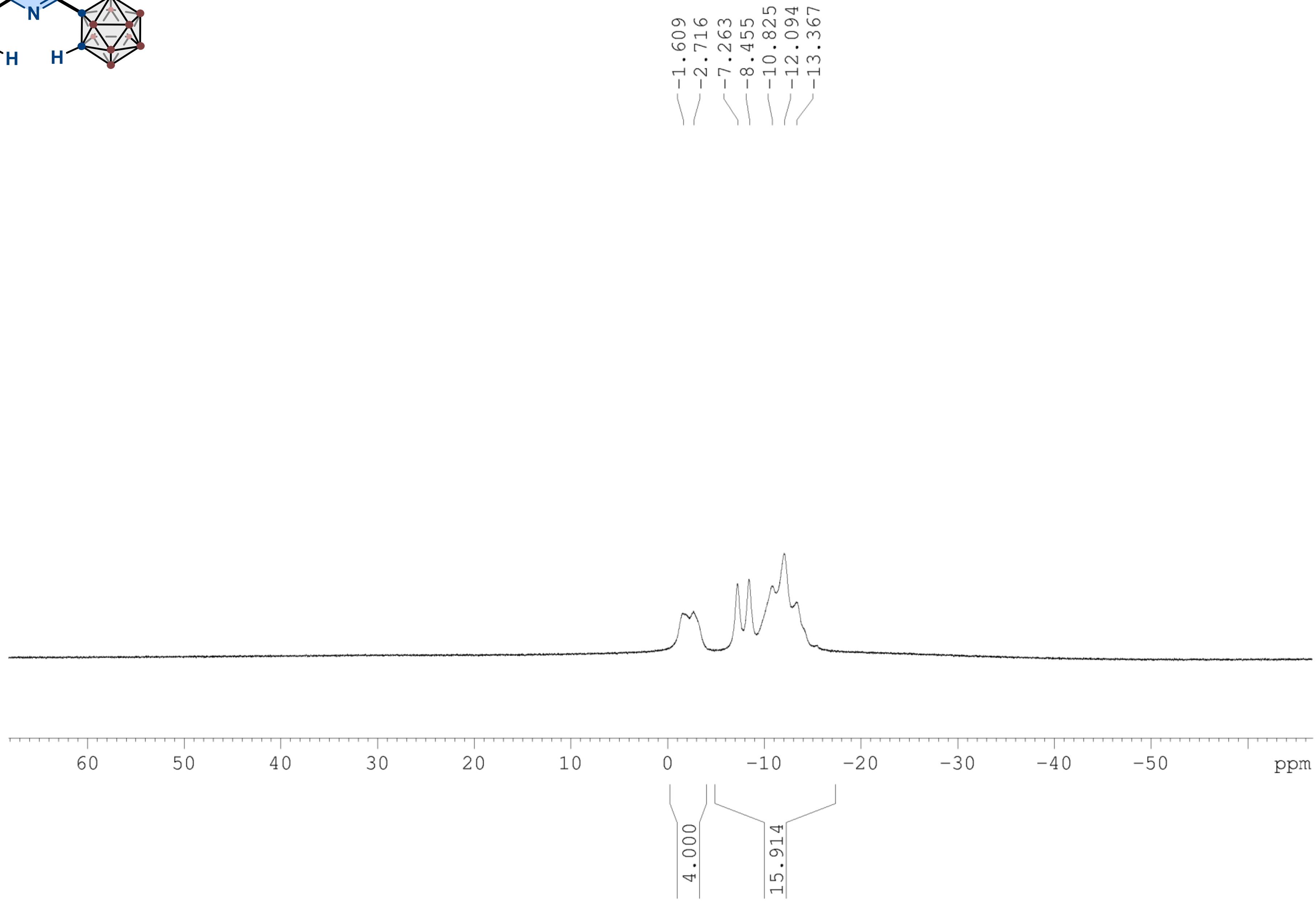

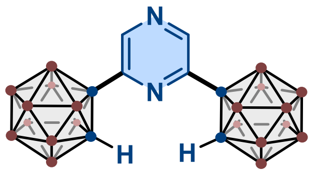

^{13}C NMR, (100 MHz, $CDCl_3$)

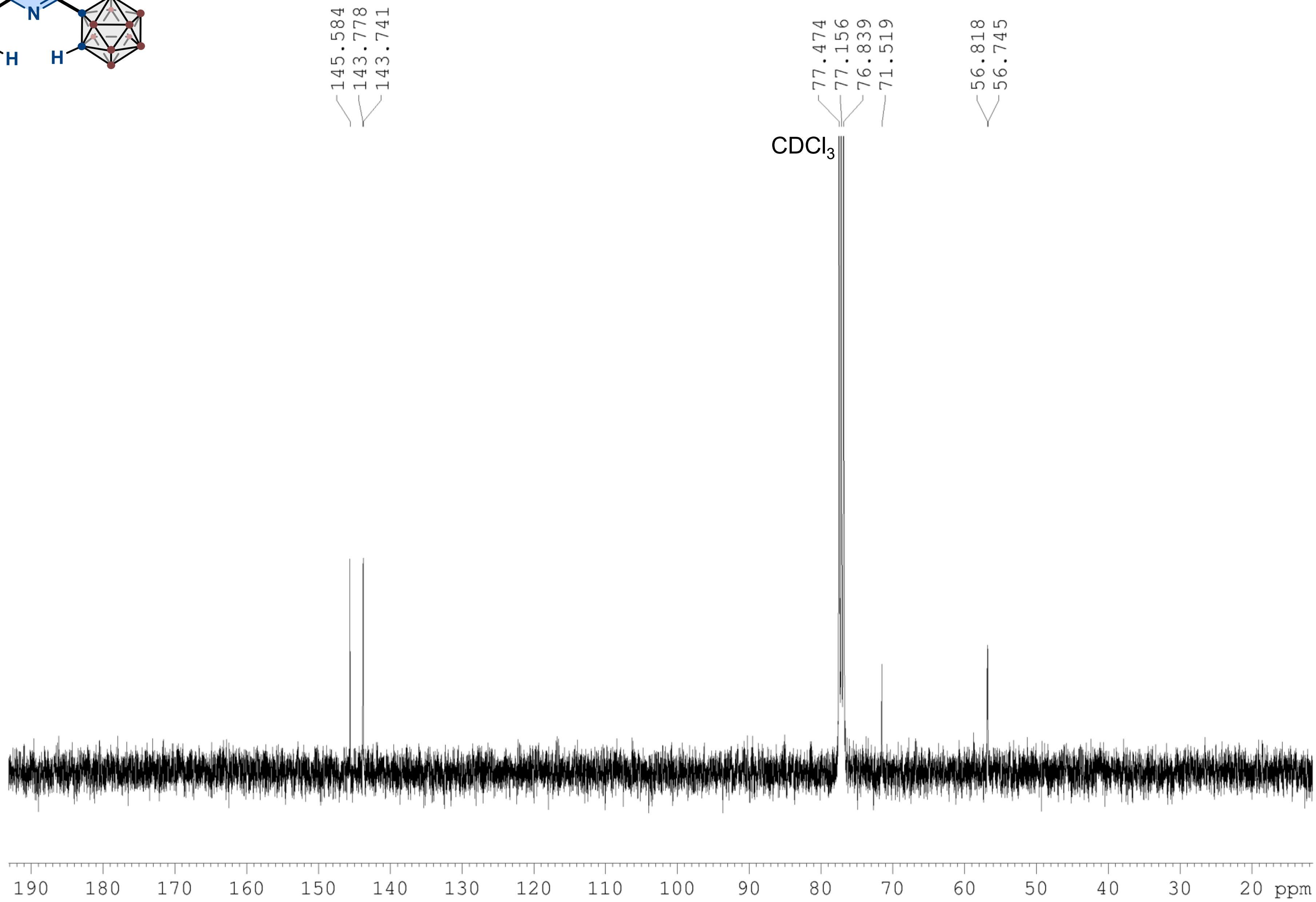

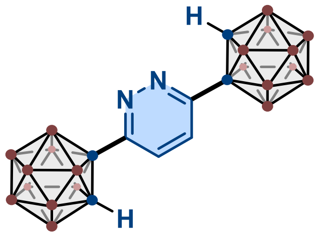

^1H NMR, (400 MHz, $CDCl_3$)

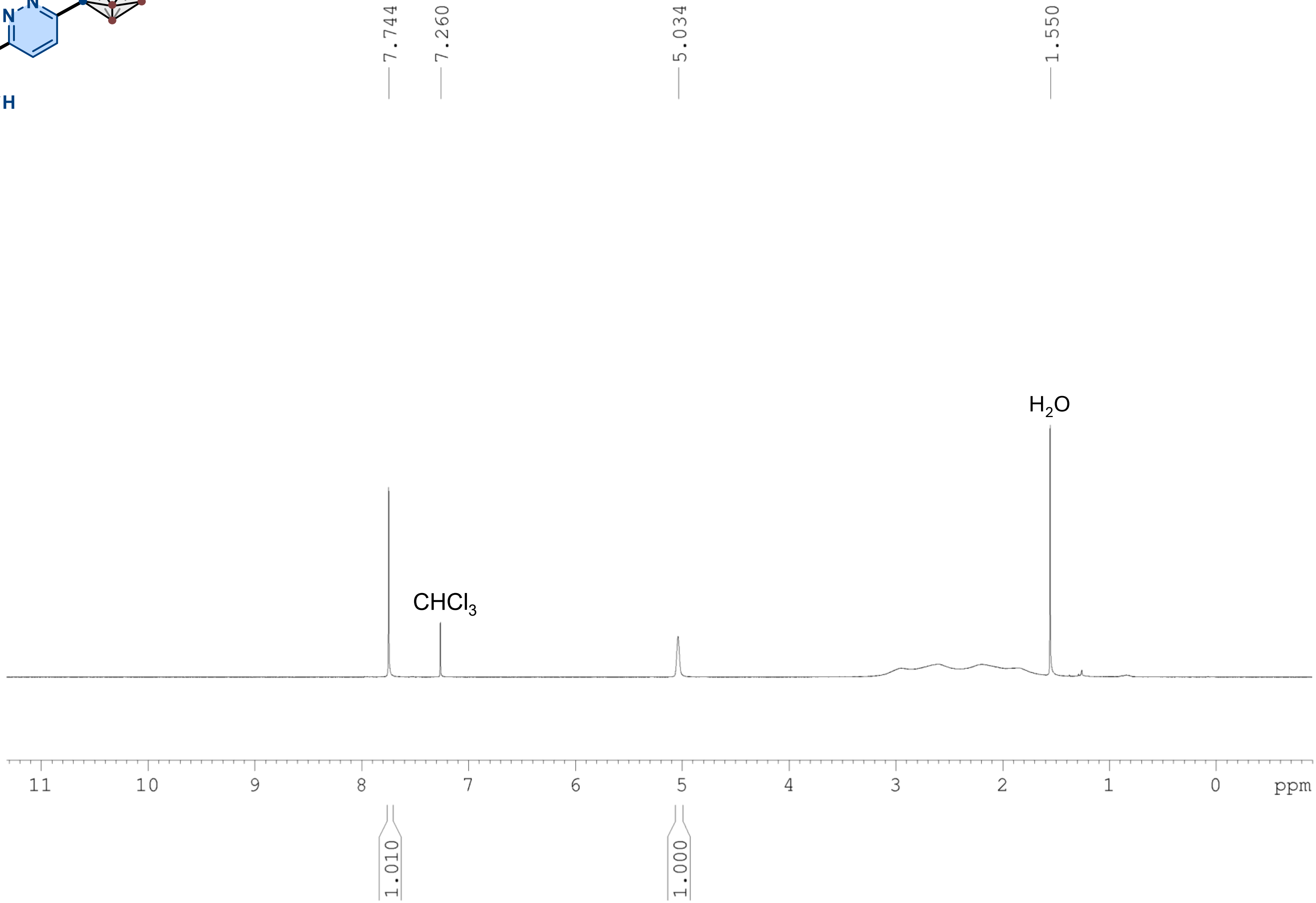

^{11}B NMR, (128 MHz, $CDCl_3$)

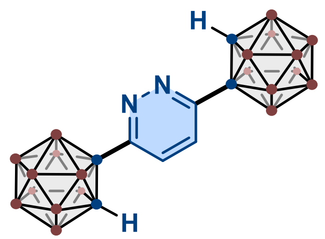

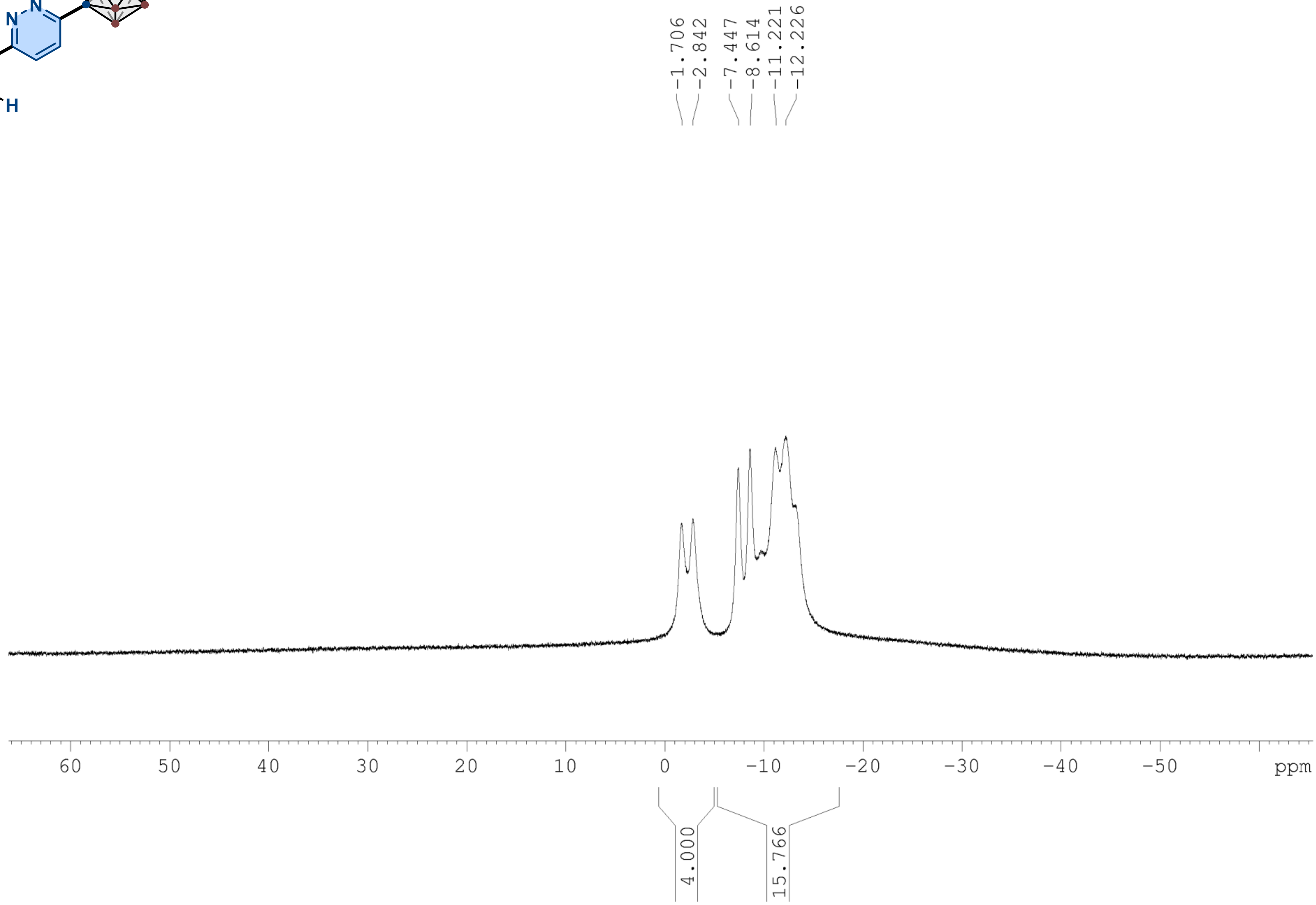

^{13}C NMR, (100 MHz, $CDCl_3$)

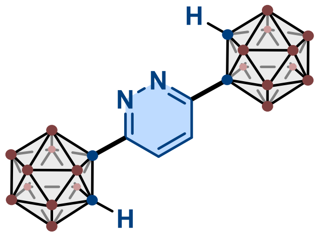

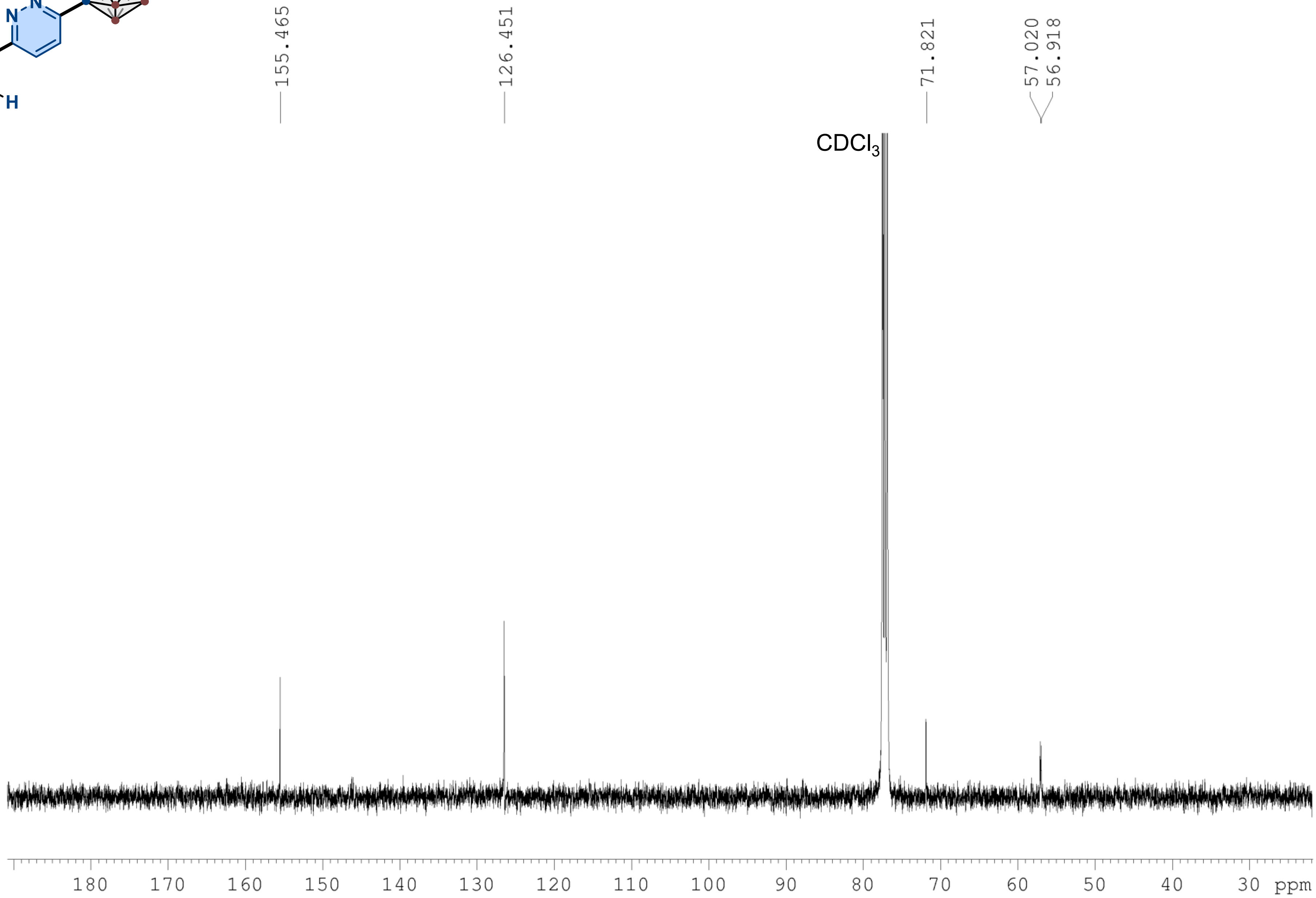

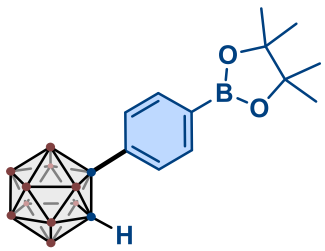

^{1}H NMR, (400 MHz, $CDCl_3$)

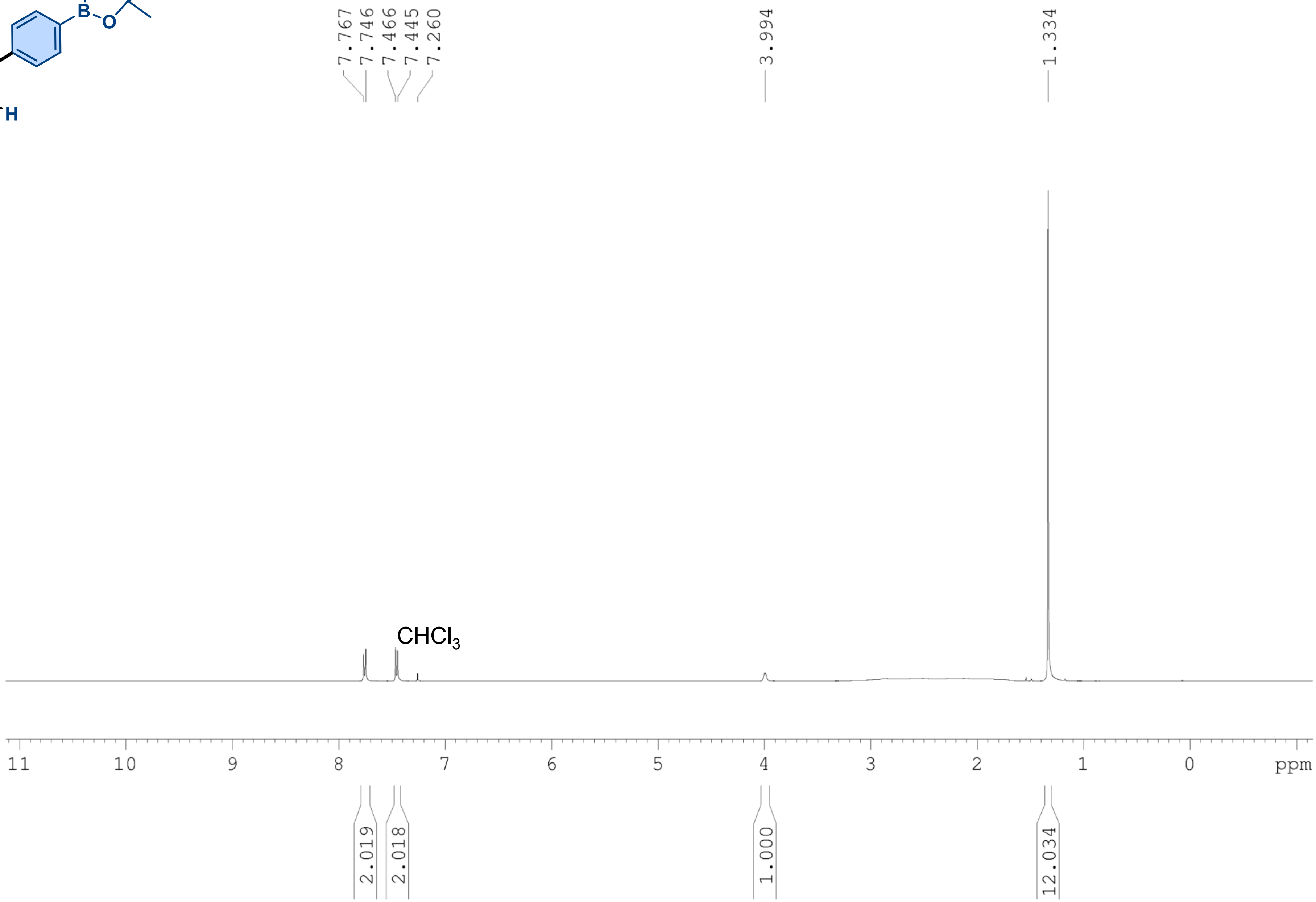

^{11}B NMR, (128 MHz, $CDCl_3$)

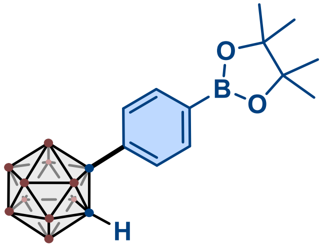

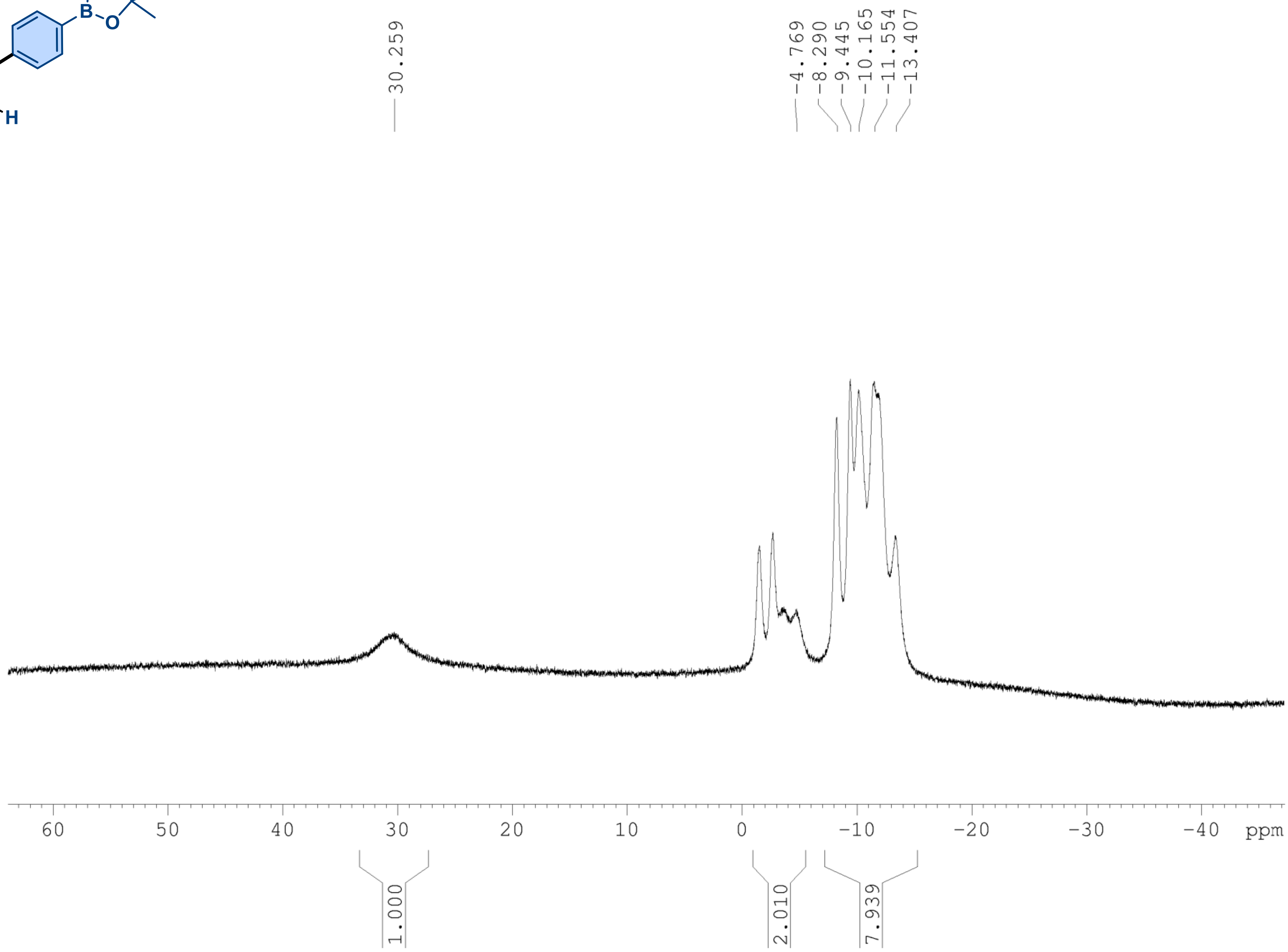

^{13}C NMR, (100 MHz, $CDCl_3$)

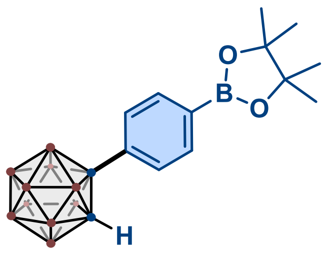

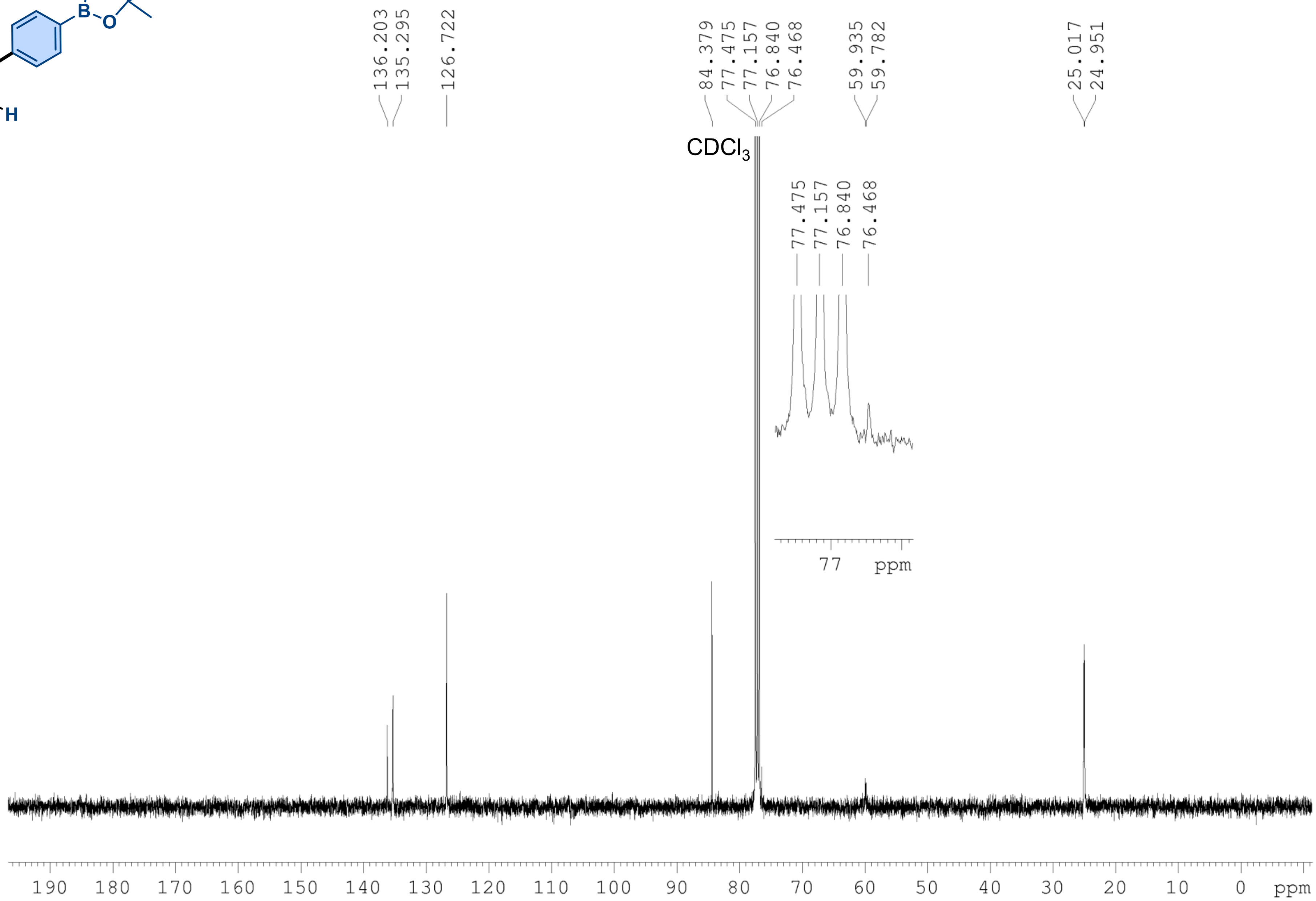

^{1}H NMR, (400 MHz, $CDCl_3$)

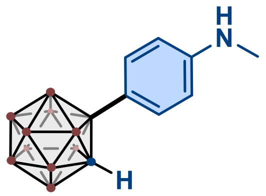

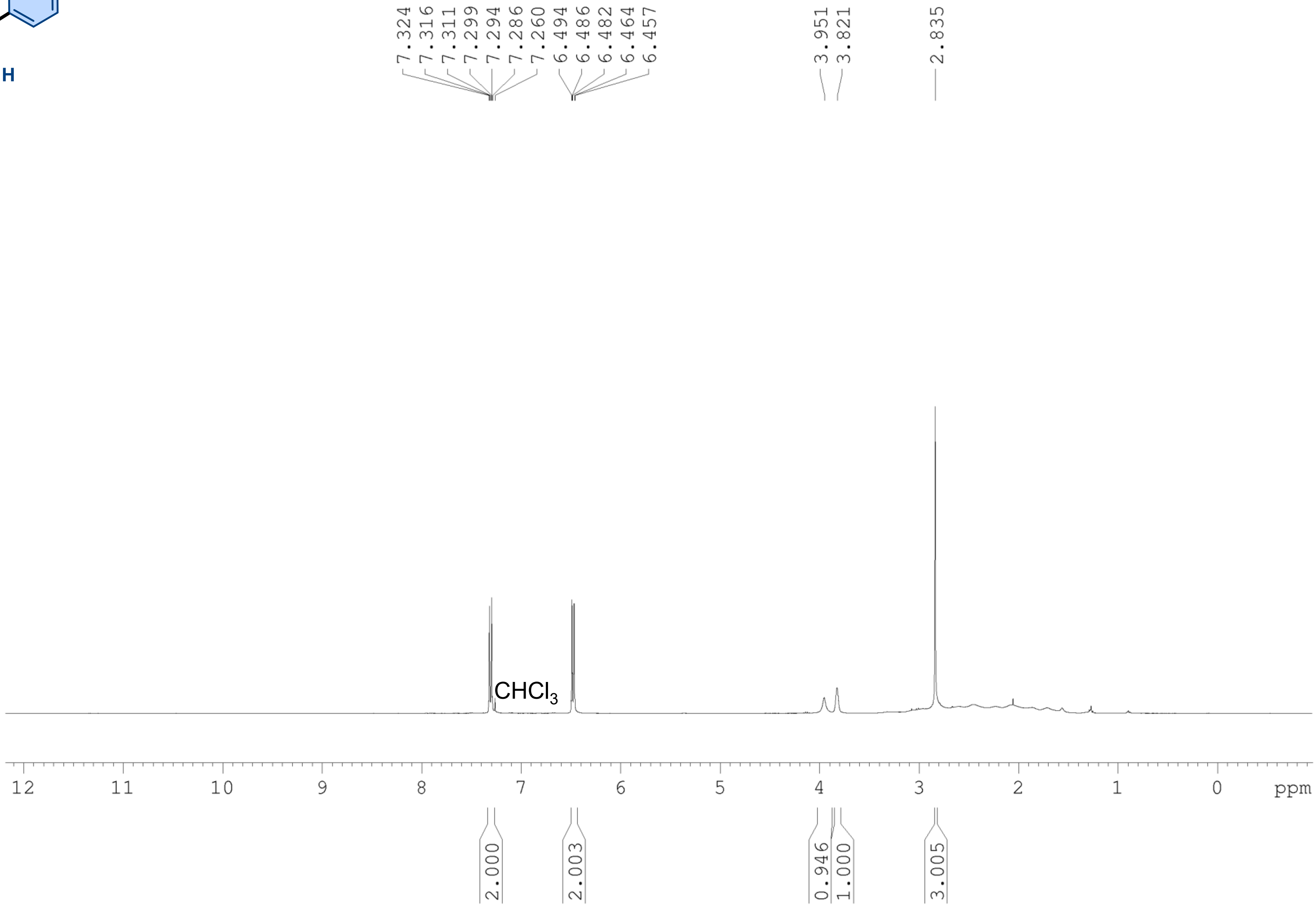

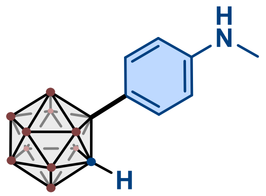

^{11}B NMR, (128 MHz, $CDCl_3$)

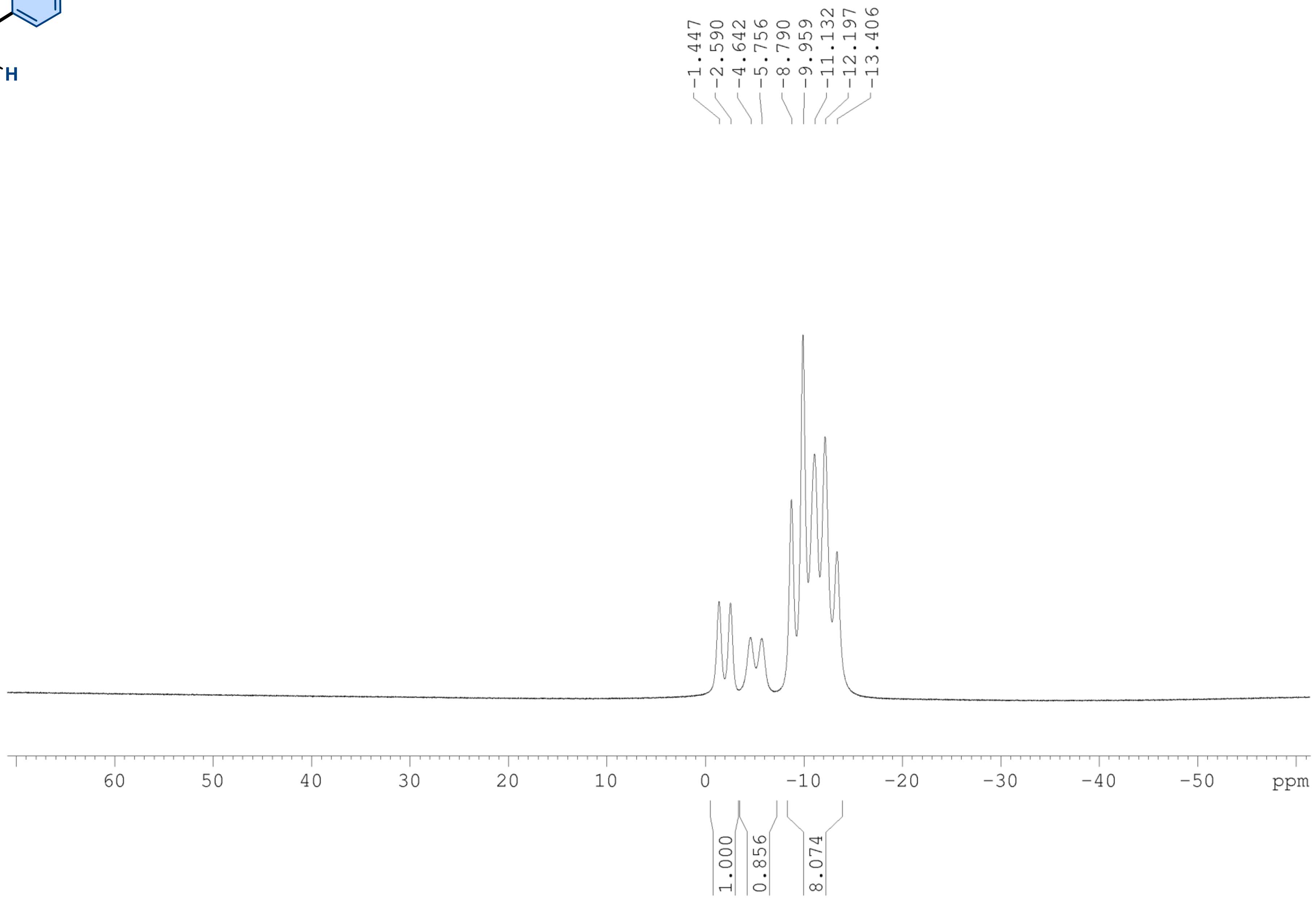

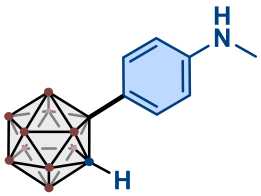

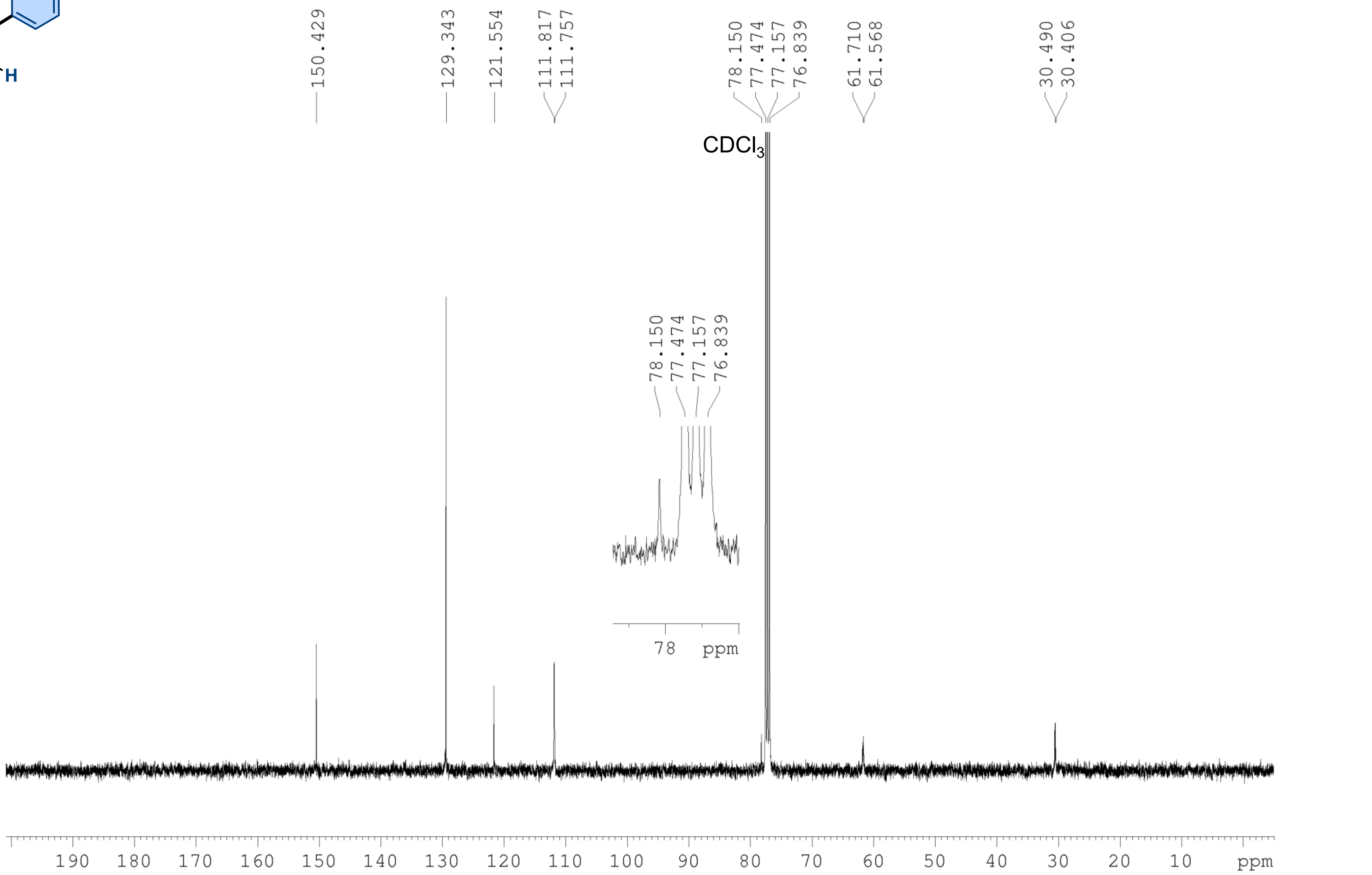

^{13}C NMR, (100 MHz, CDCl$_3$)
150.429
129.343
121.554
111.817
111.757
78.150
77.474
77.157
76.839
61.710
61.568
30.490
30.406
CDCl$_3$
78
ppm
190
180
170
160
150
140
130
120
110
100
90
80
70
60
50
40
30
20
10
ppm

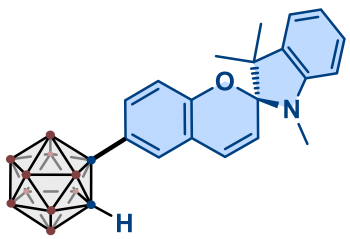

^{1}H NMR, (400 MHz, $CDCl_3$)

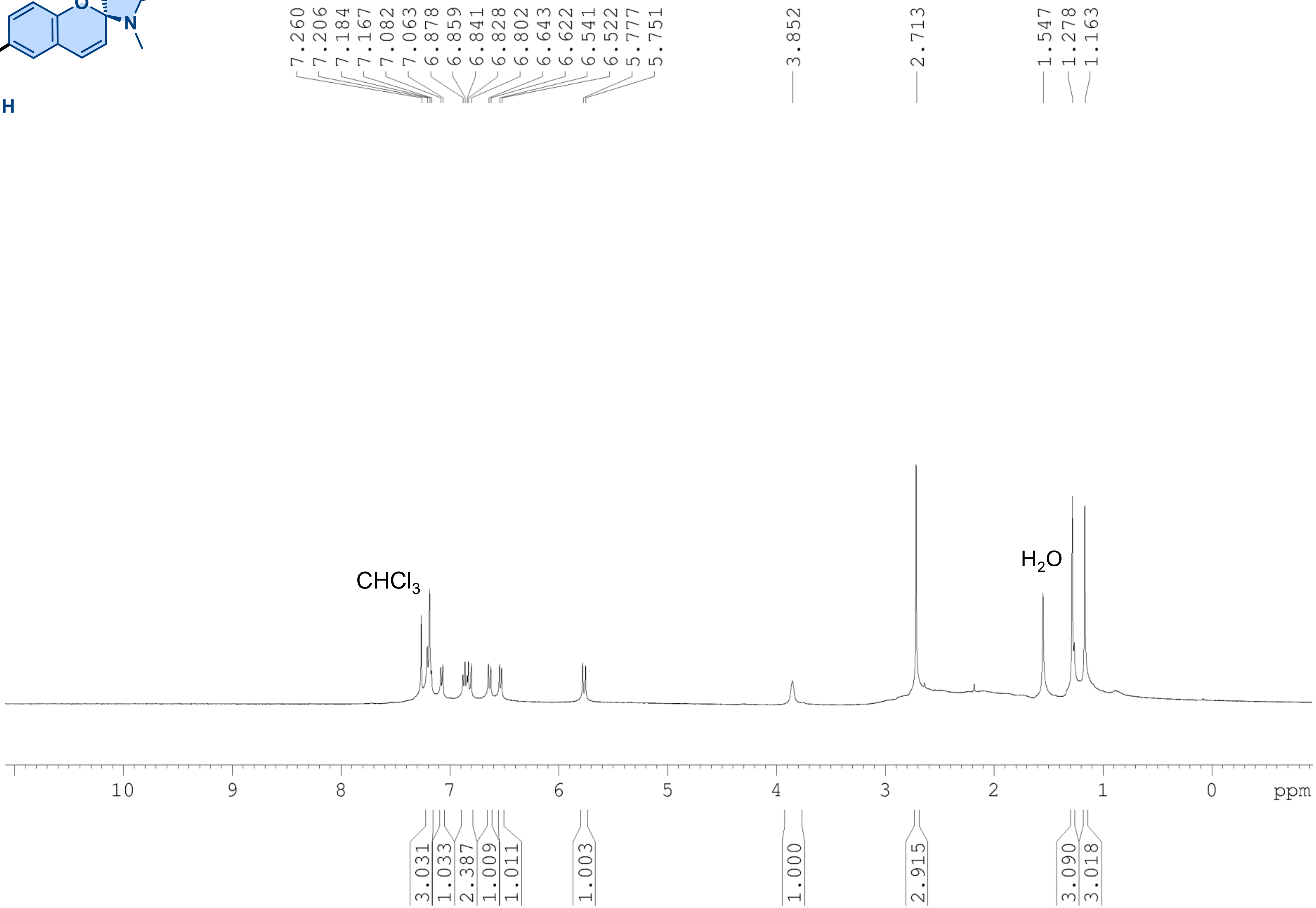

^{11}B NMR, (128 MHz, $CDCl_3$)

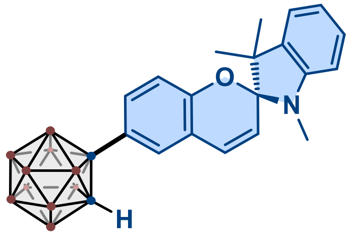

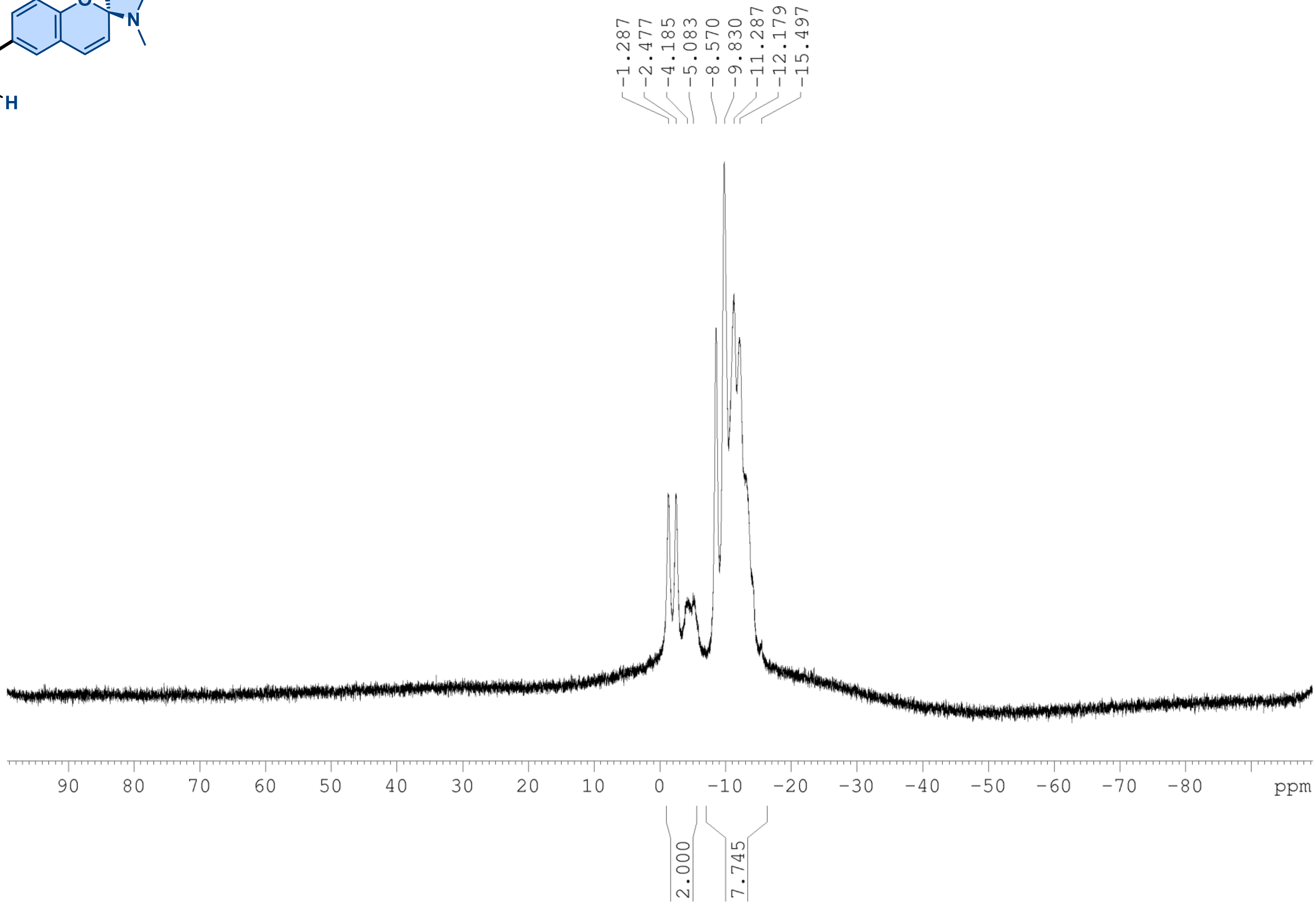

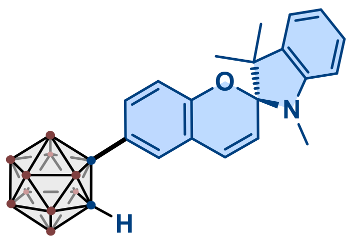

^{13}C NMR, (100 MHz, $CDCl_3$)

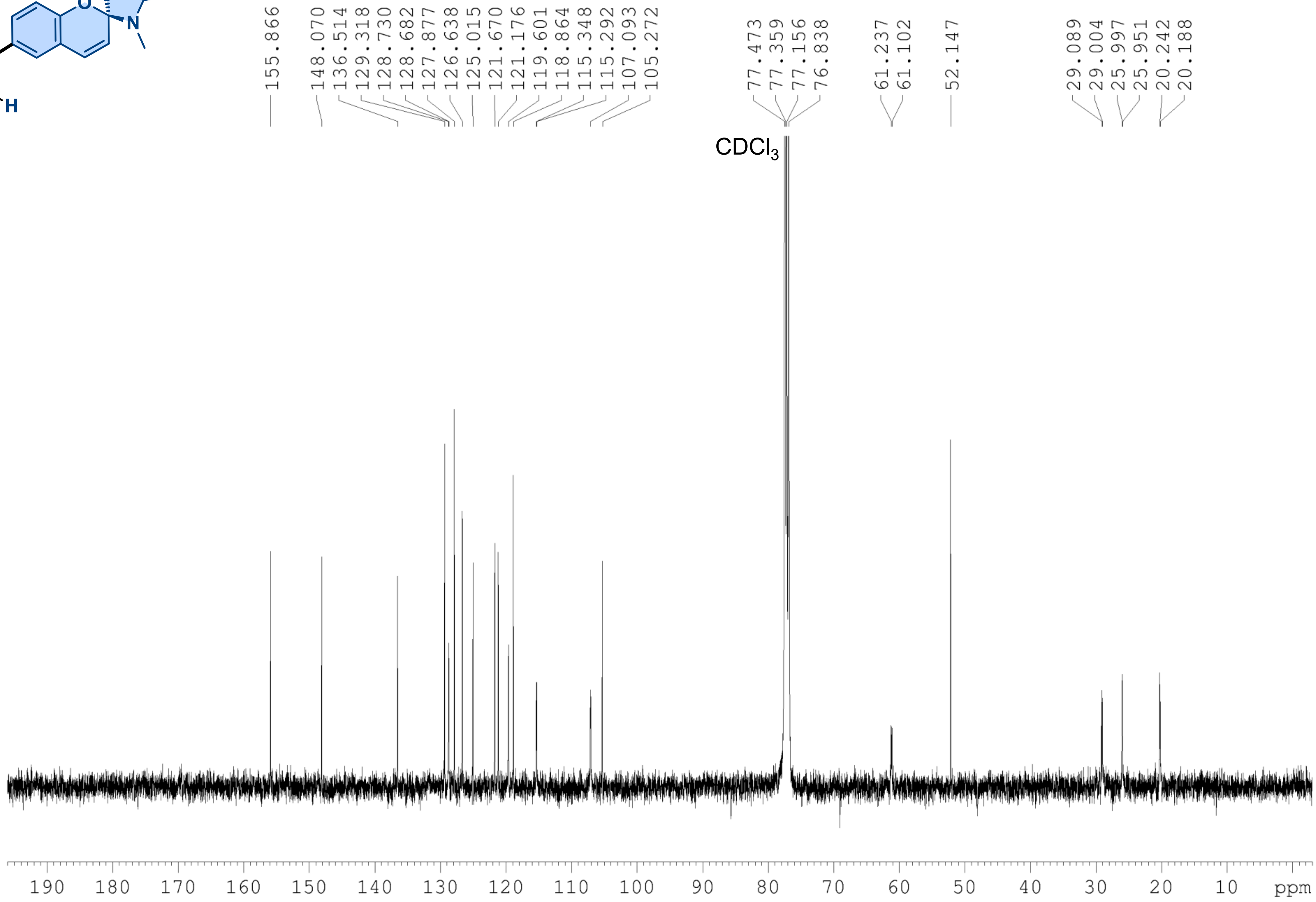

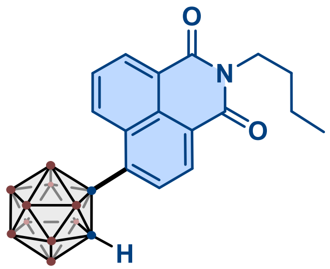

^{1}H NMR, (400 MHz, $CDCl_3$)

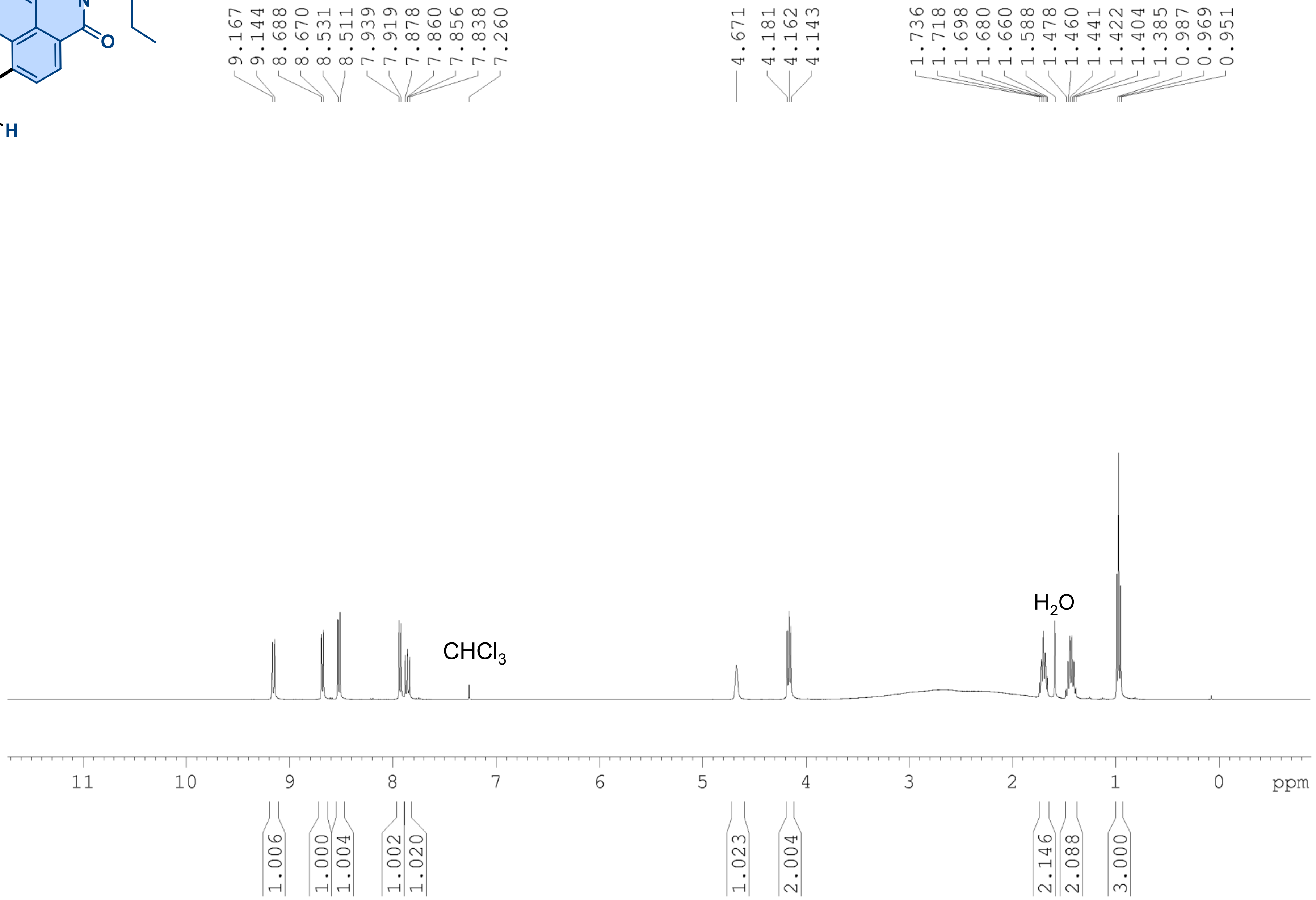

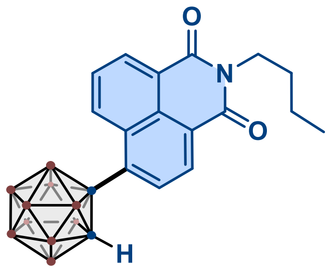

^{11}B NMR, (128 MHz, $CDCl_3$)

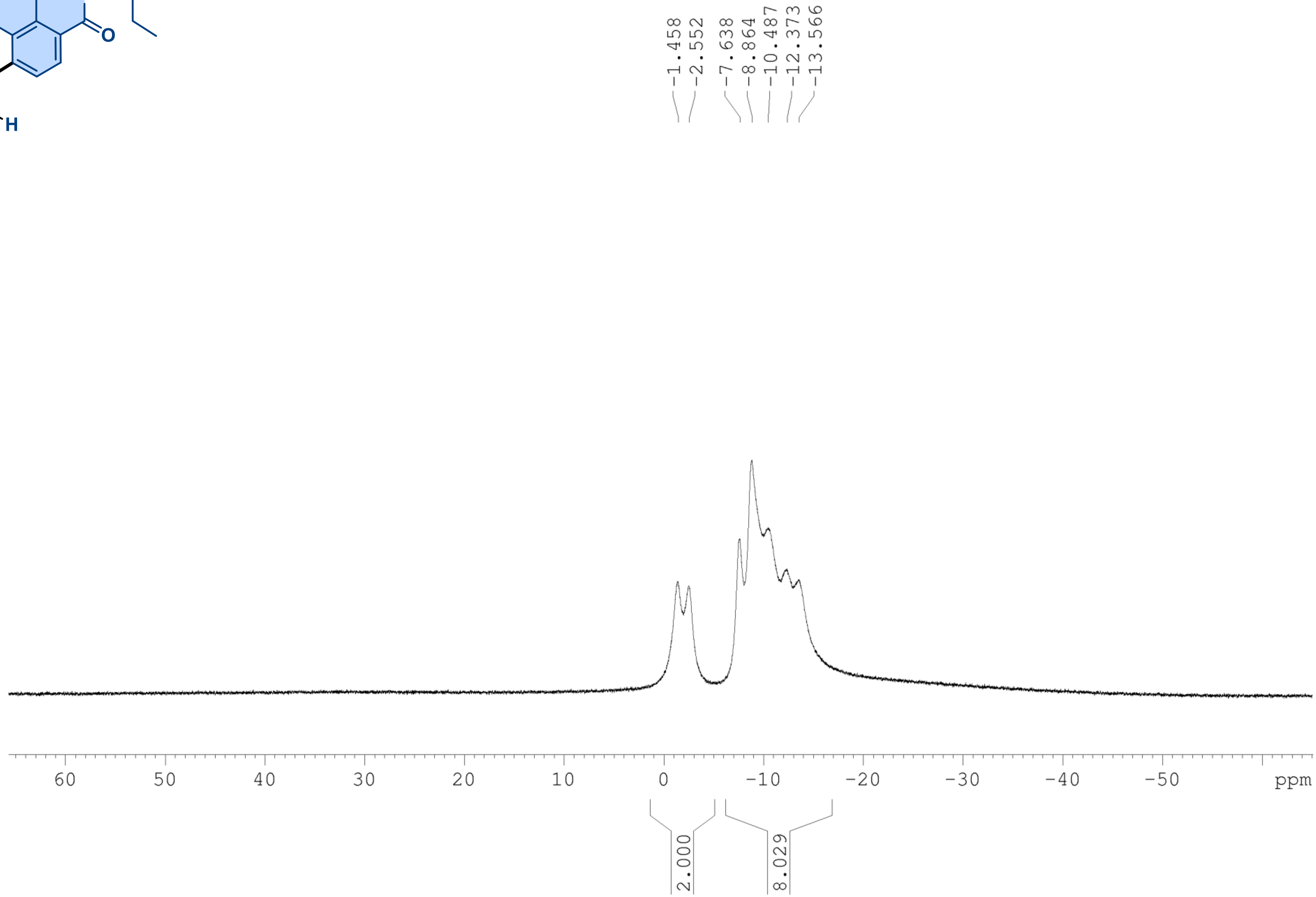

^{13}C NMR, (100 MHz, $CDCl_3$)

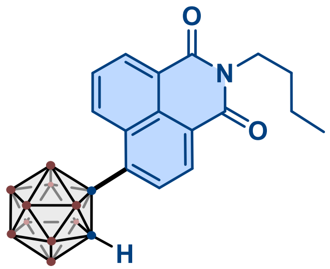

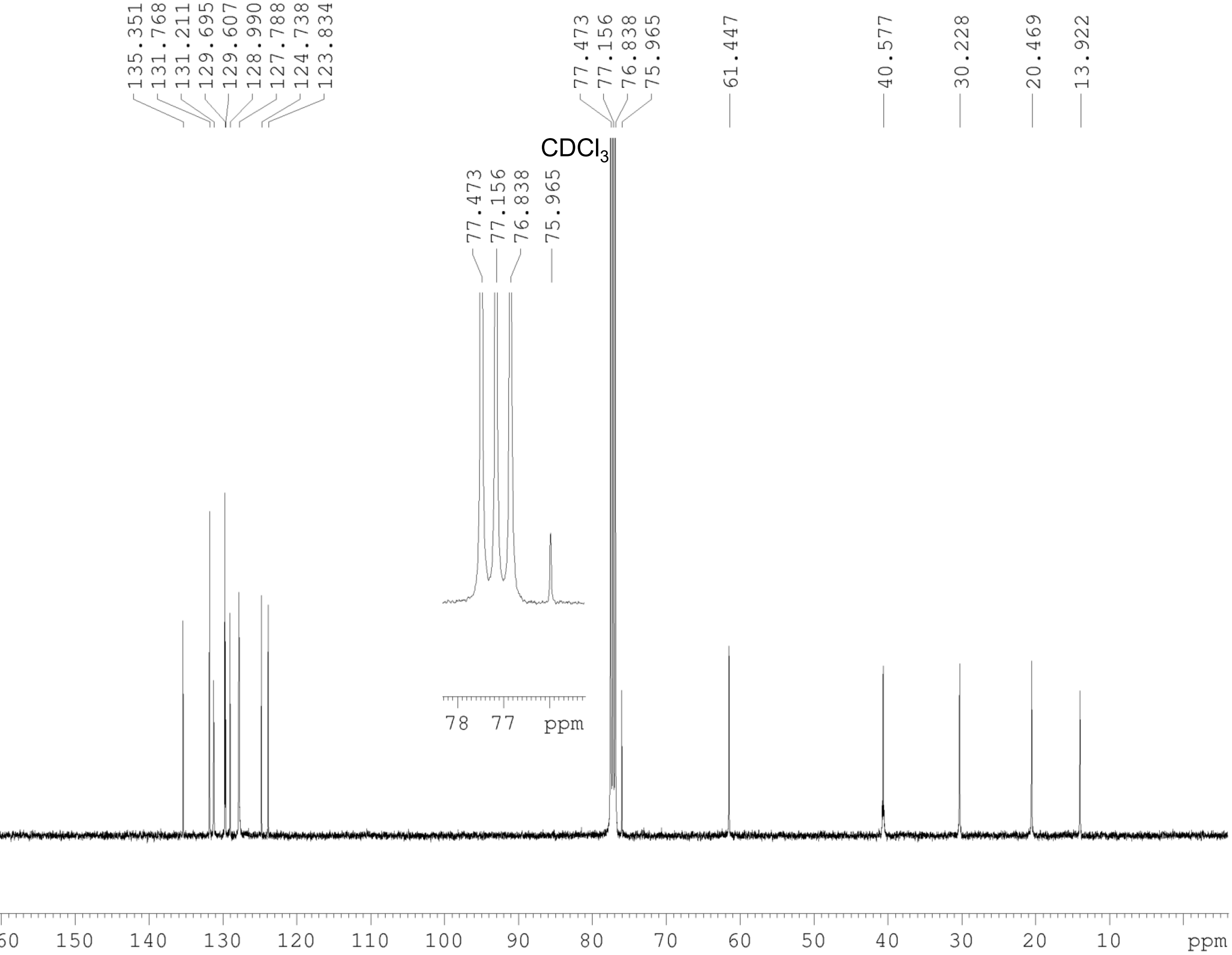

^{1}H NMR, (400 MHz, $CDCl_3$)

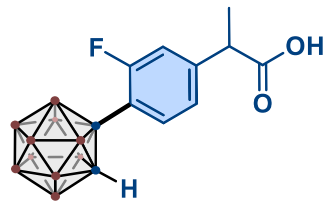

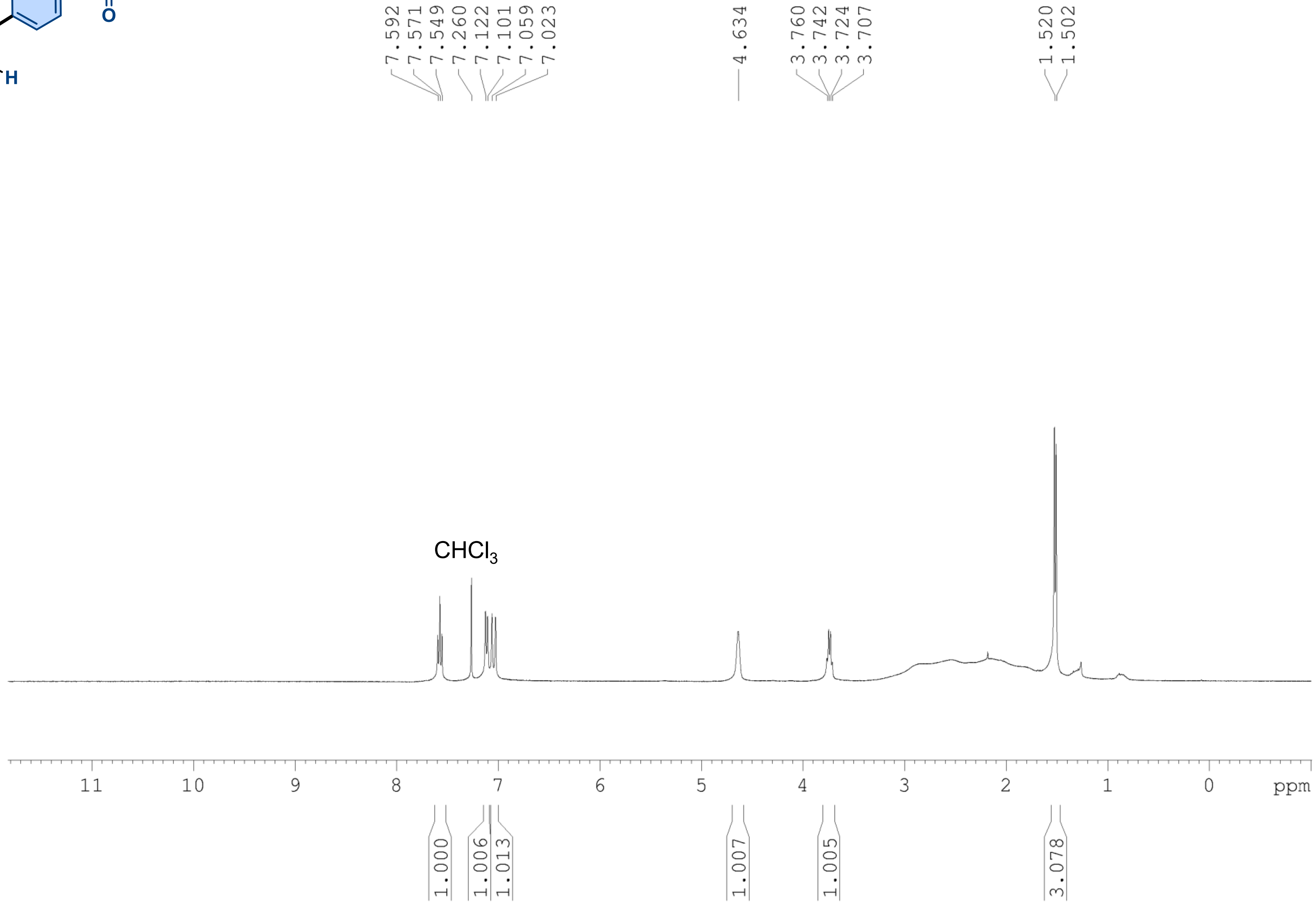

^{11}B NMR, (128 MHz, CDCl$_3$)

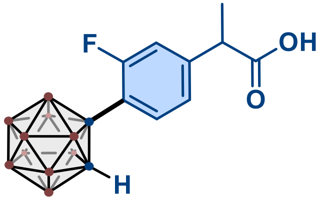

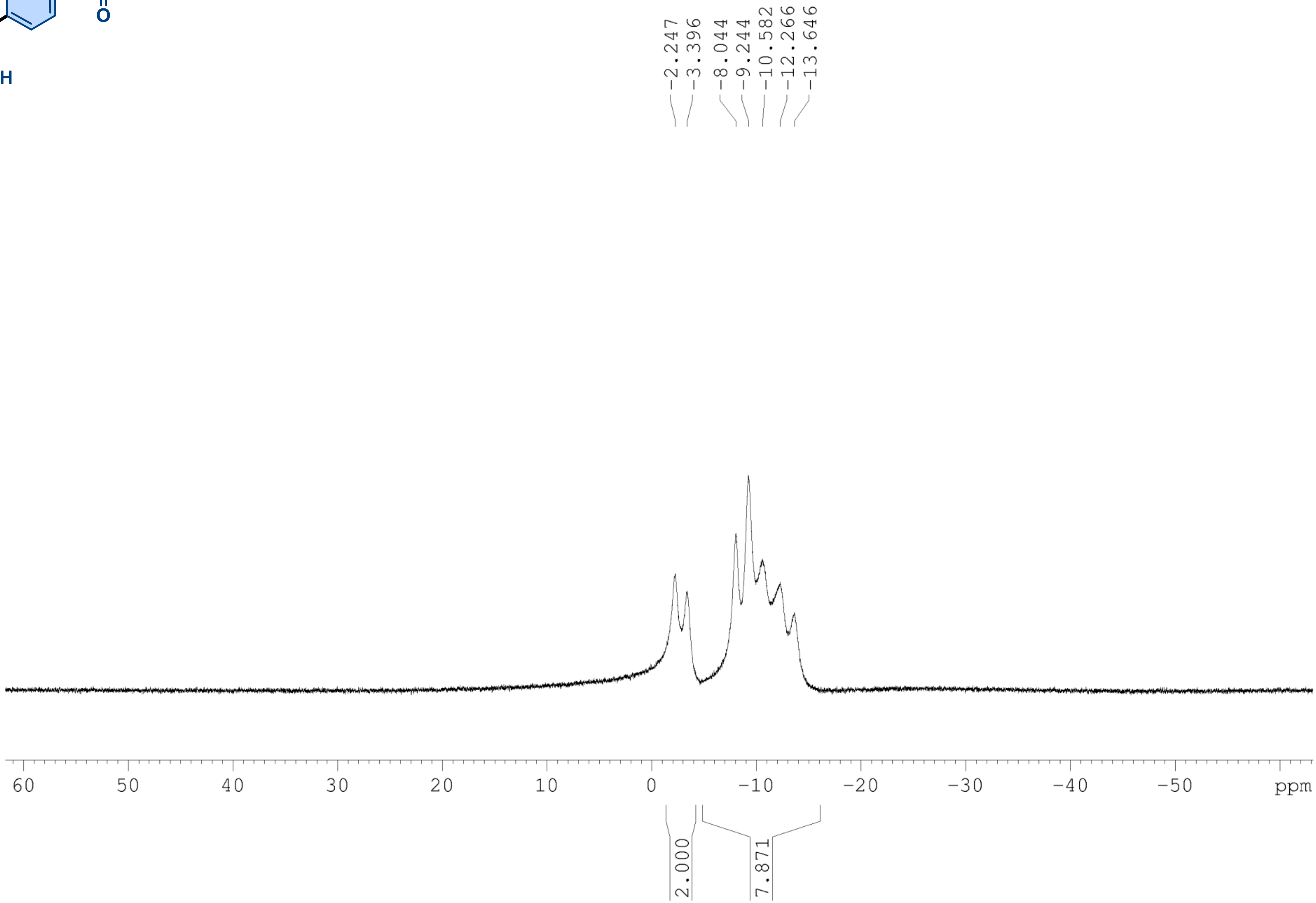

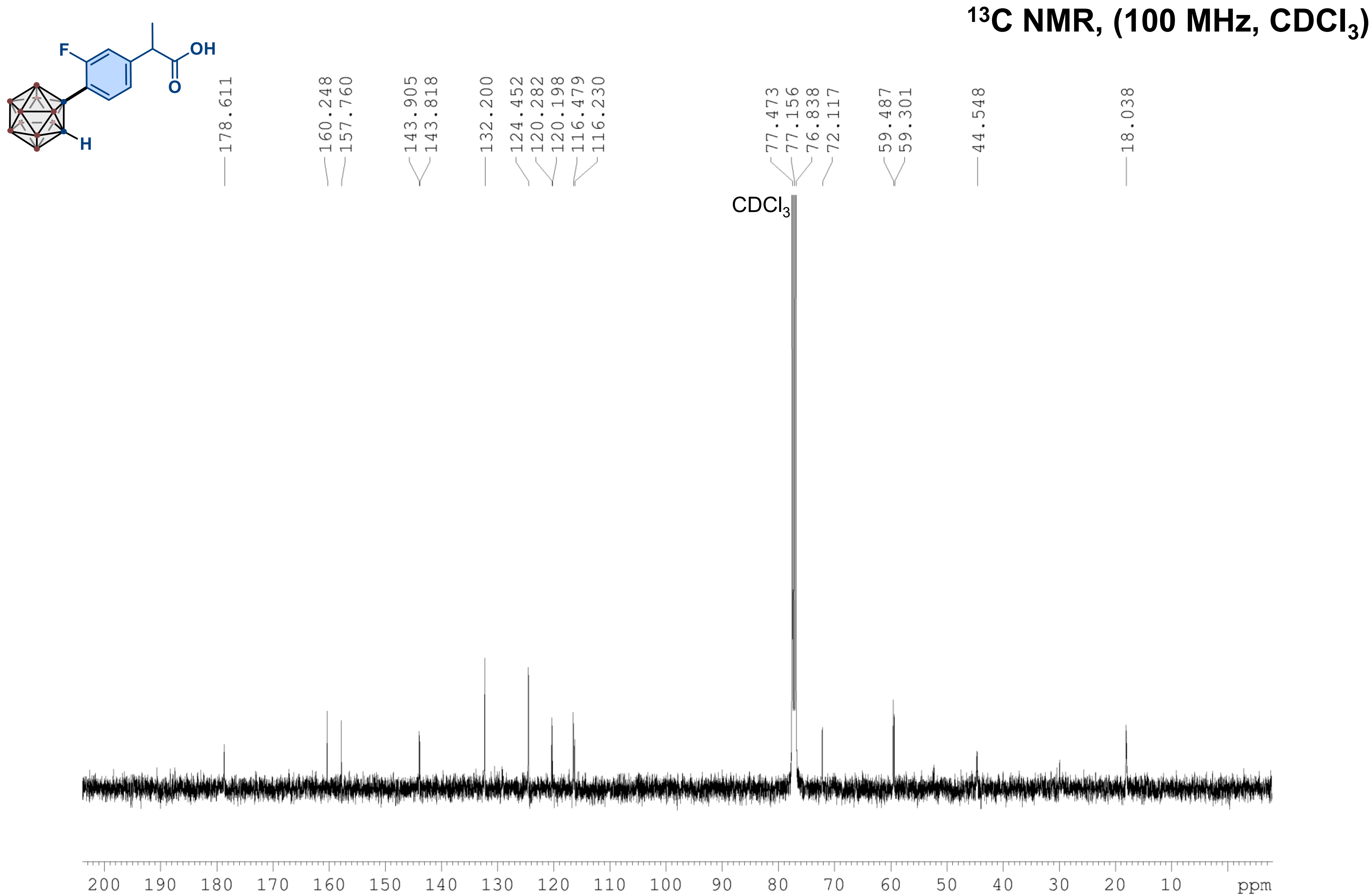

^{13}C NMR, (100 MHz, CDCl$_3$)
F
OH
O
H
178.611
160.248
157.760
143.905
143.818
132.200
124.452
120.282
120.198
116.479
116.230
77.473
77.156
76.838
72.117
59.487
59.301
44.548
18.038
CDCl$_3$
200 190 180 170 160 150 140 130 120 110 100 90 80 70 60 50 40 30 20 10 ppm

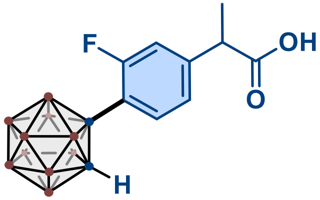

^{19}F NMR, (376 MHz, $CDCl_3$)

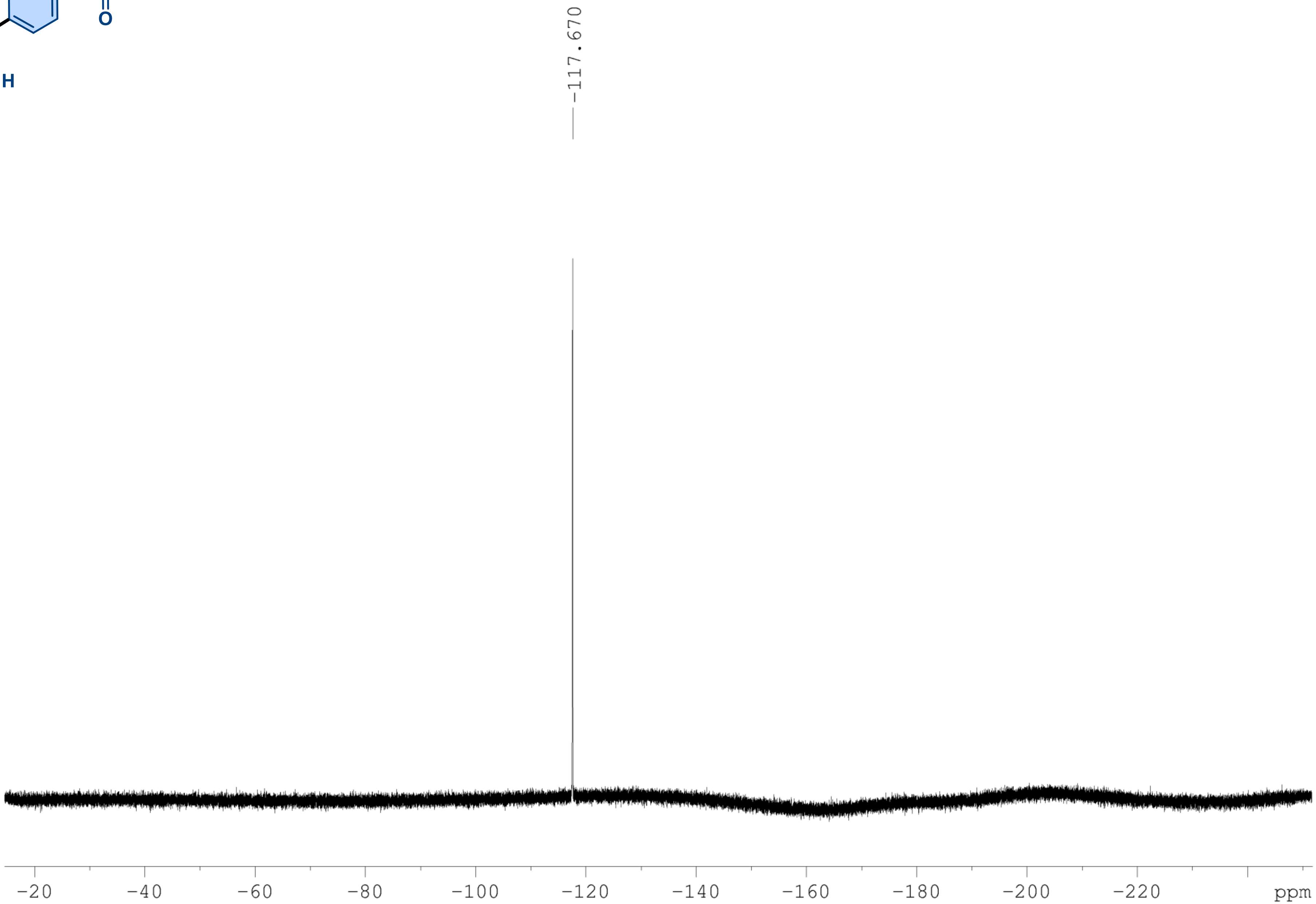

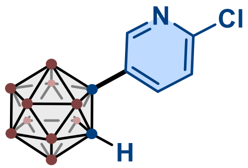

^{1}H NMR, (400 MHz, $CDCl_3$)

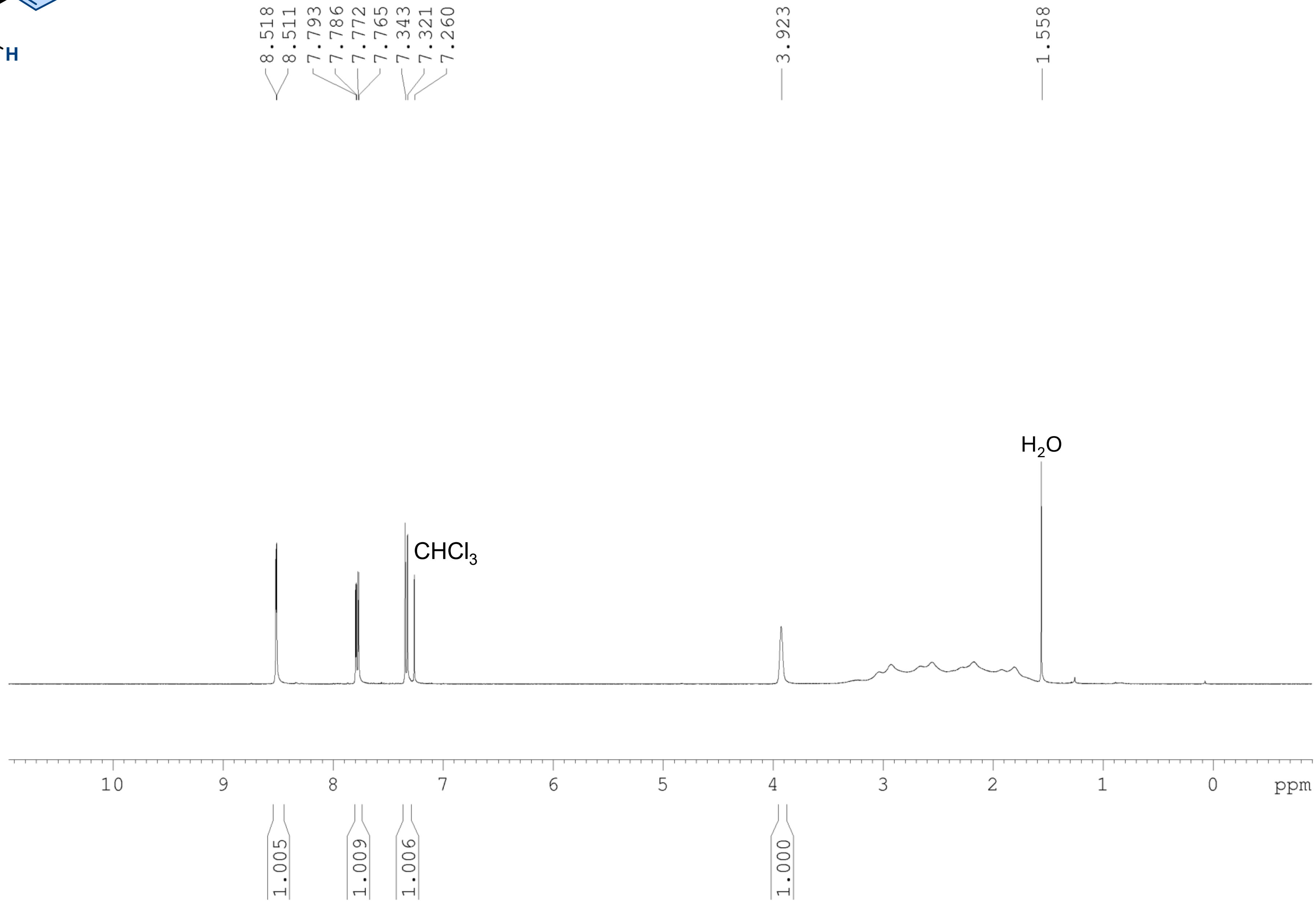

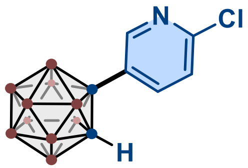

^{11}B NMR, (128 MHz, $CDCl_3$)

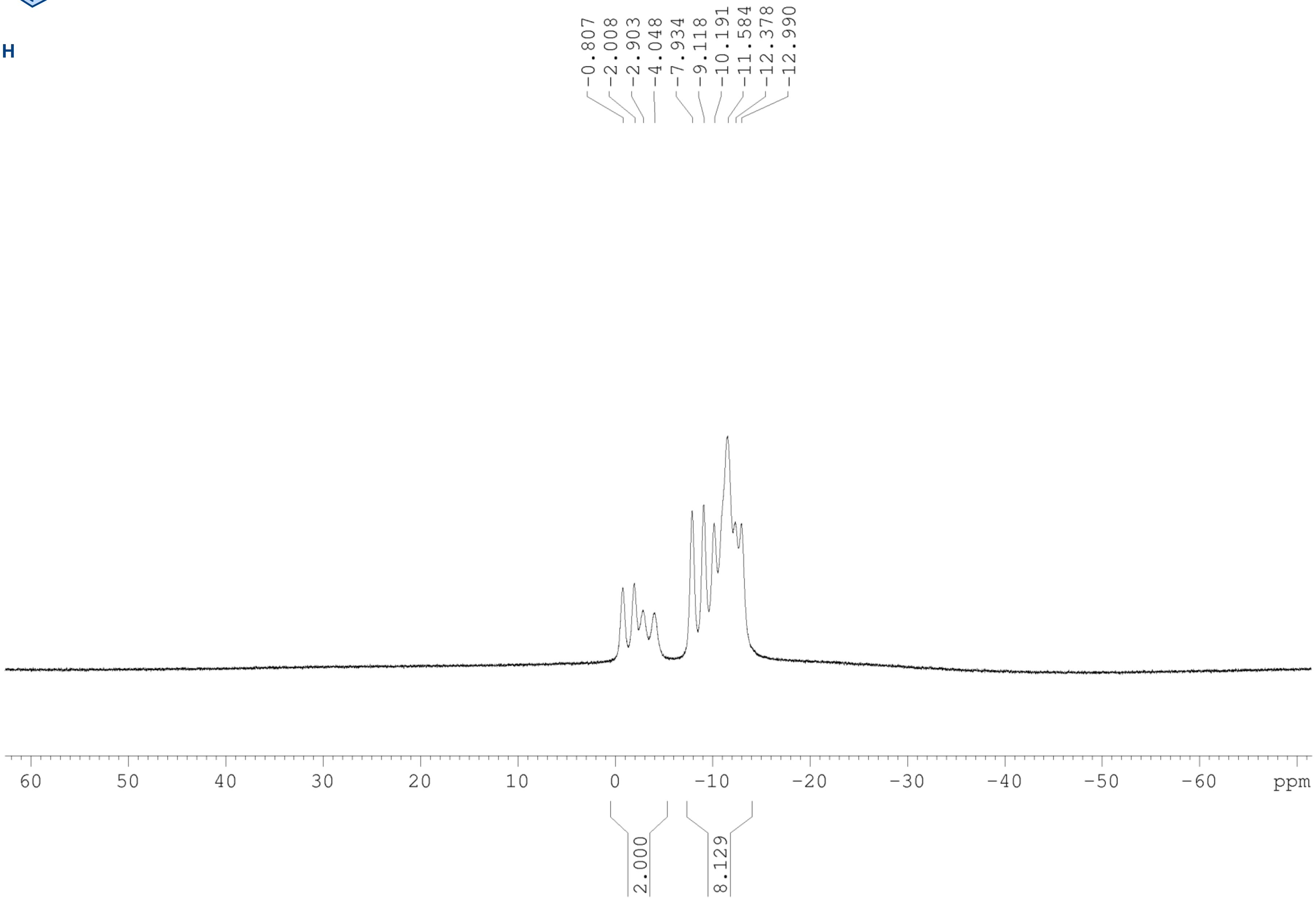

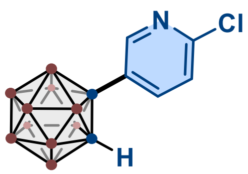

^{13}C NMR, (100 MHz, $CDCl_3$)

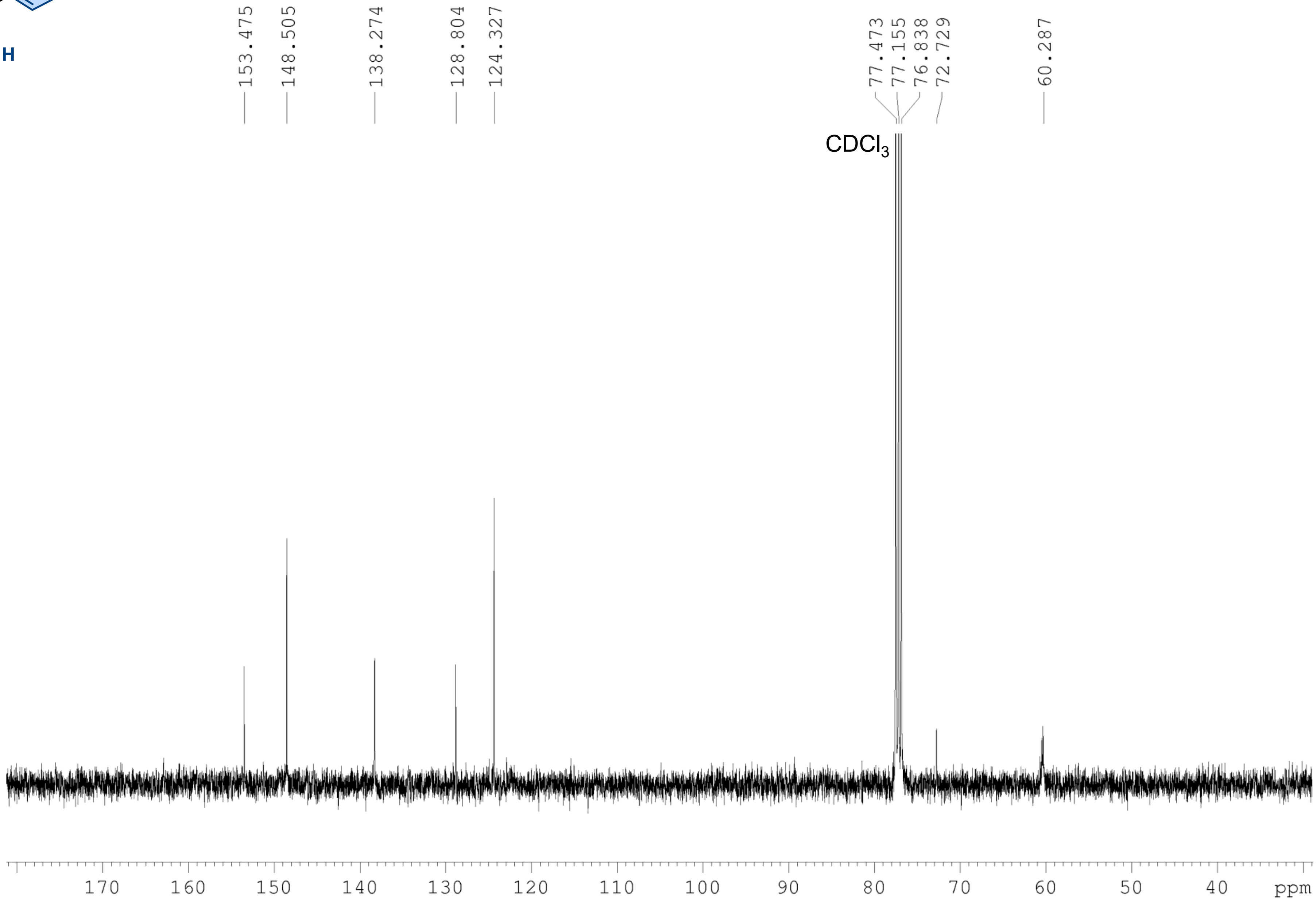

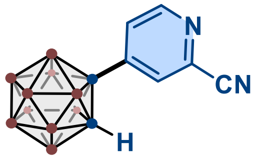

^1H NMR, (400 MHz, $CDCl_3$)

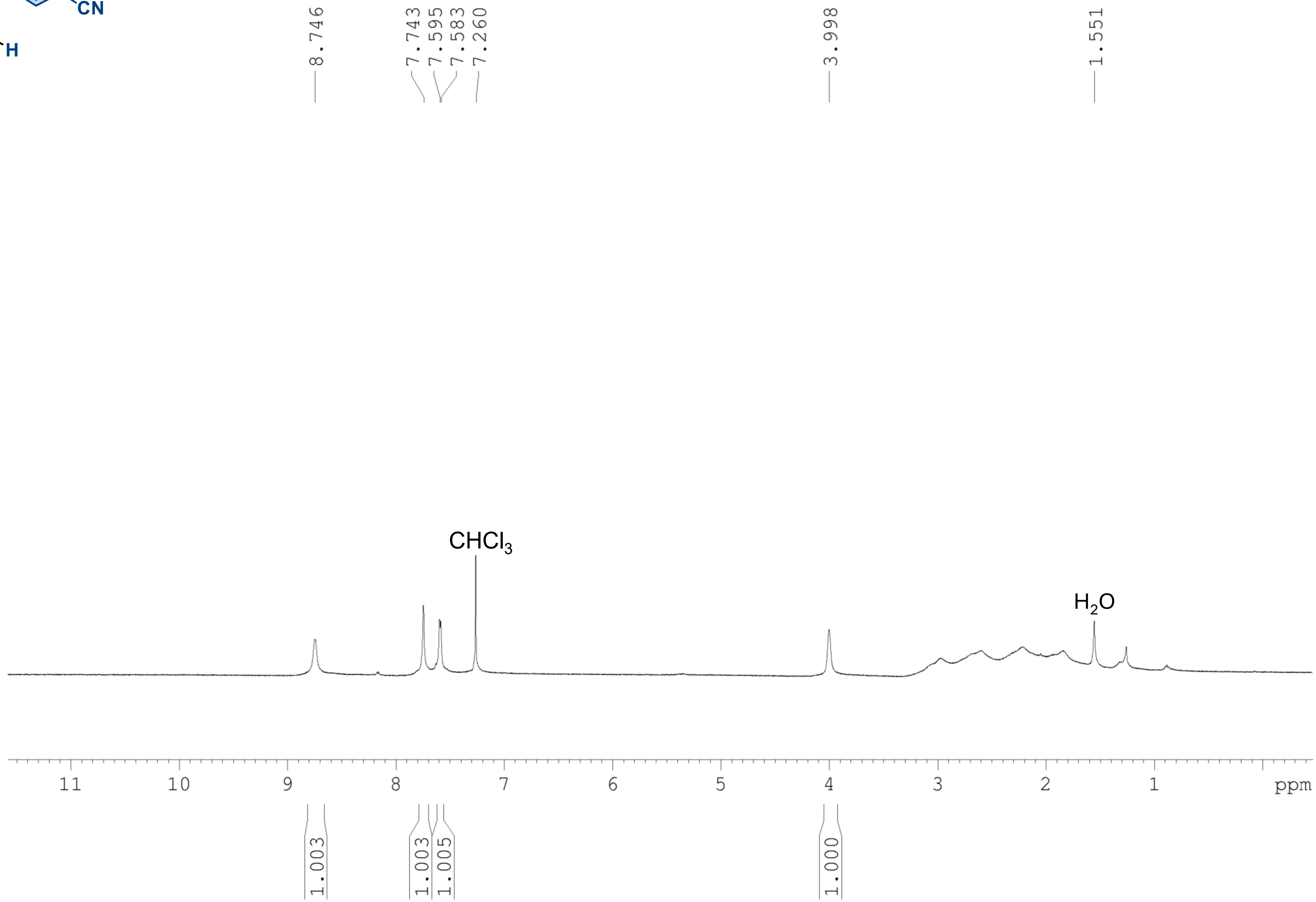

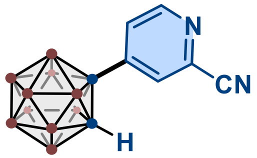

^{11}B NMR, (128 MHz, $CDCl_3$)

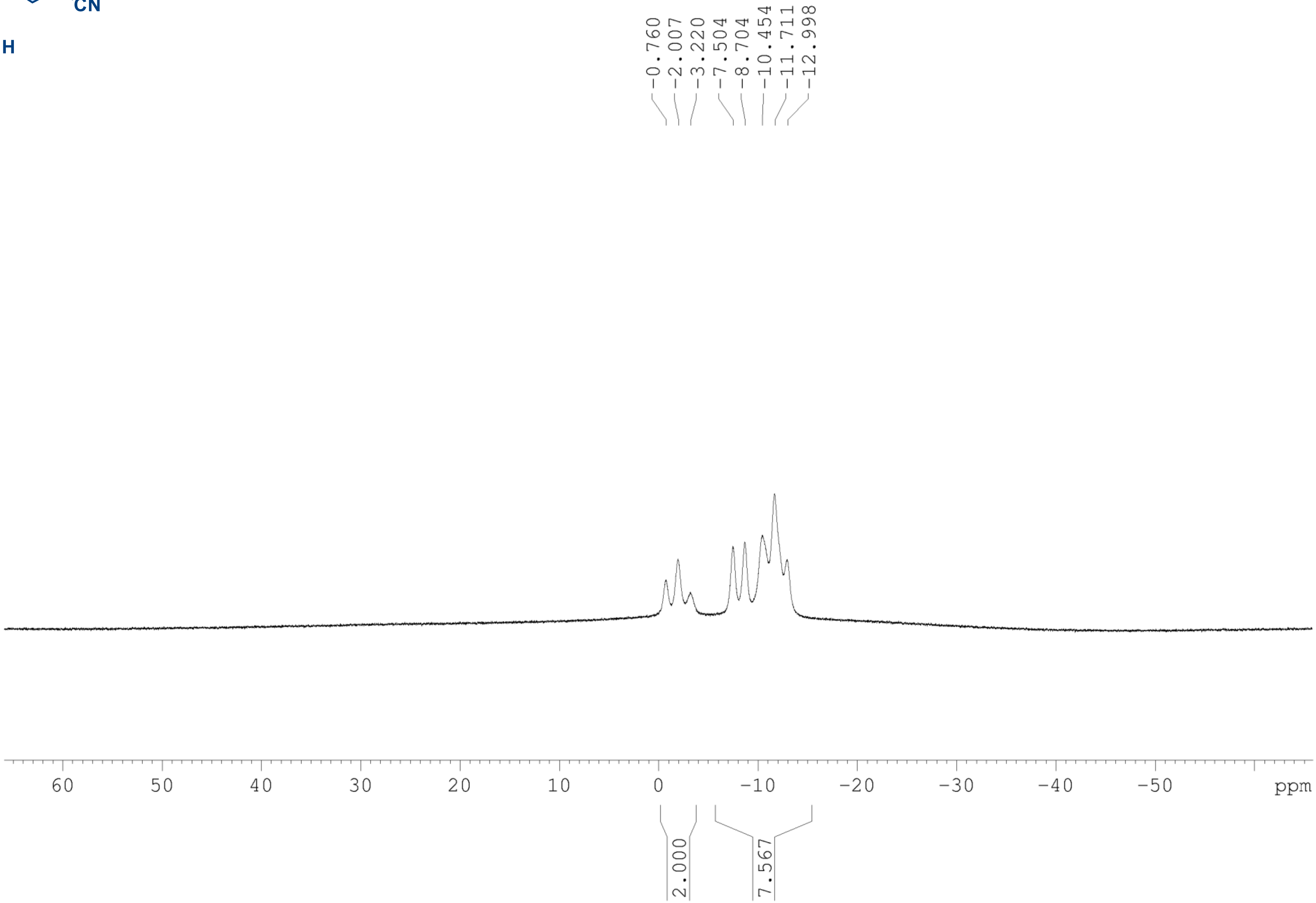

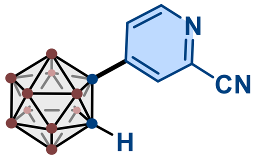

^{13}C NMR, (100 MHz, $CDCl_3$)

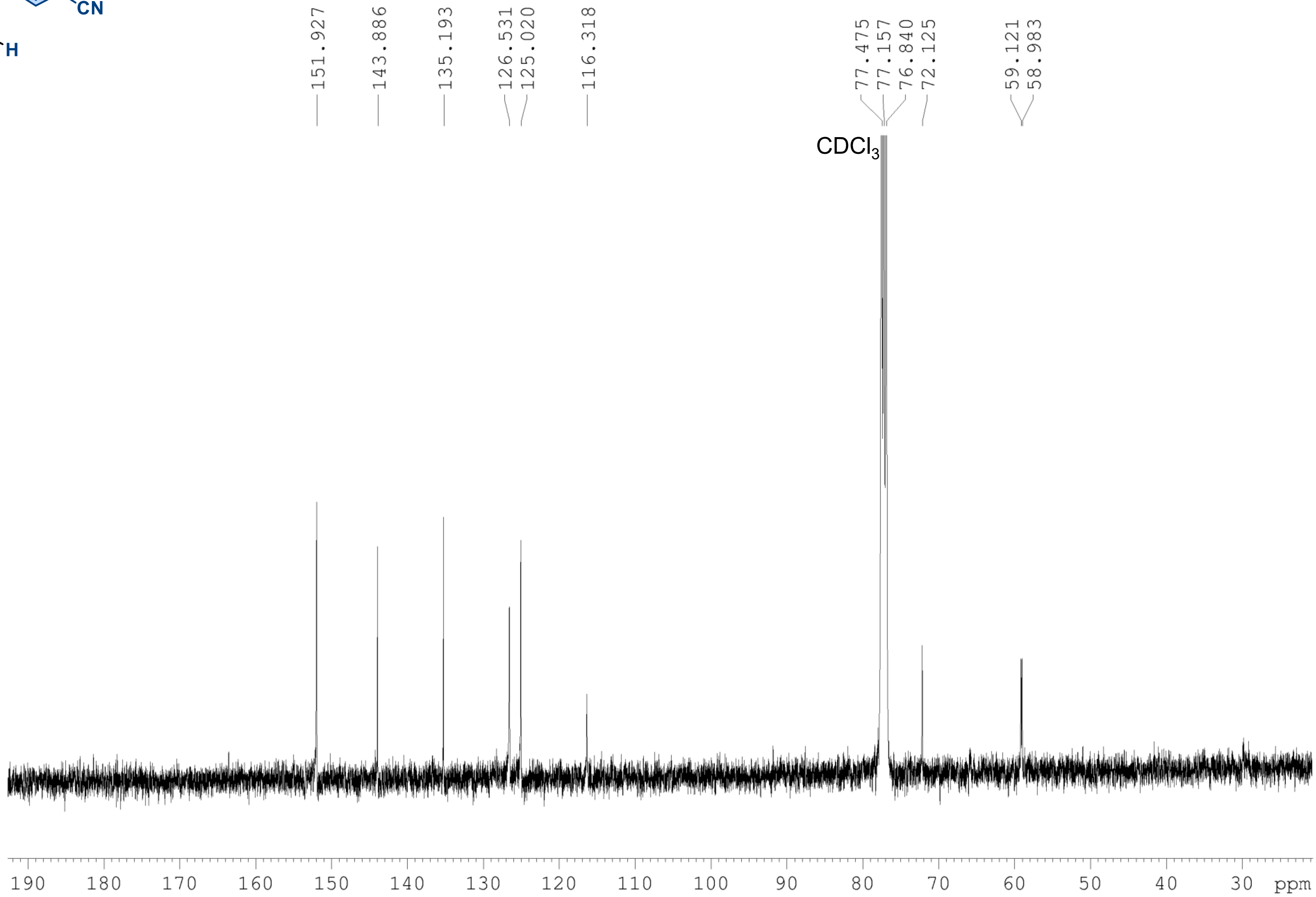

^1H NMR, (400 MHz, $CDCl_3$)

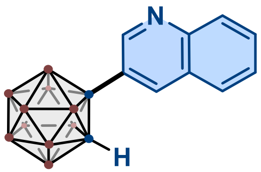

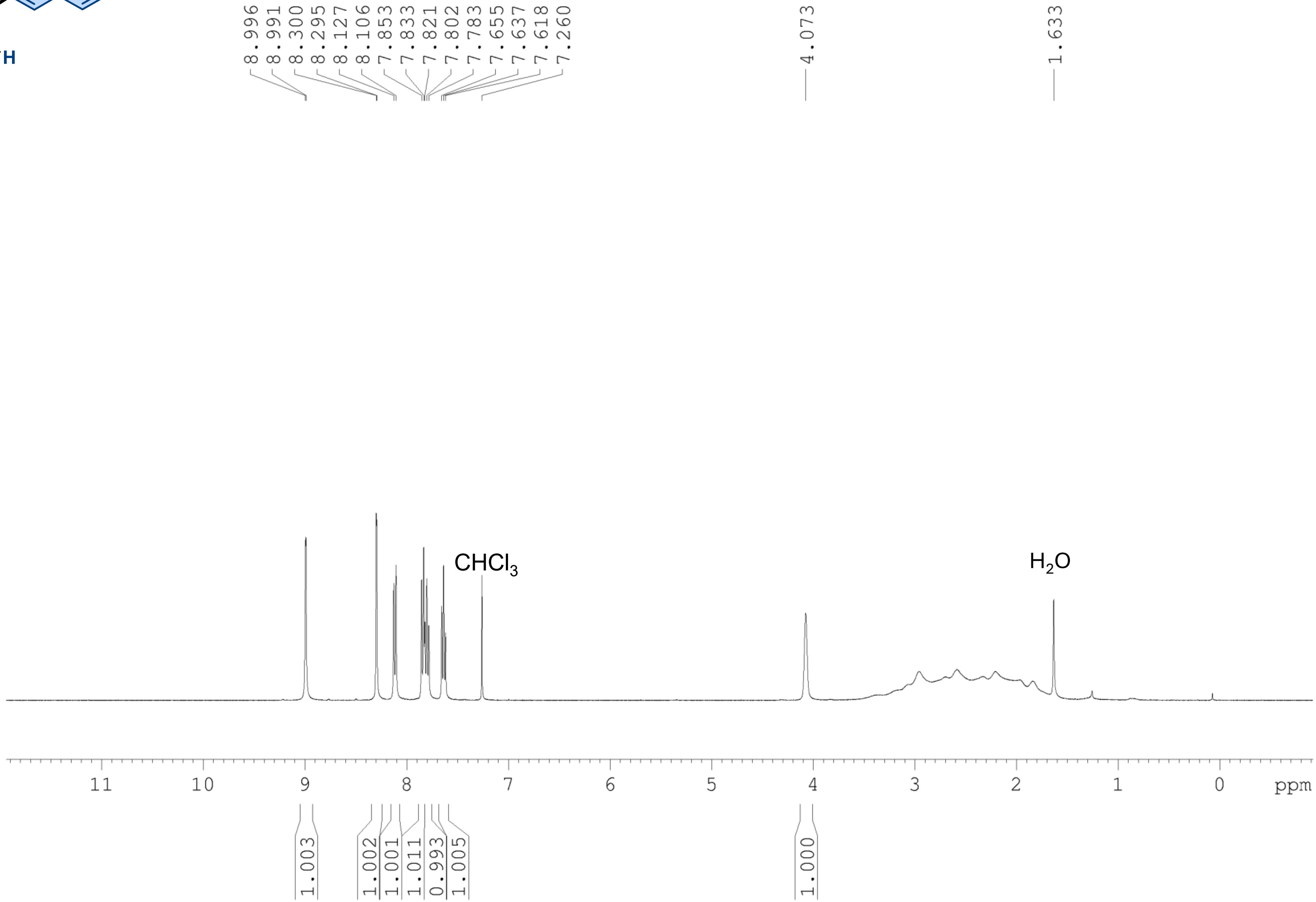

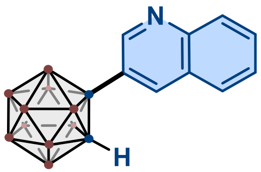

^{11}B NMR, (128 MHz, $CDCl_3$)

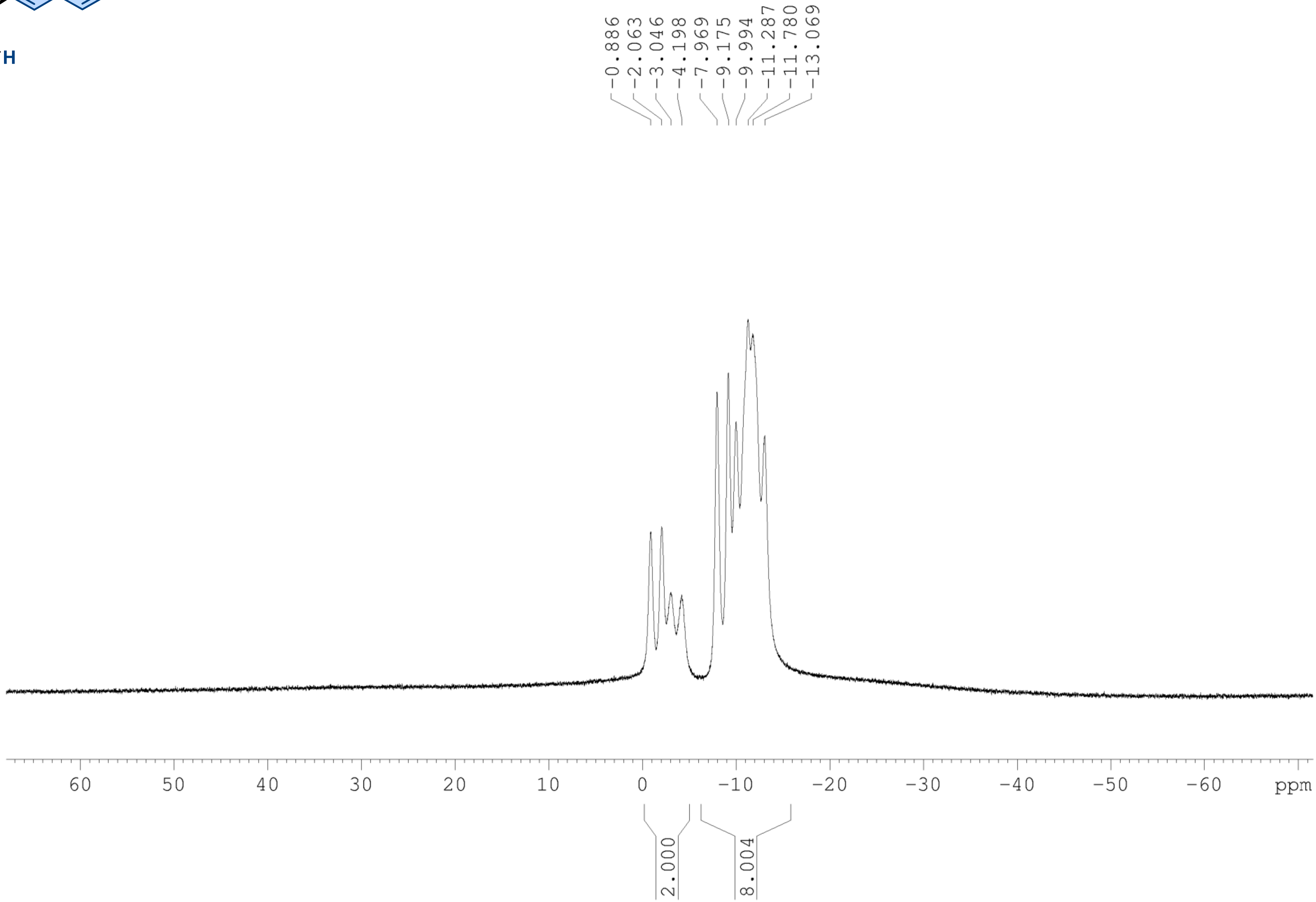

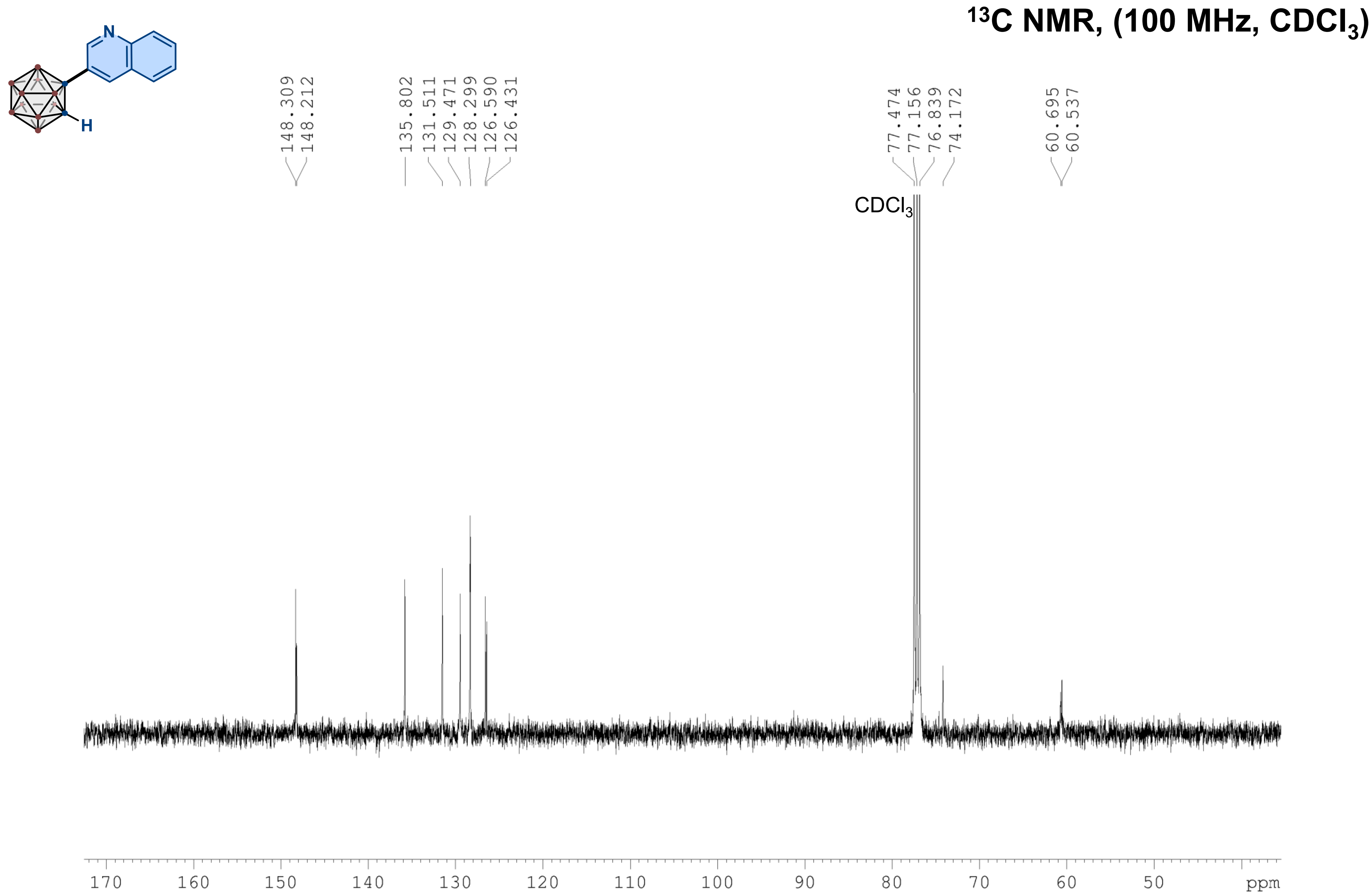

13C NMR, (100 MHz, CDCl3)
N
H
148.309
148.212
135.802
131.511
129.471
128.299
126.590
126.431
77.474
77.156
76.839
74.172
60.695
60.537
CDCl3
170
160
150
140
130
120
110
100
90
80
70
60
50
ppm

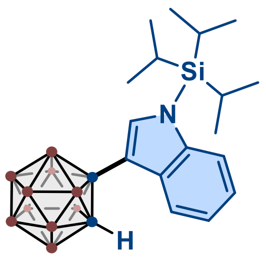

^1H NMR, (400 MHz, $CDCl_3$)

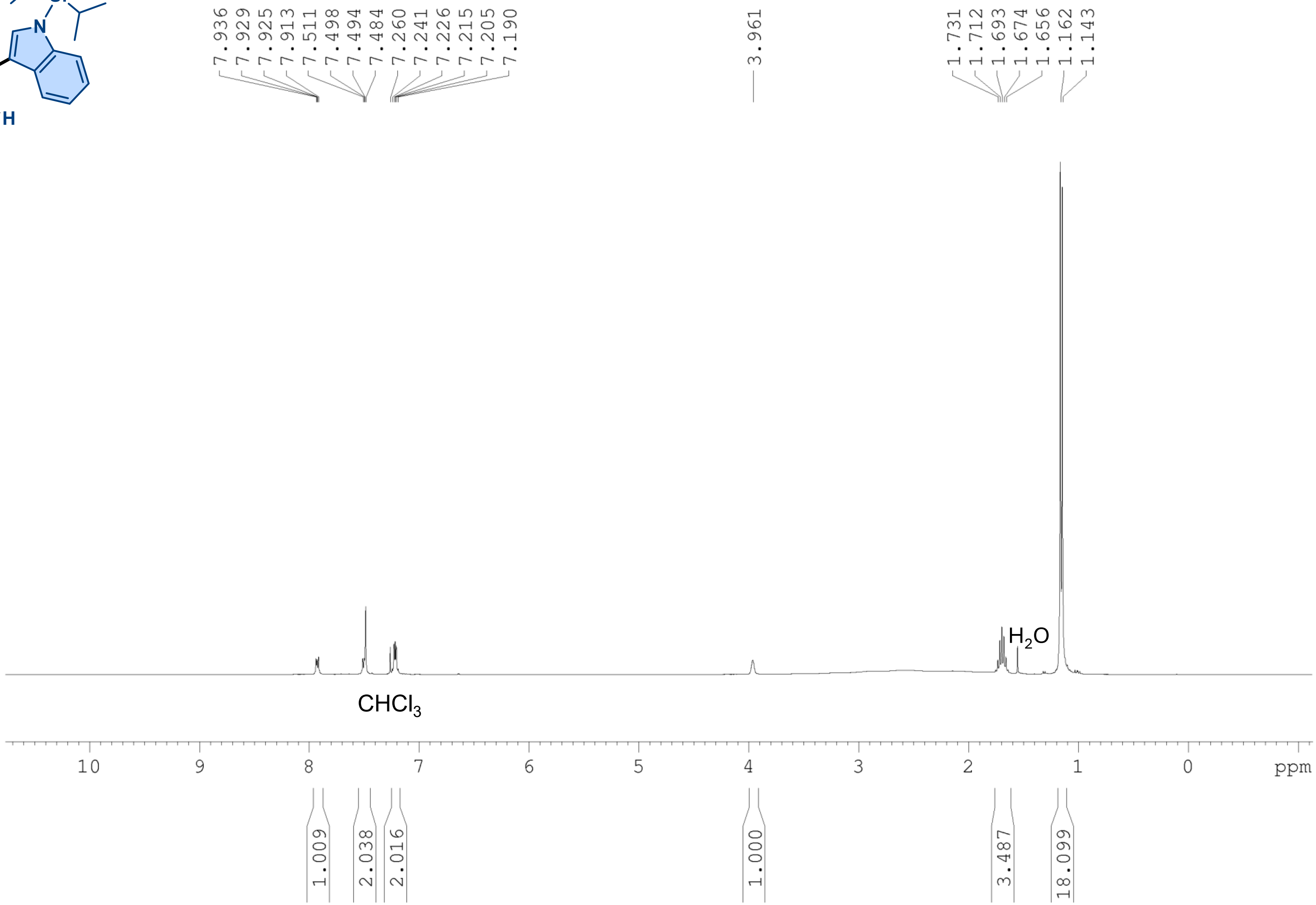

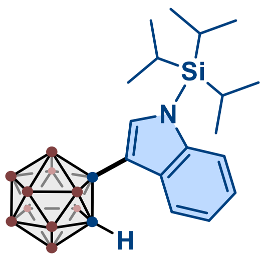

^{11}B NMR, (128 MHz, $CDCl_3$)

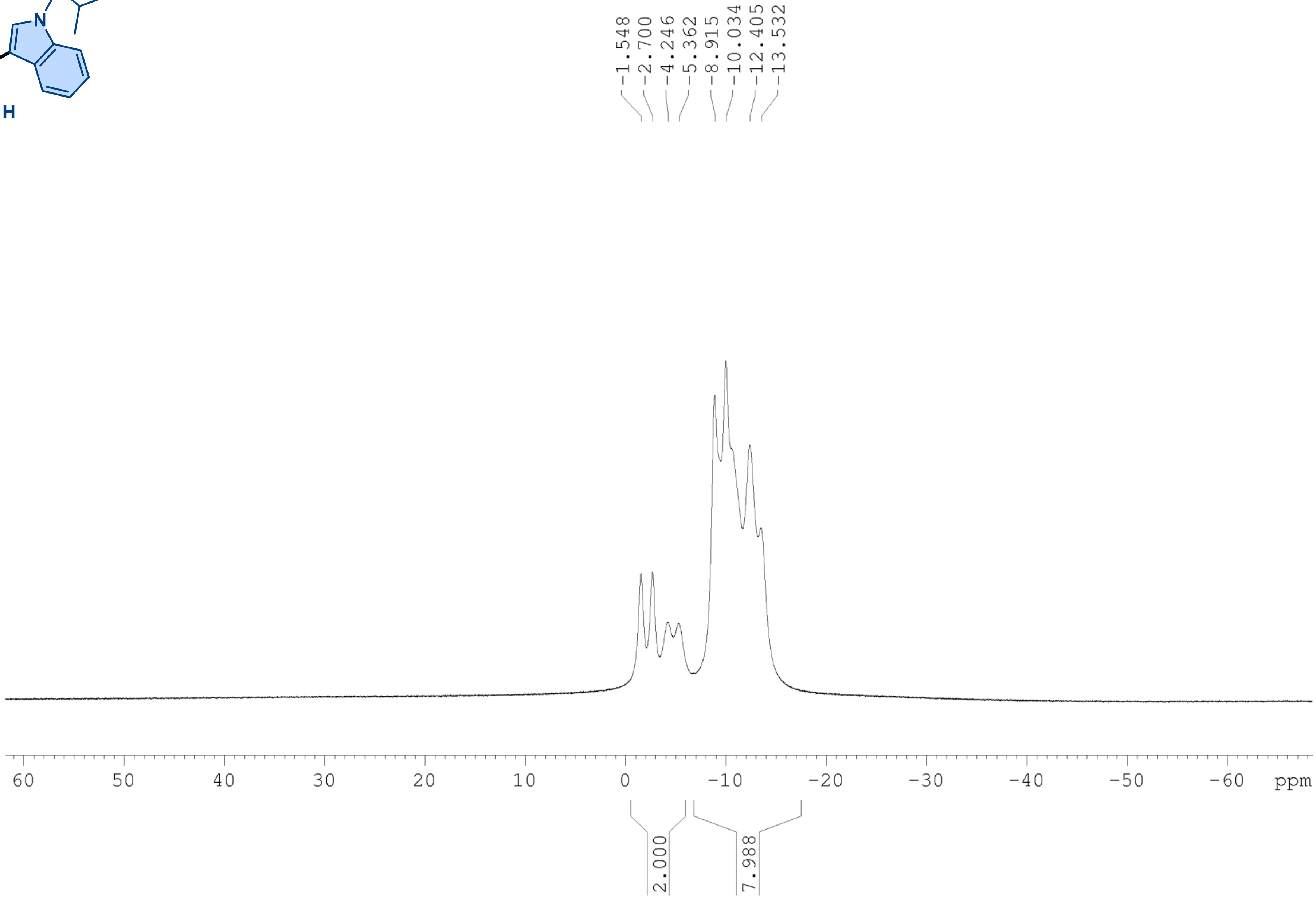

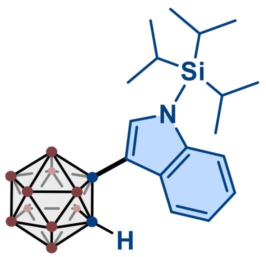

^{13}C NMR, (100 MHz, $CDCl_3$)

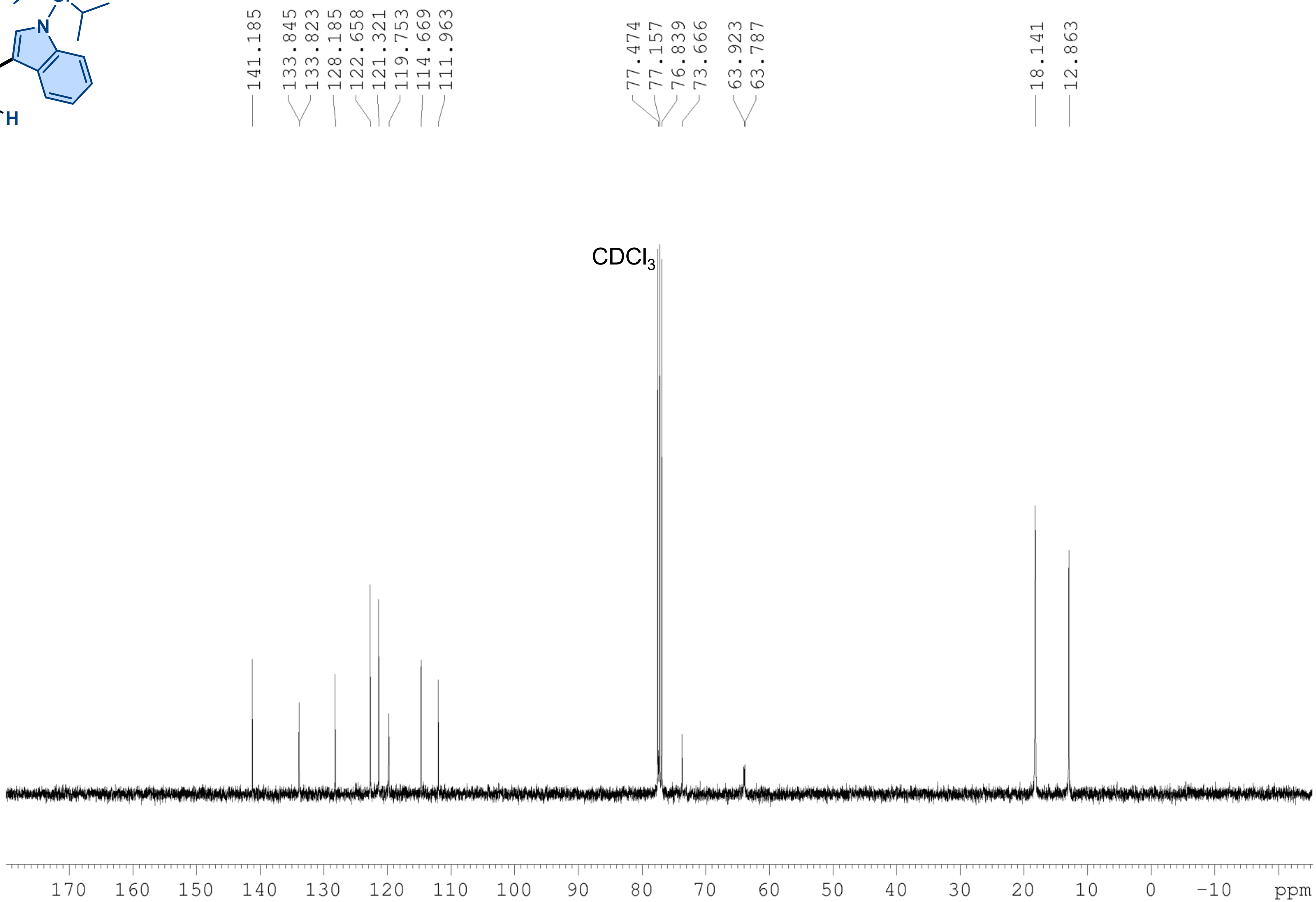

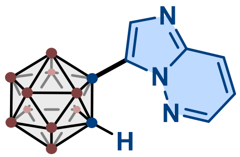

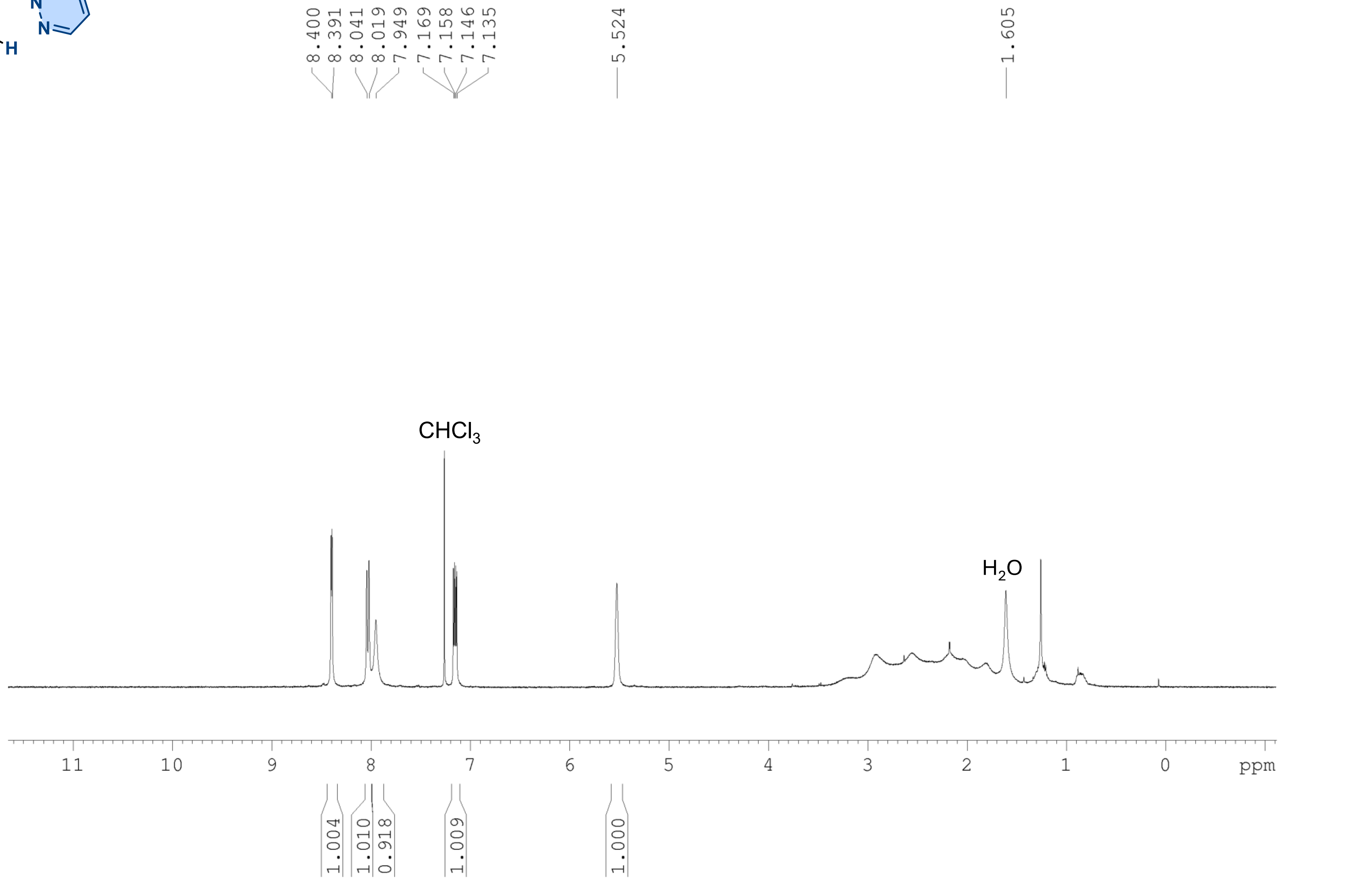
1H NMR, (400 MHz, CDCl3)
8.400
8.391
8.041
8.019
7.949
7.169
7.158
7.146
7.135
5.524
1.605
CHCl3
H2O
11
10
9
8
7
6
5
4
3
2
1
0
ppm
1.004
1.010
0.918
1.009
1.000

^{11}B NMR, (128 MHz, $CDCl_3$)

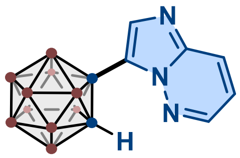

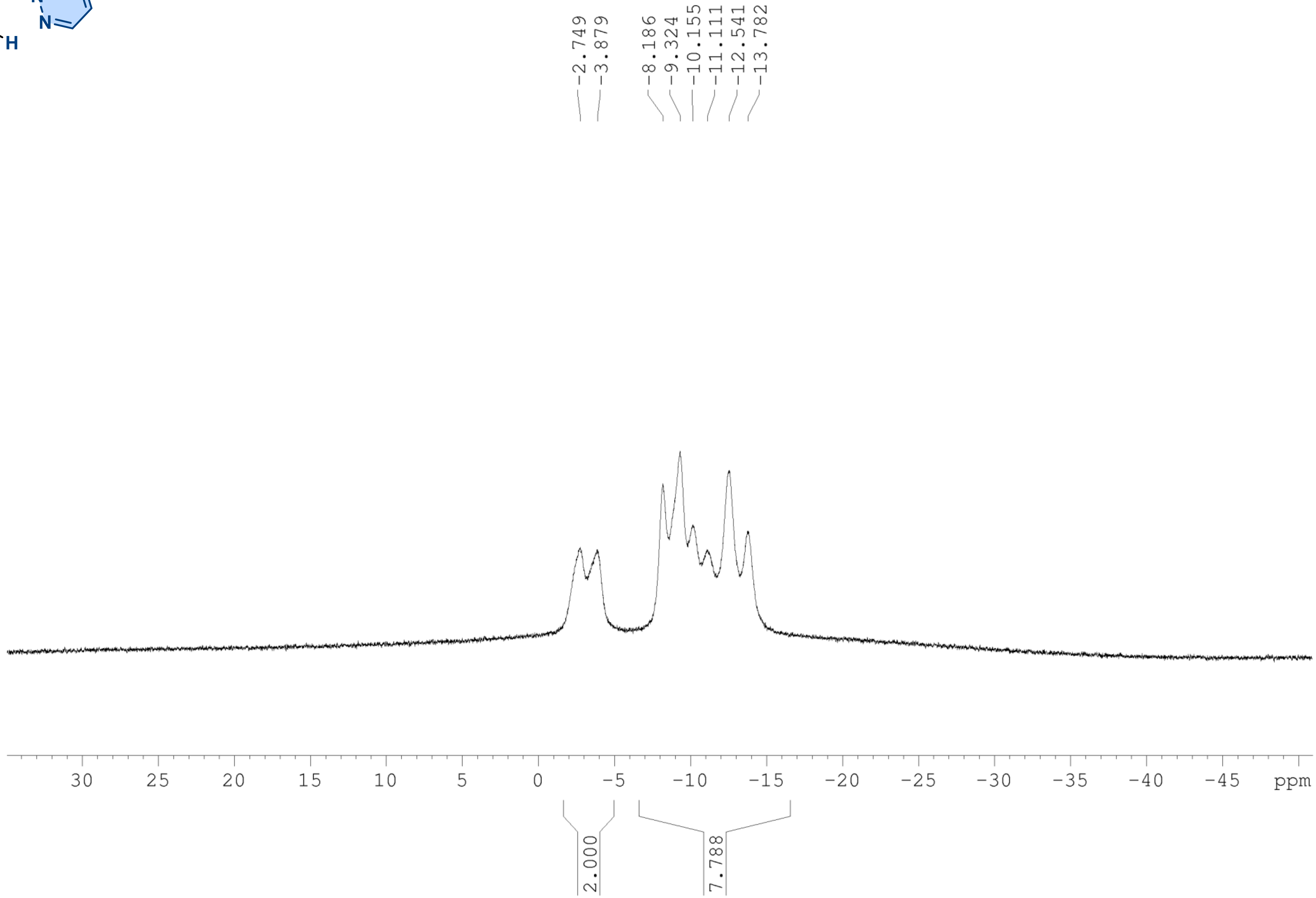

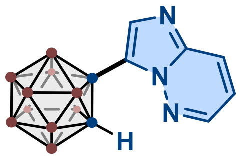

^{13}C NMR, (100 MHz, DMSO-d_6)

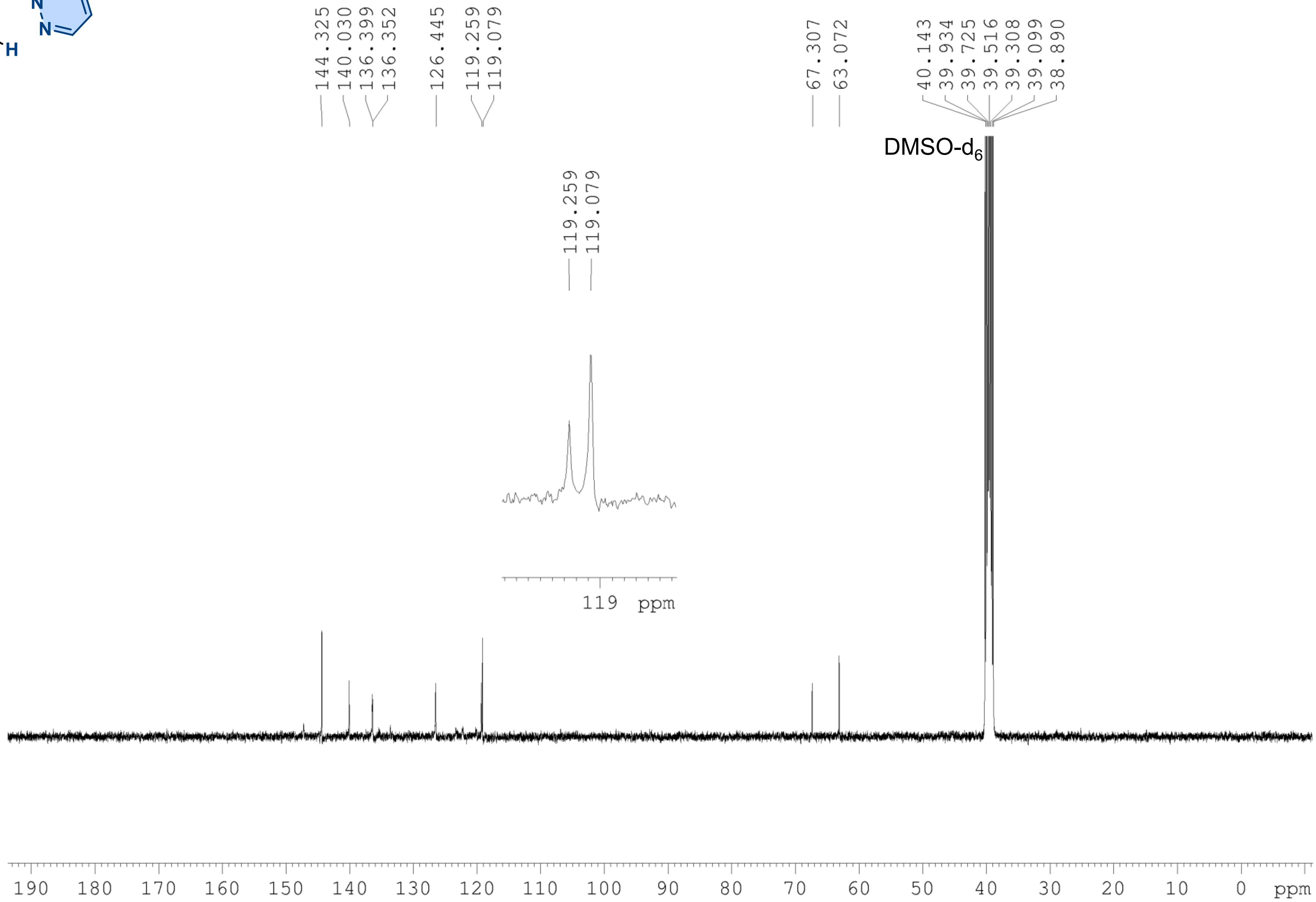

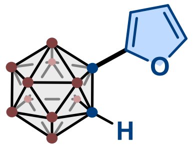

^1H NMR, (400 MHz, $CDCl_3$)

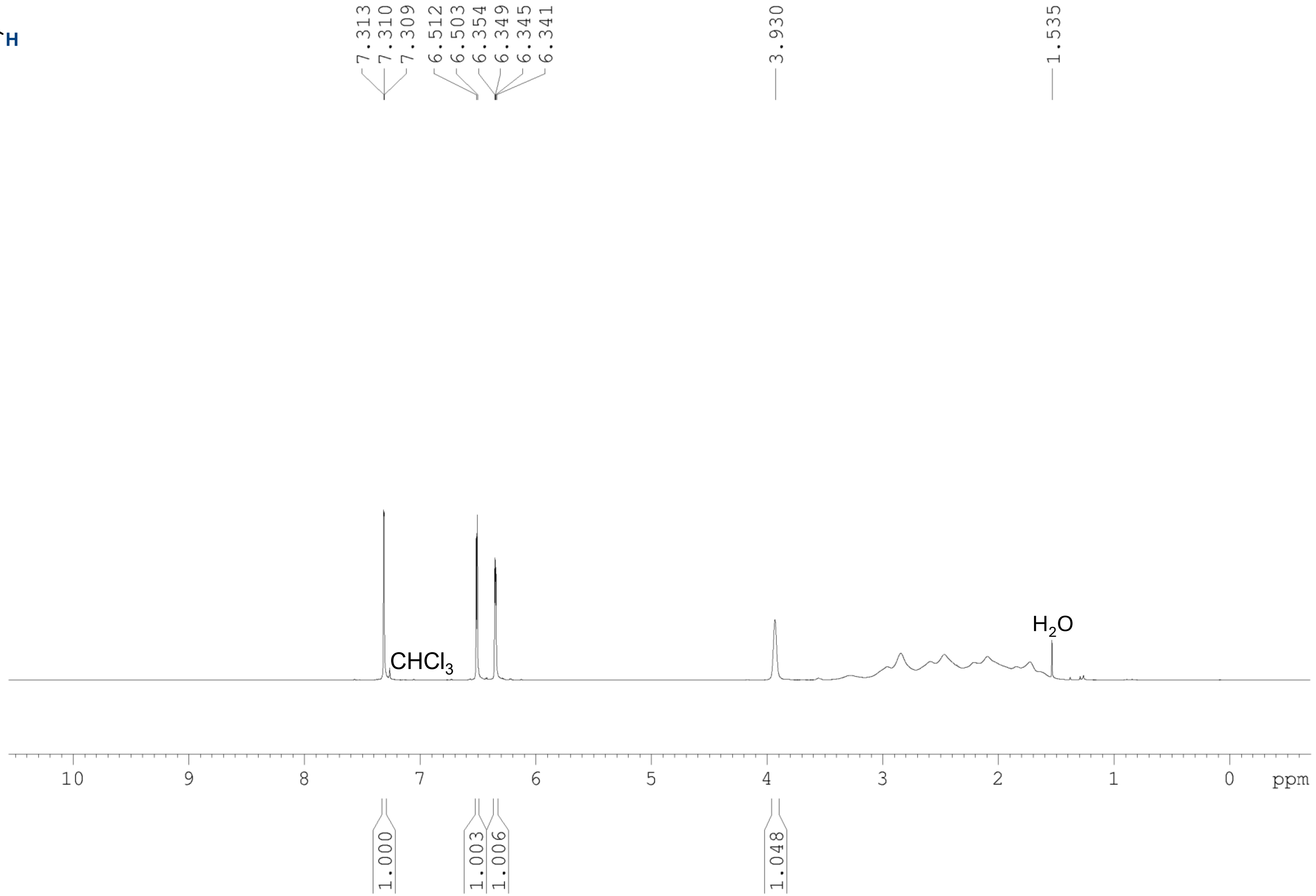

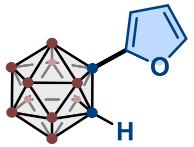

^{11}B NMR, (128 MHz, $CDCl_3$)

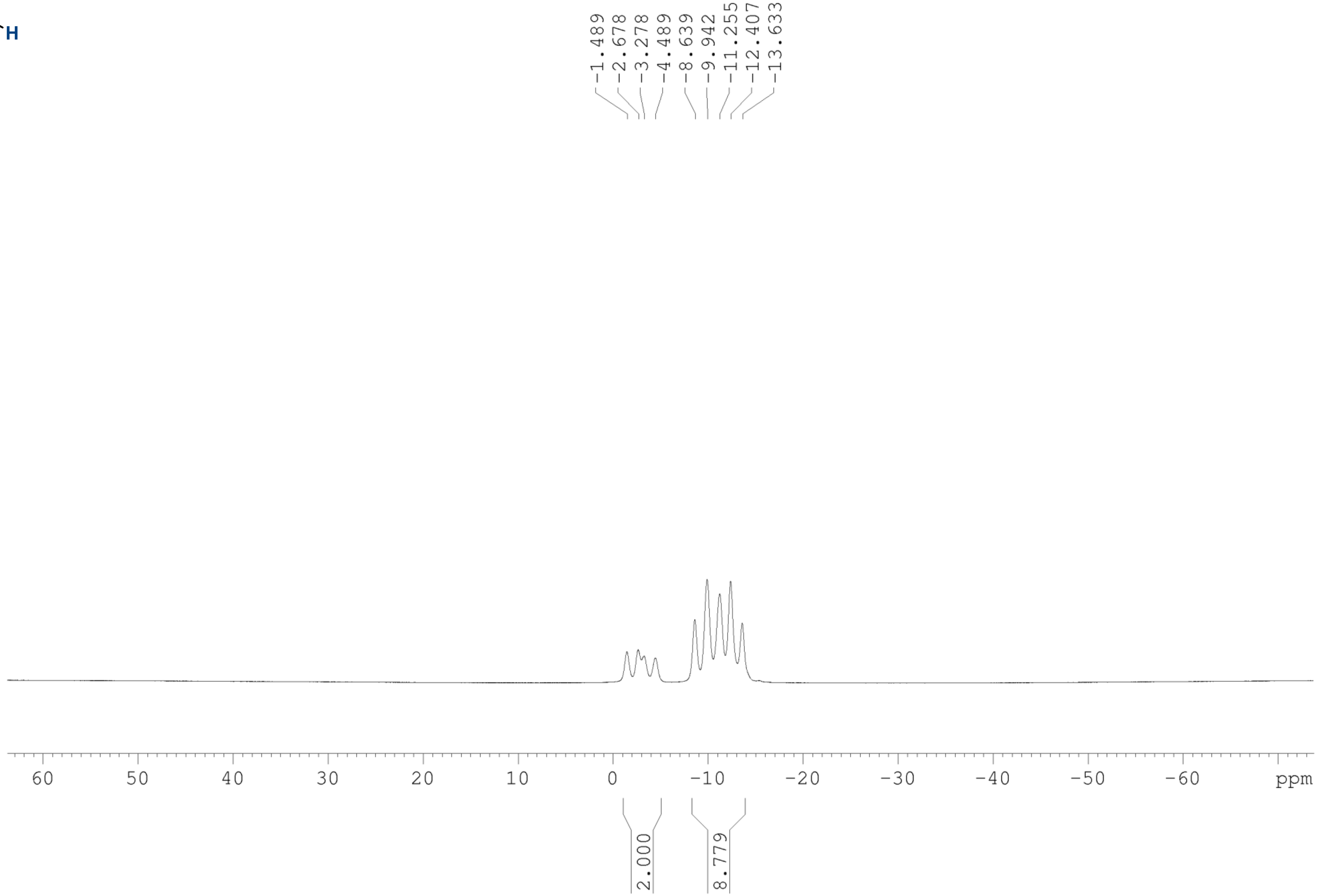

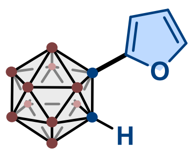

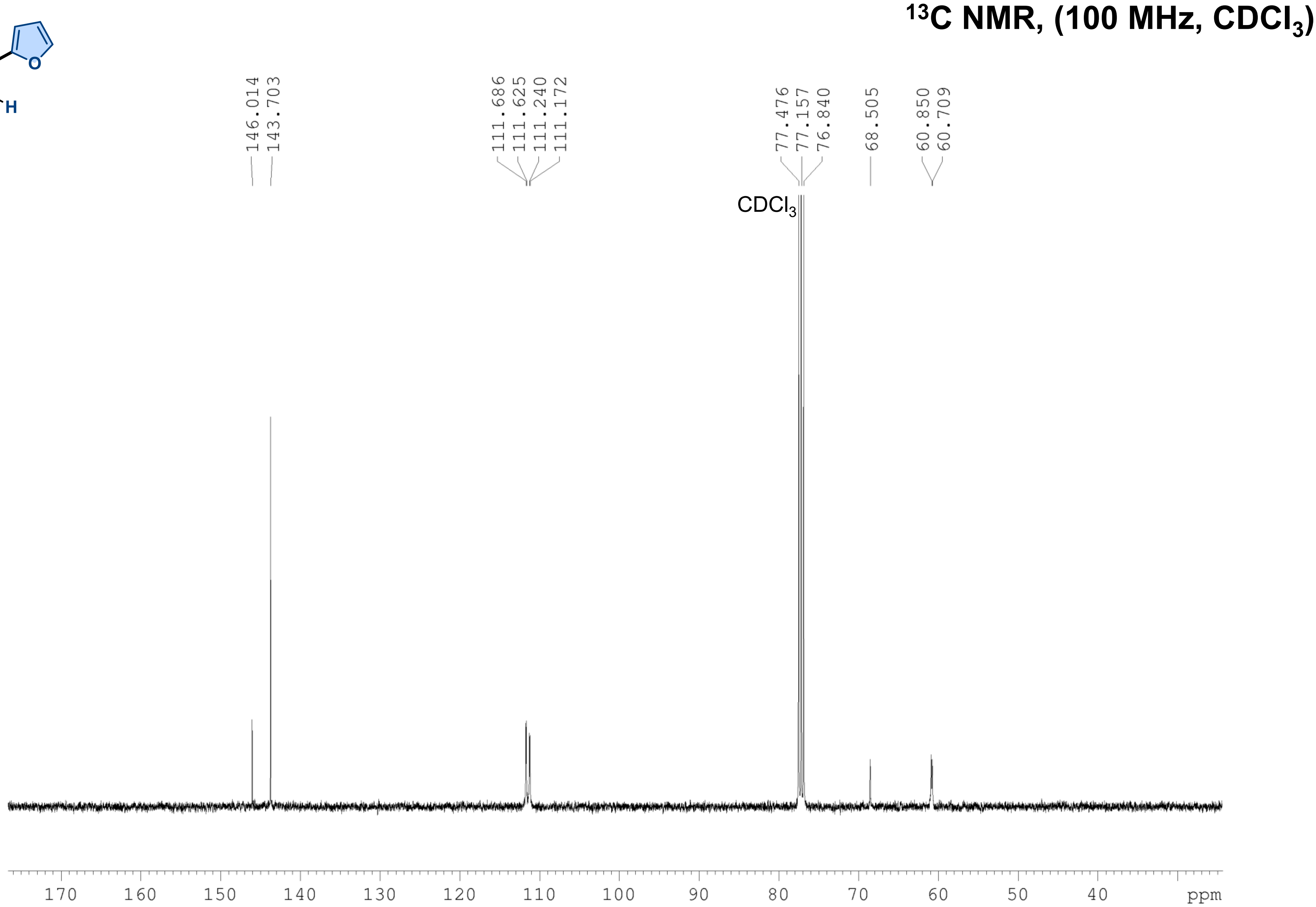

^{13}C NMR, (100 MHz, $CDCl_3$)
146.014
143.703
111.686
111.625
111.240
111.172
77.476
77.157
76.840
68.505
60.850
60.709
$CDCl_3$
170
160
150
140
130
120
110
100
90
80
70
60
50
40
ppm

^1H NMR, (400 MHz, $CDCl_3$)

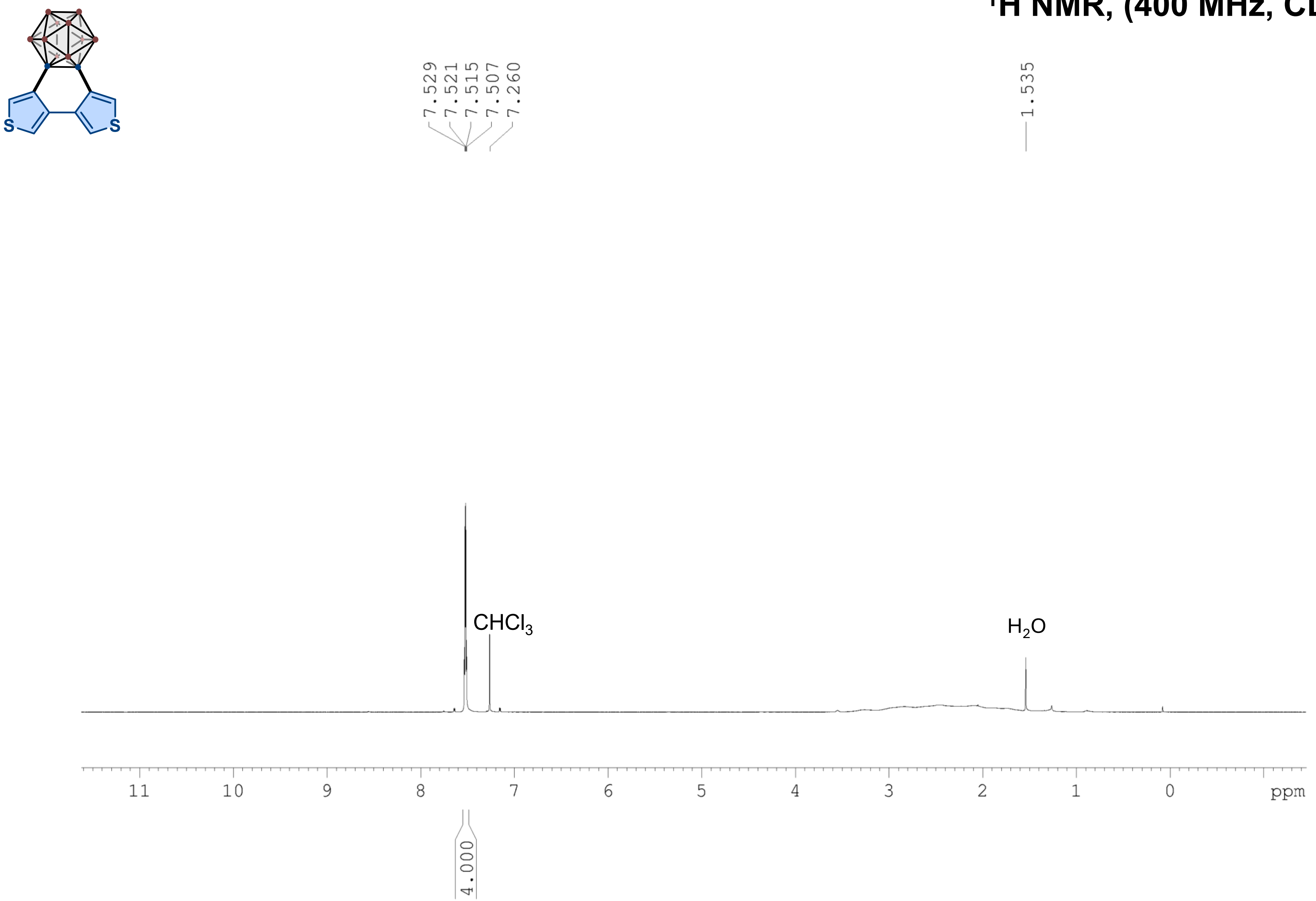

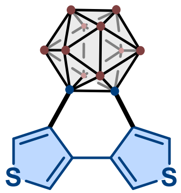

^{11}B NMR, (128 MHz, $CDCl_3$)

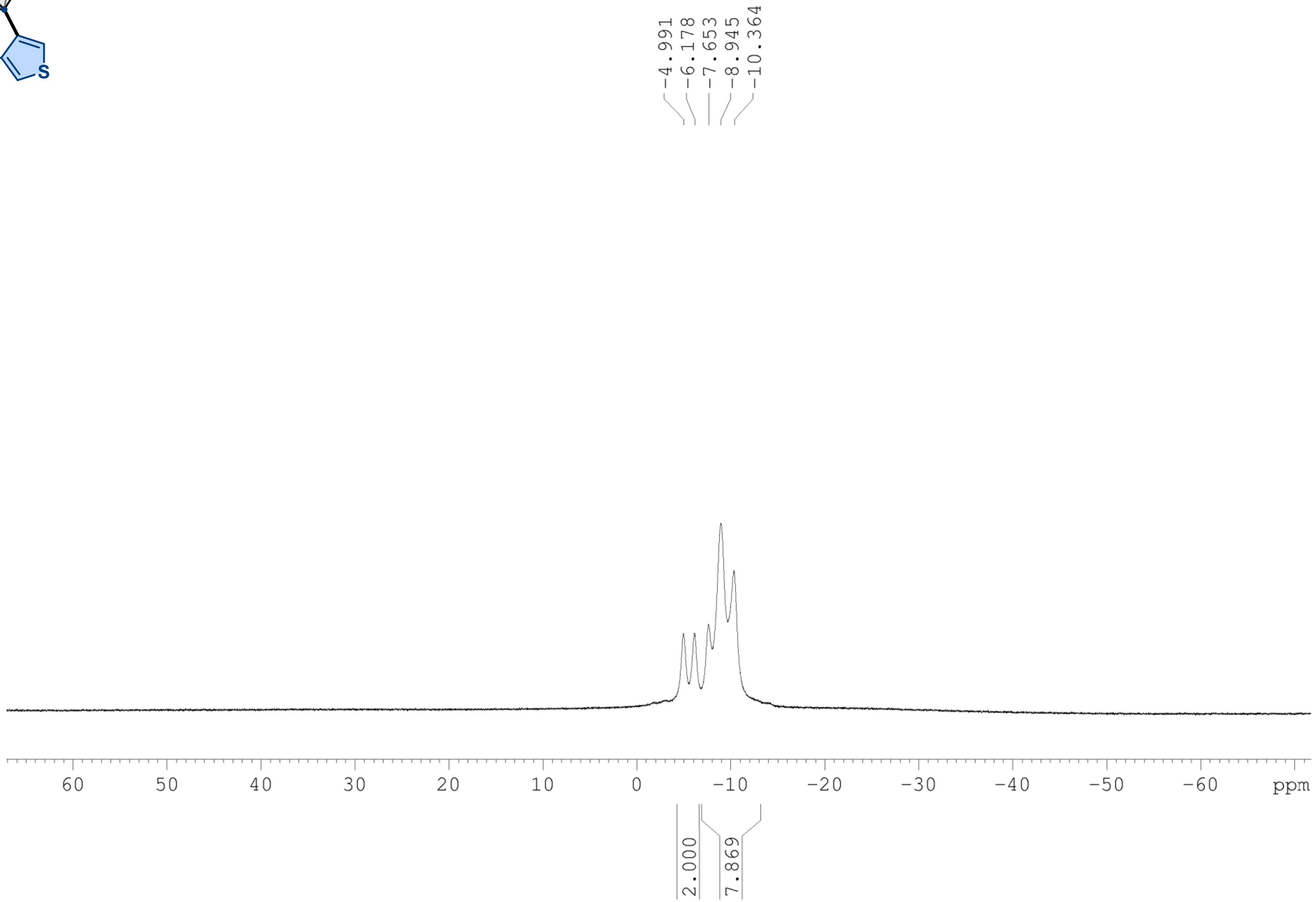

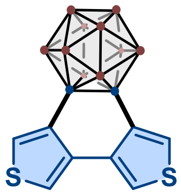

^{13}C NMR, (100 MHz, $CDCl_3$)

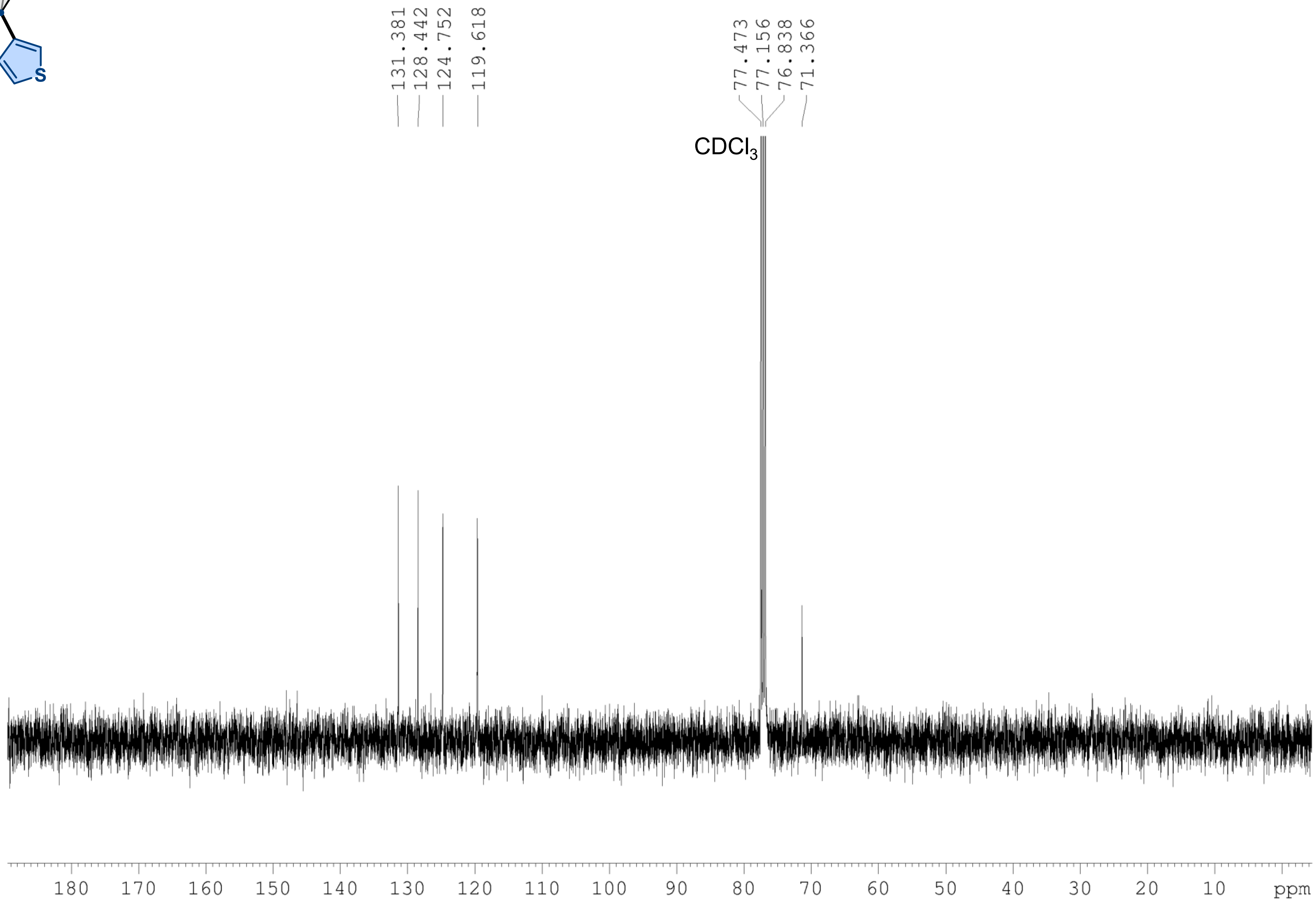

Supplement: Supplementary file 1 [file ja5c13004_si_001.pdf]
